# Copper‐Catalyzed Asymmetric Silylation of Propargyl Dichlorides: Access to Enantioenriched Functionalized Allenylsilanes

**DOI:** 10.1002/anie.201908343

**Published:** 2019-10-04

**Authors:** Zheng‐Li Liu, Chao Yang, Qi‐Yan Xue, Meng Zhao, Cui‐Cui Shan, Yun‐He Xu, Teck‐Peng Loh

**Affiliations:** ^1^ Department of Chemistry University of Science and Technology of China Hefei Anhui 230026 China; ^2^ Division of Chemistry and Biological Chemistry School of Physical and Mathematical Sciences Nanyang Technological University 637616 Singapore Singapore

**Keywords:** allenes, copper, enantioselectivity, silanes, synthetic methods

## Abstract

A copper‐catalyzed silylation of propargyl dichlorides was developed to access chloro‐substituted allenylsilanes under mild reaction conditions. Moreover, enantioenriched chloro‐substituted allenylsilanes can be synthesized in moderate to high yields and good enantioselectivities with this protocol.

## Graphical abstract

**A copper‐catalyzed** silylation reaction of propargyl dichlorides has been developed. In this protocol, racemic and enantioenriched chloro‐substituted allenylsilanes can be accessed with high efficiency under mild reaction conditions. The corresponding products were transformed into a variety of useful synthons.

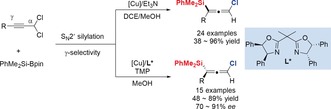

Allenic compounds have attracted much attention because of their unique structural properties and versatile reactivities.^1^ Among them, axially chiral allenylsilanes, especially the enantiomerically enriched form, have been demonstrated as valuable intermediates in the synthesis of complex pharmaceutical compounds and natural products.^2^ Traditionally, pre‐installation of a silyl group in the starting material either by addition to conjugated fragments or S_N_2′ displacement of propargylic alcohol derivatives with organometallic reagents is often employed.^3^ Another strategy is the silylation of propargyl alcohol derivatives with either silylcuprates or silylzincates.^4^ However, issues such as multistep preparation of the starting materials, harsh reaction conditions and/or limited scope etc., remain unsolved. In the past decade, a few examples on catalytic silylation of propargyl alcohol derivatives were reported. In 2009, Sawamura^5^ et al. developed a pioneering Rh‐catalyzed silylation of propargyl carbonates for the synthesis of racemic tri‐ and tetrasubstituted allenylsilanes (Scheme 1 a). Subsequently, an elegant racemic copper‐catalyzed γ‐selective silylation of propargyl chlorides and chiral propargyl phosphates was reported by Oestreich et al. using Me_2_PhSi‐Bpin and (Me_2_PhSi)_2_Zn (Scheme 1 b).^6^ To the best of our knowledge, there is no precedence on the synthesis of enantioenriched chiral allenylsilanes by catalytic propargyl silyl substitution. To address this gap, we embarked on developing catalytic silylation reactions of propargyl dichlorides, a type of substrate that has received little attention despite their facile preparation. In 2011, Knochel and co‐workers elegantly reported a copper‐mediated S_N_2′ substitution of propargyl dichlorides with organozinc reagents to synthesize the chloroallenes.^7^ After that, Alexakis et al. studied the highly enantioselective Cu‐catalyzed 1,3‐substitution of dichloropropargyl substrates with Grignard reagents to form chiral chloroallenes.^8^ Inspired by these pioneering works,^9^ and based on our interest in copper‐catalyzed C−Si bond formation,^10^ herein we communicate the preparation and application of enantioenriched chloro‐substituted allenylsilanes (Scheme 1 c).

**Scheme 1:**
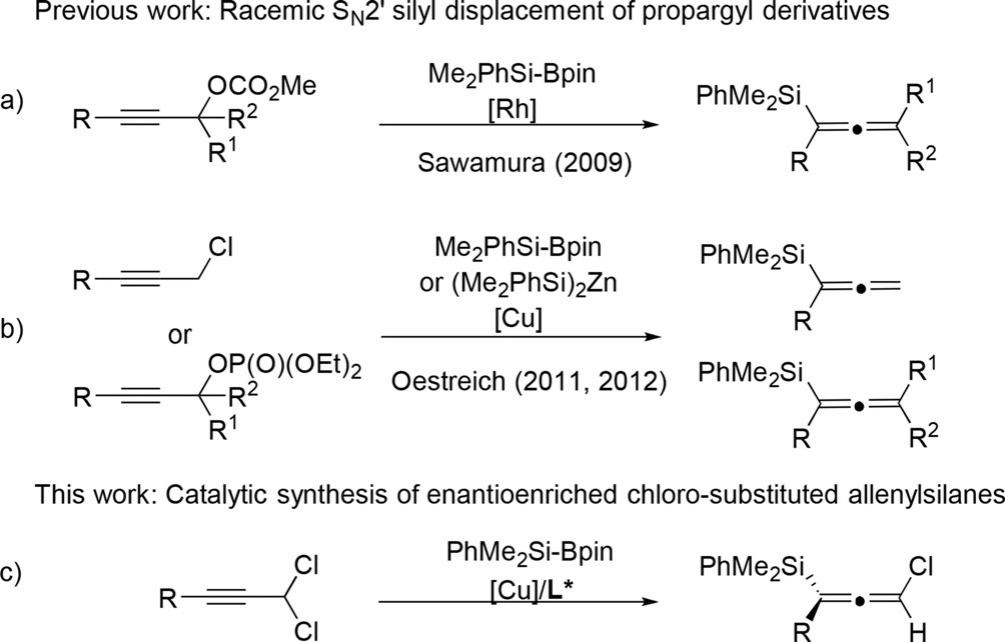
Copper‐catalyzed silylation reactions of propargyl derivatives.

Initially, the racemic copper‐catalyzed 1,3‐silyl‐substitution of propargyl dichlorides was investigated. After careful screening of catalysts, bases, solvents, and temperature (see the Supporting Information for details), the optimized reaction conditions were determined as follows: a mixture of **1 a** and **2** (for structures see Table 1) in DCE/MeOH (2:1) was stirred at −10 °C for 2 hours with 5 mol % CuI as the catalyst and 2 equivalents of Et_3_N as an additive under Ar.

Different propargyl dichlorides were subjected to this 1,3‐silyl substitution reaction under the optimal reaction conditions (Table 1). Various propargyl dichlorides, irrespective the electronic properties of the substituents on the phenyl ring afforded the desired products in moderate to good yields (**3 a**–**l**). Moreover, substrates bearing aliphatic substituents were also smoothly converted into their respective products (**3 m**–**v**). Notably, good to high yields were also obtained for the substrate bearing a bulky substituent such as either a cyclohexyl (**3 p**) or *tert*‐butyl group (**3 q**). Other functional groups such as chloro and alkoxy gave the target products, **3 s** and **3 t**, respectively, in excellent yields. However, the products **3 w** and **3 x** were only obtained in 38 and 45 % yields, respectively, from the corresponding trimethylsilyl‐ and cyclohexenyl‐substituted substrates.

**Table 1:** Investigation of different propargyl dichlorides for the synthesis of chloro‐substituted allenylsilanes.^[a,c]^

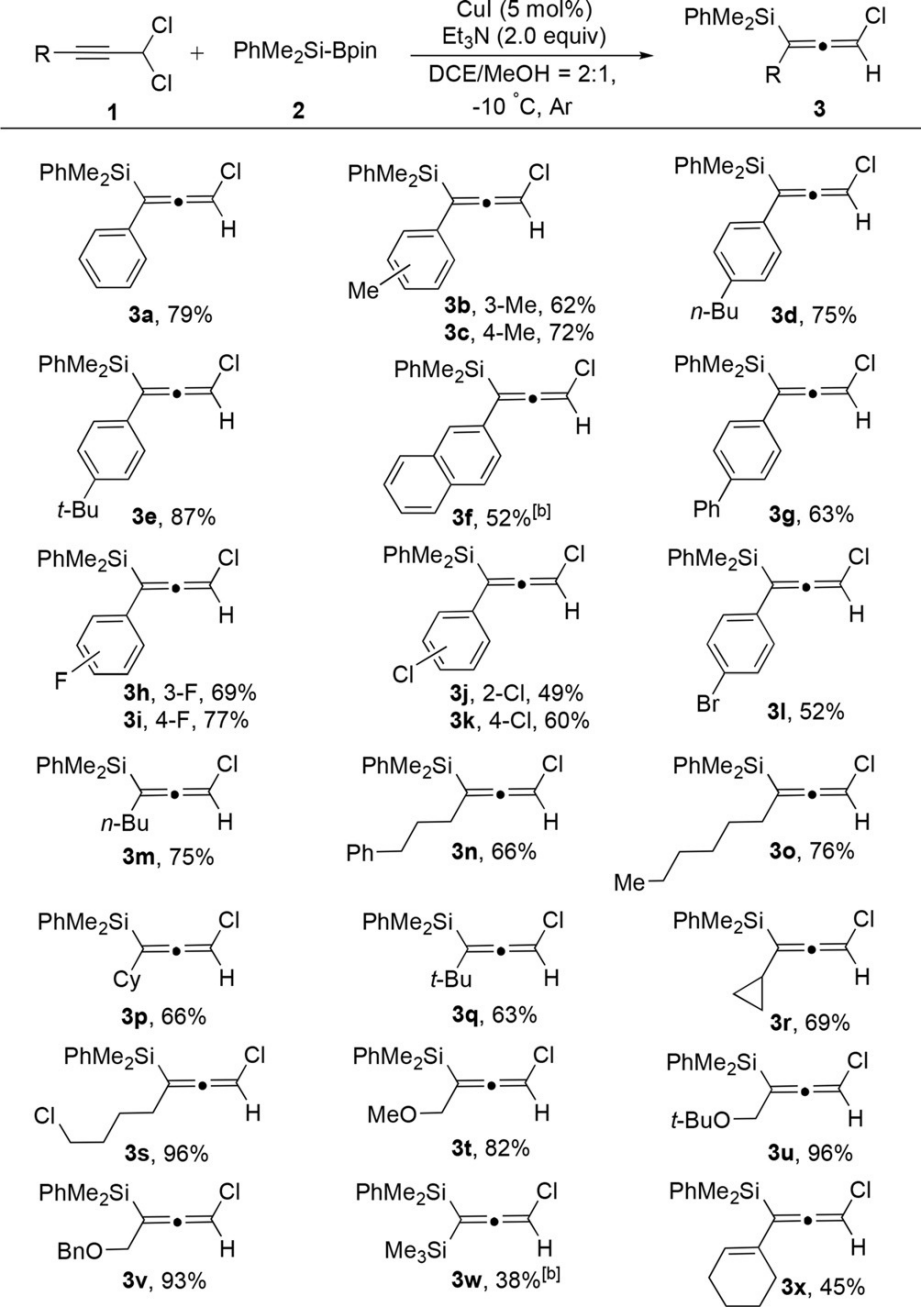

[a] Reaction was performed according to the following reaction conditions: Under argon atmosphere, the mixture of **1** (0.2 mmol), **2** (0.4 mmol, 2.0 equiv), CuI (0.05 mmol, 5 mol %), and Et_3_N (0.4 mmol, 2.0 equiv) in the indicated dry solvent (1.5 mL) was stirred for the corresponding time at −10 °C. [b] The catalyst loading was 10 mol %. [c] Yield is that of isolated product.

Compared to the catalytic allylic silyl substitution reactions,10d, 10e, 11 only few examples of an S_N_2′ silylation reaction of propargyl derivatives have been reported.^5^, ^6^ Therefore, we embarked on studying the catalytic synthesis of optically active chloro‐substituted allenylsilanes (Table 2). After careful screening of various copper catalysts, bases and different bidentate chiral P‐N and chiral oxazoline ligands, we found that the desired enantioenriched product (*S*)‐**3 c** could be obtained in 62 % yield with 90 % enantiomeric excess in the presence of 10 mol % CuF_2_, 20 mol % (4*R*,4′*R*,5*S*,5′*S*)‐2,2′‐(1‐methylethylidene)bis[4,5‐dihydro‐4,5‐diphenyl oxazole as the ligand, and 2 equivalents of 2,2,6,6‐tetramethylpiperidine as an additive in MeOH at −30 °C (entry 14). Under these optimized reaction conditions, other aryl‐substituted propargyl dichlorides were tested (Table 3). The corresponding chiral allenylsilanes could be isolated in moderate to high yields and with good enantioselectivities [(*S*)‐**3 a**–**l**]. When aliphatic substituted propargyl dichlorides were used as the substrates, the corresponding desired products were all obtained in good yields albeit with slightly decreased enantioselectivities in some cases [(*S*)‐**3 m**–**t**].

**Table 2:** Optimization of the reaction conditions for the synthesis of enantioenriched chloro‐substituted allenylsilane (*S*)‐**3 c**.^[a,b]^ 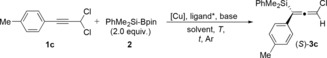

Entry	Cat. (mol %)	Ligand* (mol %)	Base (2.0 equiv)	Solvent	*T* [°C]	Yield [%]	*ee* ^[c]^ [%]
1	CuI (10)	**L_1_** (12)	Et_3_N	MeOH	−10	29	47
2	CuI (10)	**L_2_** (12)	Et_3_N	MeOH	−10	7	50
3	CuI (10)	**L_3_** (12)	Et_3_N	MeOH	−10	91	0
4	CuI (10)	**L_4_** (12)	Et_3_N	MeOH	−10	17	10
5	CuI (10)	**L_6_** (12)	Et_3_N	MeOH	−10	32	10
6	CuI (10)	**L_7_** (12)	Et_3_N	MeOH	−10	68	8
7	CuI (10)	**L_8_** (12)	Et_3_N	MeOH	−10	27	2
8	CuI (10)	**L_2_** (12)	Et_3_N	EtOH	−10	12	40
9	CuI (10)	**L_2_** (12)	PPD	MeOH	−10	22	52
10	CuI (10)	**L_5_** (12)	PPD	MeOH	−10	61	60
11	CuI (10)	**L_5_** (12)	TMP	MeOH	−10	61	66
12	CuI (10)	**L_5_** (12)	TMP	MeOH	−30	53	74
13	CuF_2_ (10)	**L_5_** (12)	TMP	MeOH	−30	60	86
14	CuF_2_ (10)	**L_5_** (20)	TMP	MeOH	−30	62	90
15	CuF_2_ (5)	**L_5_** (10)	TMP	MeOH	−30	62	84
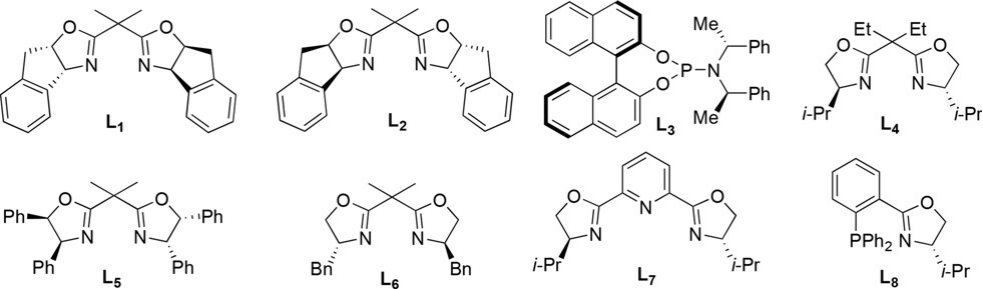							

[a] Reaction was run under the following reaction conditions: **1 c** (0.2 mmol), **2** (0.4 mmol, 2.0 equiv), base (0.4 mmol, 2.0 equiv), 10 mol % copper catalyst, and ligand in 1.0 mL of either anhydrous MeOH or EtOH at indicated temperature under argon atmosphere for corresponding time. [b] Yield of isolated product. [c] The *ee* values were determined by HPLC analysis. PPD=piperidine, TMP=2,2,6,6‐tetramethylpiperidiene.

**Table 3:** Copper‐catalyzed enantioselective synthesis of chloro‐substituted allenylsilanes.^[a,b]^

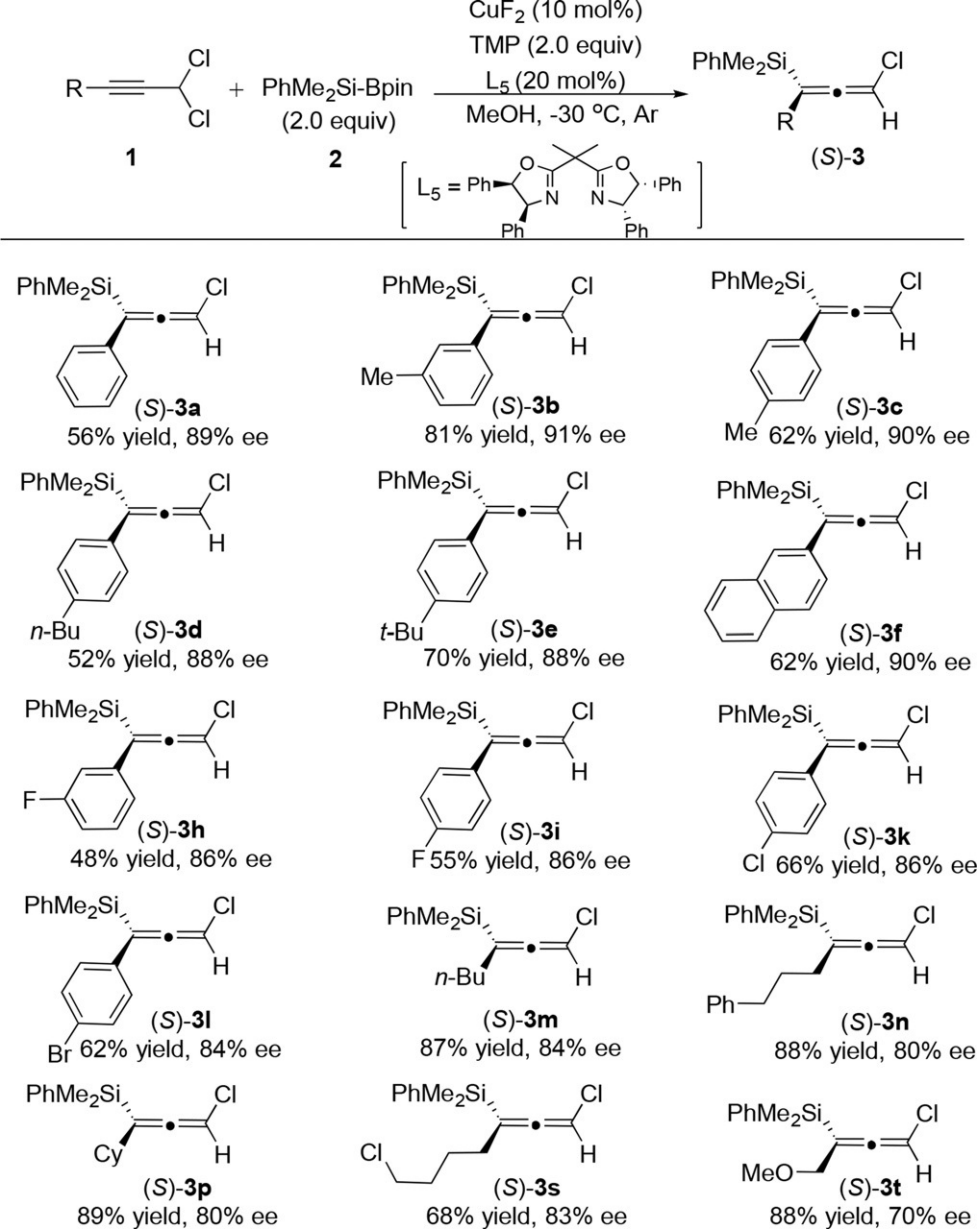

[a] Reaction was performed according to the following reaction conditions: Under argon atmosphere, the mixture of **1** (0.2 mmol), **2** (0.4 mmol, 2.0 equiv), CuF_2_ (0.01 mmol, 10 mol %), TMP (0.4 mmol, 2.0 equiv), and ligand **L_5_** (0.04 mmol, 20 mol %) in anhydrous MeOH (1.0 mL) was stirred for corresponding time at −30 °C. [b] Yield is that of isolated product.

To further evaluate the synthetic applicability of chloro‐substituted allenylsilanes, we examined the C−Cl bond functionalization.^12^ Firstly, the asymmetric disilylation of propargyl dichloride was performed to synthesize the enantioenriched allenylsilane in one pot. The desired product **6** was obtained in 74 % yield and 92 % *ee* (Scheme 2).^13^ In contrast, the enantioenriched (*S*)‐**3 p** was also used for different C−C bond‐formation reactions (Scheme 3). For instance, the alkynylation product **7** could be obtained in 52 % yield by Sonogashira coupling with retention of the enantioselectivity.8b Likewise, Kumada and Suzuki coupling reactions using (4‐(methoxycarbonyl)phenyl)magnesium chloride and potassium vinyltrifluoroborate afforded the products **8** (84 %) and **9** (88 %), respectively, without erosion of the *ee* value.8b According to the previously established methods on copper‐catalyzed substitution of chloroallenes,^7^, ^8^ an ethyl group could also be introduced to the allene **10** in good yield. In addition, to determine the efficiency of axial to central chirality transfer, the TiCl_4_‐mediated addition of **10** to pentafluorobenzaldehyde was performed. The homopropargylic alcohol **11** was obtained with slightly decreased enantiomeric purity (72 % *ee*).

**Scheme 2:**
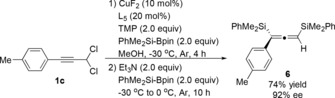
Copper‐catalyzed one‐pot synthesis of enantioenriched disilyl‐substituted allene.

**Scheme 3:**
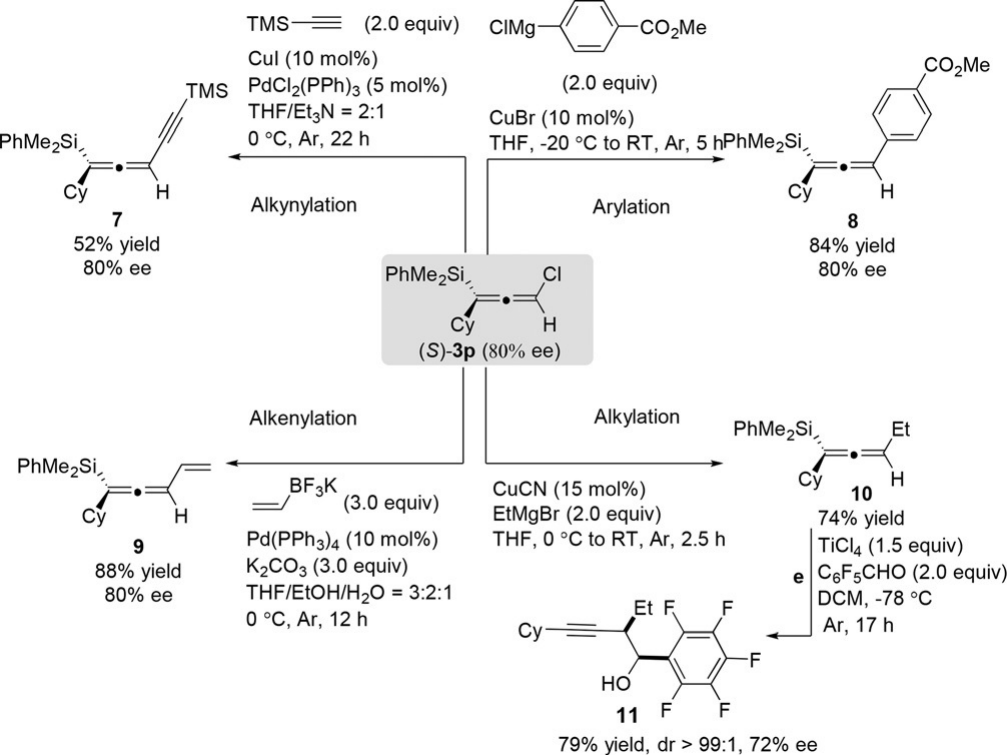
Derivation reactions of chloro‐substituted allenylsilanes.

Based on previous reports^14^ and our results, a plausible mechanistic pathway is proposed (Scheme 4). The Cu‐Si species **A** could be generated from the silylboronate **2**, CuCl, and Et_3_N in methanol. Subsequently, either *anti*‐selective elimination or direct addition process takes place, resulting the formation of either the copper(III) intermediate **C** or copper(I) intermediate **D**, respectively, both of which could be formed from the π‐complex **B**. Either reductive elimination from **C** or β‐elimination from **D** then furnishes the desired allene **3** and releases the copper catalyst for the next catalytic cycle.

**Scheme 4:**
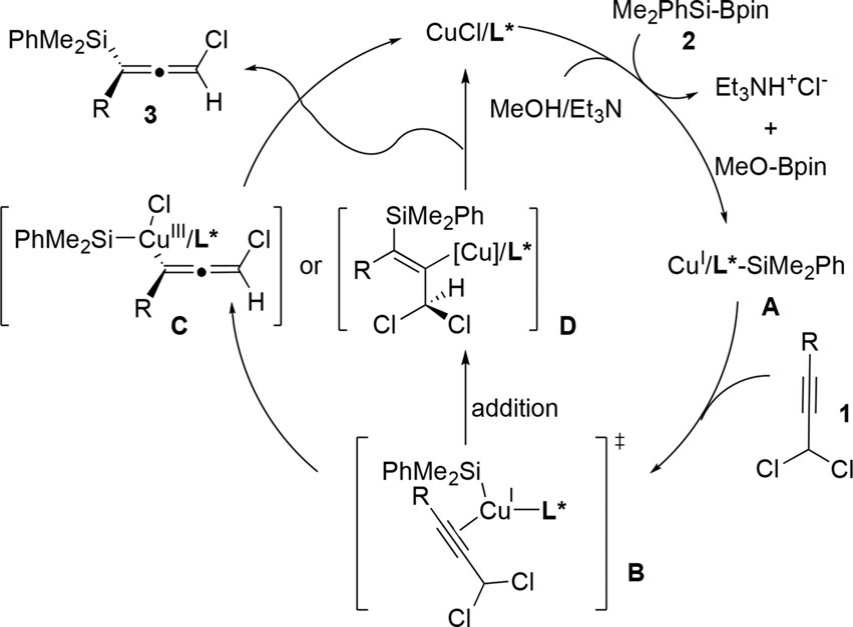
Proposed plausible mechanism for the copper‐catalyzed silylation reaction of propargyl dichlorides.

In conclusion, we have developed a general copper‐catalyzed silylation of propargyl dichlorides. Under the optimized reaction conditions, the racemic and enantioenriched chloro‐substituted allenylsilanes could be achieved by S_N_2′ propargyl silylation process. The facile conversions of the C−Cl bond, including alkynylation, arylation, vinylation, alkylation, and the chirality transfer from the enantioenriched allenylsilane product to homopropargylic alcohol, demonstrate the high synthetic value of this methodology.

## Conflict of interest

The authors declare no conflict of interest.

## Supporting information

As a service to our authors and readers, this journal provides supporting information supplied by the authors. Such materials are peer reviewed and may be re‐organized for online delivery, but are not copy‐edited or typeset. Technical support issues arising from supporting information (other than missing files) should be addressed to the authors.

Supplementary
