# Supplementary material for: Copper‐Catalyzed Asymmetric Silylation of Propargyl Dichlorides: Access to Enantioenriched Functionalized Allenylsilanes
Source: Angew Chem Int Ed Engl. 2019 Oct 4;58(46):16538–42. doi: 10.1002/anie.201908343 (PMC6899605; doi:10.1002/anie.201908343)
Supplement: Supplementary file 1 — Supplementary [file ANIE-58-16538-s001.pdf]

Angewandte Chemie
Eine Zeitschrift der Gesellschaft Deutscher Chemiker

Supporting Information

Copper-Catalyzed Asymmetric Silylation of Propargyl Dichlorides: Access to Enantioenriched Functionalized Allenylsilanes

Zheng-Li Liu+, Chao Yang+, Qi-Yan Xue, Meng Zhao, Cui-Cui Shan, Yun-He Xu, and Teck-Peng Loh*

anie_201908343_sm_miscellaneous_information.pdf

Supporting Information

1 General Information……………………………………………………..S-2

2 General Procedures for the Preparation of Dichloropropargyl Substrates………………..………...S-2

3 Optimization of the Reaction Conditions of Racemic Silyl-substituted Allenes……………..…....S-10

4 General Procedures for the Synthesis of Racemic Silyl-substituted Allenes ……………..............S-10

5 General Procedures for the Synthesis of Enantioenriched Silyl-substituted Allenes S-23

6 General Procedures for the One-pot Synthesis of Enantioenriched Disilyl-substituted Allenes S-30

7 Further Transformations of Silyl-substituted Chloroallenes……………..……..……………..…......S-31

8 Gram-scaled Synthesis of Racemic Silyl-substituted Allenes……………………..…………..…....S-34

9 Determination of the Absolute Configuration of Compound (*S*)-**3p**………………..…………......S-35

10 References………………………………………………………………………………...S-36

11 ^{1}H NMR, ^{19}F NMR, ^{13}C NMR and HPLC Spectra of the Substrates and Products…..…………..S-37

12 Crystal Data for (*E*, *S*)-**14**………………………………………………….…………..…......S-108

1 General Information

Experiments involving air sensitive components were performed in glovebox. Et_3N and DCE were fractionally distilled. Other reagents were commercially purchased and were used as received without further purification for the reactions. Proton nuclear magnetic resonance (^1H NMR) and carbon nuclear magnetic resonance (^{13}C NMR) spectra were performed on a Bruker Advance 400M NMR spectrometer ($CDCl_3$). Chemical shifts ^1H NMR spectra are reported as δ in units of parts per million (ppm) downfield from $SiMe_4$ (δ 0.00 ppm) and relative to the signal of chloroform-*d* (δ 7.26 ppm, singlet). Multiplicities were given as: s (singlet); d (doublet); t (triplet); q (quartet); tt (triplet of triplets); td (triplet of doublets); m (multiplets) and *etc*. The number of protons (n) for a given resonance is indicated by nH. Coupling constants are reported as a *J* value in Hz. Carbon nuclear magnetic resonance spectra (^{13}C NMR) are reported as in units of parts per million (ppm) downfield from $SiMe_4$ (0.00) and relative to the signal of chloroform-*d* (*J* = 77.03, triplet). High resolution mass spectral analysis (HRMS) were performed on Water XEVO G2 Q-TOF (Waters Corporation) and GCT Premier. The enantiomeric excesses were determined by HPLC analysis on Chiral Daicel Chiralpak OD-H, OJ, ID columns. Optical rotations were recorded on an Anton Paar MCP 200 polarimeter at 589 nm in dichloromethane.

Experimental Procedure：

2 General Procedures for the Preparation of Dichloropropargyl Substrates（GP1）.

All substrates were prepared according to the reported literatures.[1-3]

The general procedure A[1]

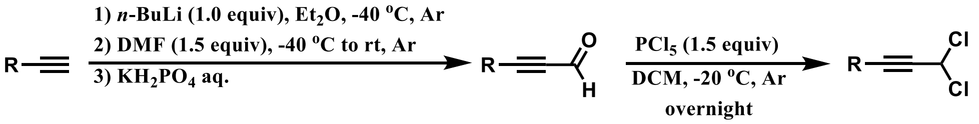

Step 1: To a solution of 1-alkyne (2.61 mL, 20 mmol) in dry Et_2O (20 mL) at -40 ℃ was added dropwise 2.5 M *n*-BuLi (12.5 mL, 20 mmol) followed by the addition of anhydrous DMF (2.4 mL, 30 mmol) in one portion. The clear reaction mixture was allowed to warmed up to room temperature and stirred until full conversion (TLC monitoring). The solution was then poured into a biphasic mixture of 100 mL 10% KH_2PO_4 aqueous solution and ether (80 mL) at 0 ℃. The mixture was stirred vigorously, and layers were partitioned. The aqueous layer was extracted with ether (3 x 50 mL). The organic layers were collected, combined, dried over Na_2SO_4, filtered and concentrated *in vacuo*. The crude product obtained was used in the following chloration step without further purification.

Step 2: The crude product was dissolved in dry CH_2Cl_2 (40 mL) and cooled to -20 ℃. Then, PCl_5 (6.2 g, 30 mmol) was added portion wise and the reaction mixture was stirred overnight. It was quenched by the addition of saturated $NaHCO_3$ solution at -20 ℃. The mixture allowed to warm back to 25 ℃, water was added to completely neutralize the solution. The aqueous phase was extracted by CH_2Cl_2 (3 x 50 mL), the organic phases were collected, dried over Na_2SO_4, concentrated under reduced pressure. Purification by column chromatography on

silica gel (eluent: petroleum ether) provided the desired compound.

The general procedure B [2]

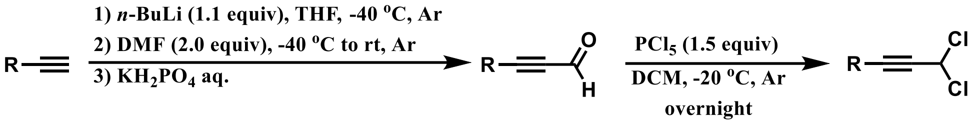

Step 1: A dried, argon-flushed flask equipped with a magnetic stirring bar was charged with dry THF (40 mL) and ethynylcyclohexane (4.33 g, 40 mmol). The solution was cooled to -40 ℃ and *n*-BuLi (17.6 mL, 44 mmol, 2.5 M in hexane) was added dropwise. The reaction mixture was stirred for 1 hour at -40 ℃ followed by the addition of dry DMF (5.85 g, 80.0 mmol). Then, the reaction mixture was allowed to warm up to room temperature until the starting material was completely consumed (monitored by TLC). The solution was then poured into a biphasic mixture of 200 mL 10% KH_2PO_4 aqueous solution and ether (160 mL) at 0 ℃. The mixture was stirred vigorously, and layers were partitioned. The aqueous layer was extracted with ether (3 x 50 mL). The organic layers were collected, combined, dried over Na_2SO_4, filtered and concentrated *in vacuo*. The residue was purified by column chromatography (eluent: PE/EA = 95:5) to afford the product (3.37 g) as colorless oil.

Step 2: The above product was dissolved in dry CH_2Cl_2 (40 mL) and cooled to -20 ℃. Then, PCl_5 (12.5 g, 60 mmol) was added portion wise and the reaction mixture was stirred overnight. It was quenched by the addition of saturated $NaHCO_3$ solution at -20 ℃. The mixture allowed to warm back to room temperature, water was added to completely neutralize the solution. The aqueous phase was extracted by CH_2Cl_2 (3 x 50 mL), the organic phases were collected, dried over Na_2SO_4, concentrated under reduced pressure. Purification by column chromatography on silica gel (eluent: PE) provided the desired compound (4.05 g) as slightly yellow oil. (85% yield over two steps)

The general procedure C [1,3]

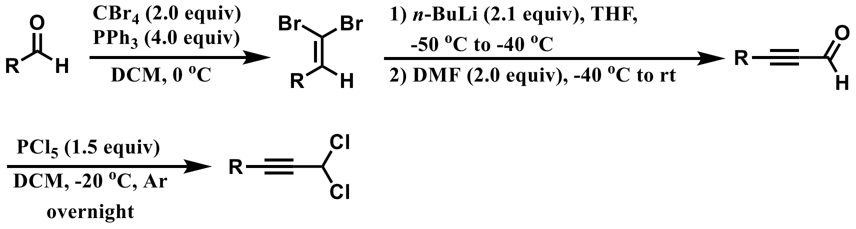

Step 1: Under argon atmosphere, a solution of triphenylphosphine (4.0 equiv) and tetrabromomethane (2.0 equiv) in DCM (0.15 M) was stirred at 0 ℃ for 30 minutes. Then the conresponding aldehyde was added over a period of five minutes, and the mixture was stirred at 0 ℃ for one hour. After addition of water, the layers were separated, and the aqueous layer was extracted with DCM (3 x 50 mL). The combined organic layers were dried over Na_2SO_4 and the solvent was removed under reduced pressure. The crude product was dry-loaded on silica and subjected to flash chromatography (silica, DCM/PE).

Step 2: Under argon atmosphere, *n*-BuLi (2.1 equiv, 2.5 M in hexane) was added over a

period of 30 minutes via syringe pump to a solution of *gem*-dibromoolefine (1.0 equiv) in dry THF (0.4 M) at -50 ℃, and the mixture was stirred at -40 ℃ for 15 minutes. After addition of dry DMF (2.0 equiv) at once, the mixture was allowed to warm to room temperature and stirred for one hour. The mixture was added to a stirring solution of aqueous NaH_2PO_4/Et_2O (1:1). After five minutes, the layers were separated and the aqueous layer was extracted with Et_2O (3 x 50 mL). The combined organic layers were dried over Na_2SO_4, the solvent was removed under reduced pressure and the crude product was subjected to flash chromatography (silica, EA/PE).

Step 3: The crude product was dissolved in dry CH_2Cl_2 (1.0 M) and cooled to -20 ℃. Then, PCl_5 (1.5 equiv) was added portion wise and the reaction mixture was stirred overnight. It was quenched by the addition of saturated $NaHCO_3$ solution at -20 ℃. The mixture allowed to warm back to 25 ℃, water was added to completely neutralize the solution. The aqueous phase was extracted by CH_2Cl_2 (3 x 50 mL); the organic phases were collected, dried over Na_2SO_4, concentrated under reduced pressure. Purification by column chromatography on silica gel (eluent: PE) provided the desired compound.

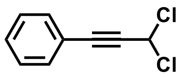

1a

Prepared according to the general procedure **B** using corresponding aldehyde (2.60 g, 20 mmol). The product **1a** was isolated in 84% yield (3.11 g) by column chromatography as yellow oil. **Eluent**: petroleum ether. **^{1}H NMR (400 MHz, $CDCl_3$):** δ 7.50 – 7.48 (m, 2H), 7.42 – 7.33 (m, 3H), 6.49 (s, 1H). **^{13}C NMR (100 MHz, $CDCl_3$):**δ 132.08, 129.94, 128.59, 120.69, 90.03, 84.17, 56.12. **HRMS (EI):** m/z calculated for $C_9H_6Cl_2$ $[M]^+$: 183.9841, found: 183.9840. **IR** (KBr): 2225, 1491, 1444, 1279, 1236, 757, 722, 687.

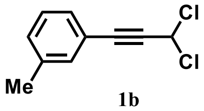

1b

Prepared according to the general procedure **B** using corresponding aldehyde (2.88 g, 20 mmol). The product **1b** was isolated in 67% yield (2.67 g) by column chromatography as yellow oil. **Eluent**: petroleum ether. **^{1}H NMR (400 MHz, $CDCl_3$):** δ 7.32 – 7.28 (m, 2H), 7.26 – 7.24 (m, 1H), 7.22 – 7.19 (m, 1H), 6.49 (s, 1H), 2.34 (s, 3H). **^{13}C NMR (100 MHz, $CDCl_3$):** δ 138.24, 132.47, 130.69, 129.01, 128.34, 120.38, 90.18, 83.72, 56.03, 21.15. **HRMS (EI):** m/z calculated for $C_{10}H_8Cl_2$ $[M]^+$: 197.9998, found: 198.0010. **IR** (KBr): 2233, 1485, 1293, 1243, 1035, 785, 723, 688.

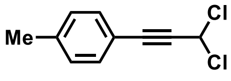

1c

Prepared according to the general procedure **B** using corresponding aldehyde (1.44 g, 10 mmol). The product **1c** was isolated in 71% yield (1.42 g) by column chromatography as yellow solid. **Eluent**: petroleum ether. **^{1}H NMR (400 MHz, $CDCl_3$):** δ 7.38 (d, *J* = 8.1 Hz, 2H), 7.16 (d, *J* = 7.9 Hz, 2H), 6.49 (s, 1H), 2.37 (s, 3H). **^{13}C NMR (100 MHz, $CDCl_3$):** δ 140.21, 131.86,

129.22, 117.50, 90.28, 83.52, 56.14, 21.60. **HRMS (EI):** m/z calculated for $C_{10}H_8Cl_2$ $[M]^+$: 197.9998, found: 197.9997. **IR** (KBr): 2224, 1509, 1282, 1232, 1197, 1023, 1007, 823, 723, 692.

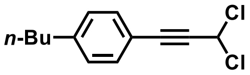

1d

Prepared according to the general procedure **B** using corresponding aldehyde (2.05 g, 11 mmol). The product **1d** was isolated in 87% yield (2.31 g) by column chromatography as yellow oil. **Eluent**: petroleum ether. **^{1}H NMR (400 MHz, CDCl$_3$):** δ 7.41 – 7.39 (m, 2H), 7.17 – 7.15 (m, 2H), 6.49 (s, 1H), 2.62 (t, *J* = 7.6 Hz, 2H), 1.62 – 1.55 (m, 2H), 1.39 – 1.29 (m, 2H), 0.92 (t, *J* = 7.4 Hz, 3H). **^{13}C NMR (100 MHz, CDCl$_3$):** δ 145.19, 131.88, 128.58, 117.69, 90.35, 83.54, 56.15, 35.64, 33.26, 22.26, 13.88. **HRMS (EI):** m/z calculated for $C_{13}H_{14}Cl_2$ $[M]^+$: 240.0467, found: 240.0484. **IR** (KBr): 2225, 1510, 1283, 1237, 1195, 1023, 1005, 835, 723, 696.

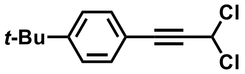

1e

Prepared according to the general procedure **B** using corresponding aldehyde (2.79 g, 15 mmol). The product **1e** was isolated in 72% yield (2.60 g) by column chromatography as yellow solid. **Eluent**: petroleum ether. **^{1}H NMR (400 MHz, CDCl$_3$):** δ 7.43 – 7.41 (m, 2H), 7.38 – 7.36 (m, 2H), 6.49 (s, 1H), 1.31 (s, 9H). **^{13}C NMR (100 MHz, CDCl$_3$):** δ 153.32, 131.73, 125.49, 117.53, 90.28, 83.55, 56.16, 34.94, 31.10. **HRMS (EI):** m/z calculated for $C_{13}H_{14}Cl_2$ $[M]^+$: 240.0467, found: 240.0475. **IR** (KBr): 2226, 1505, 1464, 1271, 1239, 1195, 1021, 1005, 835, 724, 688.

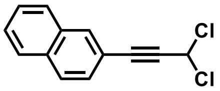

1f

Prepared according to the general procedure **C** using corresponding aldehyde (1.62 g, 9 mmol). The product **1f** was isolated in 45% yield (0.95 g) by column chromatography as yellow solid. **Eluent**: petroleum ether. **^{1}H NMR (400 MHz, CDCl$_3$):** δ 8.04 (s, 1H), 7.85 – 7.81 (m, 3H), 7.56 – 7.49 (m, 3H), 6.55 (s, 1H). **^{13}C NMR (100 MHz, CDCl$_3$):** δ 133.45, 132.68, 132.64, 128.26, 128.01, 127.85, 127.49, 126.88, 117.79, 90.30, 84.21, 56.05. **HRMS (EI):** m/z calculated for $C_{13}H_8Cl_2$ $[M]^+$: 233.9998, found: 234.0007. **IR** (KBr): 2222, 1498, 1280, 1266, 1242, 1196, 1025, 1014, 828, 750, 715, 687.

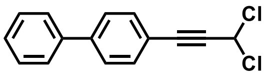

1g

Prepared according to the general procedure **B** using corresponding aldehyde (2.47 g, 12 mmol). The product **1g** was isolated in 52% yield (1.63 g) by column chromatography as yellow solid. **Eluent**: dichloromethane /petroleum ether = 20:80. **^{1}H NMR (400 MHz, CDCl$_3$):** δ 7.61 – 7.55

(m, 6H), 7.48 – 7.44 (m, 2H), 7.40 – 7.36 (m, 1H), 6.52 (s, 1H). **^{13}C NMR (100 MHz, $CDCl_3$):** δ 142.60, 139.96, 132.41, 128.94, 127.99, 127.13, 127.10, 119.37, 89.87, 84.61, 56.03 ppm; **HRMS (EI):** m/z calculated for $C_{15}H_{10}Cl_2$ $[M]^+$: 260.0154, found: 260.0162. **IR** (KBr): 2222, 1497, 1288, 1232, 1197, 1027, 1002, 840, 766, 718, 689.

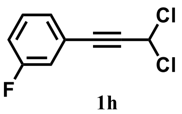

1h

Prepared according to the general procedure **B** using corresponding aldehyde (1.48 g, 10 mmol). The product **1h** was isolated in 43% yield (0.87 g) by column chromatography as yellow oil. **Eluent**: petroleum ether. **^{1}H NMR (400 MHz, $CDCl_3$):** δ 7.36 – 7.26 (m, 2H), 7.21 – 7.18 (m, 1H), 7.14 – 7.09 (m, 1H), 6.47 (s, 1H). **^{19}F NMR (376 MHz, $CDCl_3$):** δ -112.11. **^{13}C NMR (100 MHz, $CDCl_3$):** δ 162.26 (d, J = 246.2 Hz), 130.17 (d, J = 8.5 Hz), 127.88 (d, J = 3.2 Hz), 122.4 (d, J = 9.4 Hz), 118.80 (d, J = 23.2 Hz), 117.26 (d, J = 21.0 Hz), 88.30 (d, J = 3.4 Hz), 84.71, 55.60. **HRMS (EI):** m/z calculated for $C_9H_5Cl_2F[M]^+$: 201.9747, found: 201.9749. **IR** (KBr): 2237, 1609, 1583, 1487, 1296, 1244, 1176, 1154, 1024, 875, 786, 723, 679.

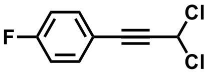

1i

Prepared according to the general procedure **B** using corresponding aldehyde (1.4 g, 9.4 mmol). The product **1i** was isolated in 78% yield (1.50 g) by column chromatography as yellow oil. **Eluent**: petroleum ether. **^{1}H NMR (400 MHz, $CDCl_3$):** δ 7.50 – 7.45 (m, 2H), 7.08 – 7.02 (m, 2H), 6.47 (s, 1H). **^{19}F NMR (376 MHz, $CDCl_3$):** δ -112.77. **^{13}C NMR (100 MHz, $CDCl_3$):** δ 163.40 (d, J = 250.5 Hz), 134.07 (d, J = 8.6 Hz), 116.70 (d, J = 3.6 Hz), 115.91 (d, J = 22.17 Hz), 88.83, 83.85, 55.85. **HRMS (EI):** m/z calculated for $C_9H_5Cl_2F[M]^+$: 201.9747, found: 201.9757. **IR** (KBr): 2229, 1601, 1479, 1283, 1237, 1157, 1014, 836, 726, 696.

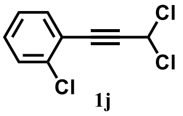

1j

Prepared according to the general procedure **B** using corresponding aldehyde (0.5 g, 3.0 mmol). The product **1j** was isolated in 50% yield (0.33 g) by column chromatography as yellow oil. **Eluent**: petroleum ether. **^{1}H NMR (400 MHz, $CDCl_3$):** δ 7.52 (dd, J_1 = 7.6 Hz, J_2 = 1.7 Hz, 1H), 7.44 – 7.41 (m, 1H), 7.33 (td, J_1 = 7.6 Hz, J_2 = 1.7 Hz, 1H), 7.25 (td, J_1 = 7.2 Hz, J_2 = 1.3 Hz, 1H), 6.53 (s, 1H). **^{13}C NMR (100 MHz, $CDCl_3$):** δ 136.52, 133.72, 130.87, 129.49, 126.58, 120.72, 88.57, 86.45, 55.69. **HRMS (EI):** m/z calculated for $C_9H_5Cl_3[M]^+$: 217.9451, found: 217.9462. **IR** (KBr): 2230, 1474, 1435, 1293, 1250, 1068, 1035, 1003, 756, 727, 690.

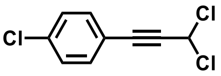

1k

Prepared according to the general procedure **B** using corresponding aldehyde (3.46 g, 21.0 mmol). The product **1k** was isolated in 88% yield (4.06 g) by column chromatography as yellow solid.

Eluent: petroleum ether. **^{1}H NMR (400 MHz, CDCl$_3$):** δ 7.44 – 7.41 (m, 2H), 7.36 – 7.32 (m, 2H), 6.47 (s, 1H). **^{13}C NMR (100 MHz, CDCl$_3$):** δ 136.11, 133.18, 128.90, 119.05, 88.62, 84.87, 55.73. **HRMS (EI):** m/z calculated for $C_9H_5Cl_3$[M]$^+$: 217.9451, found: 217.9461. **IR** (KBr): 2225, 1491, 1279, 1232, 1090, 1004, 830, 761, 717, 687.

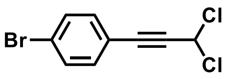

1l

Prepared according to the general procedure **B** using corresponding aldehyde (2.47 g, 11.8 mmol). The product **1l** was isolated in 80% yield (2.50 g) by column chromatography as white solid. **Eluent**: petroleum ether. **^{1}H NMR (400 MHz, CDCl$_3$):** δ 7.52 – 7.49 (m, 2H), 7.37 – 7.34 (m, 2H), 6.47 (s, 1H). **^{13}C NMR (100 MHz, CDCl$_3$):** δ 133.32, 131.83, 124.41, 119.52, 88.67, 85.02, 55.71. **HRMS (EI):** m/z calculated for $C_9H_5Cl_2Br$[M]$^+$: 261.8946, found: 261.8950. **IR** (KBr): 2224, 1582, 1487, 1278, 1239, 1207, 1069, 1011, 826, 749, 718, 686.

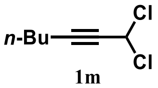

1m

Prepared according to the general procedure **A** using corresponding aldehyde (2.97 g, 27.0 mmol). The product **1m** was isolated in 66% yield (2.94 g) by column chromatography as colorless oil. **Eluent**: petroleum ether (b.p. = 30 – 60 °C). **^{1}H NMR (400 MHz, CDCl$_3$):** δ 6.26 (t, J = 2.0 Hz, 1H), 2.32 (td, J_1 = 7.0 Hz, J_2 = 2.0 Hz, 2H), 1.57 – 1.50 (m, 2H), 1.46 – 1.37 (m, 2H), 0.92 (t, J = 7.3 Hz, 3H). **^{13}C NMR (100 MHz, CDCl$_3$):** δ 92.42, 76.32, 56.05, 29.87, 21.90, 18.55, 13.51. **HRMS (EI):** m/z calculated for $C_7H_{10}Cl_2$ [M]$^+$: 164.0154, found: 164.0151. **IR** (KBr): 2959, 2928, 2858, 2239, 1465, 1379, 1254, 1197, 1159, 815, 725, 685.

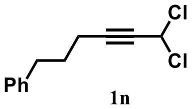

1n

Prepared according to the general procedure **B** using corresponding aldehyde (1.1 g, 6.4 mmol). The product **1n** was isolated in 45% yield (0.66 g) by column chromatography as colorless oil. **Eluent**: petroleum ether (b.p. = 30 – 60 °C). **^{1}H NMR (400 MHz, CDCl$_3$):** δ 7.31 – 7.27 (m, 2H), 7.22 – 7.18 (m, 3H), 6.27 (t, J = 2.0 Hz, 1H), 2.72 (t, J = 7.4 Hz, 2H), 2.32 (td, J_1 = 7.1 Hz, J_2 = 2.0 Hz, 2H), 1.88 (q, J = 7.0 Hz, 2H). **^{13}C NMR (100 MHz, CDCl$_3$):** δ 141.03, 128.53, 128.46, 126.10, 91.90, 76.92, 55.98, 34.64, 29.38, 18.20. **HRMS (EI):** m/z calculated for $C_{12}H_{12}Cl_2$ [M]$^+$: 226.0311, found: 226.0321. **IR** (KBr): 3027, 2945, 2861, 2238, 1496, 1455, 1426, 1255, 1198, 1158, 862, 722, 699.

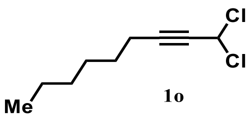

1o

Prepared according to the general procedure **A** using corresponding aldehyde (1.34 g, 9.7 mmol). The product **1o** was isolated in 68% yield (1.28 g) by column chromatography as colorless oil. **Eluent**: petroleum ether (b.p. = 30 – 60 °C). **^{1}H NMR (400 MHz, CDCl$_3$):** δ 6.27 (t, J = 4.0 Hz, 1H), 2.31 (td, J_1 = 7.1 Hz, J_2 = 2.0 Hz, 2H), 1.58 – 1.51 (m, 2H), 1.42 – 1.34 (m, 2H),

1.33 – 1.26 (m, 4H), 0.90 (t, *J* = 6.7 Hz, 3H). **^{13}C NMR (100 MHz, CDCl$_3$):** δ 92.51, 76.34, 56.05, 31.20, 28.45, 27.79, 22.47, 18.85, 13.99. **HRMS (EI):** m/z calculated for $C_7H_9Cl_2$ [M-C_2H_5]$^+$: 163.0076, found: 163.0087. **IR** (KBr): 2956, 2930, 2860, 2322, 2238, 1752, 1690, 1466, 1458, 1428, 1380, 1328, 1252, 1198, 1158, 1030, 958, 814, 780, 718, 684.

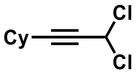

1p

Prepared according to the general procedure **B** using corresponding aldehyde (3.4 g, 25.0 mmol). The product **1p** was isolated in 85% yield (4.06 g) by column chromatography as pale yellow oil. **Eluent**: petroleum ether. **^{1}H NMR (400 MHz, CDCl$_3$):** δ 6.28 (d, *J* = 1.8 Hz, 1H), 2.54 – 2.49 (m, 1H), 1.83 – 1.78 (m, 2H), 1.73 – 1.66 (m, 2H), 1.52 – 1.44 (m, 3H), 1.36 – 1.30 (m, 3H). **^{13}C NMR (100 MHz, CDCl$_3$):** δ 96.00, 76.53, 56.13, 31.73, 29.03, 25.68, 24.60. **HRMS (EI):** m/z calculated for $C_9H_{12}Cl_2$ [M]$^+$: 190.0311, found: 190.0306. **IR** (KBr): 2934, 2856, 2234, 1449, 1252, 1197, 1165, 888, 864, 722, 688.

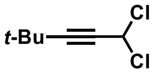

1q

Prepared according to the general procedure **B** using corresponding aldehyde (2.3 g, 20.9 mmol). The product **1q** was isolated in 34% yield (1.18 g) by column chromatography as colorless oil. **Eluent**: petroleum ether (b.p. = 30 – 60 ^{o}C). **^{1}H NMR (400 MHz, CDCl$_3$):** δ 6.26 (s, 1H), 1.25 (s, 9H). **^{13}C NMR (100 MHz, CDCl$_3$):** δ 99.66, 75.18, 56.10, 30.20, 27.65. **HRMS (EI):** m/z calculated for $C_6H_7Cl_2$ [M-CH_3]$^+$: 148.9919, found: 148.9918. **IR** (KBr): 2973, 2931, 1701, 1368, 1270, 1210, 841, 819, 728, 698.

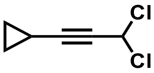

1r

Prepared according to the general procedure **A** using corresponding aldehyde (0.33 g, 3.5 mmol). The product **1r** was isolated in 55% yield (0.29 g) by column chromatography as colorless oil. **Eluent**: petroleum ether (b.p. = 30 – 60 ^{o}C). **^{1}H NMR (400 MHz, CDCl$_3$):** δ 6.24 (d, *J* = 1.7 Hz, 1H), 1.40 – 1.33 (m, 1H), 0.91 – 0.85 (m, 2H), 0.83 – 0.78 (m, 2H). **^{13}C NMR (100 MHz, CDCl$_3$):** δ 95.76, 71.66, 56.58, 9.13, 0.00. **HRMS (EI):** m/z calculated for $C_6H_6Cl_2$ [M]$^+$: 147.9841, found: 147.9843. **IR** (KBr): 2238, 1428, 1361, 1257, 1196, 1165, 1056, 1032, 889, 814, 794, 720, 678.

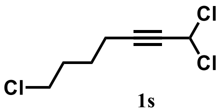

1s

Prepared according to the general procedure **B** using corresponding aldehyde (2.23 g, 15.4 mmol). The product **1s** was isolated in 52% yield (1.60 g) by column chromatography as colorless oil. **Eluent**: petroleum ether. **^{1}H NMR (400 MHz, CDCl$_3$):** δ 6.26 (t, *J* = 2.0 Hz, 1H), 3.58 (t, *J* = 6.4 Hz, 2H), 2.39 (td, J_1 = 7.0 Hz, J_2 = 1.9 Hz, 2H), 1.93 – 1.86 (m, 2H), 1.76 – 1.69 (m, 2H).

^{13}C NMR (100 MHz, CDCl$_3$): δ 91.26, 76.94, 55.83, 44.29, 31.39, 25.03, 18.19. **HRMS (EI):** m/z calculated for $C_7H_9Cl_2$ [M-Cl]$^+$: 163.0076, found: 163.0076. **IR** (KBr): 2957, 2869, 2323, 2239, 1455, 1431, 1255, 1198, 1160, 976, 812, 723, 684.

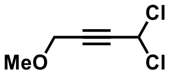

1t

Prepared according to the general procedure **A** using corresponding aldehyde (3.2 g, 32.6 mmol). The product **1t** was isolated in 37% yield (1.87 g) by column chromatography as pale yellow oil. **Eluent**: petroleum ether (b.p. = 30 – 60 °C) /diethyl ether = 90:10. **^{1}H NMR (400 MHz, CDCl$_3$):** δ 6.30 (t, *J* = 1.7 Hz, 1H), 4.22 (d, *J* = 1.7 Hz, 2H), 3.41 (s, 3H). **^{13}C NMR (100 MHz, CDCl$_3$):** δ 86.29, 81.61, 59.56, 58.00, 55.05. **HRMS (EI):** m/z calculated for $C_5H_5Cl_2O$ [M-H]$^+$: 150.9712, found: 150.9713. **IR** (KBr): 2238, 1718, 1340, 1308, 1254, 1198, 1148, 1094, 993, 810, 737, 697.

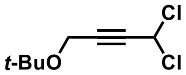

1u

Prepared according to the general procedure **A** using corresponding aldehyde (1.95 g, 13.9 mmol). The product **1u** was isolated in 84% yield (2.27 g) by column chromatography as pale yellow oil. **Eluent**: ethyl acetate /petroleum ether = 2:98. **^{1}H NMR (400 MHz, CDCl$_3$):** δ 6.29 (t, *J* = 1.7 Hz, 1H), 4.20 (d, *J* = 1.6 Hz, 2H), 1.25 (s, 9H). **^{13}C NMR (100 MHz, CDCl$_3$):** δ 88.56, 79.90, 74.96, 55.39, 50.39, 27.45. **HRMS (EI):** m/z calculated for $C_7H_9Cl_2O$[M-CH$_3$]$^+$: 179.0025, found: 179.0026. **IR** (KBr): 2978, 2230, 1602, 1472, 1393, 1368, 1253, 1191, 1148, 1067, 887, 843, 730.

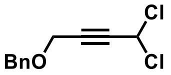

1v

Prepared according to the general procedure **B** using corresponding aldehyde (2.24 g, 12.9 mmol). The product **1v** was isolated in 56% yield (1.64 g) by column chromatography as yellow oil. **Eluent**: diethyl ether /petroleum ether = 2:98. **^{1}H NMR (400 MHz, CDCl$_3$):** δ 7.39 – 7.30 (m, 5H), 6.30 (t, *J* = 1.7 Hz, 1H), 4.61 (s, 2H), 4.28 (d, *J* = 1.7 Hz, 2H). **^{13}C NMR (100 MHz, CDCl$_3$):** δ 136.87, 128.54, 128.18, 128.11, 86.46, 81.69, 72.04, 56.96, 55.09. **HRMS (EI):** m/z calculated for $C_{11}H_9Cl_2O$[M-H]$^+$: 227.0025, found: 227.0029. **IR** (KBr): 3032, 2982, 2859, 2229, 1496, 1455, 1354, 1253, 1199, 1161, 1144, 1074, 1028, 940, 807, 730, 698.

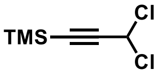

1w

Prepared according to the general procedure **A** using corresponding aldehyde (1.4 g, 11.1 mmol). The product **1w** was isolated in 50% yield (1.00 g) by column chromatography as colorless oil. **Eluent**: ethyl acetate /petroleum ether (b.p. = 30 – 60 °C) = 1:99. **^{1}H NMR (400 MHz, CDCl$_3$):** δ 6.22 (s, 1H), 0.22 (s, 9H). **^{13}C NMR (100 MHz, CDCl$_3$):** δ 99.44, 96.75, 55.69, 0.00. **HRMS (EI):** m/z calculated for $C_5H_7Cl_2Si$ [M-CH$_3$]$^+$: 164.9689, found: 164.9691. **IR** (KBr):

2925, 2854, 2228, 1911, 1458, 1376, 1252, 1114, 846, 731, 699.

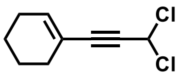

1x

Prepared according to the general procedure **B** using corresponding aldehyde (1.61 g, 12.0 mmol). The product **1x** was isolated in 84% yield (1.91 g) by column chromatography as yellow oil. **Eluent**: petroleum ether (b.p. = 30 – 60 °C). **^{1}H NMR (400 MHz, CDCl$_3$):** δ 6.39 (s, 1H), 6.27 (p, J = 1.9 Hz, 1H), 2.15 – 2.11 (m, 4H), 1.68 – 1.56 (m, 4H). **^{13}C NMR (100 MHz, CDCl$_3$):** δ 138.81, 118.91, 92.01, 81.79, 56.35, 28.33, 25.74, 21.99, 21.22. **HRMS (EI):** m/z calculated for $C_9H_{10}Cl_2[M]^+$: 188.0154, found: 188.0150. **IR** (KBr): 2936, 2861, 2222, 1628, 1449, 1435, 1274, 1224, 1195, 1137, 1078, 1049, 1005, 931, 919, 848, 799, 729, 685.

3 Optimization of the Reaction Conditions of Racemic Silyl-substituted Allenes.

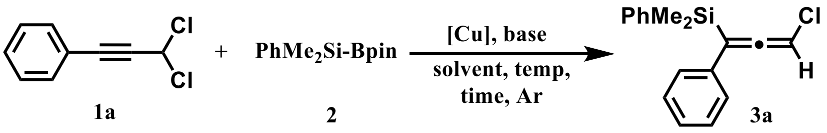

entry	cat. (mol%)	base (equiv)	solvent (mL)	temp (°C)	time (h)	yield (%)
1	CuTc (10)	NaOMe (4.0)	DCE	-30	36	0
2	CuTc (10)	NaOMe (2.0)	DCE	-30	36	54
3	CuTc (10)	NaOMe (2.0)	DCE	-10	36	60
4	CuTc (10)	NaOMe (2.0)	DCE	rt	36	6
5	CuTc (10)	NaOMe (2.0)	MeOH	-10	36	62
6	CuTc (10)	Et_3N (2.0)	MeOH	-10	45	72
7	CuTc (10)	Et_3N (2.0)	DCE/MeOH= 2:1	-10	45	76
8	CuTc (10)	Et_3N (2.0)	*i*-PrOH	-10	24	23
9	CuTc (10)	Et_3N (2.0)	*t*-AmOH	-10	24	10
10	CuCl (10)	Et_3N (2.0)	DCE/MeOH= 2:1	-10	20	76
11	CuBr (10)	Et_3N (2.0)	DCE/MeOH= 2:1	-10	20	76
12	CuCN (10)	Et_3N (2.0)	DCE/MeOH= 2:1	-10	20	63
13	CuI (5)	Et_3N (2.0)	DCE/MeOH= 2:1	-10	2	79[c]
14	--	Et_3N (2.0)	DCE/MeOH= 2:1	-10	2	--
15	CuI (5)	--	DCE/MeOH= 2:1	-10	2	--

[a]Unless noted otherwise, the reaction was conducted with **1a** (0.2 mmol), **2** (0.4 mmol, 2.0 equiv), copper catalyst (0.02 mmol,10 mol %), and base (0.4 mmol, 2.0 equiv) in indicated dry solvent (1.5 mL) for corresponding time under argon atmosphere. [b]NMR yield with 1,3,5 - trimethylbenzene as internal standard. [c]Isolated yield.

4. General Procedure for the Synthesis of Racemic Silyl-substituted Allenes. (GP2)

4.1 Procedures for Synthesis of Products 3a-3x.

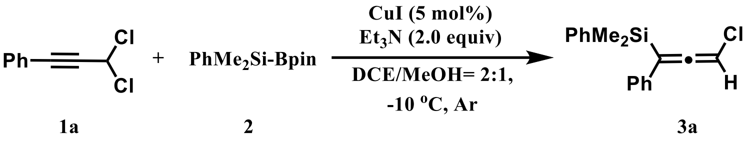

3a: A dried screw-vial seal tube was charged with CuI (1.9 mg, 0.01 mmol, 5 mol %) in glove box, then removed it out of the glove box. The tube was equipped with an argon balloon, dry Et_3N (40.5 mg, 0.4 mmol, 2.0 equiv), dry DCE (1.0 mL) and dry MeOH (0.5 mL) were added into it. The mixture was stirred for 1 hour at room temperature to form a white suspension. Then the tube was cooled to -10 °C, **1a** (37 mg, 0.2 mmol, 1.0 equiv) and $PhMe_2Si$-Bpin (105 mg, 0.4 mmol, 2.0 equiv) were added in sequence *via* syringe. It was continued to stir for 2 hours at -10 °C. The tube was opened quickly at room temperature, the final solution was filtered through celite using CH_2Cl_2 (30 mL) under air. The filtrate was concentrated under reduced pressure to afford the crude product, which was purified by flash column chromatography on silica gel (eluent: PE) to obtain the desired product **3a** (45.0 mg, 79%) as yellow oil. **^{1}H NMR (400 MHz, $CDCl_3$):** δ 7.60 – 7.57 (m, 2H), 7.41 – 7.34 (m, 3H), 7.25 – 7.17 (m, 5H), 6.20 (s, 1H), 0.52 (s, 3H), 0.51 (s, 3H). **^{13}C NMR (100 MHz, $CDCl_3$):** δ 209.13, 139.01, 137.11, 136.00, 131.65, 130.65, 130.46, 130.14, 129.67, 113.03, 89.62, 0.06, 0.00. **HRMS (EI):** m/z calculated for $C_{17}H_{17}ClSi[M]^+$: 284.0783, found: 284.0798. **IR** (KBr): 3053, 3021, 2959, 1921, 1490, 1428, 1304, 1251, 1211, 1113, 917, 835, 814, 781, 743, 696, 646.

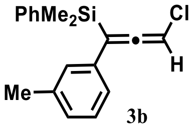

3b: A dried screw-vial seal tube was charged with CuI (1.9 mg, 0.01 mmol, 5 mol %) in glove box, then removed it out of the glove box. The tube was equipped with an argon balloon, dry Et_3N (40.5 mg, 0.4 mmol, 2.0 equiv), dry DCE (1.0 mL) and dry MeOH (0.5 mL) were added into it. The mixture was stirred for 1 hour at room temperature to form a white suspension. Then the tube was cooled to -10 °C, **1b** (39.8 mg, 0.2 mmol, 1.0 equiv) and $PhMe_2Si$-Bpin (105 mg, 0.4 mmol, 2.0 equiv) were added in sequence *via* syringe. It was continued to stir for 4 hours at -10 °C. The tube was opened quickly at room temperature, the final solution was filtered through celite using CH_2Cl_2 (30 mL) under air. The filtrate was concentrated under reduced pressure to afford the crude product, which was purified by flash column chromatography on silica gel (eluent: PE) to obtain the desired product **3b** (36.9 mg, 62%) as yellow oil. **^{1}H NMR (400 MHz, $CDCl_3$):** δ 7.60 – 7.57 (m, 2H), 7.39 – 7.35 (m, 3H), 7.13 – 7.07 (m, 2H), 7.02 – 6.99 (m, 2H), 6.18 (s, 1H), 2.26 (s, 3H), 0.52(s, 3H), 0.51(s, 3H). **^{13}C NMR (100 MHz, $CDCl_3$):** δ 208.98, 140.27, 139.09, 137.01, 136.00, 131.59, 131.14, 130.47, 130.45, 130.09, 127.51, 113.12, 89.46, 23.43, 0.07, 0.00. **HRMS (EI):** m/z calculated for $C_{18}H_{19}ClSi[M]^+$: 298.0939, found: 298.0941. **IR** (KBr): 3050, 2959, 2924, 2868, 1924, 1602, 1487, 1458, 1428, 1296, 1248, 1112, 949, 822, 781, 730, 699, 648.

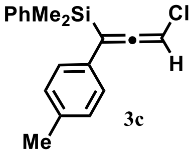

3c

3c: A dried screw-vial seal tube was charged with CuI (1.9 mg, 0.01 mmol, 5 mol %) in glove box, then moved it out of the glove box. The tube was equipped with an argon balloon, dry Et_3N (40.5 mg, 0.4 mmol, 2.0 equiv), dry DCE (1.0 mL) and dry MeOH (0.5 mL) were added into it. The mixture was stirred for 1 hour at room temperature to form a white suspension. Then the tube was cooled to -10 ^{o}C, **1c** (39.8 mg, 0.2 mmol, 1.0 equiv) and $PhMe_2Si$-Bpin (105 mg, 0.4 mmol, 2.0 equiv) were added in sequence *via* syringe. It was continued to stir for 3 hours at -10 ^{o}C. The tube was opened quickly at room temperature, the final solution was filtered through celite using CH_2Cl_2 (30 mL) under air. The filtrate was concentrated under reduced pressure to afford the crude product, which was purified by flash column chromatography on silica gel (eluent: PE) to obtain the desired product **3c** (42.9 mg, 72%) as yellow oil. **^{1}H NMR (400 MHz, $CDCl_3$):** δ 7.60 - 7.58 (m, 2H), 7.39 - 7.34 (m, 3H), 7.14 (d, *J* = 6.7 Hz, 2H), 7.07 (d, *J* = 7.7 Hz, 2H), 6.19 (s, 1H), 2.29 (s, 3H), 0.51 (s, 6H). **^{13}C NMR (100 MHz, $CDCl_3$):** δ 208.68, 139.50, 139.03, 135.87, 133.84, 131.46, 131.25, 130.27, 129.99, 112.65, 89.42, 23.09, 0.00, -0.07. **HRMS (EI):** m/z calculated for $C_{18}H_{19}ClSi[M]^+$: 298.0939, found: 298.0951. **IR** (KBr): 3050, 3022, 2958, 2920, 1919, 1508, 1428, 1317, 1303, 1251, 1207, 1113, 908, 819, 780, 733, 701, 656.

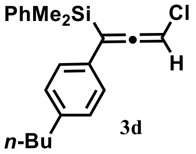

3d

3d: A dried screw-vial seal tube was charged with CuI (1.9 mg, 0.01 mmol, 5 mol %) in glove box, then moved it out of the glove box. The tube was equipped with an argon balloon, dry Et_3N (40.5 mg, 0.4 mmol, 2.0 equiv), dry DCE (1.0 mL) and dry MeOH (0.5 mL) were added into it. The mixture was stirred for 1 hour at room temperature to form a white suspension. Then the tube was cooled to -10 ^{o}C, **1d** (48.2 mg, 0.2 mmol, 1.0 equiv) and $PhMe_2Si$-Bpin (105 mg, 0.4 mmol, 2.0 equiv) were added in sequence *via* syringe. It was continued to stir for 4 hours at -10 ^{o}C. The tube was opened quickly at room temperature, the final solution was filtered through celite using CH_2Cl_2 (30 mL) under air. The filtrate was concentrated under reduced pressure to afford the crude product, which was purified by flash column chromatography on silica gel (eluent: PE) to obtain the desired product **3d** (51.4 mg, 75%) as yellow oil. **^{1}H NMR (400 MHz, $CDCl_3$):** δ 7.60 - 7.58 (m, 2H), 7.39 - 7.35 (m, 3H), 7.17 - 7.14 (m, 2H), 7.06 - 7.04 (m, 2H), 6.19 (s, 1H), 2.54 (t, *J* = 8.0 Hz, 2H), 1.58 - 1.52 (m, 3H), 1.36 - 1.27 (m, 2H), 0.89 (t, *J* = 7.9 Hz, 3H), 0.52 (s, 3H), 0.51 (s, 3H). **^{13}C NMR (100 MHz, $CDCl_3$):** δ 208.71, 144.53, 139.07, 135.87, 134.00, 131.43, 130.59, 130.25, 129.97, 112.71, 89.39, 37.23, 35.38, 24.26, 15.85, 0.00, -0.07. **HRMS (EI):** m/z calculated for $C_{21}H_{25}ClSi[M]^+$: 340.1409, found: 340.1408. **IR** (KBr): 3050, 2957, 2929, 2858, 1919, 1507, 1457, 1428, 1310, 1251, 1213, 1113, 909, 835, 780, 734, 700, 658.

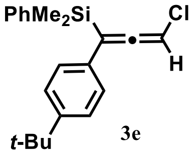

3e

3e: A dried screw-vial seal tube was charged with CuI (1.9 mg, 0.01 mmol, 5 mol %) in glove box, then moved it out of the glove box. The tube was equipped with an argon balloon, dry Et_3N (40.5 mg, 0.4 mmol, 2.0 equiv), dry DCE (1.0 mL) and dry MeOH (0.5 mL) were added into it. The mixture was stirred for 1 hour at room temperature to form a white suspension. Then the tube was cooled to -10 ^oC, **1e** (48.2 mg, 0.2 mmol, 1.0 equiv) and $PhMe_2Si$-Bpin (105 mg, 0.4 mmol, 2.0 equiv) were added in sequence *via* syringe. It was continued to stir for 3 hours at -10 ^oC. The tube was opened quickly at room temperature, the final solution was filtered through celite using CH_2Cl_2 (30 mL) under air. The filtrate was concentrated under reduced pressure to afford the crude product, which was purified by flash column chromatography on silica gel (eluent: PE) to obtain the desired product **3e** (62.8 mg, 87%) as yellow oil. **^{1}H NMR (400 MHz, $CDCl_3$):** δ 7.61 - 7.59 (m, 2H), 7.39 - 7.34 (m, 3H), 7.27 - 7.25 (m, 2H), 7.20 - 7.18 (m, 2H), 6.19 (s, 1H), 1.27(s, 9H), 0.52 (s, 3H), 0.51(s, 3H). **^{13}C NMR (100 MHz, $CDCl_3$):** δ 208.83, 152.68, 139.08, 135.84, 133.70, 131.41, 130.04, 129.96, 127.46, 112.47, 89.41, 36.45, 33.13, 0.00, -0.10. **HRMS (EI):** m/z calculated for $C_{21}H_{25}ClSi[M]^+$: 340.1409, found: 340.1412. **IR** (KBr): 3051, 2962, 2920, 2851, 1918, 1508, 1428, 1364, 1252, 1112, 913, 835, 813, 780, 732, 701, 654.

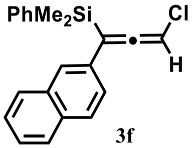

3f

3f: A dried screw-vial seal tube was charged with CuI (3.8 mg, 0.01 mmol, 10 mol %) in glove box, then moved it out of the glove box. The tube was equipped with an argon balloon, dry Et_3N (40.5 mg, 0.4 mmol, 2.0 equiv), dry DCE (1.0 mL) and dry MeOH (0.5 mL) were added into it. The mixture was stirred for 1 hour at room temperature to form a white suspension. Then the tube was cooled to -10 ^oC, **1f** (47 mg, 0.2 mmol, 1.0 equiv) and $PhMe_2Si$-Bpin (105 mg, 0.4 mmol, 2.0 equiv) were added in sequence *via* syringe. It was continued to stir for 4 hours at -10 ^oC. The tube was opened quickly at room temperature, the final solution was filtered through celite using CH_2Cl_2 (30 mL) under air. The filtrate was concentrated under reduced pressure to afford the crude product, which was purified by flash column chromatography on silica gel (eluent: PE) to obtain the desired product **3f** (71.7 mg, 52%) as yellow oil. **^{1}H NMR (400 MHz, $CDCl_3$):** δ 7.70 - 7.77 (m, 2H), 7.59 - 7.66 (m, 4H), 7.35 - 7.45 (m, 6H), 6.27 (s, 1H), 0.58(s, 3H), 0.57(s, 3H). **^{13}C NMR (100 MHz, $CDCl_3$):** δ 209.48, 138.94, 135.95, 135.28, 134.65, 134.39, 131.61, 130.19, 130.10, 130.06, 129.54, 129.50, 128.39, 128.16, 113.12, 89.82, 0.07, 0.00. **HRMS (EI):** m/z calculated for $C_{21}H_{19}ClSi[M]^+$: 334.0939, found: 334.0946. **IR** (KBr): 3052, 2958, 2920, 2850, 1916, 1596, 1504, 1427, 1406, 1288, 1251, 1185, 1113, 961, 914, 820, 735, 701, 656.

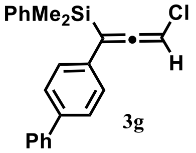

3g

3g: A dried screw-vial seal tube was charged with CuI (1.9 mg, 0.01 mmol, 5 mol %) in glove box, then moved it out of the glove box. The tube was equipped with an argon balloon, dry Et_3N (40.5 mg, 0.4 mmol, 2.0 equiv), dry DCE (1.0 mL) and dry MeOH (0.5 mL) were added into it. The mixture was stirred for 1 hour at room temperature to form a white suspension. Then the tube was cooled to -10 ^oC, **1g** (52.2 mg, 0.2 mmol, 1.0 equiv) and $PhMe_2Si$-Bpin (105 mg, 0.4 mmol, 2.0 equiv) were added in sequence *via* syringe. It was continued to stir for 3.5 hours at -10 ^oC. The tube was opened quickly at room temperature, the final solution was filtered through celite using CH_2Cl_2 (30 mL) under air. The filtrate was concentrated under reduced pressure to afford the crude product, which was purified by flash column chromatography on silica gel (eluent: PE) to obtain the desired product **3g** (45.5 mg, 63%) as yellow oil. **^{1}H NMR (400 MHz, $CDCl_3$):** δ 7.63 – 7.60 (m, 2H), 7.55 – 7.52 (m, 2H), 7.48 – 7.46 (m, 2H), 7.40 – 7.38 (m, 6H), 7.33 – 7.30 (m, 2H), 6.24 (s, 1H), 0.55 (s, 3H), 0.54 (s, 3H). **^{13}C NMR (100 MHz, $CDCl_3$):** δ 209.14, 142.40, 142.36, 138.86, 135.88, 135.85, 131.56, 130.77, 130.72, 130.05, 129.35, 129.20, 128.88, 112.51, 89.65, 0.00, -0.09. **HRMS (EI):** m/z calculated for $C_{23}H_{21}ClSi[M]^+$: 360.1096, found: 360.1108. **IR** (KBr): 3051, 3028, 2958, 1918, 1600, 1485, 1447, 1427, 1404, 1317, 1251, 1213, 1136, 1111, 1007, 909, 837, 780, 734, 697, 659.

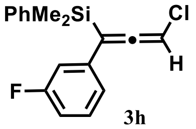

3h

3h: A dried screw-vial seal tube was charged with CuI (1.9 mg, 0.01 mmol, 5 mol %) in glove box, then moved it out of the glove box. The tube was equipped with an argon balloon, dry Et_3N (40.5 mg, 0.4 mmol, 2.0 equiv), dry DCE (1.0 mL) and dry MeOH (0.5 mL) were added into it. The mixture was stirred for 1 hour at room temperature to form a white suspension. Then the tube was cooled to -10 ^oC, **1h** (40.6 mg, 0.2 mmol, 1.0 equiv) and $PhMe_2Si$-Bpin (105 mg, 0.4 mmol, 2.0 equiv) were added in sequence *via* syringe. It was continued to stir for 3 hours at -10 ^oC. The tube was opened quickly at room temperature, the final solution was filtered through celite using CH_2Cl_2 (30 mL) under air. The filtrate was concentrated under reduced pressure to afford the crude product, which was purified by flash column chromatography on silica gel (eluent: PE) to obtain the desired product **3h** (41.9 mg, 69%) as yellow oil. **^{1}H NMR (400 MHz, $CDCl_3$):** δ 7.58 – 7.56 (m, 2H), 7.41 – 7.36 (m, 3H), 7.21 – 7.16 (m, 1H), 6.99 – 6.87 (m, 3H), 6.22 (s, 1H), 0.52 (s, 3H), 0.51(s, 3H). **^{19}F NMR (376 MHz, $CDCl_3$):** δ -112.77. **^{13}C NMR (100 MHz, $CDCl_3$):** δ 207.41, 162.78 (d, J = 244.8 Hz), 137.45 (d, J = 7.5 Hz), 136.44, 133.87, 129.89 (d, J = 8.3 Hz), 129.72, 128.14, 124.04 (d, J = 2.8 Hz), 115.2, 115.0, 114.59, 114.38, 110.23, 87.80, -2.13, -2.22. **HRMS (EI):** m/z calculated for $C_{17}H_{16}SiClF[M]^+$: 302.0688, found: 302.0698. **IR** (KBr): 3053, 2960, 1927, 1608, 1583, 1483, 1428, 1315, 1241, 1113, 984, 874, 830, 785, 733, 701, 647.

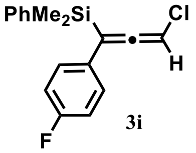

3i

3i: A dried screw-vial seal tube was charged with CuI (1.9 mg, 0.01 mmol, 5 mol %) in glove box, then moved it out of the glove box. The tube was equipped with an argon balloon, dry Et_3N (40.5 mg, 0.4 mmol, 2.0 equiv), dry DCE (1.0 mL) and dry MeOH (0.5 mL) were added into it. The mixture was stirred for 1 hour at room temperature to form a white suspension. Then the tube was cooled to -10 ^oC, **1i** (40.6 mg, 0.2 mmol, 1.0 equiv) and $PhMe_2Si$-Bpin (105 mg, 0.4 mmol, 2.0 equiv) were added in sequence *via* syringe. It was continued to stir for 4 hours at -10 ^oC. The tube was opened quickly at room temperature, the final solution was filtered through celite using CH_2Cl_2 (30 mL) under air. The filtrate was concentrated under reduced pressure to afford the crude product, which was purified by flash column chromatography on silica gel (eluent: PE) to obtain the desired product **3i** (46.6 mg, 77%) as yellow oil. **^{1}H NMR (400 MHz, $CDCl_3$):** δ 7.58 – 7.56 (m, 2H), 7.40 – 7.35 (m, 3H), 7.20 – 7.17 (m, 2H), 6.94 – 6.90 (m, 2H), 6.22 (s, 1H), 0.51 (s, 3H), 0.50 (s, 3H). **^{19}F NMR (376 MHz, $CDCl_3$):** δ -114.16. **^{13}C NMR (100 MHz, $CDCl_3$):** δ 209.08 (d, *J* = 1.8 Hz), 164.4 (d, *J* = 246.2 Hz), 138.80, 136.05, 133.10 (d, *J* = 3.4 Hz), 132.15 (d, *J* = 8.1 Hz), 131.86, 130.31, 117.72 (d, *J* = 21.5 Hz), 112.17, 89.89, 0.07, 0.00. **HRMS (EI):** m/z calculated for $C_{17}H_{16}SiClF[M]^+$: 302.0688, found: 302.0703. **IR** (KBr): 3069, 2959, 2922, 1922, 1598, 1505, 1428, 1312, 1252, 1232, 1159, 1113, 908, 837, 781, 734, 701, 657.

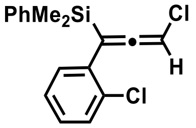

3j

3j: A dried screw-vial seal tube was charged with CuI (1.9 mg, 0.01 mmol, 5 mol %) in glove box, then moved it out of the glove box. The tube was equipped with an argon balloon, dry Et_3N (40.5 mg, 0.4 mmol, 2.0 equiv), dry DCE (1.0 mL) and dry MeOH (0.5 mL) were added into it. The mixture was stirred for 1 hour at room temperature to form a white suspension. Then the tube was cooled to -10 ^oC, **1j** (43.9 mg, 0.2 mmol, 1.0 equiv) and $PhMe_2Si$-Bpin (105 mg, 0.4 mmol, 2.0 equiv) were added in sequence *via* syringe. It was continued to stir for 3.5 hours at -10 ^oC. The tube was opened quickly at room temperature, the final solution was filtered through celite using CH_2Cl_2 (30 mL) under air. The filtrate was concentrated under reduced pressure to afford the crude product, which was purified by flash column chromatography on silica gel (eluent: PE) to obtain the desired product **3j** (31.1 mg, 49%) as colorless oil. **^{1}H NMR (400 MHz, $CDCl_3$):** δ 7.55 – 7.52 (m, 2H), 7.38 – 7.33 (m, 4H), 7.15 – 7.07 (m, 2H), 6.92 – 6.90 (m, 1H), 6.04 (s, 1H), 0.47 (s, 3H), 0.46 (s, 3H). **^{13}C NMR (100 MHz, $CDCl_3$):** δ 206.35, 136.26, 134.76, 133.98, 132.39, 129.82, 129.61, 129.46, 128.14, 127.90, 126.53, 108.78, 86.72, -2.74, -2.85. **HRMS (EI):** m/z calculated for $C_{17}H_{16}SiCl_2[M]^+$: 318.0393, found: 318.0391. **IR** (KBr): 3068, 2958, 1921, 1489, 1428, 1312, 1252, 1208, 1113, 1011, 908, 830, 780, 733, 700.

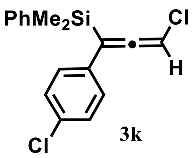

3k

3k: A dried screw-vial seal tube was charged with CuI (1.9 mg, 0.01 mmol, 5 mol %) in glove box, then moved it out of the glove box. The tube was equipped with an argon balloon, dry Et_3N (40.5 mg, 0.4 mmol, 2.0 equiv), dry DCE (1.0 mL) and dry MeOH (0.5 mL) were added into it. The mixture was stirred for 1 hour at room temperature to form a white suspension. Then the tube was cooled to -10 ^oC, **1k** (43.9 mg, 0.2 mmol, 1.0 equiv) and $PhMe_2Si$-Bpin (105 mg, 0.4 mmol, 2.0 equiv) were added in sequence *via* syringe. It was continued to stir for 4 hours at -10 ^oC. The tube was opened quickly at room temperature, the final solution was filtered through celite using CH_2Cl_2 (30 mL) under air. The filtrate was concentrated under reduced pressure to afford the crude product, which was purified by flash column chromatography on silica gel (eluent: PE) to obtain the desired product **3k** (38.4 mg, 60%) as yellow oil. **^{1}H NMR (400 MHz, $CDCl_3$):** δ 7.57 – 7.55 (m, 2H), 7.40 – 7.34 (m, 3H), 7.21 – 7.18 (m, 2H), 7.16 – 7.13 (m, 2H), 6.22 (s, 1H), 0.51 (s, 3H), 0.50 (s, 3H). **^{13}C NMR (100 MHz, $CDCl_3$):** δ 209.37, 138.71, 136.07, 135.74, 135.70, 131.92, 131.79, 130.95, 130.36, 112.22, 90.04, 0.08, 0.00. **HRMS (EI):** m/z calculated for $C_{17}H_{16}SiCl_2[M]^+$: 318.0393, found: 318.0392. **IR** (KBr): 3069, 3051, 2958, 1922, 1589, 1488, 1428, 1312, 1252, 1209, 1113, 1092, 1014, 905, 830, 781, 732, 701, 653.

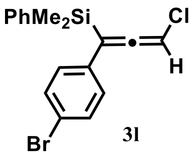

3l

3l: A dried screw-vial seal tube was charged with CuI (1.9 mg, 0.01 mmol, 5 mol %) in glove box, then moved it out of the glove box. The tube was equipped with an argon balloon, dry Et_3N (40.5 mg, 0.4 mmol, 2.0 equiv), dry DCE (1.0 mL) and dry MeOH (0.5 mL) were added into it. The mixture was stirred for 1 hour at room temperature to form a white suspension. Then the tube was cooled to -10 ^oC, **1l** (52.8 mg, 0.2 mmol, 1.0 equiv) and $PhMe_2Si$-Bpin (105 mg, 0.4 mmol, 2.0 equiv) were added in sequence *via* syringe. It was continued to stir for 4 hours at -10 ^oC. The tube was opened quickly at room temperature, the final solution was filtered through celite using CH_2Cl_2 (30 mL) under air. The filtrate was concentrated under reduced pressure to afford the crude product, which was purified by flash column chromatography on silica gel (eluent: PE) to obtain the desired product **3l** (37.8 mg, 52%) as yellow oil. **^{1}H NMR (400 MHz, $CDCl_3$):** δ 7.57 – 7.55 (m, 2H), 7.40 – 7.33 (m, 5H), 7.09 – 7.06 (m, 2H), 6.21 (s, 1H), 0.51 (s, 3H), 0.50 (s, 3H). **^{13}C NMR (100 MHz, $CDCl_3$):** δ 209.29, 138.61, 136.16, 135.99, 133.83, 132.02, 131.86, 130.29, 123.82, 112.21, 90.00, 0.00, -0.09. **HRMS (EI):** m/z calculated for $C_{17}H_{16}SiClBr[M]^+$: 361.9888, found: 361.9901. **IR** (KBr): 3068, 3050, 2958, 1921, 1584, 1484, 1427, 1397, 1311, 1252, 1207, 1111, 1073, 1010, 906, 824, 736, 703, 652.

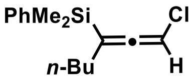

3m

3m: A dried screw-vial seal tube was charged with CuI (1.9 mg, 0.01 mmol, 5 mol %) in glove box, then moved it out of the glove box. The tube was equipped with an argon balloon, dry Et_3N (40.5 mg, 0.4 mmol, 2.0 equiv), dry DCE (1.0 mL) and dry MeOH (0.5 mL) were added into it. The mixture was stirred for 1 hour at room temperature to form a white suspension. Then the tube was cooled to -10 °C, **1m** (33 mg, 0.2 mmol, 1.0 equiv) and $PhMe_2Si$-Bpin (105 mg, 0.4 mmol, 2.0 equiv) were added in sequence *via* syringe. It was continued to stir for 4 hours at -10 °C. The tube was opened quickly at room temperature, the final solution was filtered through celite using CH_2Cl_2 (30 mL) under air. The filtrate was concentrated under reduced pressure to afford the crude product, which was purified by flash column chromatography on silica gel (eluent: PE) to obtain the desired product **3m** (39.8 mg, 75%) as slightly yellow oil. **^{1}H NMR (400 MHz, $CDCl_3$):** δ 7.53 – 7.51 (m, 2H), 7.39 – 7.35 (m, 3H), 5.91 (t, *J* = 2.4 Hz, 1H), 2.03 (tt, J_1 = 7.2 Hz, J_2 = 2.0 Hz, 2H), 1.40 (q, *J* = 7.4 Hz, 2H), 1.32 – 1.22 (m, 2H), 0.84 (t, *J* = 7.3 Hz), 0.42 (s, 6H). **^{13}C NMR (100 MHz, $CDCl_3$):** δ 203.67, 136.66, 133.85, 129.45, 127.90, 109.90, 86.70, 30.63, 29.64, 22.15, 13.84, -3.25, -3.35. **HRMS (EI):** m/z calculated for $C_{14}H_{18}SiCl[M-CH_3]^+$: 249.0861, found: 249.0870. **IR** (KBr): 3069, 2959, 2930, 1932, 1465, 1428, 1304, 1250, 1113, 1017, 835, 817, 780, 734, 700.

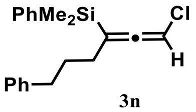

3n

3n: A dried screw-vial seal tube was charged with CuI (1.9 mg, 0.01 mmol, 5 mol %) in glove box, then moved it out of the glove box. The tube was equipped with an argon balloon, dry Et_3N (40.5 mg, 0.4 mmol, 2.0 equiv), dry DCE (1.0 mL) and dry MeOH (0.5 mL) were added into it. The mixture was stirred for 1 hour at room temperature to form a white suspension. Then the tube was cooled to -10 °C, **1n** (45 .4 mg, 0.2 mmol, 1.0 equiv) and $PhMe_2Si$-Bpin (105 mg, 0.4 mmol, 2.0 equiv) were added in sequence *via* syringe. It was continued to stir for 4 hours at -10 °C. The tube was opened quickly at room temperature, the final solution was filtered through celite using CH_2Cl_2 (30 mL) under air. The filtrate was concentrated under reduced pressure to afford the crude product, which was purified by flash column chromatography on silica gel (eluent: PE) to obtain the desired product **3n** (43 mg, 66%) as colorless oil. **^{1}H NMR (400 MHz, $CDCl_3$):** δ 7.54 – 7.50 (m, 2H), 7.39 - 7.35 (m, 3H), 7.24 – 7.22 (m, 2H), 7.18 – 7.15 (m, 1H), 7.11 – 7.09 (m, 2H), 5.96 – 5.93 (m, 1H), 2.62 – 2.50 (m, 2H), 2.10 – 2.06 (m, 2H), 1.80 – 1.72 (m, 2H), 0.43 (s, 3H), 0.42 (s, 3H). **^{13}C NMR (100 MHz, $CDCl_3$):** δ 203.74, 142.17, 136.54, 133.89, 129.55, 128.49, 128.31, 127.99, 125.76, 109.51, 87.05, 35.11, 30.22, 29.39, -3.25, -3.38. **HRMS (EI):** m/z calculated for $C_{20}H_{23}SiCl[M]^+$: 326.1252, found: 326.1257. **IR** (KBr): 3067, 3025, 2934, 2857, 1931, 1603, 1496, 1454, 1428, 1303, 1250, 1112, 1066, 1030, 997, 815, 780, 745, 699, 647.

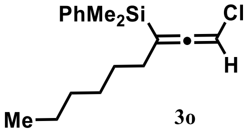

3o

3o: A dried screw-vial seal tube was charged with CuI (1.9 mg, 0.01 mmol, 5 mol %) in glove box, then moved it out of the glove box. The tube was equipped with an argon balloon, dry Et_3N (40.5 mg, 0.4 mmol, 2.0 equiv), dry DCE (1.0 mL) and dry MeOH (0.5 mL) were added into it. The mixture was stirred for 1 hour at room temperature to form a white suspension. Then the tube was cooled to -10 °C, **1o** (38.6 mg, 0.2 mmol, 1.0 equiv) and $PhMe_2Si$-Bpin (105 mg, 0.4 mmol, 2.0 equiv) were added in sequence *via* syringe. It was continued to stir for 4 hours at -10 °C. The tube was opened quickly at room temperature, the final solution was filtered through celite using CH_2Cl_2 (30 mL) under air. The filtrate was concentrated under reduced pressure to afford the crude product, which was purified by flash column chromatography on silica gel (eluent: PE) to obtain the desired product **3o** (44.7 mg, 76%) as yellow oil. **^{1}H NMR (400 MHz, $CDCl_3$):** δ 7.53 – 7.50 (m, 2H), 7.38 – 7.34 (m, 3H), 5.90 (t, J = 2.4 Hz, 1H), 2.02 (td, J_1 = 7.2 Hz, J_2 = 1.1 Hz, 2H), 1.44 – 1.38 (m, 2H), 1.28 – 1.18 (m, 6H), 0.85 (t, J = 6.7 Hz, 3H), 0.42 (s, 6H). **^{13}C NMR (100 MHz, $CDCl_3$):** δ 203.70, 136.68, 133.86, 129.46, 127.92, 109.92, 86.72, 31.62, 29.97, 28.75, 28.48, 22.60, 14.07, -3.23, -3.34. **HRMS (EI):** m/z calculated for $C_{17}H_{25}SiCl[M]^+$: 292.1409, found: 292.1415. **IR** (KBr): 3070, 2958, 2928, 2857, 1933, 1428, 1305, 1250, 1113, 835, 816, 780, 733, 699.

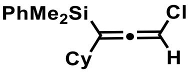

3p

3p: A dried screw-vial seal tube was charged with CuI (1.9 mg, 0.01 mmol, 5 mol %) in glove box, then moved it out of the glove box. The tube was equipped with an argon balloon, dry Et_3N (40.5 mg, 0.4 mmol, 2.0 equiv), dry DCE (1.0 mL) and dry MeOH (0.5 mL) were added into it. The mixture was stirred for 1 hour at room temperature to form a white suspension. Then the tube was cooled to -10 °C, **1p** (38.2 mg, 1.0 equiv) and $PhMe_2Si$-Bpin (105 mg, 0.4 mmol, 2.0 equiv) were added in sequence *via* syringe. It was continued to stir for 4 hours at -10 °C. The tube was opened quickly at room temperature, the final solution was filtered through celite using CH_2Cl_2 (30 mL) under air. The filtrate was concentrated under reduced pressure to afford the crude product, which was purified by flash column chromatography on silica gel (eluent: PE) to obtain the desired product **3p** (38.6 mg, 66%) as colorless oil. **^{1}H NMR (400 MHz, $CDCl_3$):** δ 7.54 – 7.51 (m, 2H), 7.40 – 7.33 (m, 3H), 5.91 (d, J = 1.4 Hz, 1H), 1.92 (t, J = 9.8 Hz, 1H), 1.71 – 1.66 (m, 4H), 1.59 (s, 1H), 1.22 – 1.08 (m, 5H), 0.42 (s, 3H), 0.42 (s, 3H). **^{13}C NMR (100 MHz, $CDCl_3$):** δ 203.93, 137.10, 133.85, 129.37, 127.86, 115.30, 87.11, 39.59, 33.57, 33.53, 26.40, 26.34, 25.93, -2.67, -2.76. **HRMS (EI):** m/z calculated for $C_{17}H_{23}SiCl[M]^+$: 290.1252, found: 290.1264. **IR** (KBr): 3069, 2926, 2852, 1926, 1448, 1428, 1286, 1250, 1113, 1022, 977, 819, 759, 730, 700, 651.

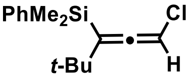

3q

3q: A dried screw-vial seal tube was charged with CuI (1.9 mg, 0.01 mmol, 5 mol %) in glove box, then moved it out of the glove box. The tube was equipped with an argon balloon, dry Et_3N (40.5 mg, 0.4 mmol, 2.0 equiv), dry DCE (1.0 mL) and dry MeOH (0.5 mL) were added into it. The mixture was stirred for 1 hour at room temperature to form a white

suspension. Then the tube was cooled to -10 °C, **1q** (33.0 mg, 0.2 mmol, 1.0 equiv) and $PhMe_2Si$-Bpin (105 mg, 0.4 mmol, 2.0 equiv) were added in sequence *via* syringe. It was continued to stir for 4 hours at -10 °C. The tube was opened quickly at room temperature, the final solution was filtered through celite using CH_2Cl_2 (30 mL) under air. The filtrate was concentrated under reduced pressure to afford the crude product, which was purified by flash column chromatography on silica gel (eluent: PE) to obtain the desired product **3q** (33.3 mg, 63%) as yellow oil. **^{1}H NMR (400 MHz, $CDCl_3$):** δ 7.55 – 7.53 (m, 2H), 7.36 – 7.33 (m, 3H), 5.89 (s, 1H), 1.03 (s, 9H), 0.49 (s, 3H), 0.47 (s, 3H). **^{13}C NMR (100 MHz, $CDCl_3$):** δ 204.78, 138.94, 134.75, 130.05, 128.65, 120.69, 87.66, 37.20, 31.81, 0.22, 0.00. **HRMS (EI):** m/z calculated for $C_{14}H_{18}SiCl[M-CH_3]^+$: 249.0861, found: 249.0864. **IR** (KBr): 3069, 2964, 1933, 1428, 1363, 1292, 1113, 953, 837, 820, 777, 745, 701, 657.

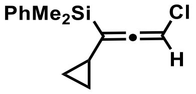

3r

3r: A dried screw-vial seal tube was charged with CuI (1.9 mg, 0.01 mmol, 5 mol %) in glove box, then moved it out of the glove box. The tube was equipped with an argon balloon, dry Et_3N (40.5 mg, 0.4 mmol, 2.0 equiv), dry DCE (1.0 mL) and dry MeOH (0.5 mL) were added into it. The mixture was stirred for 1 hour at room temperature to form a white suspension. Then the tube was cooled to -10 °C, **1r** (29.8 mg, 0.2 mmol, 1.0 equiv) and $PhMe_2Si$-Bpin (105 mg, 0.4 mmol, 2.0 equiv) were added in sequence *via* syringe. It was continued to stir for 4 hours at -10 °C. The tube was opened quickly at room temperature, the final solution was filtered through celite using CH_2Cl_2 (30 mL) under air. The filtrate was concentrated under reduced pressure to afford the crude product, which was purified by flash column chromatography on silica gel (eluent: PE) to obtain the desired product **3r** (34.1 mg, 69%) as colorless oil. **^{1}H NMR (400 MHz, $CDCl_3$):** δ 7.58 – 7.55 (m, 2H), 7.40 – 7.36 (m, 3H), 5.93 (d, J = 1.0 Hz, 1H), 1.17 – 1.10 (m, 1H), 0.78 – 0.68 (m, 2H), 0.55 – 0.47 (m, 2H), 0.46 (s, 3H), 0.46 (s, 3H). **^{13}C NMR (100 MHz, $CDCl_3$):** δ 201.24, 136.67, 133.95, 129.48, 127.90, 114.97, 87.98, 10.29, 9.55, 9.30, -3.02, -3.11. **HRMS (EI):** m/z calculated for $C_{14}H_{17}SiCl[M]^+$: 248.0783, found: 248.0791. **IR** (KBr): 3070, 3003, 2960, 2926, 1930, 1428, 1315, 1250, 1201, 1113, 999, 855, 833, 814, 779, 735, 700.

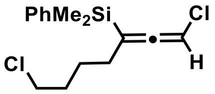

3s

3s: A dried screw-vial seal tube was charged with CuI (1.9 mg, 0.01 mmol, 5 mol %) in glove box, then moved it out of the glove box. The tube was equipped with an argon balloon, dry Et_3N (40.5 mg, 0.4 mmol, 2.0 equiv), dry DCE (1.0 mL) and dry MeOH (0.5 mL) were added into it. The mixture was stirred for 1 hour at room temperature to form a white suspension. Then the tube was cooled to -10 °C, **1s** (39.9 mg, 0.2 mmol, 1.0 equiv) and $PhMe_2Si$-Bpin (105 mg, 0.4 mmol, 2.0 equiv) were added in sequence *via* syringe. It was continued to stir for 3.5 hours at -10 °C. The tube was opened quickly at room temperature, the final solution was filtered through celite using CH_2Cl_2 (30 mL) under air. The filtrate was concentrated under reduced pressure to afford the crude product, which was purified by flash column

chromatography on silica gel (eluent: PE) to obtain the desired product **3s** (57.4 mg, 96%) as slightly yellow oil. **^{1}H NMR (400 MHz, CDCl$_3$):** δ 7.53 – 7.50 (m, 2H), 7.39 – 7.34 (m, 3H), 5.94 (t, J = 2.4 Hz, 1H), 3.45 (t, J = 6.6 Hz, 2H), 2.06 (td, J_1 = 7.2 Hz, J_2 = 2.4 Hz, 2H), 1.77 – 1.68 (m, 2H), 1.61 – 1.56 (m, 2H), 0.43 (s, 6H). **^{13}C NMR (100 MHz, CDCl$_3$):** δ 203.73, 136.37, 133.83, 129.59, 128.00, 109.29, 87.12, 44.75, 31.80, 29.09, 25.64, -3.31, -3.43. **HRMS (EI):** m/z calculated for $C_{14}H_{17}SiCl_2$[M-CH$_3$]$^+$: 283.0471, found: 283.0480. **IR** (KBr): 3069, 2957, 1933, 1447, 1428, 1305, 1251, 1112, 997, 835, 816, 781, 735, 701, 649.

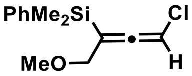

3t

3t: A dried screw-vial seal tube was charged with CuI (1.9 mg, 0.01 mmol, 5 mol %) in glove box, then moved it out of the glove box. The tube was equipped with an argon balloon, dry Et$_3$N (40.5 mg, 0.4 mmol, 2.0 equiv), dry DCE (1.0 mL) and dry MeOH (0.5 mL) were added into it. The mixture was stirred for 1 hour at room temperature to form a white suspension. Then the tube was cooled to -10 ^{o}C, **1t** (30.6 mg, 0.2 mmol, 1.0 equiv) and PhMe$_2$Si-Bpin (105 mg, 0.4 mmol, 2.0 equiv) were added in sequence *via* syringe. It was continued to stir for 3.5 hours at -10 ^{o}C. The tube was opened quickly at room temperature, the final solution was filtered through celite using CH$_2$Cl$_2$ (30 mL) under air. The filtrate was concentrated under reduced pressure to afford the crude product, which was purified by flash column chromatography on silica gel (eluent: PE/Et$_2$O = 97:3) to obtain the desired product **3t** (41.5 mg, 82%) as colorless oil. **^{1}H NMR (400 MHz, CDCl$_3$):** δ 7.56 – 7.53 (m, 2H), 7.39 – 7.36 (m, 3H), 5.98 (t, J = 2.1 Hz, 1H), 4.04 – 3.96 (m, 2H), 3.25 (s, 3H), 0.46 (s, 6H). **^{13}C NMR (100 MHz, CDCl$_3$):** δ 203.97, 136.13, 133.86, 129.57, 127.93, 107.38, 86.99, 71.15, 58.10, -3.08, -3.12. **HRMS (EI):** m/z calculated for $C_{13}H_{17}SiClO$[M]$^+$: 252.0732, found: 252.0740. **IR** (KBr): 3069, 2925, 2821, 1940, 1912, 1428, 1369, 1311, 1250, 1192, 1113, 969, 910, 837, 818, 781, 734, 700.

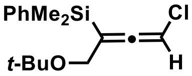

3u

3u: A dried screw-vial seal tube was charged with CuI (1.9 mg, 0.01 mmol, 5 mol %) in glove box, then moved it out of the glove box. The tube was equipped with an argon balloon, dry Et$_3$N (40.5 mg, 0.4 mmol, 2.0 equiv), dry DCE (1.0 mL) and dry MeOH (0.5 mL) were added into it. The mixture was stirred for 1 hour at room temperature to form a white suspension. Then the tube was cooled to -10 ^{o}C, and **1u** (39.0 mg, 0.2 mmol, 1.0 equiv) and PhMe$_2$Si-Bpin (105 mg, 0.4 mmol, 2.0 equiv) were added in sequence *via* syringe. It was continued to stir for 4 hours at -10 ^{o}C. The tube was opened quickly at room temperature, the final solution was filtered through celite using CH$_2$Cl$_2$ (30 mL) under air. The filtrate was concentrated under reduced pressure to afford the crude product, which was purified by flash column chromatography on silica gel (eluent: PE/Et$_2$O = 98:2) to obtain the desired product **3u** (56.8 mg, 96%) as slightly yellow oil. **^{1}H NMR (400 MHz, CDCl$_3$):** δ 7.57 – 7.54 (m, 2H), 7.38 – 7.25 (m, 3H), 5.93 (t, J = 2.1 Hz, 1H), 4.01 (d, J = 2.1 Hz, 2H), 1.09 (s, 9H), 0.46 (s, 3H), 0.45 (s, 3H). **^{13}C NMR (100 MHz, CDCl$_3$):** δ 203.71, 136.73, 134.00, 129.34, 127.76,

109.26, 86.67, 73.66, 61.43, 27.34, -2.62, -2.66. **HRMS (EI):** m/z calculated for $C_{15}H_{20}SiClO[M-CH_3]^+$: 279.0966, found: 279.0968. **IR** (KBr): 3070, 2974, 2933, 1940, 1428, 1389, 1363, 1304, 1249, 1194, 1114, 1076, 880, 837, 817, 781, 733, 700.

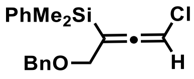

3v

3v: A dried screw-vial seal tube was charged with CuI (1.9 mg, 0.01 mmol, 5 mol %) in glove box, then moved it out of the glove box. The tube was equipped with an argon balloon, dry Et_3N (40.5 mg, 0.4 mmol, 2.0 equiv), dry DCE (1.0 mL) and dry MeOH (0.5 mL) were added into it. The mixture was stirred for 1 hour at room temperature to form a white suspension. Then the tube was cooled to -10 °C, and **1v** (45.8 mg, 0.2 mmol, 1.0 equiv) and $PhMe_2Si$-Bpin (105 mg, 0.4 mmol, 2.0 equiv) were added in sequence *via* syringe. It was continued to stir for 4 hours at -10 °C. The tube was opened quickly at room temperature, the final solution was filtered through celite using CH_2Cl_2 (30 mL) under air. The filtrate was concentrated under reduced pressure to afford the crude product, which was purified by flash column chromatography on silica gel (eluent: PE/Et_2O = 98:2) to obtain the desired product **3v** (61.3 mg, 93%) as yellow oil. **^{1}H NMR (400 MHz, $CDCl_3$):** δ 7.55 – 7.52 (m, 2H), 7.39 – 7.32 (m, 4H), 7.31 – 7.27 (m, 2H), 7.24 – 7.22 (m, 2H), 5.99 (t, *J* = 2.0 Hz, 1H), 4.41 (q, *J* = 11.8 Hz, 2H), 4.16 – 4.08 (m, 2H), 0.46 (s, 6H). **^{13}C NMR (100 MHz, $CDCl_3$):** δ 204.34, 137.81, 136.19, 133.91, 129.55, 128.33, 127.94, 127.85, 127.63, 107.42, 87.00, 72.09, 68.76, -2.98, -3.05. **HRMS (EI):** m/z calculated for $C_{19}H_{21}SiO[M-Cl]^+$: 293.1356, found: 293.1363. **IR** (KBr): 3067, 2958, 2852, 1940, 1911, 1455, 1428, 1350, 1304, 1250, 1113, 837, 817, 781, 734, 698.

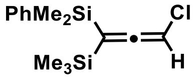

3w

3w: A dried screw-vial seal tube was charged with CuI (3.8 mg, 0.01 mmol, 10 mol %) in glove box, then moved it out of the glove box. The tube was equipped with an argon balloon, dry Et_3N (40.5 mg, 0.4 mmol, 2.0 equiv), dry DCE (1.0 mL) and dry MeOH (0.5 mL) were added into it. The mixture was stirred for 1 hour at room temperature to form a white suspension. Then the tube was cooled to -10 °C, **1w** (36.2 mg, 0.2 mmol, 1.0 equiv) and $PhMe_2Si$-Bpin (105 mg, 0.4 mmol, 2.0 equiv) were added in sequence *via* syringe. It was continued to stir for 3.5 hours at -10 °C. The tube was opened quickly at room temperature, the final solution was filtered through celite using CH_2Cl_2 (30 mL) under air. The filtrate was concentrated under reduced pressure to afford the crude product, which was purified by flash column chromatography on silica gel (eluent: PE) to obtain the desired product **3w** (21.2 mg, 38%) as slightly yellow oil. **^{1}H NMR (400 MHz, $CDCl_3$):** δ 7.54 – 7.51 (m, 2H), 7.39 – 7.35 (m, 3H), 5.66 (s, 1H), 0.46 (s, 6H), 0.04 (s, 9H). **^{13}C NMR (100 MHz, $CDCl_3$):** δ 209.09, 137.76, 134.19, 129.71, 128.14, 103.49, 81.16, 0.00, -1.42, -1.49. **HRMS (EI):** m/z calculated for $C_{14}H_{21}ClSi_2[M]^+$: 280.0865, found: 280.0879. **IR** (KBr): 3070, 2959, 1922, 1429, 1273, 1251, 1112, 883, 842, 807, 751, 732, 700.

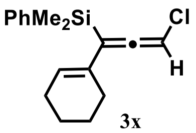

3x

3x: A dried screw-vial seal tube was charged with CuI (1.9 mg, 0.01 mmol, 5 mol %) in glove box, then moved it out of the glove box. The tube was equipped with an argon balloon, dry Et_3N (40.5 mg, 0.4 mmol, 2.0 equiv), dry DCE (1.0 mL) and dry MeOH (0.5 mL) were added into it. The mixture was stirred for 1 hour at room temperature to form a white suspension. Then the tube was cooled to -10 °C, **1x** (37.8 mg, 0.2 mmol, 1.0 equiv) and $PhMe_2Si$-Bpin (105 mg, 0.4 mmol, 2.0 equiv) were added in sequence *via* syringe. It was continued to stir for 3 hours at -10 °C. The tube was opened quickly at room temperature, the final solution was filtered through celite using CH_2Cl_2 (30 mL) under air. The filtrate was concentrated under reduced pressure to afford the crude product, which was purified by flash column chromatography on silica gel (eluent: PE) to obtain the desired product **3x** (25.9 mg, 45%) as yellow oil. **^{1}H NMR (400 MHz, $CDCl_3$):** δ 7.55 – 7.52 (m, 2H), 7.38 – 7.34 (m, 3H), 6.03 (s, 1H), 5.70 (t, J = 3.6 Hz, 1H), 2.13 – 2.10 (m, 2H), 2.02 – 1.99 (m, 2H), 1.66 – 1.60 (m, 2H), 1.55 – 1.48 (m, 2H), 0.46 (s, 6H). **^{13}C NMR (100 MHz, $CDCl_3$):** δ 207.36, 139.25, 135.32, 133.78, 132.28, 130.76, 129.39, 114.53, 88.97, 29.59, 27.69, 24.25, 23.47, 0.00, -0.06. **HRMS (EI):** m/z calculated for $C_{17}H_{21}SiCl[M]^+$: 288.1096, found: 288.1097. **IR** (KBr): 3068, 2931, 2858, 1909, 1709, 1585, 1428, 1311, 1250, 1188, 1113, 998, 838, 777, 731, 700, 652.

4.2 The results of reactions between different silylboronates with 1a.

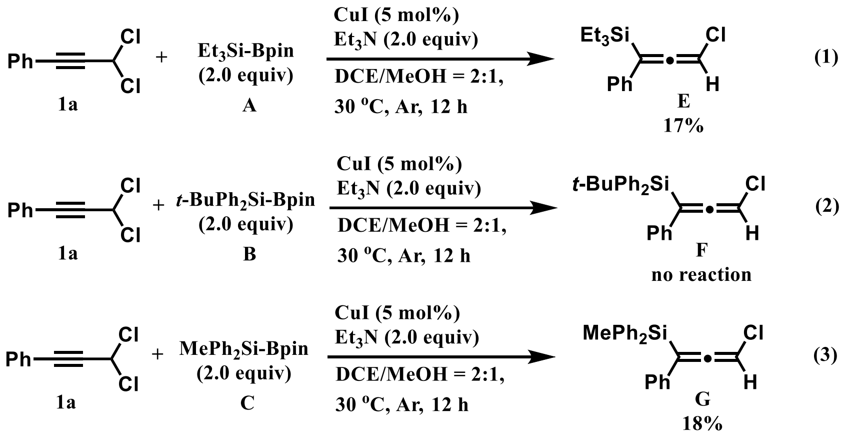

Procedures for the reaction between **1a** and silylboronate **A**:

E: A dried seal tube was charged with CuI (1.9 mg, 0.01 mmol, 5 mol %) in glove box, then moved it out of the glove box. The tube was equipped with an argon balloon, dry Et_3N (40.5 mg, 0.4 mmol, 2.0 equiv), dry DCE (1.0 mL) and dry MeOH (0.5 mL) were added into it. The mixture was stirred for 1 hour at room temperature to form a white suspension. Then **1a** (37.0 mg, 0.2 mmol, 1.0 equiv) and Et_3Si-Bpin (96.9 mg, 0.4 mmol, 2.0 equiv) were added in sequence *via* syringe. It was continued to stir for 12 hours at 30 °C. The tube was opened quickly at room temperature, the final solution was filtered through celite using CH_2Cl_2 (30

mL) under air. The filtrate was concentrated under reduced pressure to afford the crude product, which was purified by preparative thin-layer chromatography (PTLC) (eluent: distilled PE) to obtain the desired product **E** (9.1 mg, 17%) as slightly yellow oil. **^{1}H NMR (400 MHz, $CDCl_3$):** δ 7.35 – 7.28 (m, 4H), 7.27 – 7.23 (m, 1H), 6.12 (s, 1H), 0.97 (t, *J* = 8.0 Hz, 9H), 0.81 – 0.75 (m, 6H). **^{13}C NMR (100 MHz, $CDCl_3$):** δ 206.57, 136.10, 128.61, 127.93, 127.39, 109.74, 86.55, 7.23, 3.78. **HRMS (EI):** m/z calculated for $C_{15}H_{21}Si[M-Cl]^+$: 229.1407, found: 229.1398. **IR** (KBr): 3056, 2956, 2876, 1922, 1490, 1458, 1416, 1305, 1239, 1210, 1128, 1004, 914, 896, 764, 736, 694.

Procedures for the reaction between **1a** and silylboronate **C**:

G: A dried seal tube was charged with CuI (1.9 mg, 0.01 mmol, 5 mol %) in glove box, then moved it out of the glove box. The tube was equipped with an argon balloon, dry Et_3N (40.5 mg, 0.4 mmol, 2.0 equiv), dry DCE (1.0 mL) and dry MeOH (0.5 mL) were added into it. The mixture was stirred for 1 hour at room temperature to form a white suspension. Then **1a** (37.0 mg, 0.2 mmol, 1.0 equiv) and $MePh_2Si$-Bpin (129.7 mg, 0.4 mmol, 2.0 equiv) were added in sequence *via* syringe. It was continued to stir for 12 hours at 30 °C. The tube was opened quickly at room temperature, the final solution was filtered through celite using CH_2Cl_2 (30 mL) under air. The filtrate was concentrated under reduced pressure to afford the crude product, which was purified by preparative thin-layer chromatography (PTLC) (eluent: distilled PE) to obtain the desired product **G** (11.7 mg, 18%) as yellow oil. **^{1}H NMR (400 MHz, $CDCl_3$):** δ 7.59 – 7.56 (m, 4H), 7.43 – 7.34 (m, 8H), 7.24 – 7.18 (m, 3H), 6.06 (s, 1H), 0.77 (s, 3H). **^{13}C NMR (100 MHz, $CDCl_3$):** δ 208.59, 135.06, 135.03, 134.68, 134.63, 129.80, 129.79, 128.65, 128.53, 127.99, 127.98, 127.80, 127.67, 109.69, 88.12, -2.75. **HRMS (EI):** m/z calculated for $C_{22}H_{17}DClSi[M-H]^+$: 346.0924, found: 346.0929. **IR** (KBr): 3068, 2924, 2853, 1922, 1489, 1428, 1303, 1259, 1112, 1029, 916, 793, 726, 697.

5 General Procedures for the Synthesis of Enantioenriched Silyl-substituted Allenes (GP3)

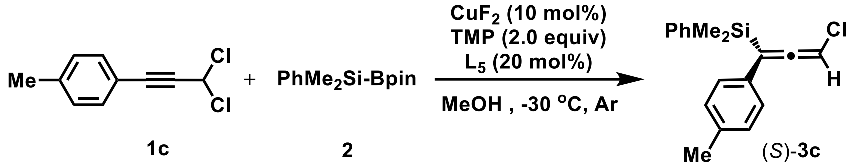

(*S*)-**3c:** A dried screw-vial seal tube was charged with CuF_2 (2.0 mg, 0.02 mmol, 10 mol %) and ligand $\mathbf{L_5}$ (19.5 mg, 0.04 mmol, 20 mol%) in glove box, then moved it out of the glove box. The tube was equipped with an argon balloon, TMP (56.5 mg, 0.4 mmol, 2.0 equiv) and extra dry MeOH (1.0 mL) were added into it. The mixture was stirred for 1 hour at room temperature to form a white solution. Then the tube was cooled to -30 °C, **1c** (39.8 mg, 0.2 mmol, 1.0 equiv) and $PhMe_2Si$-Bpin (105 mg, 0.4 mmol, 2.0 equiv) were added in sequence via syringe. It was continued to stir for 4 hours at -30 °C. The tube was opened quickly at room temperature, the final solution was filtered through celite using CH_2Cl_2 (30 mL) under air. The filtrate was concentrated under reduced pressure to afford the crude product, which

was purified by flash column chromatography on silica gel (eluent: PE) to obtain the desired product (*S*)-**3c** (36.8 mg, 62%) as yellow oil.

$[\alpha]_D^{20}$ +208.9° (c = 1.19, CH_2Cl_2)

The enantiomeric excess of (*S*)-**3c** was determined by chiral HPLC analysis on Chiralcel OJ column. Conditions: hexane/isopropanol = 99:1, flow rate = 0.5 mL/min, UV-Vis detection at λ = 273 nm, t_{R1} = 10.2 min (minor), t_{R2} = 16.1 min (major), 20 °C, ee = 90%.

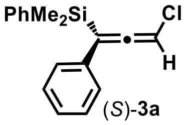

(*S*)-**3a**

(*S*)-**3a:** A dried screw-vial seal tube was charged with CuF_2 (2.0 mg, 0.02 mmol, 10 mol %) and ligand **L**$_5$ (19.5 mg, 0.04 mmol, 20 mol%) in glove box, then moved it out of the glove box. The tube was equipped with an argon balloon, TMP (56.5 mg, 0.4 mmol, 2.0 equiv) and extra dry MeOH (1.0 mL) were added into it. The mixture was stirred for 1 hour at room temperature to form a white solution. Then the tube was cooled to -30 °C, **1a** (37 mg, 0.2 mmol, 1.0 equiv) and $PhMe_2Si$-Bpin (105 mg, 0.4 mmol, 2.0 equiv) were added in sequence via syringe. It was continued to stir for 4 hours at -30 °C. The tube was opened quickly at room temperature, the final solution was filtered through celite using CH_2Cl_2 (30 mL) under air. The filtrate was concentrated under reduced pressure to afford the crude product, which was purified by flash column chromatography on silica gel (eluent: PE) to obtain the desired product (*S*)-**3a** (33.2 mg, 56%) as yellow oil.

$[\alpha]_D^{20}$ +199.7° (c = 1.00, CH_2Cl_2)

The enantiomeric excess of (*S*)-**3a** was determined by chiral HPLC analysis on Chiralcel OJ column. Conditions: hexane/isopropanol = 99:1, flow rate = 0.5 mL/min, UV-Vis detection at λ = 273 nm, t_{R1} = 10.4 min (minor), t_{R2} = 14.3 min (major), 20 °C, ee = 89%.

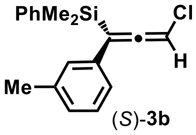

(*S*)-**3b**

(*S*)-**3b:** A dried screw-vial seal tube was charged with CuF_2 (2.0 mg, 0.02 mmol, 10 mol %) and ligand **L**$_5$ (19.5 mg, 0.04 mmol, 20 mol%) in glove box, then moved it out of the glove box. The tube was equipped with an argon balloon, TMP (56.5 mg, 0.4 mmol, 2.0 equiv) and extra dry MeOH (1.0 mL) were added into it. The mixture was stirred for 1 hour at room temperature to form a white solution. Then the tube was cooled to -30 °C, **1b** (39.8 mg, 0.2 mmol, 1.0 equiv) and $PhMe_2Si$-Bpin (105 mg, 0.4 mmol, 2.0 equiv) were added in sequence *via* syringe. It was continued to stir for 4.5 hours at -30 °C. The tube was opened quickly at room temperature, the final solution was filtered through celite using CH_2Cl_2 (30 mL) under air. The filtrate was concentrated under reduced pressure to afford the crude product, which was purified by flash column chromatography on silica gel (eluent: PE) to obtain the desired product (*S*)-**3b** (48.2 mg, 81%) as yellow oil.

$[\alpha]_D^{20}$ +92.8° (c = 1.19, CH_2Cl_2)

The enantiomeric excess of (*S*)-**3b** was determined by chiral HPLC analysis on Chiralcel OD-H column. Conditions: hexane/isopropanol = 100:0, flow rate = 0.6 mL/min, UV-Vis

detection at λ = 210 nm, t_{R1} = 13.3 min (major), t_{R2} = 22.4 min (minor), 20 °C, ee = 91%.

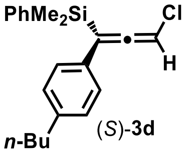

(*S*)-**3d**

(*S*)-**3d:** A dried screw-vial seal tube was charged with CuF_2 (2.0 mg, 0.02 mmol , 10 mol %) and ligand $\mathbf{L_5}$ (19.5 mg, 0.04 mmol, 20 mol%) in glove box, then moved it out of the glove box. The tube was equipped with an argon balloon, TMP (56.5 mg, 0.4 mmol, 2.0 equiv) and extra dry MeOH (1.0 mL) were added into it. The mixture was stirred for 1 hour at room temperature to form a white solution. Then the tube was cooled to -30 °C, **1d** (48.2 mg, 0.2 mmol, 1.0 equiv) and $PhMe_2Si$-Bpin (105 mg, 0.4 mmol, 2.0 equiv) were added in sequence via syringe. It was continued to stir for 7 hours at -30 °C. The tube was opened quickly at room temperature, the final solution was filtered through celite using CH_2Cl_2 (30 mL) under air. The filtrate was concentrated under reduced pressure to afford the crude product, which was purified by flash column chromatography on silica gel (eluent: PE) to obtain the desired product (*S*)-**3d** (35.4 mg, 52%) as yellow oil.

$[\alpha]_D^{20}$ +110.9° (c = 1.02, CH_2Cl_2)

The enantiomeric excess of (*S*)-**3d** was determined by chiral HPLC analysis on Chiralcel OD-H column. Conditions: hexane/isopropanol = 100:0, flow rate = 0.6 mL/min, UV-Vis detection at λ = 210 nm, t_{R1} = 12.1 min (major), t_{R2} = 15.3 min (minor), 20 °C, ee = 88%.

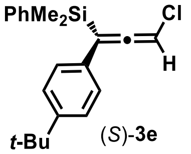

(*S*)-**3e**

(*S*)-**3e:** A dried screw-vial seal tube was charged with CuF_2 (2.0 mg, 0.02 mmol, 10 mol %) and ligand $\mathbf{L_5}$ (19.5 mg, 0.04 mmol, 20 mol %) in glove box, then moved it out of the glove box. The tube was equipped with an argon balloon, TMP (56.5 mg, 0.4 mmol, 2.0 equiv) and extra dry MeOH (1.0 mL) were added into it. The mixture was stirred for 1 hour at room temperature to form a white solution. Then the tube was cooled to -30 °C, **1e** (48.2 mg, 0.2 mmol, 1.0 equiv) and $PhMe_2Si$-Bpin (105 mg, 0.4 mmol, 2.0 equiv) were added in sequence via syringe. It was continued to stir for 15 hours at -30 °C. The tube was opened quickly at room temperature, the final solution was filtered through celite using CH_2Cl_2 (30 mL) under air. The filtrate was concentrated under reduced pressure to afford the crude product, which was purified by flash column chromatography on silica gel (eluent: PE) to obtain the desired product (*S*)-**3e** (47.7 mg, 70%) as yellow oil.

$[\alpha]_D^{20}$ +100.3° (c = 1.06, CH_2Cl_2)

The enantiomeric excess of (*S*)-**3e** was determined by chiral HPLC analysis on Chiralcel OJ column. Conditions: hexane/isopropanol = 95:5, flow rate = 0.6 mL/min, UV-Vis detection at λ = 210 nm, t_{R1} = 5.6 min (minor), t_{R2} = 6.9 min (major), 20 °C, ee = 88%.

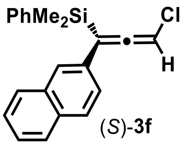

(*S*)-**3f**

(*S*)-**3f:** A dried screw-vial seal tube was charged with CuF_2 (2.0 mg, 0.02 mmol, 10 mol %) and ligand $\mathbf{L_5}$ (19.5 mg, 0.04 mmol, 20 mol %) in glove box, then moved it out of the glove box. The tube was equipped with an argon balloon, TMP (56.5 mg, 0.4 mmol, 2.0 equiv) and extra dry MeOH (1.0 mL) were added into it. The mixture was stirred for 1 hour at room temperature to form a white solution. Then the tube was cooled to -30 ^{o}C, **1f** (47.0 mg, 0.2 mmol, 1.0 equiv) and $PhMe_2Si$-Bpin (105 mg, 0.4 mmol, 2.0 equiv) were added in sequence via syringe. It was continued to stir for 15 hours at -30 ^{o}C. The tube was opened quickly at room temperature, the final solution was filtered through celite using CH_2Cl_2 (30 mL) under air. The filtrate was concentrated under reduced pressure to afford the crude product, which was purified by flash column chromatography on silica gel (eluent: PE) to obtain the desired product (*S*)-**3f** (41.5 mg, 62%) as yellow oil.

$[\alpha]_D{}^{20}$ +151.2^{o} (c = 1.21, CH_2Cl_2)

The enantiomeric excess of (*S*)-**3f** was determined by chiral HPLC analysis on Chiralcel OJ column. Conditions: hexane/isopropanol = 98:2, flow rate = 0.5 mL/min, UV-Vis detection at λ = 273 nm, t_{R1} = 13.6 min (minor), t_{R2} = 26.4 min (major), 20 ^{o}C, ee = 90%.

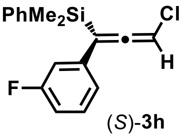

(*S*)-**3h**

(*S*)-**3h:** A dried screw-vial seal tube was charged with CuF_2 (2.0 mg, 0.02 mmol, 10 mol %) and ligand $\mathbf{L_5}$ (19.5 mg, 0.04 mmol, 20 mol %) in glove box, then moved it out of the glove box. The tube was equipped with an argon balloon, TMP (56.5 mg, 0.4 mmol, 2.0 equiv) and extra dry MeOH (1.0 mL) were added into it. The mixture was stirred for 1 hour at room temperature to form a white solution. Then the tube was cooled to -30 ^{o}C, **1h** (40.6 mg, 0.2 mmol, 1.0 equiv) and $PhMe_2Si$-Bpin (105 mg, 0.4 mmol, 2.0 equiv) were added in sequence via syringe. It was continued to stir for 10 hours at -30 ^{o}C. The tube was opened quickly at room temperature, the final solution was filtered through celite using CH_2Cl_2 (30 mL) under air. The filtrate was concentrated under reduced pressure to afford the crude product, which was purified by flash column chromatography on silica gel (eluent: PE) to obtain the desired product (*S*)-**3h** (29 mg, 48%) as yellow oil.

$[\alpha]_D{}^{20}$ +37.7^{o} (c = 1.71, CH_2Cl_2)

The enantiomeric excess of (*S*)-**3h** was determined by chiral HPLC analysis on Chiralcel OD-H column. Conditions: hexane/isopropanol = 100:0, flow rate = 0.6 mL/min, UV-Vis detection at λ = 210 nm, t_{R1} = 13.7 min (major), t_{R2} = 15.7 min (minor), 20 ^{o}C, ee = 86%.

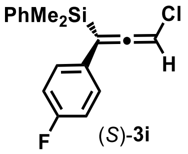

(*S*)-**3i**

(*S*)-**3i:** A dried screw-vial seal tube was charged with CuF_2 (2.0 mg, 0.02 mmol, 10 mol %) and ligand $\mathbf{L_5}$ (19.5 mg, 0.04 mmol, 20 mol %) in glove box, then moved it out of the glove box. The tube was equipped with an argon balloon, TMP (56.5 mg, 0.4 mmol, 2.0 equiv) and extra dry MeOH (1.0 mL) were added into it. The mixture was stirred for 1 hour at room temperature to form a white solution. Then the tube was cooled to -30 °C, **1i** (40.6 mg, 0.2 mmol, 1.0 equiv) and $PhMe_2Si$-Bpin (105 mg, 0.4 mmol, 2.0 equiv) were added in sequence via syringe. It was continued to stir for 4 hours at -30 °C. The tube was opened quickly at room temperature, the final solution was filtered through celite using CH_2Cl_2 (30 mL) under air. The filtrate was concentrated under reduced pressure to afford the crude product, which was purified by flash column chromatography on silica gel (eluent: PE) to obtain the desired product (*S*)-**3i** (33.3 mg, 55%) as yellow oil.
$[\alpha]_D^{20}$ +216.9° (c = 1.06, CH_2Cl_2)
The enantiomeric excess of (*S*)-**3i** was determined by chiral HPLC analysis on Chiralcel OD-H column. Conditions: hexane/isopropanol = 100:0, flow rate = 0.6 mL/min, UV-Vis detection at λ = 210 nm, t_{R1} = 11.3 min (major), t_{R2} = 13.0 min (minor), 20 °C, ee = 86%.

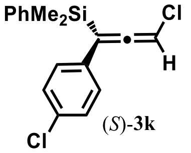

(*S*)-**3k**

(*S*)-**3k:** A dried screw-vial seal tube was charged with CuF_2 (2.0 mg, 0.02 mmol, 10 mol %) and ligand $\mathbf{L_5}$ (19.5 mg, 0.04 mmol, 20 mol %) in glove box, then moved it out of the glove box. The tube was equipped with an argon balloon, TMP (56.5 mg, 0.4 mmol, 2.0 equiv) and extra dry MeOH (1.0 mL) were added into it. The mixture was stirred for 1 hour at room temperature to form a white solution. Then the tube was cooled to -30 °C, **1k** (43.9 mg, 0.2 mmol, 1.0 equiv) and $PhMe_2Si$-Bpin (105 mg, 0.4 mmol, 2.0 equiv) were added in sequence via syringe. It was continued to stir for 4 hours at -30 °C. The tube was opened quickly at room temperature, the final solution was filtered through celite using CH_2Cl_2 (30 mL) under air. The filtrate was concentrated under reduced pressure to afford the crude product, which was purified by flash column chromatography on silica gel (eluent: PE) to obtain the desired product (*S*)-**3k** (42.4 mg, 66%) as yellow oil.
$[\alpha]_D^{20}$ +256.6° (c = 1.10, CH_2Cl_2)
The enantiomeric excess of (*S*)-**3k** was determined by chiral HPLC analysis on Chiralcel OD-H column. Conditions: hexane/isopropanol = 100:0, flow rate = 0.6 mL/min, UV-Vis detection at λ = 210 nm, t_{R1} = 12.5 min (major), t_{R2} = 14.3 min (minor), 20 °C, ee = 86%.

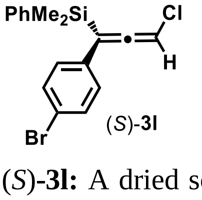

(*S*)-**3l**

(*S*)-**3l:** A dried screw-vial seal tube was charged with CuF_2 (2.0 mg, 0.02 mmol, 10 mol %) and ligand $\mathbf{L_5}$ (19.5 mg, 0.04 mmol, 20 mol %) in glove box, then moved it out of the glove box. The tube was equipped with an argon balloon, TMP (56.5 mg, 0.4 mmol, 2.0 equiv) and extra dry MeOH (1.0 mL) were added into it. The mixture was stirred for 1 hour at room

temperature to form a white solution. Then the tube was cooled to -30 ºC, **1l** (52.8 mg, 0.2 mmol, 1.0 equiv) and PhMe$_2$Si-Bpin (105 mg, 0.4 mmol, 2.0 equiv) were added in sequence *via* syringe. It was continued to stir for 15 hours at -30 ºC. The tube was opened quickly at room temperature, the final solution was filtered through celite using CH_2Cl_2 (30 mL) under air. The filtrate was concentrated under reduced pressure to afford the crude product, which was purified by flash column chromatography on silica gel (eluent: PE) to obtain the desired product (*S*)-**3l** (45.3 mg, 62%) as yellow oil.

$[\alpha]_D^{20}$ -54.1º (c = 0.63, CH_2Cl_2)

The enantiomeric excess of (*S*)-**3l** was determined by chiral HPLC analysis on Chiralcel OJ column. Conditions: hexane/isopropanol = 95:5, flow rate = 0.6 mL/min, UV-Vis detection at λ = 273 nm, t_{R1} = 6.9 min (minor), t_{R2} = 9.5 min (major), 20 ºC, ee = 84%.

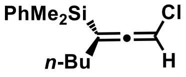

(*S*)-**3m**

(*S*)-**3m:** A dried screw-vial seal tube was charged with CuF_2 (2.0 mg, 0.02 mmol, 10 mol %) and ligand **L**$_5$ (19.5 mg, 0.04 mmol, 20 mol %) in glove box, then moved it out of the glove box. The tube was equipped with an argon balloon, TMP (56.5 mg, 0.4 mmol, 2.0 equiv) and extra dry MeOH (1.0 mL) were added into it. The mixture was stirred for 1 hour at room temperature to form a white solution. Then the tube was cooled to -30 ºC, **1m** (33.0 mg, 0.2 mmol, 1.0 equiv) and PhMe$_2$Si-Bpin (105 mg, 0.4 mmol, 2.0 equiv) were added in sequence *via* syringe. It was continued to stir for 4 hours at -30 ºC. The tube was opened quickly at room temperature, the final solution was filtered through celite using CH_2Cl_2 (30 mL) under air. The filtrate was concentrated under reduced pressure to afford the crude product, which was purified by flash column chromatography on silica gel (eluent: PE) to obtain the desired product (*S*)-**3m** (45.9 mg, 87%) as slightly yellow oil.

$[\alpha]_D^{20}$ -32.9º (c = 0.78, CH_2Cl_2)

The enantiomeric excess of (*S*)-**3m** was determined by chiral HPLC analysis on Chiralcel OJ column. Conditions: hexane/isopropanol = 100:0, flow rate = 0.2 mL/min, UV-Vis detection at λ = 210 nm, t_{R1} = 21.0 min (major), t_{R2} = 23.0 min (minor), 20 ºC, ee = 84%.

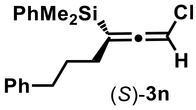

(*S*)-**3n**

(*S*)-**3n:** A dried screw-vial seal tube was charged with CuF_2 (2.0 mg, 0.02 mmol, 10 mol %) and ligand **L**$_5$ (19.5 mg, 0.04 mmol, 20 mol %) in glove box, then moved it out of the glove box. The tube was equipped with an argon balloon, TMP (56.5 mg, 0.4 mmol, 2.0 equiv) and extra dry MeOH (1.0 mL) were added into it. The mixture was stirred for 1 hour at room temperature to form a white solution. Then the tube was cooled to -30 ºC, **1n** (45.4 mg, 0.2 mmol, 1.0 equiv) and PhMe$_2$Si-Bpin (105 mg, 0.4 mmol, 2.0 equiv) were added in sequence via syringe. It was continued to stir for 4 hours at -30 ºC. The tube was opened quickly at room temperature, the final solution was filtered through celite using CH_2Cl_2 (30 mL) under air. The filtrate was concentrated under reduced pressure to afford the crude product, which was purified by flash column chromatography on silica gel (eluent: PE/Et_2O = 98:2) to obtain

the desired product (*S*)-**3n** (57.6 mg, 88%) as colorless oil.
$[\alpha]_D^{20}$ +6.5° (c = 1.49, CH_2Cl_2)
The enantiomeric excess of (*S*)-**3n** was determined by chiral HPLC analysis on Chiralcel OD-H column. Conditions: hexane/isopropanol = 100:0, flow rate = 0.3 mL/min, UV-Vis detection at λ = 210 nm, t_{R1} = 44.1 min (minor), t_{R2} = 46.5 min (major), 20 °C, ee = 80%.

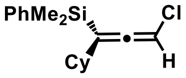

(*S*)-**3p**

(*S*)-**3p:** A dried screw-vial seal tube was charged with CuF_2 (2.0 mg, 0.02 mmol , 10 mol %) and ligand $\mathbf{L_5}$ (19.5 mg, 0.04 mmol, 20 mol %) in glove box, then moved it out of the glove box. The tube was equipped with an argon balloon, TMP (56.5 mg, 0.4 mmol, 2.0 equiv) and extra dry MeOH (1.0 mL) were added into it. The mixture was stirred for 1 hour at room temperature to form a white solution. Then the tube was cooled to -30 °C, and **1p** (38.2 mg, 0.2 mmol, 1.0 equiv) and $PhMe_2Si$-Bpin (105 mg, 0.4 mmol, 2.0 equiv) were added in sequence via syringe. It was continued to stir for 4 hours at -30 °C. The tube was opened quickly at room temperature, the final solution was filtered through celite using CH_2Cl_2 (30 mL) under air. The filtrate was concentrated under reduced pressure to afford the crude product, which was purified by flash column chromatography on silica gel (eluent: PE) to obtain the desired product (*S*)-**3p** (51.7 mg, 89%) as slightly yellow oil.
$[\alpha]_D^{20}$ +17.3° (c = 1.10, CH_2Cl_2)
The enantiomeric excess of (*S*)-**3p** was determined by chiral HPLC analysis on Chiralcel OD-H column. Conditions: hexane/isopropanol = 99:1, flow rate = 0.5 mL/min, UV-Vis detection at λ = 210 nm, t_{R1} = 6.5 min (minor), t_{R2} = 6.9 min (major), 20 °C, ee = 80%.

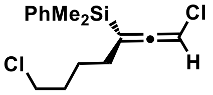

(*S*)-**3s**

(*S*)-**3s:** A dried screw-vial seal tube was charged with CuF_2 (2.0 mg, 0.02 mmol, 10 mol %) and ligand $\mathbf{L_5}$ (19.5 mg, 0.04 mmol, 20 mol %) in glove box, then moved it out of the glove box. The tube was equipped with an argon balloon, TMP (56.5 mg, 0.4 mmol, 2.0 equiv) and extra dry MeOH (1.0 mL) were added into it. The mixture was stirred for 1 hour at room temperature to form a white solution. Then the tube was cooled to -30 °C, **1s** (39.9 mg, 0.2 mmol, 1.0 equiv) and $PhMe_2Si$-Bpin (105 mg, 0.4 mmol, 2.0 equiv) were added in sequence via syringe. It was continued to stir for 4 hours at -30 °C. The tube was opened quickly at room temperature, the final solution was filtered through celite using CH_2Cl_2 (30 mL) under air. The filtrate was concentrated under reduced pressure to afford the crude product, which was purified by flash column chromatography on silica gel (eluent: PE/Et_2O = 98:2) to obtain the desired product (*S*)-**3s** (40.8 mg, 68%) as colorless oil.
$[\alpha]_D^{20}$ -11.2° (c = 1.35, CH_2Cl_2)
The enantiomeric excess of (*S*)-**3s** was determined by chiral HPLC analysis on Chiralcel OJ column. Conditions: hexane/isopropanol = 95:5, flow rate = 0.7 mL/min, UV-Vis detection at λ = 210 nm, t_{R1} = 6.2 min (minor), t_{R2} = 12.0 min (major), 20 °C, ee = 83%.

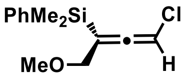

(*S*)-**3t**

(*S*)-**3t:** A dried screw-vial seal tube was charged with CuF_2 (2.0 mg, 0.02 mmol, 10 mol %) and ligand $\mathbf{L_5}$ (19.5 mg, 0.04 mmol, 20 mol %) in glove box, then moved it out of the glove box. The tube was equipped with an argon balloon, TMP (56.5 mg, 0.4 mmol, 2.0 equiv) and extra dry MeOH (1.0 mL) were added into it. The mixture was stirred for 1 hour at room temperature to form a white solution. Then the tube was cooled to -30 ^oC, **1t** (30.6 mg, 0.2 mmol, 1.0 equiv) and $PhMe_2Si$-Bpin (105 mg, 0.4 mmol, 2.0 equiv) were added in sequence *via* syringe. It was continued to stir for 4 hours at -30 ^oC. The tube was opened quickly at room temperature, the final solution was filtered through celite using CH_2Cl_2 (30 mL) under air. The filtrate was concentrated under reduced pressure to afford the crude product, which was purified by flash column chromatography on silica gel (eluent: PE/Et_2O = 99:1) to obtain the desired product (*S*)-**3t** (44.5 mg, 88%) as slightly yellow oil.

$[\alpha]_D^{20}$ -29.8^o (c = 0.99, CH_2Cl_2)

The enantiomeric excess of (*S*)-**3t** was determined by chiral HPLC analysis on Chiralcel OJ column. Conditions: hexane/isopropanol = 95:5, flow rate = 0.7 mL/min, UV-Vis detection at λ = 210 nm, t_{R1} = 7.3 min (minor), t_{R2} = 8.7 min (major), 20 ^oC, ee = 70%.

6 General Procedures for the One-pot Synthesis of Enantioenriched Disilyl-substituted Allenes.

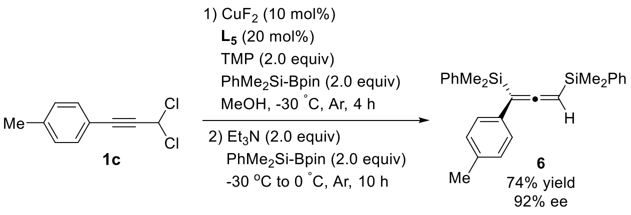

6: A dried screw-vial seal tube was charged with CuF_2 (2.0 mg, 0.02 mmol) and ligand $\mathbf{L_5}$ (19.5 mg, 0.04 mmol) in glove box, then moved it out of the glove box. The tube was equipped with an argon balloon, TMP (56.5 mg, 0.4 mmol) and dry MeOH (1.0 mL) were added into it. The mixture was stirred for 1 hour at room temperature to form an off-white solution. Then the tube was cooled to -30 ^oC, **1c** (39.8 mg, 0.2 mmol) and $PhMe_2Si$-Bpin (105 mg, 0.4 mmol) were added in sequence *via* syringe. It was continued to stir for 4 hours at -30 ^oC, the final yellow solution was added dry Et_3N (40.5 mg, 0.4 mmol). It was continued to stir for 20 minutes at -30 ^oC, $PhMe_2Si$-Bpin (105 mg, 0.4 mmol) was added dropwise *via* syringe. The system was warmed to 0 ^oC, and stirred 10 hours at this temperature. The tube was opened quickly at room temperature, the final solution was filtered through celite using CH_2Cl_2 (30 mL) under air. The filtrate was concentrated under reduced pressure to afford the crude product, which was purified by flash column chromatography on silica gel (eluent: PE) to obtain the desired product **6** (59 mg, 74 %) as yellow oil. **^{1}H NMR (400 MHz, $CDCl_3$):** δ 7.57 – 7.54 (m, 4H), 7.38 – 7.33 (m, 6H), 7.11 -7.09 (m, 2H), 7.02 – 7.00 (m, 2H), 5.09 (s, 1H), 2.28 (s, 1H), 0.44 (s, 3H), 0.43 (s, 6H), 0.40 (s, 3H). **^{13}C NMR (100 MHz, $CDCl_3$):** δ 213.67, 140.40, 140.22, 137.10, 135.88, 135.61, 135.43, 131.03, 130.96, 130.88, 129.66, 129.33, 91.07, 78.04, 22.85, 0.36, 0.26, 0.01, 0.00. **HRMS (EI):** m/z calculated for

$C_{26}H_{30}Si_2[M]^+$: 398.1881, found: 398.1894. **IR** (KBr): 2957, 1901, 1508, 1428, 1304, 1251, 1115, 914, 829, 730, 699, 656.

$[\alpha]_D^{20}$ +54.0° (c = 0.80, CH_2Cl_2)

The enantiomeric excess of **6** was determined by chiral HPLC analysis on Chiralcel OJ column. Conditions: hexane/isopropanol = 97:3, flow rate = 0.1 mL/min, UV-Vis detection at λ = 214 nm, t_{R1} = 33.7 min (major), t_{R2} = 47.1 min (minor), 20 °C, ee = 92%.

7 Further Transformations of Silyl-substituted Chloroallenes.

7.1 General Procedure for Sonogashira Coupling Reaction.

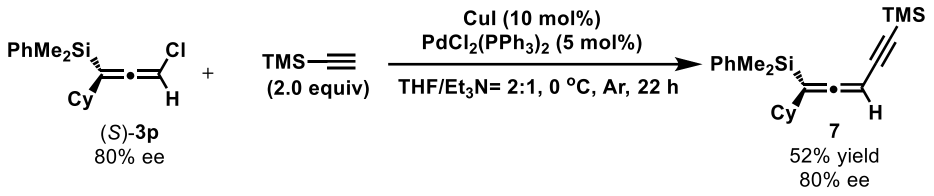

7: A dried screw-vial seal tube was charged with CuI (3.8 mg, 0.02 mmol, 10 mol %) and $PdCl_2(PPh_3)_2$ (7.0 mg, 0.01 mmol, 5 mol %) in glove box, then moved it out of the glove box. The tube was equipped with an argon balloon, (*S*)-**3p** (58.2mg, 0.2 mmol, 1.0 equiv), a dried and degassed mixture of THF (2.0 mL) and Et_3N (1.0 mL) were added into it. The atmosphere of the tube was displaced by argon for 20 minutes, then it was cooled to 0 °C for 10 minutes, trimethylsilylacetylene (39.3 mg, 0.4 mmol, 2.0 equiv) was introduced dropwise via syringe. The reaction mixture was stirred for 22 hours at 0 °C under dark environment. The tube was opened quickly at room temperature, the final black solution was filtered through celite using CH_2Cl_2 (30 mL) under air. The filtrate was concentrated under reduced pressure to afford the crude product, which was purified by flash column chromatography on silica gel (eluent: PE) to obtain the desired product **7** (36.7 mg, 52%) as brown oil. **^{1}H NMR (400 MHz, $CDCl_3$):** δ 7.56 – 7.53 (m, 2H), 7.37 – 7.33 (m, 3H), 5.14 (d, *J* = 1.9 Hz, 1H), 1.87 – 1.83 (m, 1H), 1.72 – 1.66 (m, 4H), 1.58 – 1.56 (m, 1H), 1.19 – 1.10 (m, 5H), 0.42 (s, 3H), 0.41 (s, 3H), 0.19. **^{13}C NMR (100 MHz, $CDCl_3$):** δ 212.52, 137.64, 133.71, 129.06, 127.62, 103.30, 99.38, 94.01, 71.50, 38.99, 33.63, 33.60, 26.35, 26.30, 25.81, 0.00, -2.46, -2.79. **HRMS (EI):** m/z calculated for $C_{22}H_{32}Si_2[M]^+$: 352.2037, found: 352.2036. **IR** (KBr): 3069, 2927, 2852, 2153, 1913, 1428, 1370, 1112, 1062, 999, 842, 733, 700.

$[\alpha]_D^{20}$ +2560.0° (c = 0.20, CH_2Cl_2)

The enantiomeric excess of **7** was determined by chiral HPLC analysis on Chiralcel OD-H&OD-H column. Conditions: hexane/isopropanol = 100:0, flow rate = 0.2 mL/min, UV-Vis detection at λ = 260 nm, t_{R1} = 48.3 min (major), t_{R2} = 50.1 min (minor), 20 °C, ee = 80%.

7.2 General Procedure for Kumada Reaction.[4]

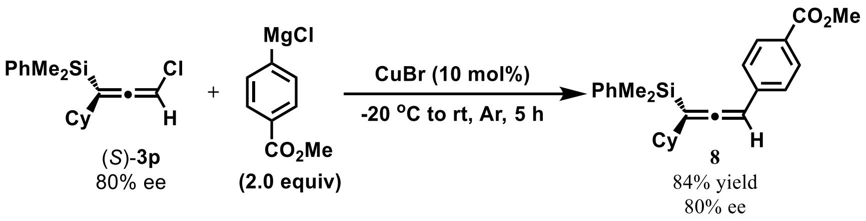

8: An oven-dried, argon-flushed Schlenk tube equipped with a magnetic stirring bar and a septum was charged with methyl 4-iodobenzoate (157.2 mg, 0.6 mmol) in THF (0.6 mL). *i*-PrMgCl·LiCl (0.49 mL, 0.64 mmol, 1.3 M in THF) was added at -20 °C and the mixture was stirred for 1 hour at this temperature. The CuBr (2.8 mg, 0.02 mmol, 10 mol%) and (*S*)-**3p** (58.2 mg, 0.2 mmol) were added quickly under argon atmosphere. The reaction mixture was stirred at room temperature (20 °C) until (*S*)-**3p** was consumed completely (TLC). The solution was diluted with DCM (10 mL), then poured into an ice-cooled saturated aqueous NH_4Cl solution (15 mL). After extraction with DCM (3 x 20 mL), the organic layers were dried (Na_2SO_4), filtered, and concentrated *in vacuo*. Product **8** was purified as yellow oil (65.7 mg, 84%) by flash column chromatography on silica gel (eluent: distilled PE/Et_2O = 100:0 ~ 96:4). **^{1}H NMR (400 MHz, $CDCl_3$):** δ 7.93 (d, *J* = 8.3 Hz, 2H), 7.60 – 7.50 (m, 2H), 7.38 – 7.31 (m, 3H), 7.27 (d, *J* = 8.3 Hz, 2H), 5.98 (s, 1H), 3.89 (s, 3H), 1.98 – 1.93 (m, 1H), 1.80 – 1.73 (m, 2H), 1.67 – 1.63 (m, 2H), 1.60 – 1.54 (m, 1H), 1.21 – 1.07 (m, 5H), 0.42 (s, 3H), 0.40 (s, 3H). **^{13}C NMR (100 MHz, $CDCl_3$):** δ 209.39, 169.46, 143.96, 140.18, 136.18, 132.39, 131.67, 130.23, 129.76, 127.82, 108.76, 93.40, 54.35, 42.03, 36.89, 36.52, 28.97, 28.36, 0.00, -0.03. **HRMS (EI):** m/z calculated for $C_{25}H_{30}O_2Si$ $[M]^+$: 390.2015, found: 390.2018. **IR** (KBr): 3069, 2925, 2851, 1911, 1720, 1605, 1434, 1276, 1173, 1111, 1017, 974, 817, 734, 699.
$[\alpha]_D^{20}$ +801.1° (c = 0.80, CH_2Cl_2)
The enantiomeric excess of **8** was determined by chiral HPLC analysis on Chiralcel OD-H column. Conditions: hexane/isopropanol = 98:2, flow rate = 0.6 mL/min, UV-Vis detection at λ = 260 nm, t_{R1} = 7.2 min (major), t_{R2} = 7.8 min (minor), 20 °C, ee = 80%.

7.3 General Procedure for Suzuki Coupling Reaction.

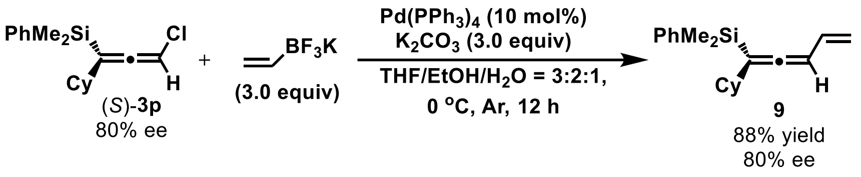

9: A dried argon-flushed Schlenk tube was charged with K_2CO_3 (83 mg, 0.6 mmol, 3.0 equiv), potassium vinyltrifluoroborate (107.2 mg, 0.6 mmol, 3.0 equiv) and $Pd(PPh_3)_4$ (23.1 mg, 0.02 mmol, 10 mol %) under Ar. Then a degassed mixture of THF (1.2 mL), EtOH (0.8 mL) and H_2O (0.4 mL) was added into it under Ar. The tube was cooled to 0 °C for 5 minutes, (*S*)-**3p** (58.2 mg, 0.2 mmol, 1.0 equiv) was introduced under Ar. The Schlenk tube was sealed and the reaction mixture was stirred at 0 °C for 12 hours under dark environment. The final black solution was quenched by water (5 mL) and the aqueous phase was extracted with CH_2Cl_2 (3 x 15 mL). The combined organic phases were dried over Na_2SO_4, concentrated under reduced pressure, and purified by flash column chromatography on silica gel (eluent: PE). Product **9** was obtained as yellow oil (49.7 mg, 88%). **^{1}H NMR (400 MHz, $CDCl_3$):** δ 7.54 – 7.52 (m,

2H), 7.36 – 7.33 (m, 3H), 6.21 – 6.12 (m, 1H), 5.67 (d, *J* = 10.3 Hz, 1H), 5.09 (d, *J* = 17.0 Hz, 1H), 4.82 (d, *J* = 10.1 Hz, 1H), 1.83 – 1.79 (m, 1H), 1.68 – 1.64 (m, 4H), 1.58 (s, 1H), 1.17 – 1.07 (m, 5H), 0.37 (s, 6H). **^{13}C NMR (100 MHz, $CDCl_3$):** δ 211.41, 140.75, 136.46, 136.17, 131.41, 130.09, 114.81, 105.28, 93.75, 41.33, 36.51, 36.33, 28.96, 28.94, 28.40, 0.00, -0.05. **HRMS (EI):** m/z calculated for $C_{19}H_{26}Si$ $[M]^+$: 282.1798, found: 282.1810. **IR** (KBr): 3069, 2925, 2852, 1913, 1612, 1448, 1428, 1248, 1112, 1028, 986, 888, 851, 818, 774, 731, 699, 650.

$[\alpha]_D^{20}$ -113.6° (c = 1.00, CH_2Cl_2)

The enantiomeric excess of **9** was determined by chiral HPLC analysis on Chiralcel OJ-H&OJ column. Conditions: hexane/isopropanol = 98:2, flow rate = 0.5 mL/min, UV-Vis detection at λ = 260 nm, t_{R1} = 16.1 min (major), t_{R2} = 16.8 min (minor), 20 °C, ee = 80%.

7.4 Copper-catalyzed Derivation Reactions of Chloroallenes.[5]

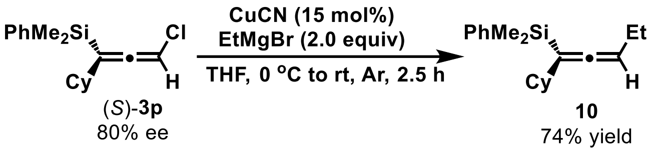

10: A dried screw-vial seal tube was charged with CuCN (1.4 mg, 0.015 mmol, 15 mol %) in glove box, then moved it out of the glove box. The tube was equipped with an argon balloon, (*S*)-**3p** (29.1 mg, 0.1 mmol, 1.0 equiv) and dry THF (1.0 mL) were added into it. It was cooled to 0 °C for further 5 minutes, the Ethylmagnesiumbromide (3.0 M in Et_2O, 67 μL, 0.2 mmol, 2.0 equiv) was added dropwise *via* syringe. The reaction mixture was allowed to warm back to room temperature and stir for 2.5 hours. The reaction mixture was quenched by saturated aqueous NH_4Cl solution and the aqueous phase was extracted with Et_2O (3 x 15 mL). The combined organic phases were dried over Na_2SO_4, concentrated under reduced pressure, and purified by flash column chromatography on silica gel (eluent: PE/Et_2O = 99.5:0.5). Product **10** was obtained as colorless oil (21.1 mg, 74%). **^{1}H NMR (400 MHz, $CDCl_3$):** δ 7.54 – 7.52 (m, 2H), 7.35 – 7.32 (m, 3H), 4.95 (td, J_1 = 6.0 Hz, J_2 = 1.6 Hz, 1H), 2.00 – 1.93 (m, 2H), 1.78 – 1.73 (m, 1H), 1.66 – 1.64 (m, 4H), 1.59 – 1.57 (m, 1H), 1.17 – 1.06 (m, 5H), 0.98 (t, *J* = 7.4 Hz, 3H), 0.34 (s, 6H). **^{13}C NMR (100 MHz, $CDCl_3$):** δ 208.61, 141.41, 136.14, 131.07, 129.87, 104.02, 91.04, 40.85, 36.46, 36.25, 28.99, 28.97, 28.43, 23.80, 16.10, 0.00, -0.01. **HRMS (EI):** m/z calculated for $C_{19}H_{28}Si$ $[M]^+$: 284.1955, found: 284.1953. **IR** (KBr): 2925, 2853, 1913, 1448, 1260, 1112, 1023, 888, 818, 776, 732, 700.

The product **10** can't be separated by HPLC, the subsequent $TiCl_4$-mediated addition of **10** to aldehyde afforded the homopropargylic alcohol. At beginning, we tried the 4-methylbenzaldehyde and isobutyraldehyde to react with the racemic allenylsilane **10** in the presence of different Lewis acids. Finally, it was found that only $TiCl_4$ could afford the desired products with excellent dr ratios albeit in low yields. Therefore, we consider that a highly reactive reactant possibly could favor a better yield of the desired product *via* a rapid nucleophilic addition process. Then the pentafluorobenzaldehyde was tested to react with the racemic allenylsilane **10** and enantioenriched allenylsilane **10** respectively. In both cases, the desired products were obtained in good yields and with excellent distereoselectivities. And

the chirality of the enantioenriched allenylsilane **10** was also well transformed during the synthesis of homoallylic alcohol product **11**. The detailed results are shown as below:

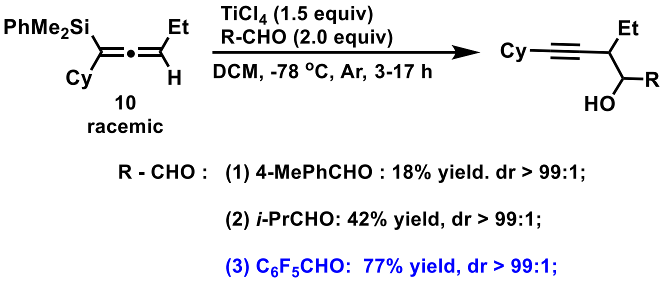

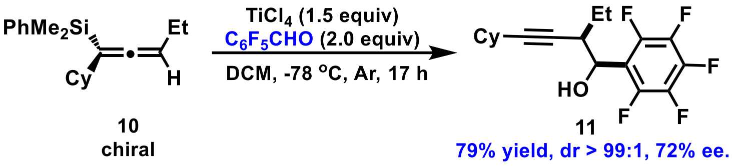

11: A dried argon-flushed Schlenk tube was charged with **10** (42.7 mg, 0.15 mmol), 2,3,4,5,6-pentafluorobenzaldehyde (58.8 mg, 0.3 mmol). It was added dry DCM (0.5 mL) and cooled to -78 °C for further 5 minutes, $TiCl_4$ solution (43.1 mg, dissolved in 0.2 mL dry DCM) was added dropwise at –78 ˚C, the mixture was stirred for 17 hours at this temperature. Then the solution was diluted with EA and quenched by 2N HCl aq., After stirring at room temperature for 10 minutes, the mixture was neutralized to pH = 7 with $NaHCO_3$ aq., the organic layer was separated. The aqueous layer was extracted with EA (3 x 20 mL), the combined organic phases were dried over Na_2SO_4, concentrated under reduced pressure, and purified by flash column chromatography on silica gel (eluent: PE/EA = 90:10). Product **11** was obtained as yellow oil (41.0 mg, 79%). **^{1}H NMR (400 MHz, $CDCl_3$):** δ 4.91 (dd, J_1 = 8.0 Hz, J_2 = 8.0 Hz, 1H), 2.85 (dt, J_1 = 8.0 Hz, J_2 = 1.2 Hz, 1H), 2.82 – 2.76 (m, 1H), 2.43 – 2.36 (m, 1H), 1.85 – 1.78 (m, 2H), 1.73 – 1.64 (m, 2H), 1.54 – 1.40 (m, 5H), 1.35 – 1.28 (m, 3H), 1.03 (t, J = 8.0 Hz, 3H). **^{19}F NMR (376 MHz, $CDCl_3$):** -142.38 – -142.46 (m, 1F), -154.88 – -154.99 (m, 2F), -161.97 – -162.11 (m, 2F). **^{13}C NMR (100 MHz, $CDCl_3$):** δ 146.27 – 146.04 (m), 143.84 – 143.56 (m), 142.12 – 141.66 (m), 138.94 – 138.58 (m), 136.45 – 136.08 (m), 115.50 – 115.18 (m), 90.56, 77.32, 69.06, 41.61, 32.84, 29.08, 25.82, 25.01, 24.86, 11.69. **HRMS (EI):** m/z calculated for $C_{18}H_{19}F_5O$ $[M]^+$: 346.1356, found: 346.1359. **IR** (KBr): 2955, 2911, 2875, 1892, 1428, 1249, 1112, 1089, 1015, 896, 839, 806, 730, 699.
$[\alpha]_D^{20}$ +3.0° (c = 1.23, CH_2Cl_2)
The enantiomeric excess of **11** was determined by chiral HPLC analysis on Chiralcel OJ column. Conditions: hexane/isopropanol = 99:1, flow rate = 0.5 mL/min, UV-Vis detection at λ = 220 nm, t_{R1} = 7.3 min (major), t_{R2} = 7.9 min (minor), 20 °C, ee = 72%.

8 Gram-scaled Synthesis of Racemic Silyl-substituted Allenes.

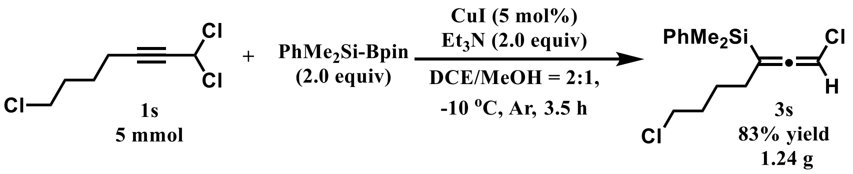

A dried screw-vial flask was charged with CuI (47.5 mg, 0.25 mmol) in glove box, then

moved it out of the glove box. The flask was equipped with an argon balloon, dry Et_3N (1.0 g, 10.0 mmol), dry DCE (25.0 mL) and dry MeOH (12.5 mL) were added into it. The mixture was stirred for 1 hour at room temperature to form a white suspension. Then the flask was cooled to -10 °C, **1s** (1.0 g, 5.0 mmol) and $PhMe_2Si$-Bpin (2.63 g, 10.0 mmol) were added in sequence via syringe. It was continued to stir for 3.5 hours at -10 °C. The flask was opened quickly at room temperature, the final solution was filtered through celite using CH_2Cl_2 (30 mL) under air. The filtrate was concentrated under reduced pressure to afford the crude product, which was purified by flash column chromatography on silica gel (eluent: PE) to obtain the desired product **3s** (1.24 g, 83%) as pale yellow oil.

9 Determination of the Absolute Configuration of Compound (*S*)-3p.

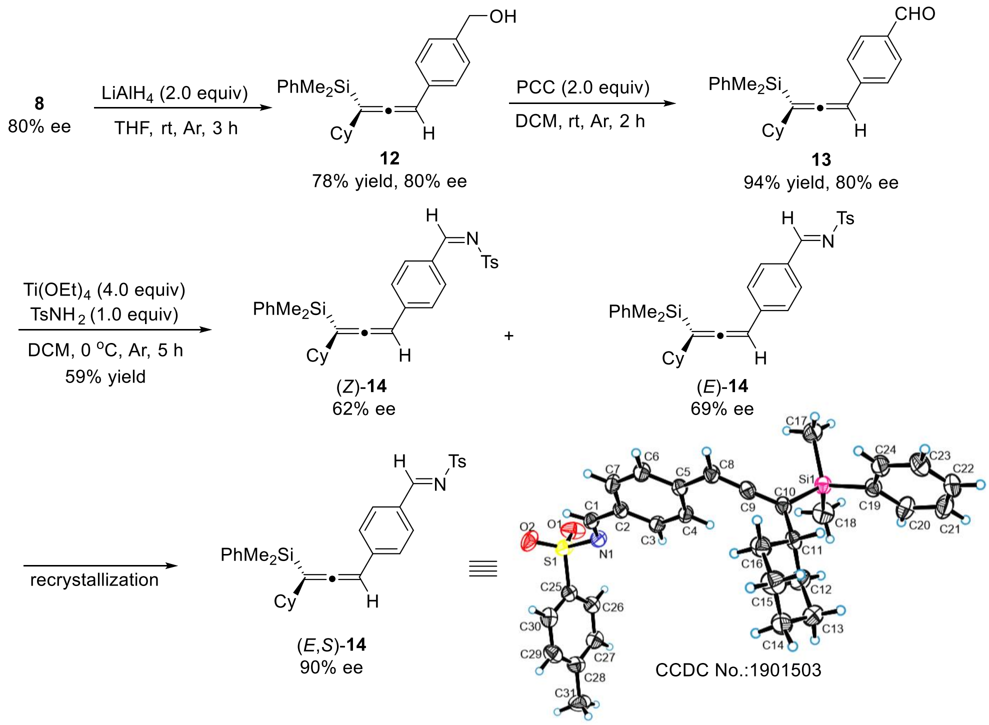

Step 1: In an oven-dried 35 mL screw-vial seal tube equipped with a stirring bar, **8** (78.1 mg, 0.2 mmol) was dissolved in 2 mL of dry THF under argon atmosphere. The solution was added $LiAlH_4$ (15.2 mg, 0.4 mmol) in four batches at 20 ℃. The final solution was continued to stir for 3 hours at room temperature (20 ℃). Then the reaction was quenched with water and excess amount of saturated potassium sodium tartrate was introduced, and the solution was stirred for 1 hour at room temperature (20 ℃). The final solution was extracted with ethyl acetate (4 x 15 mL), and the combined organic layers were washed with saturated brine (5 mL) and dried over anhydrous Na_2SO_4. The filtrate was concentrated under vacuum to afford the crude product which was purified through flash column chromatography (Eluent: PE/EA = 90:10) to furnish the related product **12** (56.6 mg, 78%, 80% ee) as yellow oil.

Step 2: A dried screw-vial seal tube equipped with an argon balloon, **12** (72.5 mg, 0.2 mmol) and dry DCM (1.0 mL) were introduced. PCC (86.2 mg, 0.4 mmol) was added into it, the mixture was continued to stir for 2 hours at 25 °C. Then the final solution was filtered through celite using CH_2Cl_2 (30 mL) and the solvent was evaporated under reduced pressure to afford the crude product, which was purified by flash column chromatography on silica gel (eluent:

PE/EA = 90:10) to obtain the desired product **13** (67.5 mg, 94%, 80% ee) as yellow oil.

Step 3:[6] A dried screw-vial seal tube equipped with an argon balloon was charged with 4-methylbenzenesulfonamide (59.9 mg, 0.35 mmol), **13** (126.3 mg, 0.35 mmol, dissolved in 1.8 mL dry DCM). It was cooled to 0 ^oC for further 10 minutes, $Ti(OEt)_4$ (319.4 mg, 1.4 mmol) was added dropwise via syringe. The tube was stirred for 5 hours at this temperature and monitored by TLC. The reaction mixture was quenched at 0 ^oC by addition of H_2O (6 mL), the turbid solution was filtered through celite, and the filter cake was washed with DCM (50 mL). The filtrate was concentrated under reduced pressure to afford the crude product, which was purified by flash column chromatography on silica gel (eluent: PE/EA = 90:10) to obtain the mixture of desired product (*Z*)**-14** (62% ee) and (*E*)**-14** (69% ee) as yellow solid (106.1 mg, 59%). After several recrystallization in mixed solvent (DCM/ PE = 1:1) at 5 ^oC, the single product (*E*)**-14** was obtained as white solid (90% ee). Finally, single-crystal X-ray diffraction analysis produced the molecular structure of (*E*)**-14** with (*S*)-configuration (CCDC No.:1901503). **^{1}H NMR (400 MHz, $CDCl_3$):** δ 8.96 (s, 1H), 7.88 (d, *J* = 7.9 Hz, 2H), 7.81 (d, *J* = 7.9 Hz, 2H), 7.53 (d, *J* = 6.6 Hz, 2H), 7.36 – 7.25 (m, 7H), 5.98 (s, 1H), 2.42 (s, 3H), 2.00 – 1.91 (m, 1H), 1.79 – 1.73 (m, 2H), 1.69 – 1.62 (m, 2H), 1.59 – 1.56 (m, 1H), 1.22 – 1.09 (m, 5H), 0.42 (s, 3H), 0.40 (s, 3H). **^{13}C NMR (100 MHz, $CDCl_3$):** δ 209.60, 172.01, 146.90, 146.76, 139.88, 138.07, 136.16, 134.36, 132.29, 132.18, 131.83, 130.39, 130.31, 128.52, 109.09, 93.63, 42.15, 36.95, 36.59, 28.96, 28.35, 24.10, 0.00, -0.03. **HRMS (ESI):** m/z Calcd. for $C_{31}H_{35}NO_2SSiNa$ $[M+Na]^+$: 536.2055, found: 536.2058. **IR** (KBr): 2955, 2910, 2875, 1892, 1428, 1249, 1112, 896, 839, 806, 730, 699.

$[\alpha]_D^{20}$ +80.6^o (c = 0.46, CH_2Cl_2)

The enantiomeric excess of (*E*, *S*)**-14** was determined by chiral HPLC analysis on Chiralcel Chiralpak ID column. Conditions: hexane/isopropanol = 90:10, flow rate = 0.6 mL/min, UV-Vis detection at λ = 210 nm, t_{R1} = 41.0 min (minor), t_{R2} = 42.8 min (major), 20 ^oC, ee = 90%.

10 References

(1) Li, H.-L.; Müller, D.; Guénée, L.; Alexakis, A. *Org. Lett.* **2012**, *14*, 5880–5883.

(2) Belot, S.; Vogt, K. A.; Besnard, C.; Krause, N.; Alexakis, A. *Angew. Chem. Int. Ed.* **2009**, *48*, 8923–8926.

(3) Hack, D.; Pankaj, C.; Deckers, K.; Mizutani, Y.; Raabe, G.; Enders, D. *Chem. Commun.* **2015**, *51*, 2266–2269.

(4) Schade, M. A.; Yamada, S.; Knochel, P. *Chem. Eur. J.* **2011**, *17*, 4232–4237.

(5) Ohmiya, H.; Ito, H.; Sawamura, M. *Org. Lett.* **2009**, *11*, 5618–5620.

(6) Davis, F. A.; Zhang, Y.; Andemichael, Y.; Fang, T.; Fanelli, D. L.; Zhang, H. *J. Org. Chem.* **1999**, *64*, 1403–1406.

11 ^{1}H NMR, ^{19}F NMR, ^{13}C NMR and HPLC Spectra of the Substrates and Products.

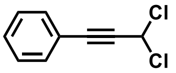

1a

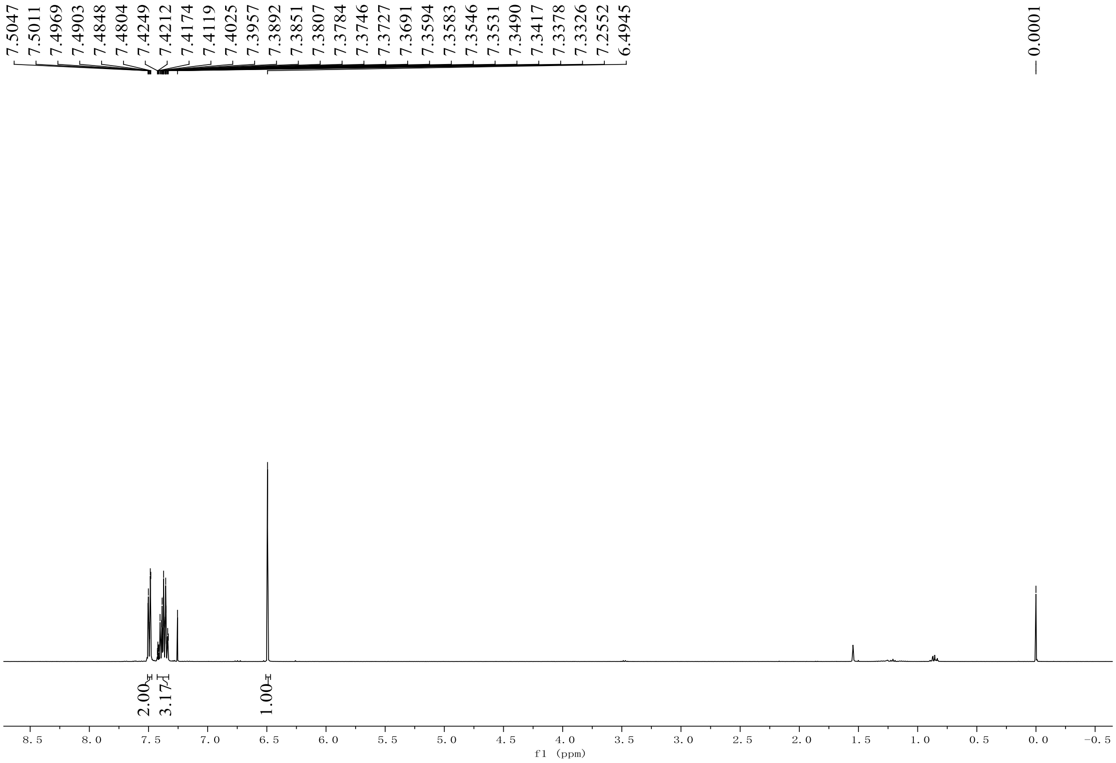

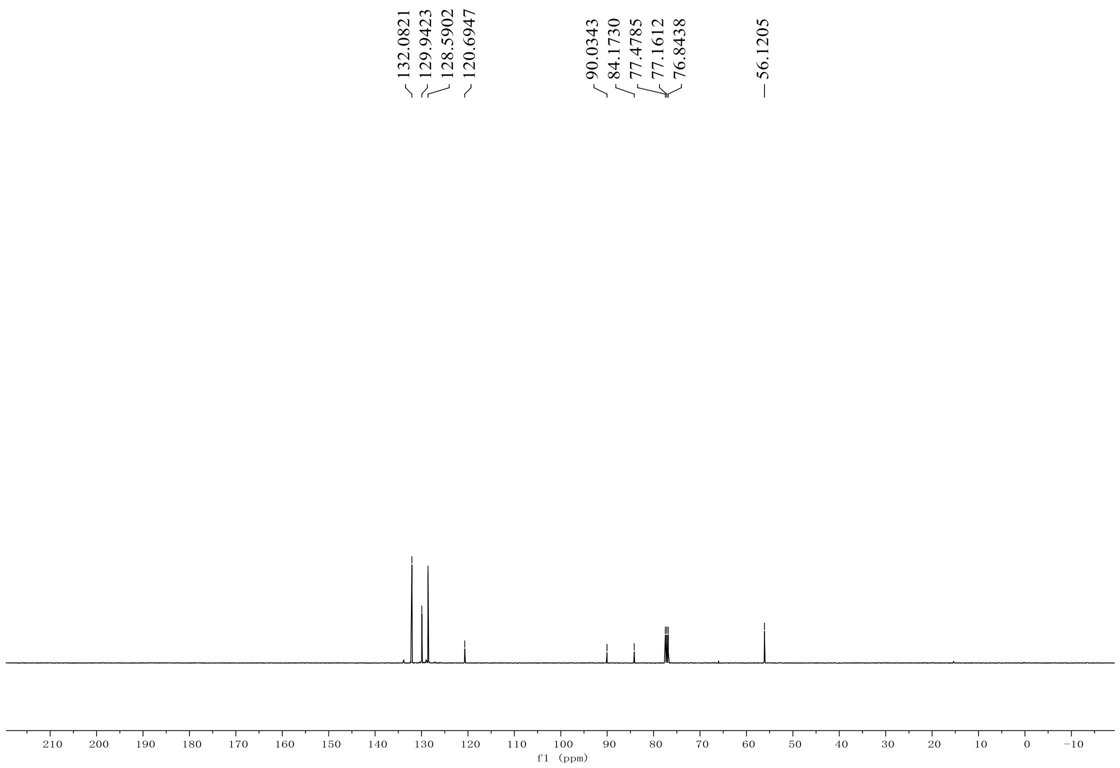
132.0821
129.9423
128.5902
120.6947
90.0343
84.1730
77.4785
77.1612
76.8438
56.1205
210 200 190 180 170 160 150 140 130 120 110 100 90 80 70 60 50 40 30 20 10 0 -10
f1 (ppm)

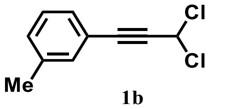
Cl
Cl
Me

1b

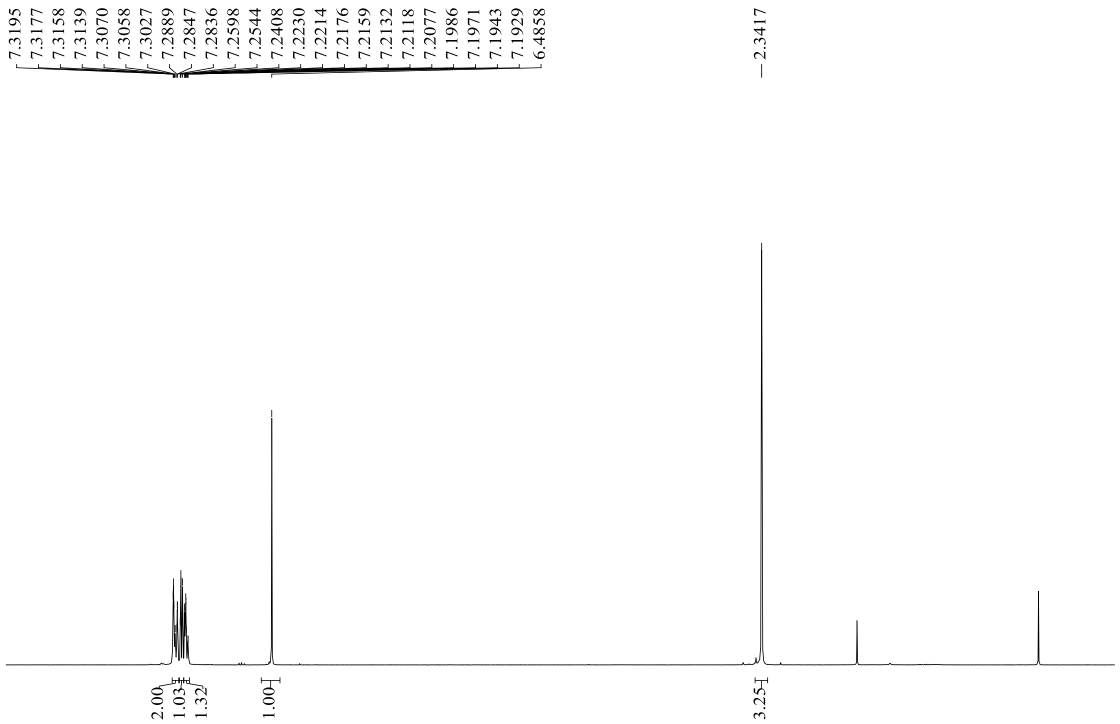
7.3195
7.3177
7.3158
7.3139
7.3070
7.3058
7.3027
7.2889
7.2847
7.2836
7.2598
7.2544
7.2408
7.2230
7.2214
7.2176
7.2159
7.2132
7.2118
7.2077
7.1986
7.1971
7.1943
7.1929
6.4858
2.3417
2.00
1.03
1.32
1.00
3.25
8.5 8.0 7.5 7.0 6.5 6.0 5.5 5.0 4.5 4.0 3.5 3.0 2.5 2.0 1.5 1.0 0.5 0.0 -0.5
f1 (ppm)

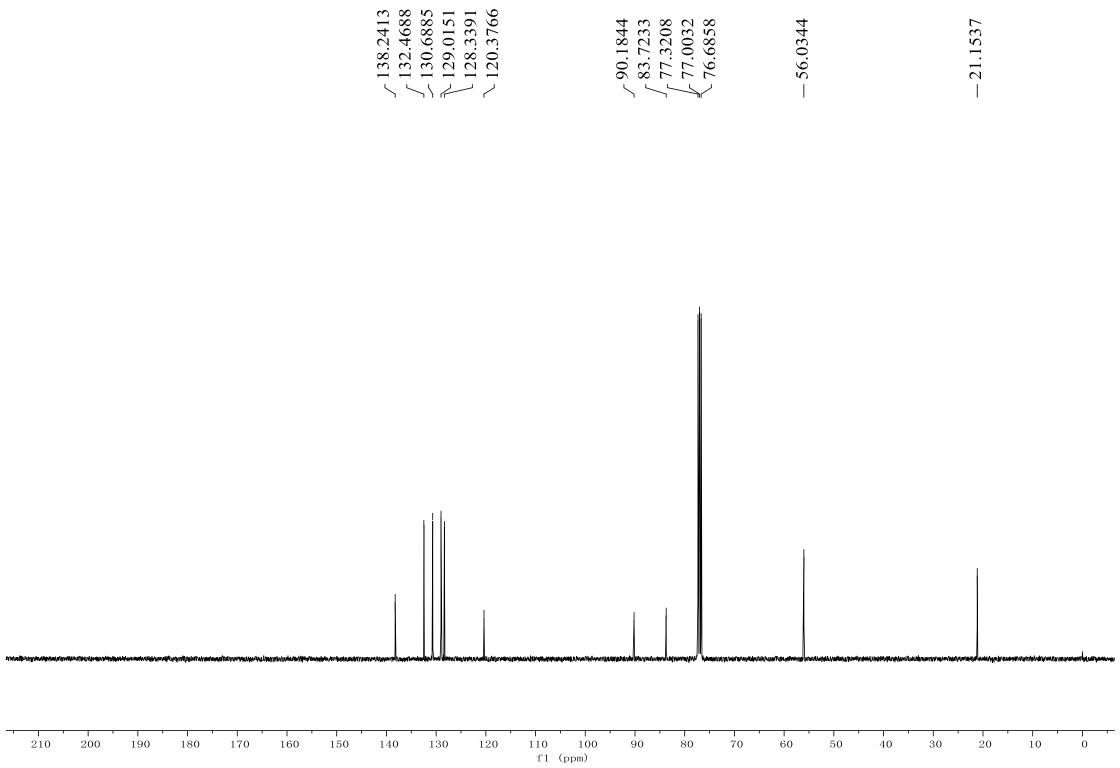
138.2413
132.4688
130.6885
129.0151
128.3391
120.3766
90.1844
83.7233
77.3208
77.0032
76.6858
56.0344
21.1537
210 200 190 180 170 160 150 140 130 120 110 100 90 80 70 60 50 40 30 20 10 0
f1 (ppm)

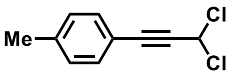
Me
Cl
Cl

1c

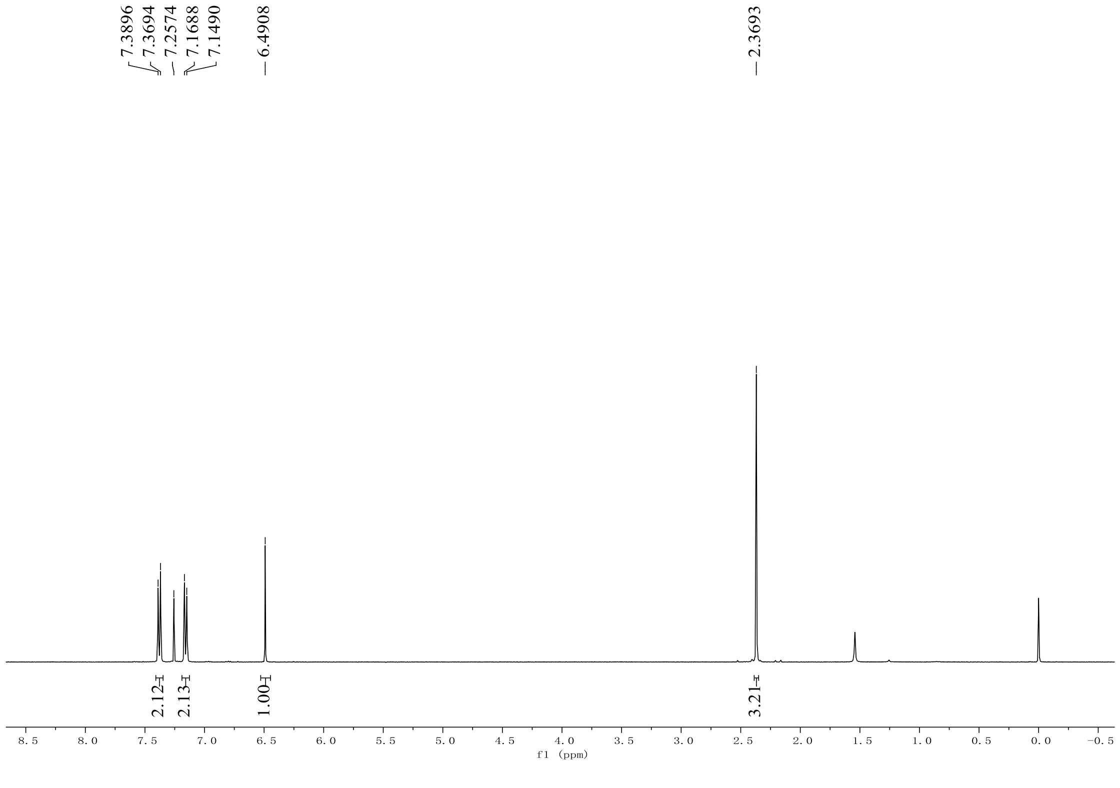
7.3896
7.3694
7.2574
7.1688
7.1490
6.4908
2.3693
2.12
2.13
1.00
3.21
8.5 8.0 7.5 7.0 6.5 6.0 5.5 5.0 4.5 4.0 3.5 3.0 2.5 2.0 1.5 1.0 0.5 0.0 -0.5
f1 (ppm)

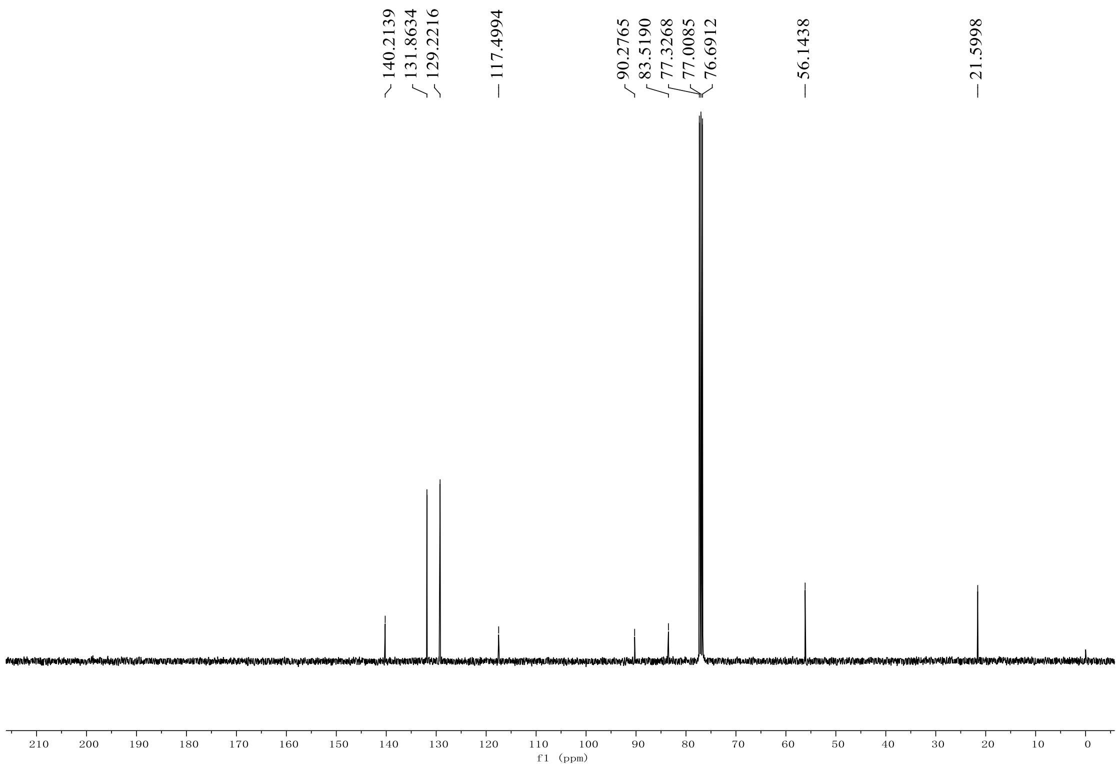
140.2139
131.8634
129.2216
117.4994
90.2765
83.5190
77.3268
77.0085
76.6912
56.1438
21.5998
210 200 190 180 170 160 150 140 130 120 110 100 90 80 70 60 50 40 30 20 10 0
f1 (ppm)

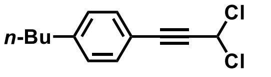
n-Bu
Cl
Cl

1d

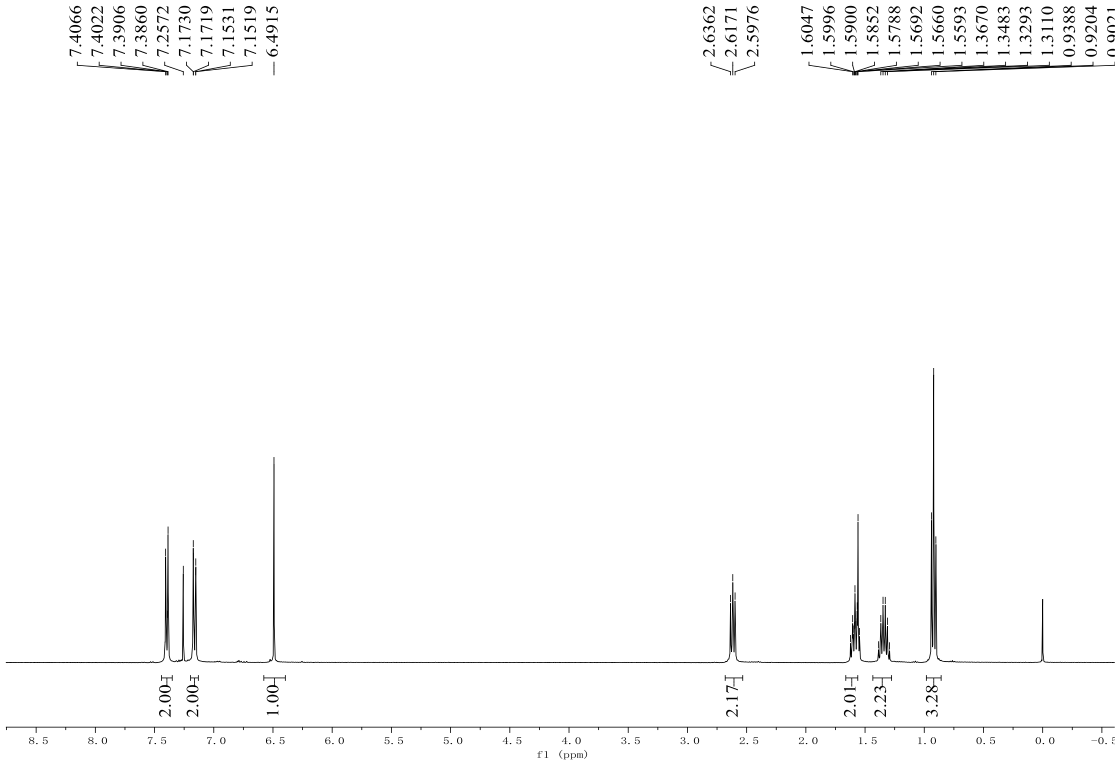
7.4066
7.4022
7.3906
7.3860
7.2572
7.1730
7.1719
7.1531
7.1519
6.4915
2.6362
2.6171
2.5976
1.6047
1.5996
1.5900
1.5852
1.5788
1.5692
1.5660
1.5593
1.3670
1.3483
1.3293
1.3110
0.9388
0.9204
2.00
2.00
1.00
2.17
2.01
2.23
3.28
8.5 8.0 7.5 7.0 6.5 6.0 5.5 5.0 4.5 4.0 3.5 3.0 2.5 2.0 1.5 1.0 0.5 0.0
f1 (ppm)

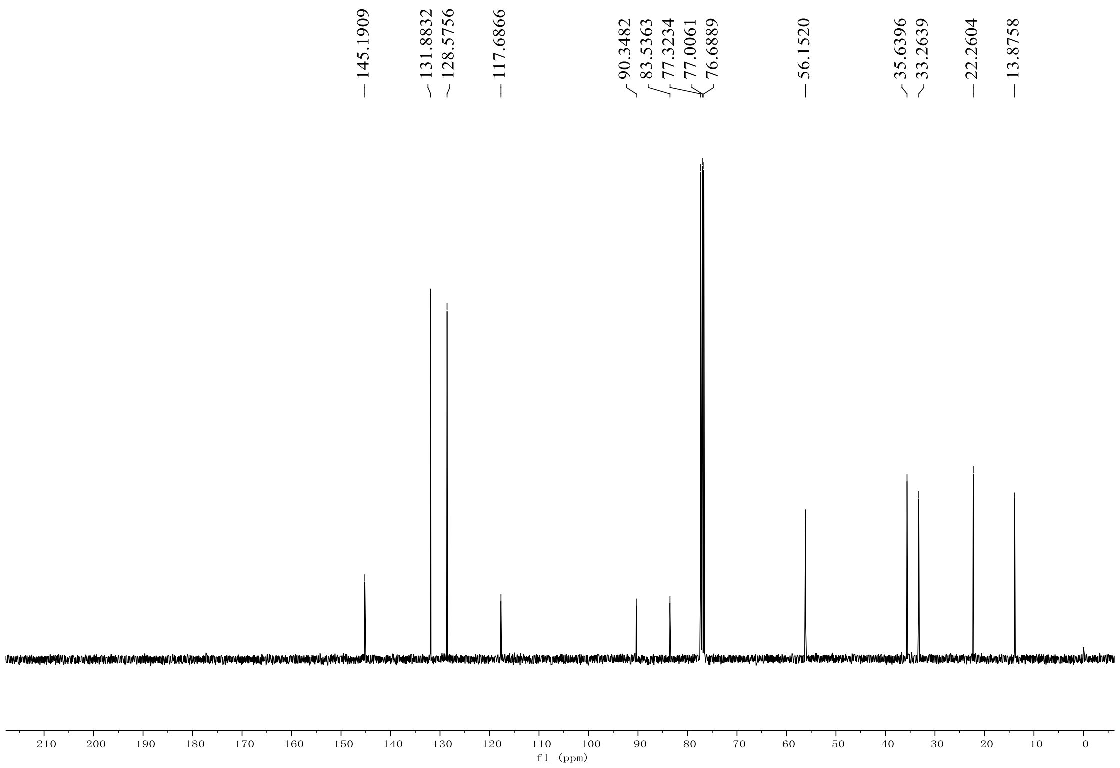
145.1909
131.8832
128.5756
117.6866
90.3482
83.5363
77.3234
77.0061
76.6889
56.1520
35.6396
33.2639
22.2604
13.8758
f1 (ppm)

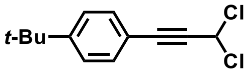
t-Bu
Cl
Cl

1e

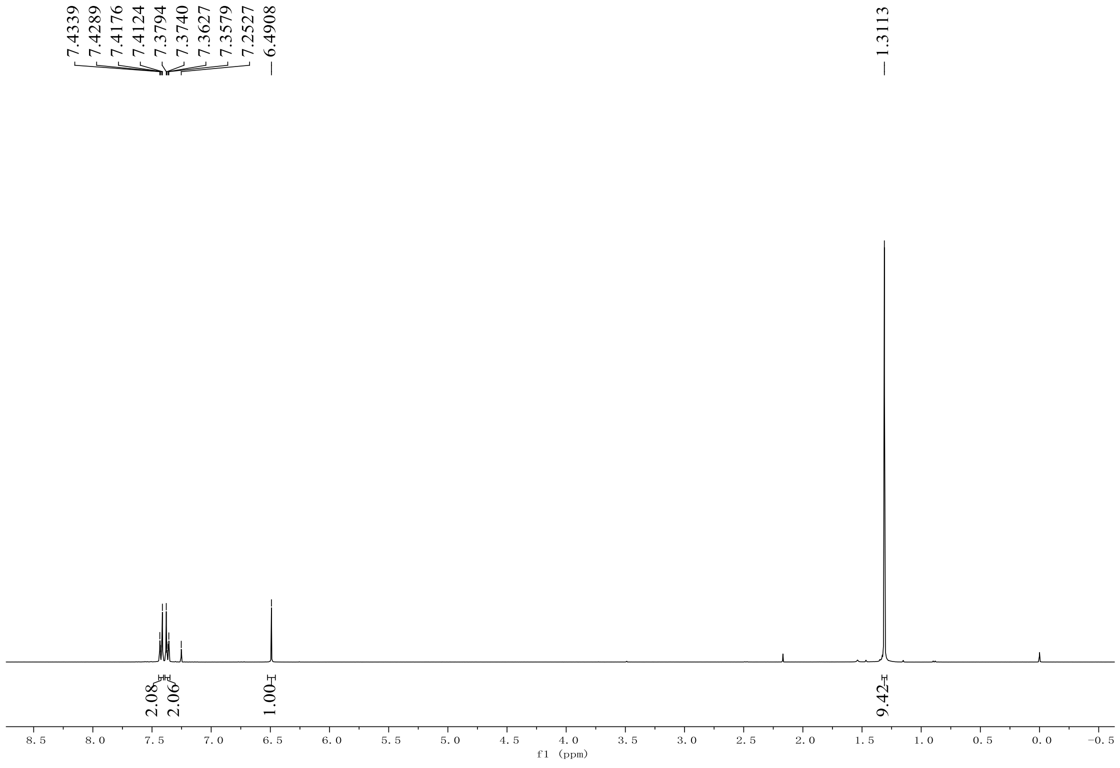
7.4339
7.4289
7.4176
7.4124
7.3794
7.3740
7.3627
7.3579
7.2527
6.4908
1.3113
2.08
2.06
1.00
9.42
f1 (ppm)

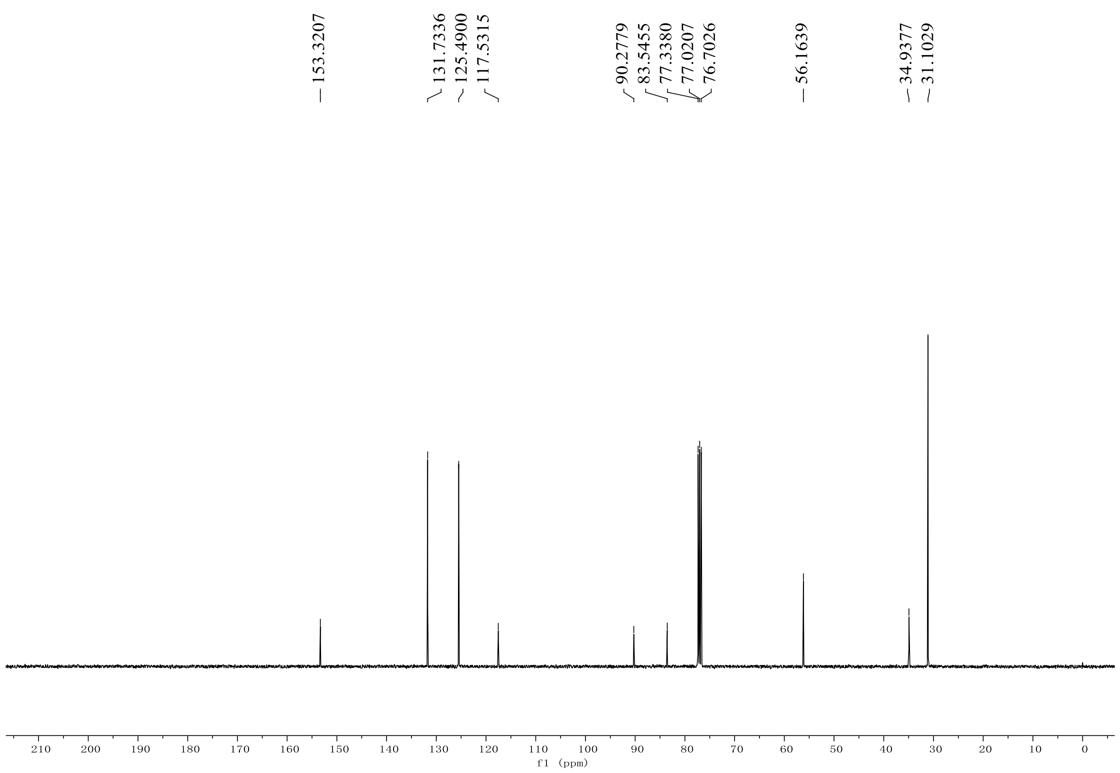
153.3207
131.7336
125.4900
117.5315
90.2779
83.5455
77.3380
77.0207
76.7026
56.1639
34.9377
31.1029
f1 (ppm)

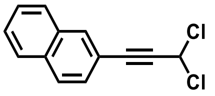
Cl
Cl

1f

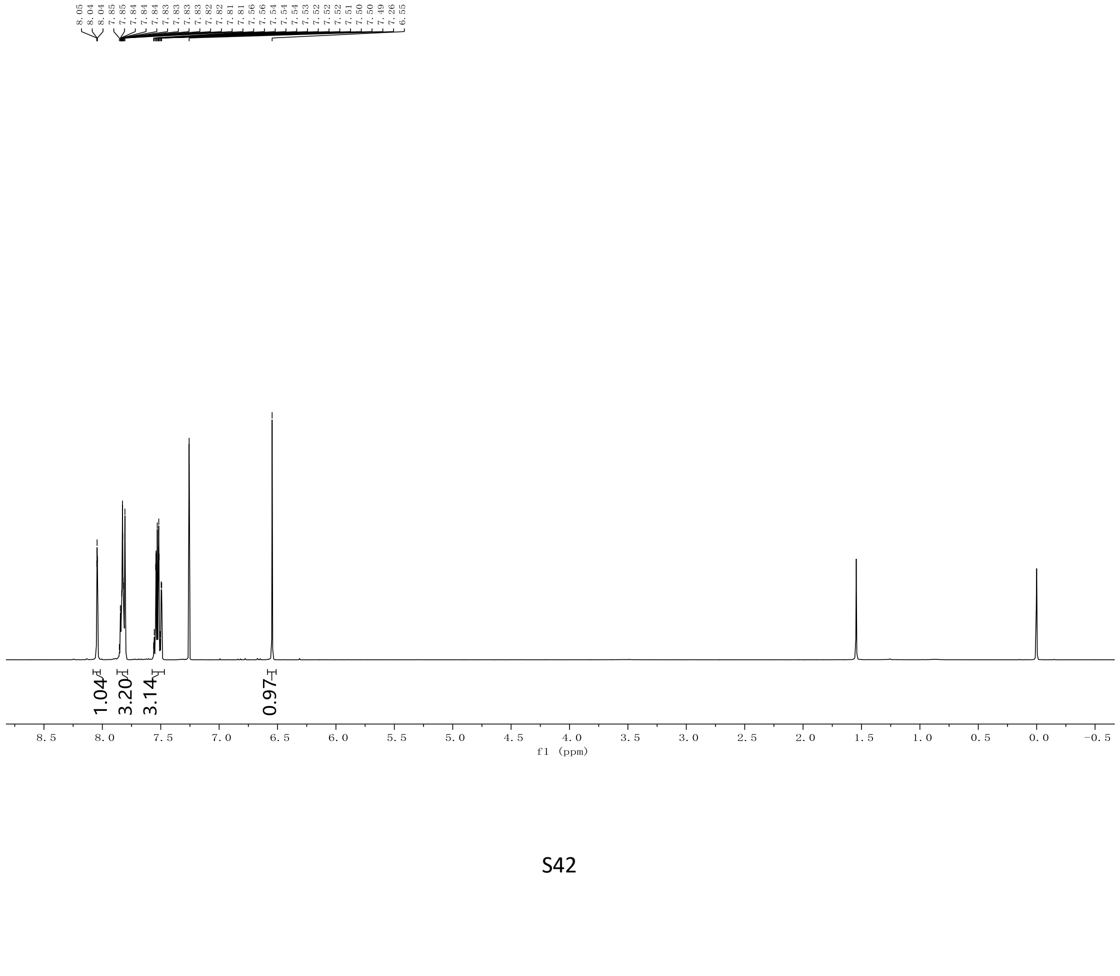
1.04
3.20
3.14
0.97
f1 (ppm)

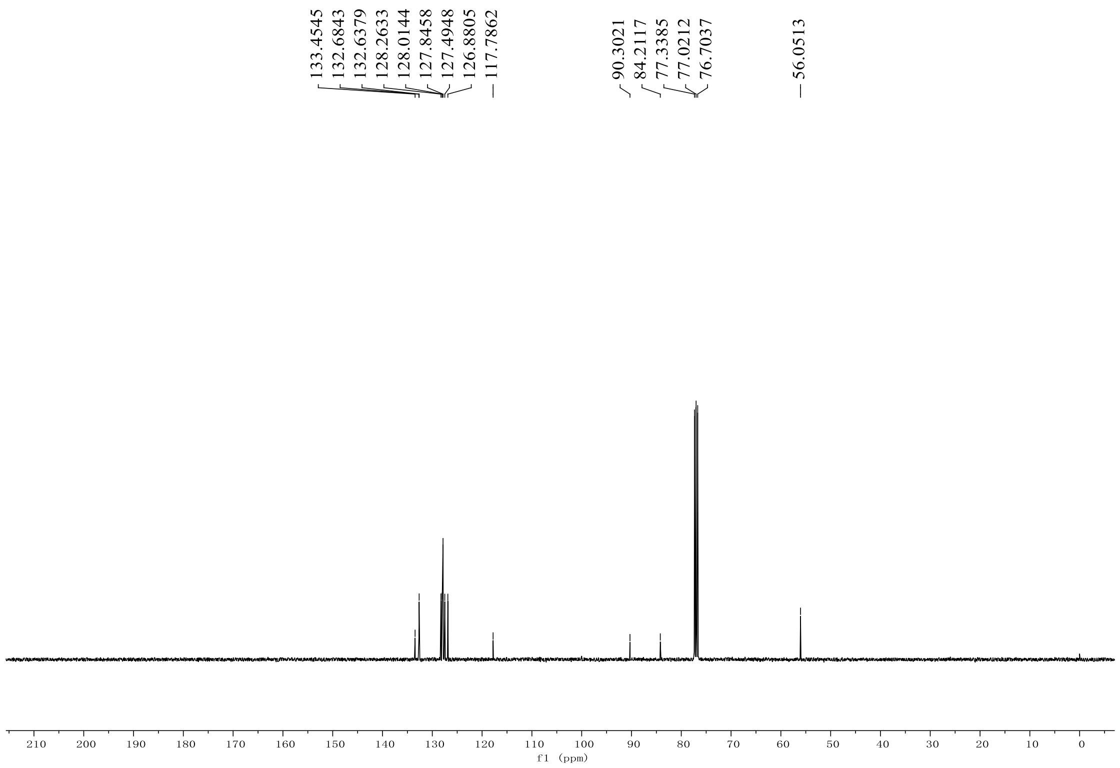
133.4545
132.6843
132.6379
128.2633
128.0144
127.8458
127.4948
126.8805
117.7862
90.3021
84.2117
77.3385
77.0212
76.7037
56.0513
f1 (ppm)

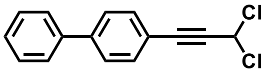
Cl
Cl

1g

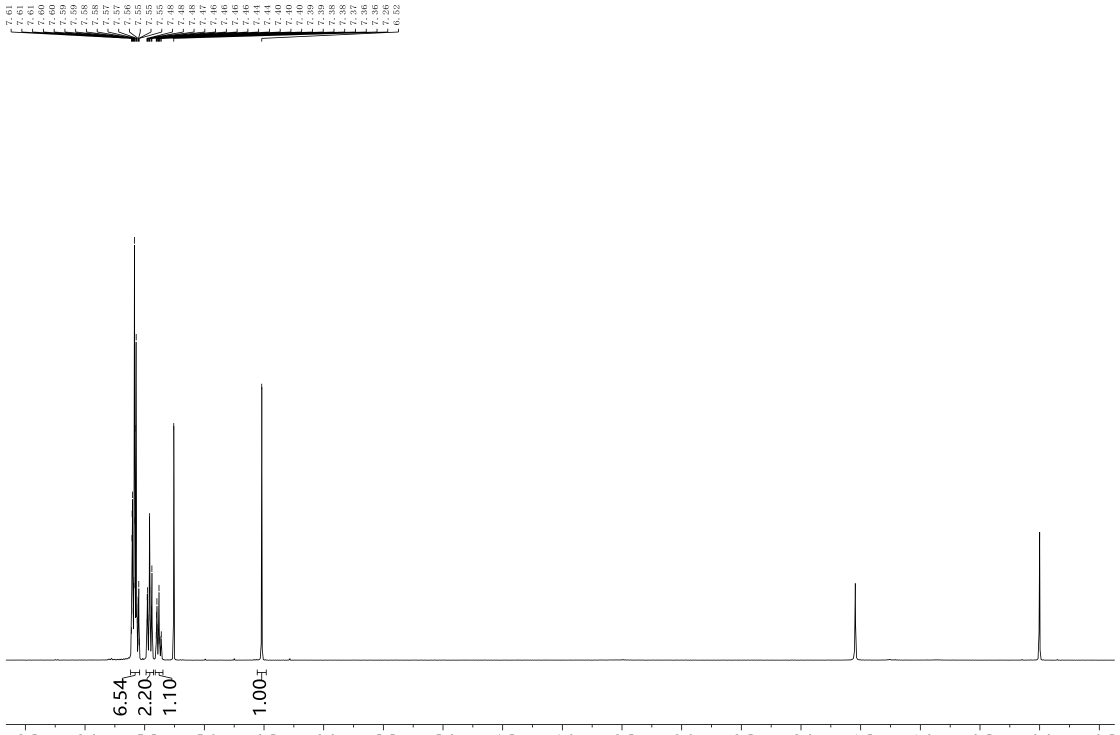
6.54
2.20
1.10
1.00
f1 (ppm)

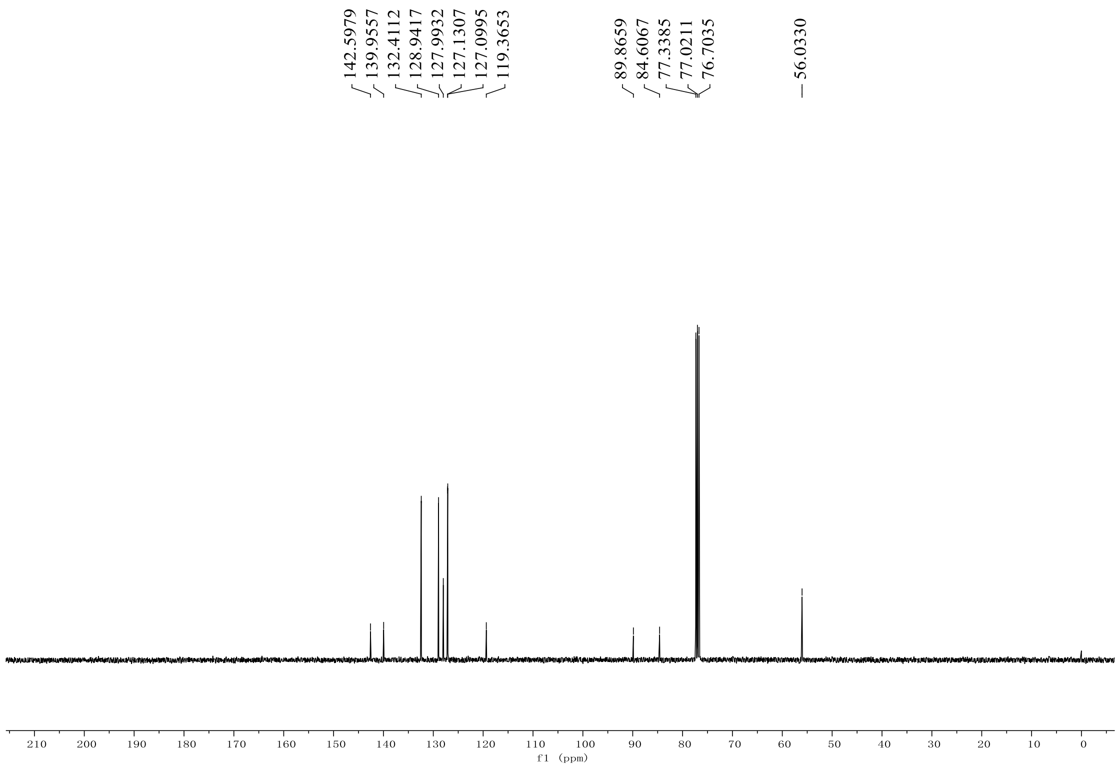
142.5979
139.9557
132.4112
128.9417
127.9932
127.1307
127.0995
119.3653
89.8659
84.6067
77.3385
77.0211
76.7035
56.0330
f1 (ppm)

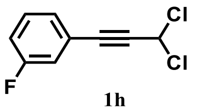
Cl
Cl
F

1h

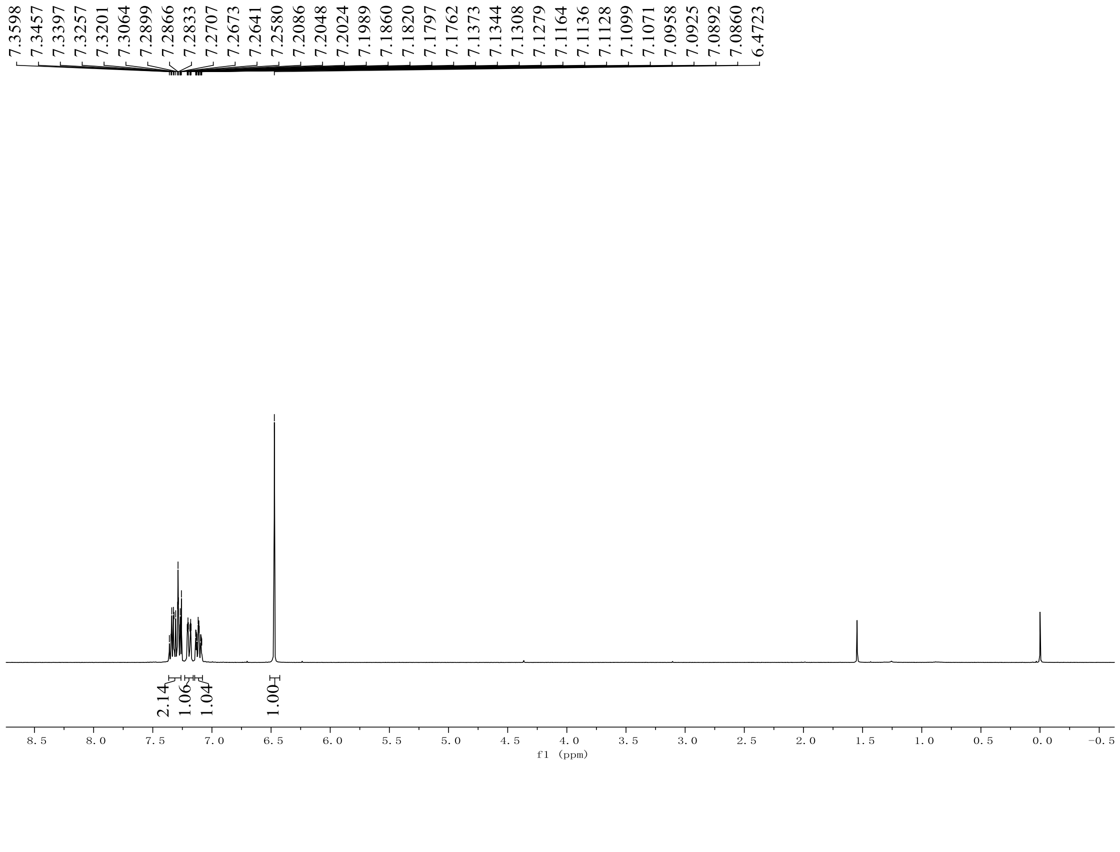
7.3598
7.3457
7.3397
7.3257
7.3201
7.3064
7.2899
7.2866
7.2833
7.2707
7.2673
7.2641
7.2580
7.2086
7.2048
7.2024
7.1989
7.1860
7.1820
7.1797
7.1762
7.1373
7.1344
7.1308
7.1279
7.1164
7.1136
7.1128
7.1099
7.1071
7.0958
7.0925
7.0892
7.0860
6.4723
2.14
1.06
1.04
1.00
f1 (ppm)

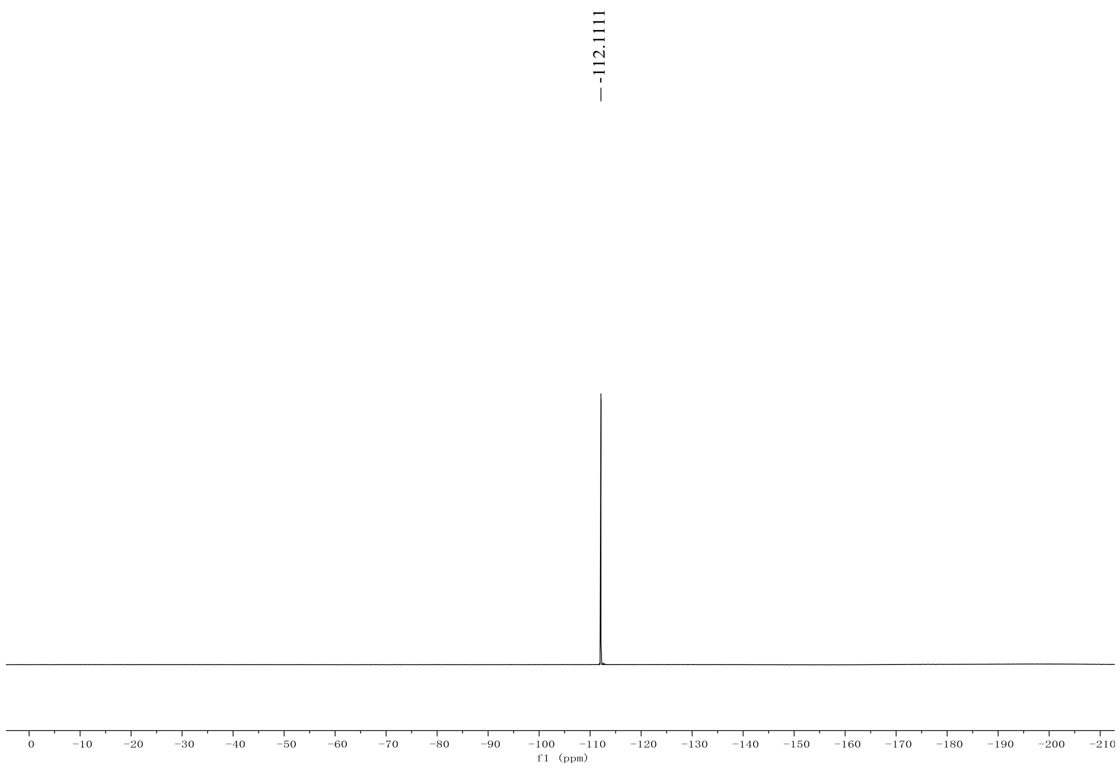
-112.1111
0 −10 −20 −30 −40 −50 −60 −70 −80 −90 −100 −110 −120 −130 −140 −150 −160 −170 −180 −190 −200 −210
f1 (ppm)

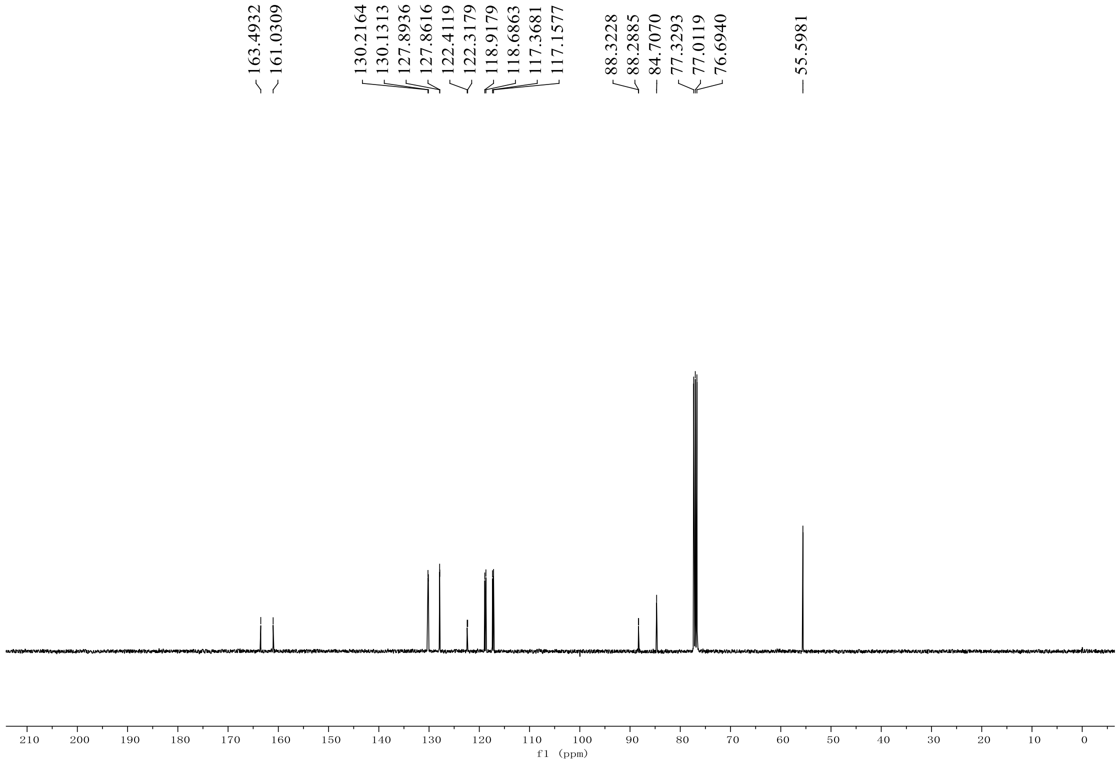
163.4932
161.0309
130.2164
130.1313
127.8936
127.8616
122.4119
122.3179
118.9179
118.6863
117.3681
117.1577
88.3228
88.2885
84.7070
77.3293
77.0119
76.6940
55.5981
210 200 190 180 170 160 150 140 130 120 110 100 90 80 70 60 50 40 30 20 10 0
f1 (ppm)

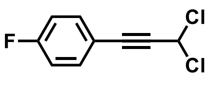
F
Cl
Cl

1i

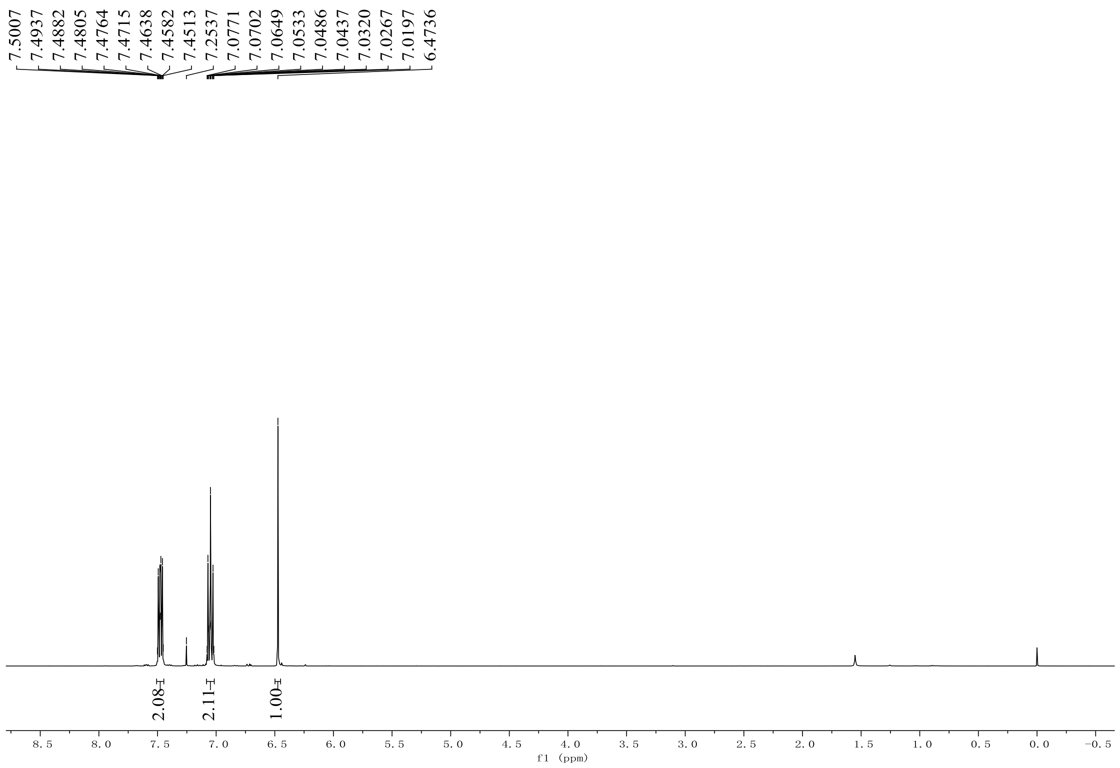
7.5007
7.4937
7.4882
7.4805
7.4764
7.4715
7.4638
7.4582
7.4513
7.2537
7.0771
7.0702
7.0649
7.0533
7.0486
7.0437
7.0320
7.0267
7.0197
6.4736
2.08
2.11
1.00
8.5 8.0 7.5 7.0 6.5 6.0 5.5 5.0 4.5 4.0 3.5 3.0 2.5 2.0 1.5 1.0 0.5 0.0 -0.5
f1 (ppm)

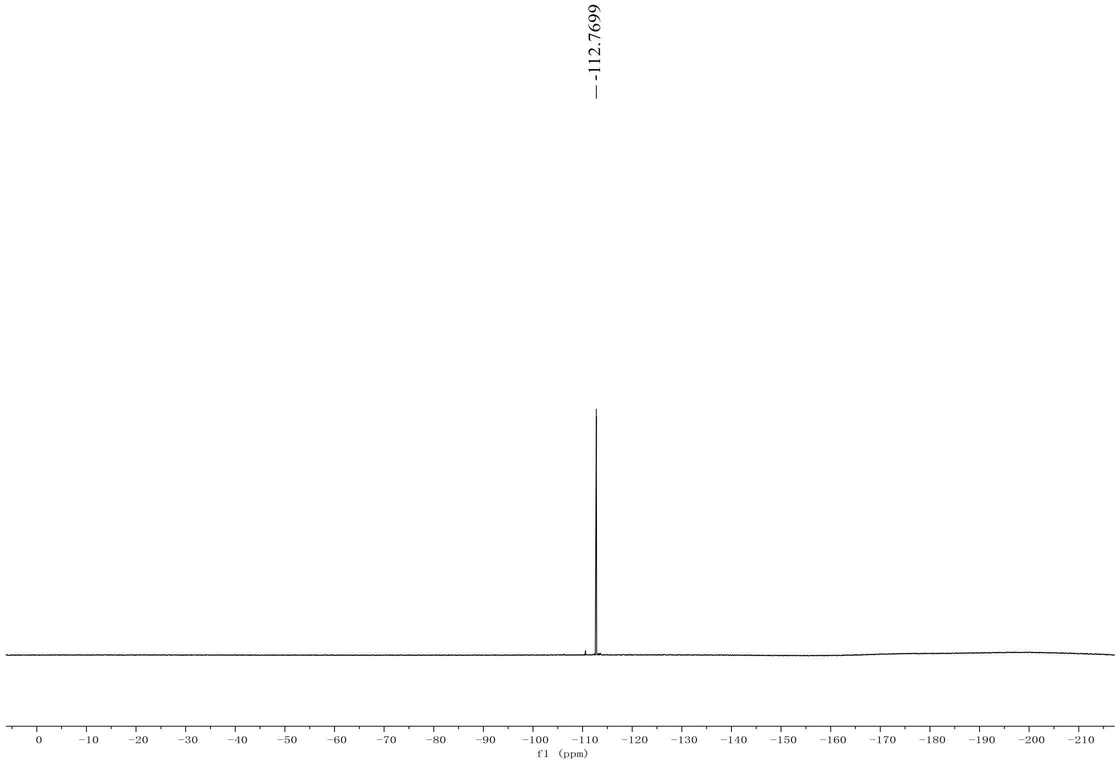
-112.7699
0 -10 -20 -30 -40 -50 -60 -70 -80 -90 -100 -110 -120 -130 -140 -150 -160 -170 -180 -190 -200 -210
f1 (ppm)

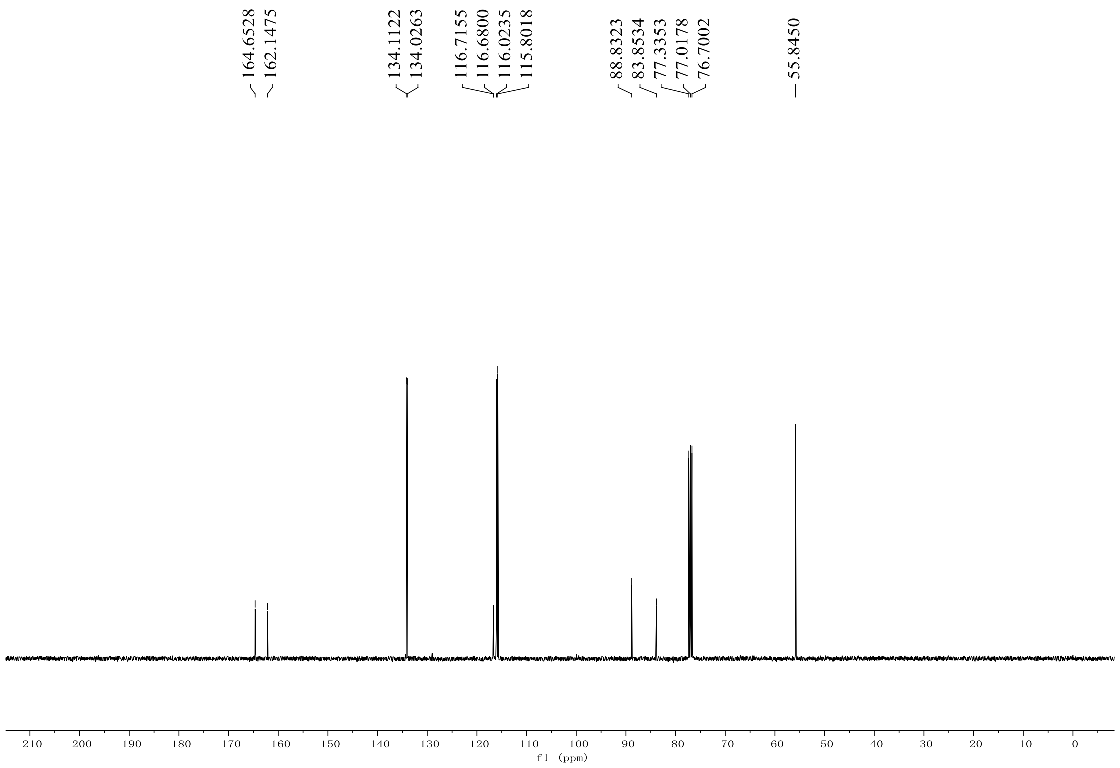
164.6528
162.1475
134.1122
134.0263
116.7155
116.6800
116.0235
115.8018
88.8323
83.8534
77.3353
77.0178
76.7002
55.8450
210 200 190 180 170 160 150 140 130 120 110 100 90 80 70 60 50 40 30 20 10 0
f1 (ppm)

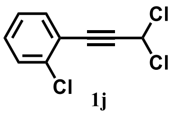
Cl
Cl
Cl
1j

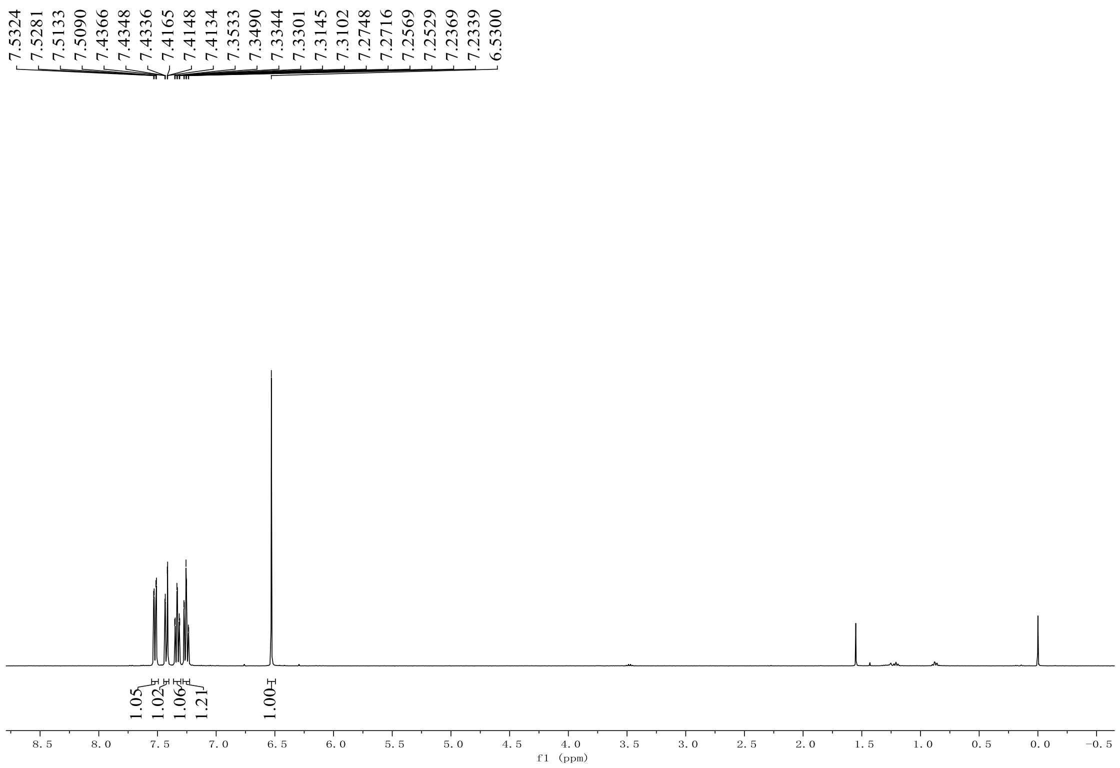
7.5324
7.5281
7.5133
7.5090
7.4366
7.4348
7.4336
7.4165
7.4148
7.4134
7.3533
7.3490
7.3344
7.3301
7.3145
7.3102
7.2748
7.2716
7.2569
7.2529
7.2369
7.2339
6.5300
1.05
1.02
1.06
1.21
1.00
8.5
8.0
7.5
7.0
6.5
6.0
5.5
5.0
4.5
4.0
3.5
3.0
2.5
2.0
1.5
1.0
0.5
0.0
-0.5
f1 (ppm)

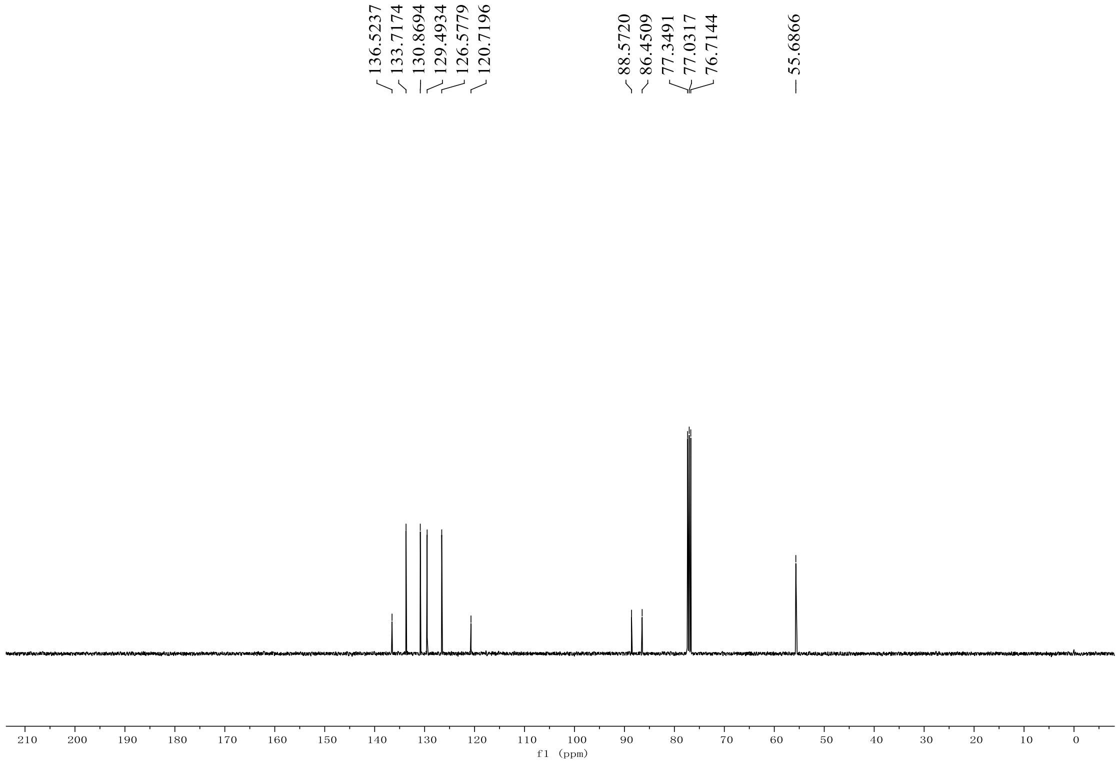
136.5237
133.7174
130.8694
129.4934
126.5779
120.7196
88.5720
86.4509
77.3491
77.0317
76.7144
55.6866
210
200
190
180
170
160
150
140
130
120
110
100
90
80
70
60
50
40
30
20
10
0
f1 (ppm)

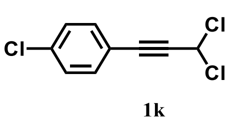
Cl
Cl
Cl

1k

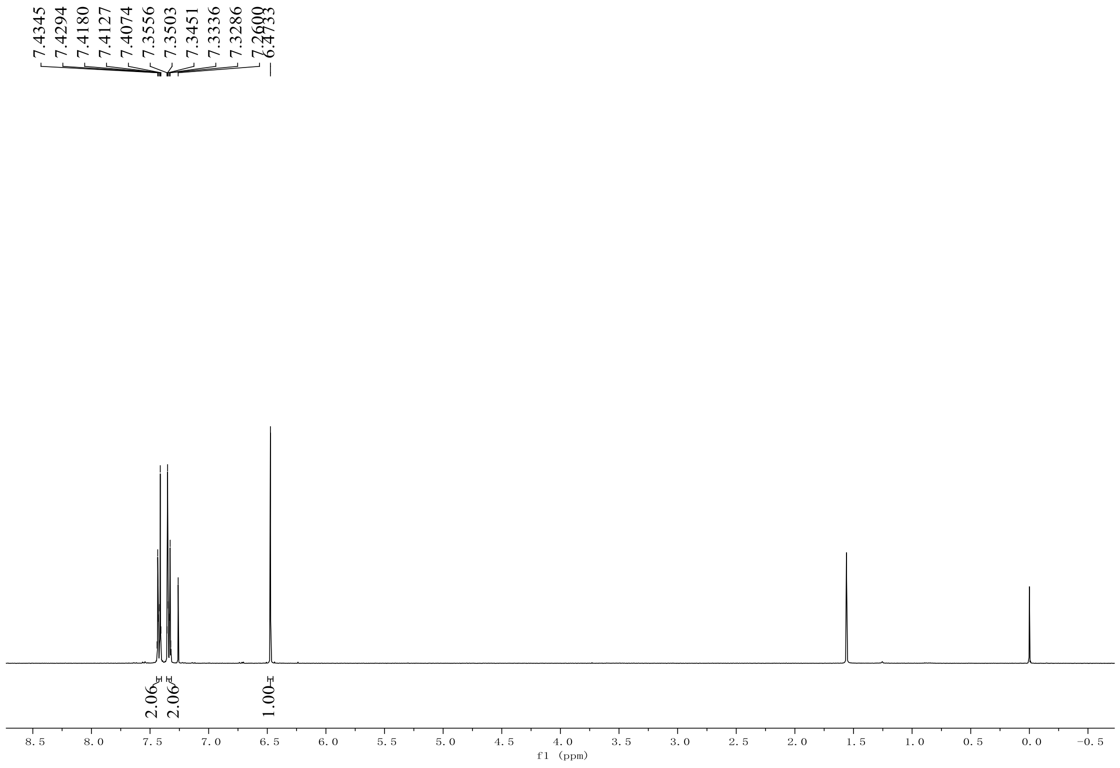
7.4345
7.4294
7.4180
7.4127
7.4074
7.3556
7.3503
7.3451
7.3336
7.3286
7.2600
6.4733
2.06
2.06
1.00
8.5 8.0 7.5 7.0 6.5 6.0 5.5 5.0 4.5 4.0 3.5 3.0 2.5 2.0 1.5 1.0 0.5 0.0 -0.5
f1 (ppm)

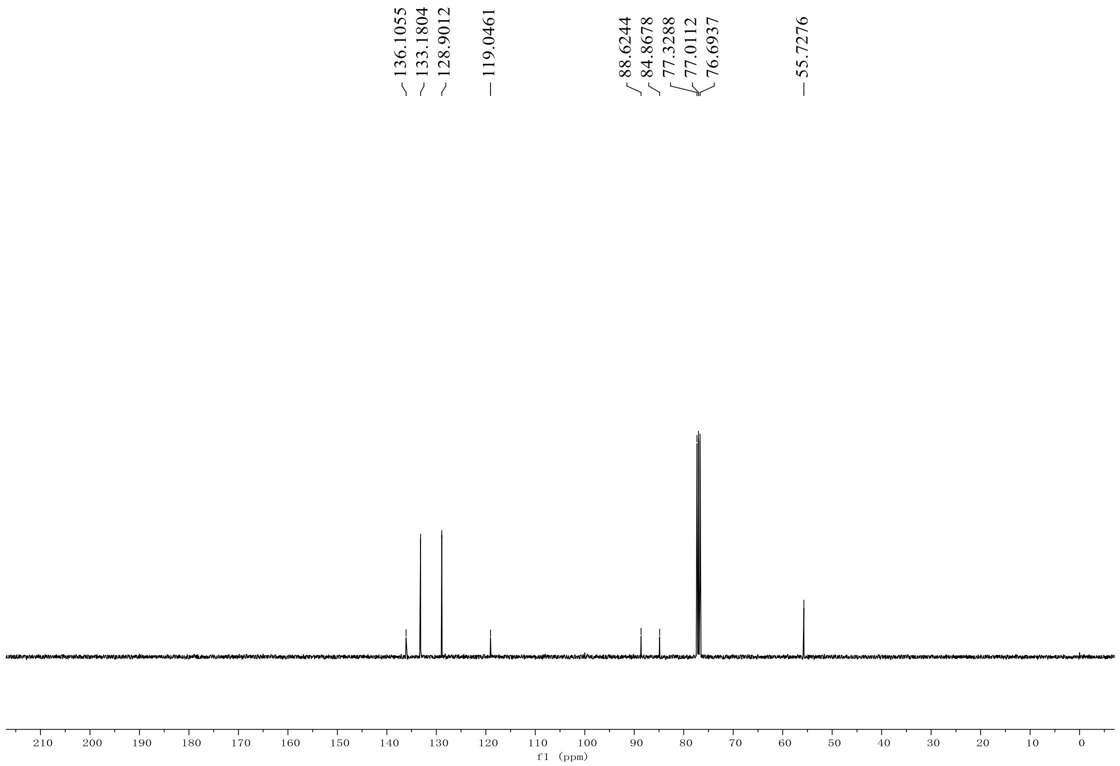
136.1055
133.1804
128.9012
119.0461
88.6244
84.8678
77.3288
77.0112
76.6937
55.7276
210 200 190 180 170 160 150 140 130 120 110 100 90 80 70 60 50 40 30 20 10 0
f1 (ppm)

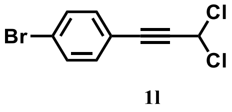
Br
Cl
Cl

1l

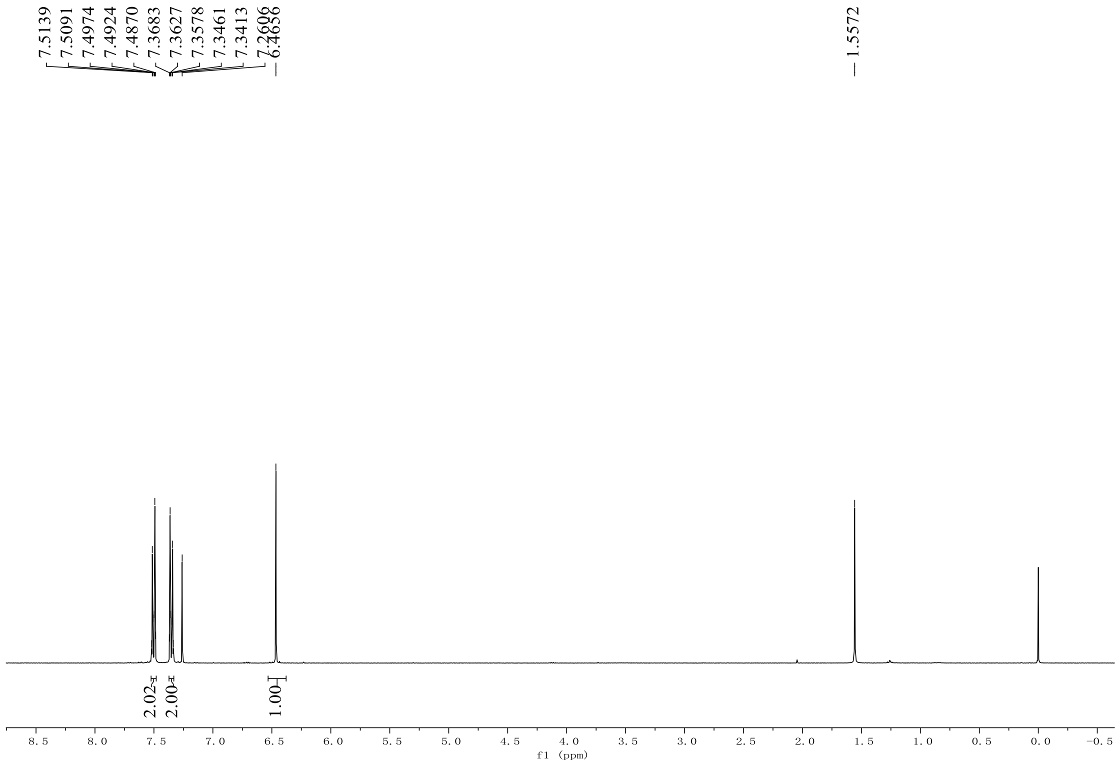
7.5139
7.5091
7.4974
7.4924
7.4870
7.3683
7.3627
7.3578
7.3461
7.3413
7.2606
6.4656
1.5572
2.02
2.00
1.00
8.5 8.0 7.5 7.0 6.5 6.0 5.5 5.0 4.5 4.0 3.5 3.0 2.5 2.0 1.5 1.0 0.5 0.0 -0.5
f1 (ppm)

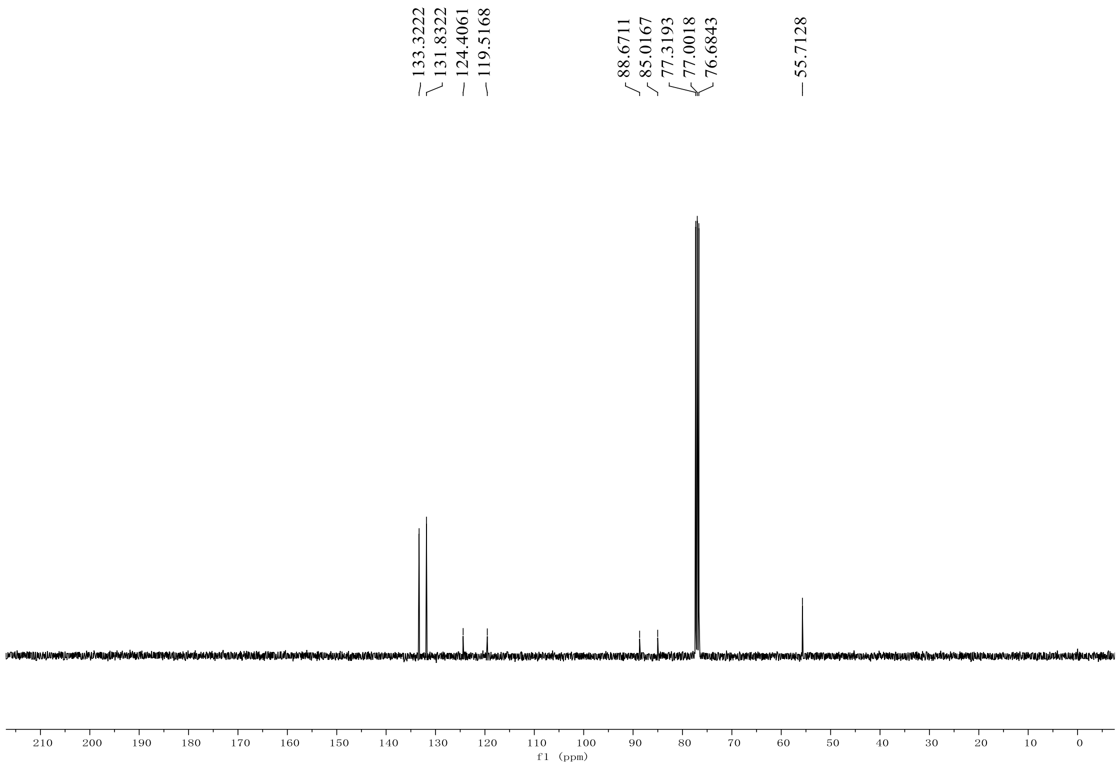
133.3222
131.8322
124.4061
119.5168
88.6711
85.0167
77.3193
77.0018
76.6843
55.7128
210 200 190 180 170 160 150 140 130 120 110 100 90 80 70 60 50 40 30 20 10 0
f1 (ppm)

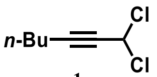
n-Bu
Cl
Cl

1m

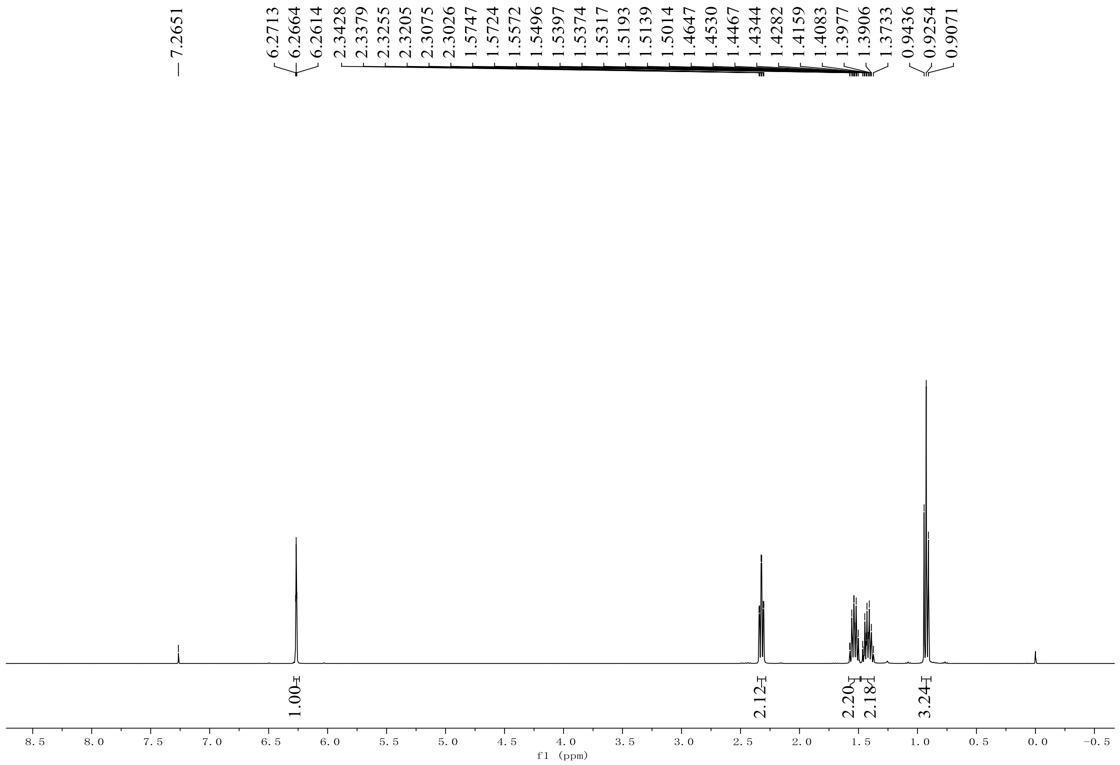
7.2651
6.2713
6.2664
6.2614
2.3428
2.3379
2.3255
2.3205
2.3075
2.3026
1.5747
1.5724
1.5572
1.5496
1.5397
1.5374
1.5317
1.5193
1.5139
1.5014
1.4647
1.4530
1.4467
1.4344
1.4282
1.4159
1.4083
1.3977
1.3906
1.3733
0.9436
0.9254
0.9071
1.00
2.12
2.20
2.18
3.24
8.5 8.0 7.5 7.0 6.5 6.0 5.5 5.0 4.5 4.0 3.5 3.0 2.5 2.0 1.5 1.0 0.5 0.0 -0.5
f1 (ppm)

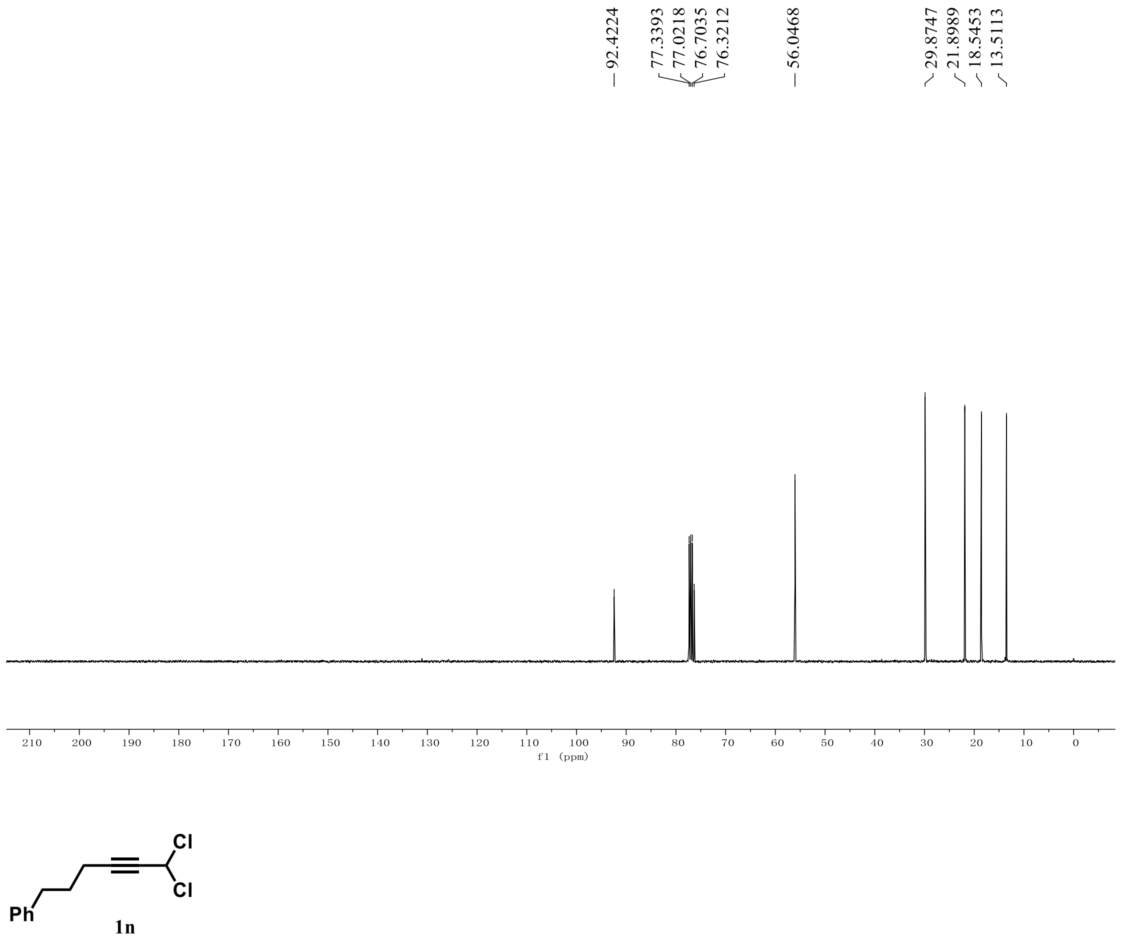
92.4224
77.3393
77.0218
76.7035
76.3212
56.0468
29.8747
21.8989
18.5453
13.5113
210 200 190 180 170 160 150 140 130 120 110 100 90 80 70 60 50 40 30 20 10 0
f1 (ppm)

Cl
Cl
Ph

1n

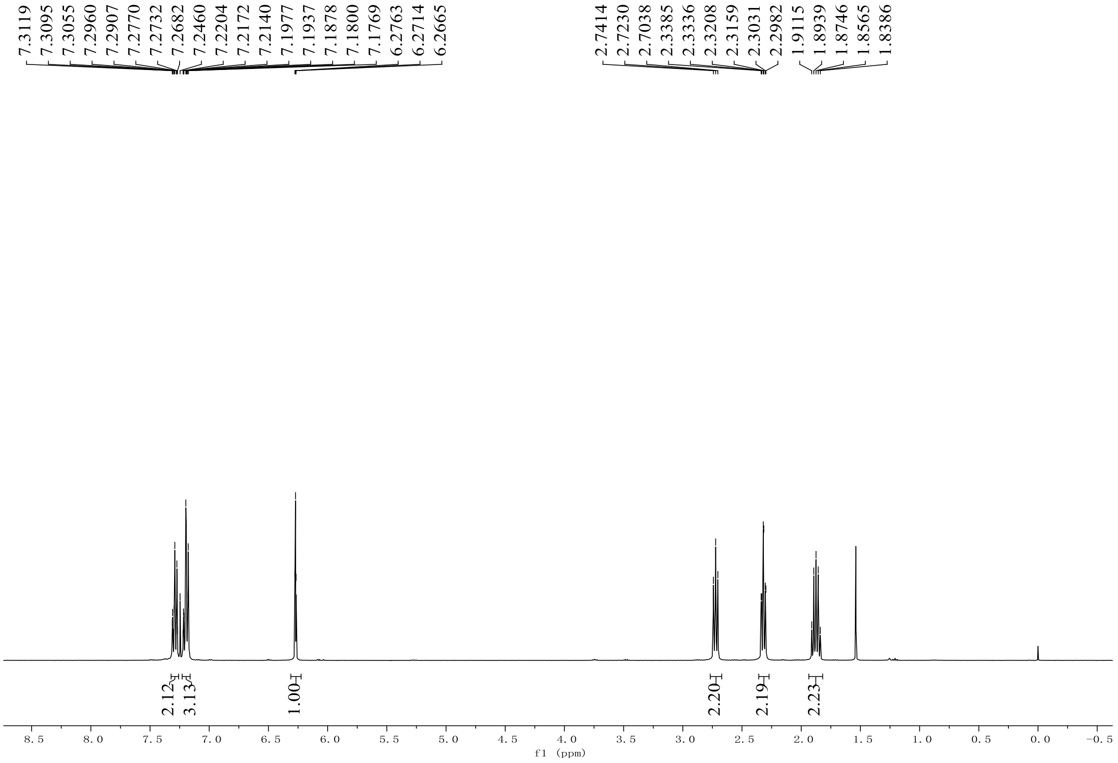
7.3119
7.3095
7.3055
7.2960
7.2907
7.2770
7.2732
7.2682
7.2460
7.2204
7.2172
7.2140
7.1977
7.1937
7.1878
7.1800
7.1769
6.2763
6.2714
6.2665
2.7414
2.7230
2.7038
2.3385
2.3336
2.3208
2.3159
2.3031
2.2982
1.9115
1.8939
1.8746
1.8565
1.8386
2.12
3.13
1.00
2.20
2.19
2.23
f1 (ppm)

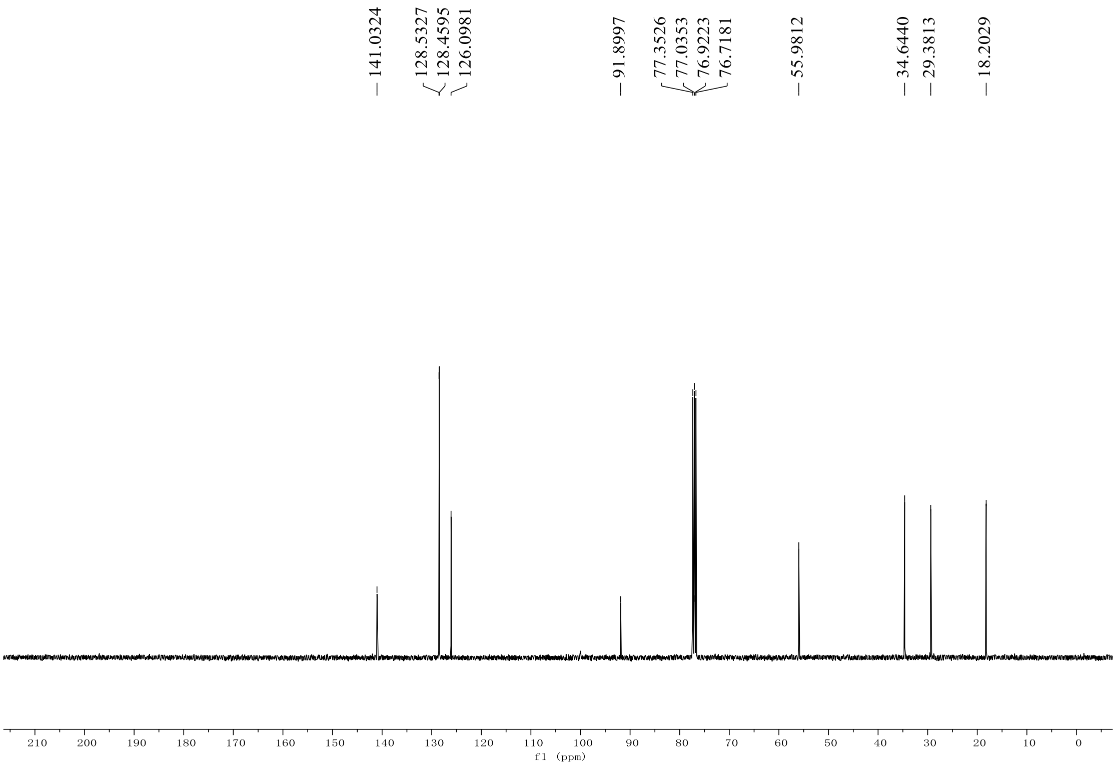
141.0324
128.5327
128.4595
126.0981
91.8997
77.3526
77.0353
76.9223
76.7181
55.9812
34.6440
29.3813
18.2029
f1 (ppm)

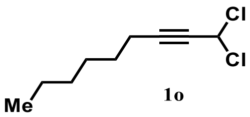
Cl
Cl
Me

1o

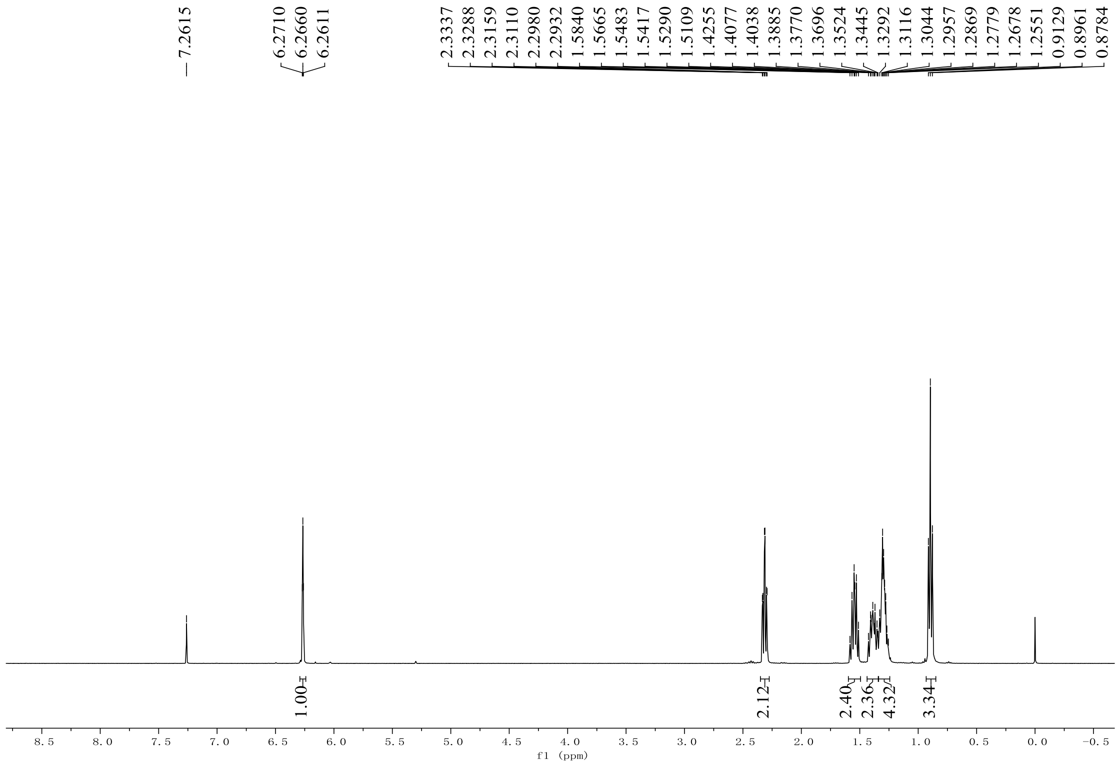
7.2615
6.2710
6.2660
6.2611
2.3337
2.3288
2.3159
2.3110
2.2980
2.2932
1.5840
1.5665
1.5483
1.5417
1.5290
1.5109
1.4255
1.4077
1.4038
1.3885
1.3770
1.3696
1.3524
1.3445
1.3292
1.3116
1.3044
1.2957
1.2869
1.2779
1.2678
1.2551
0.9129
0.8961
0.8784
1.00
2.12
2.40
2.36
4.32
3.34
f1 (ppm)

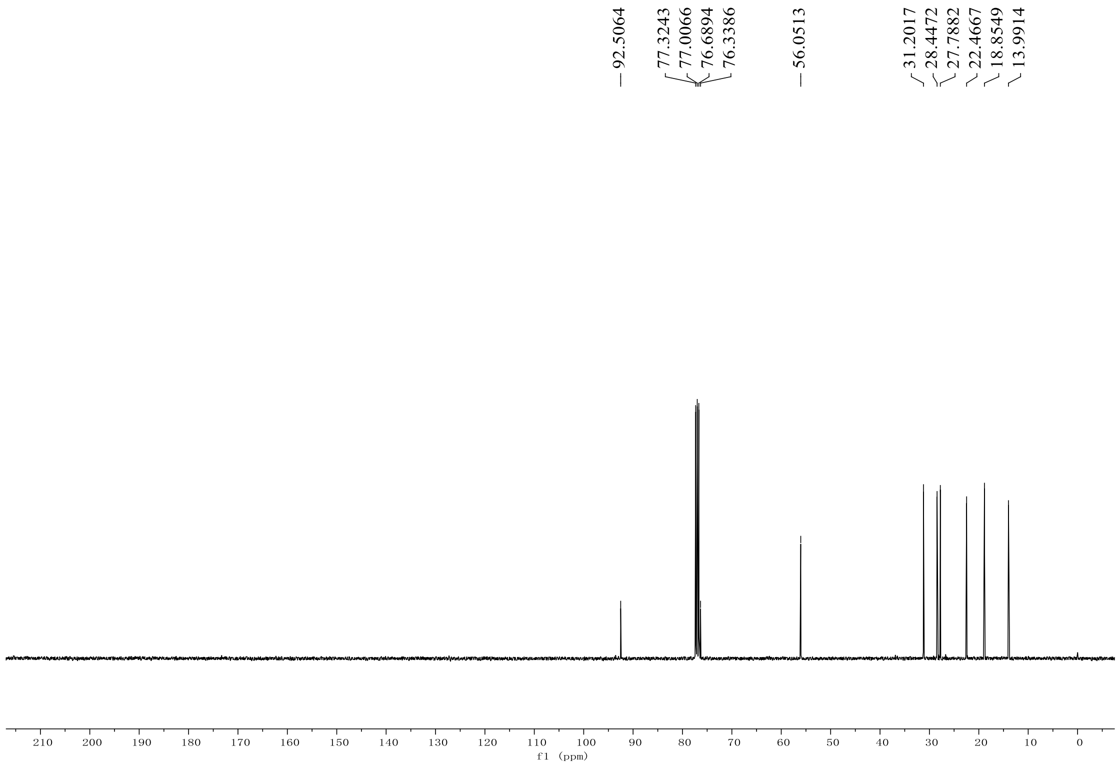
92.5064
77.3243
77.0066
76.6894
76.3386
56.0513
31.2017
28.4472
27.7882
22.4667
18.8549
13.9914
f1 (ppm)

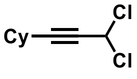
Cy
Cl
Cl

1p

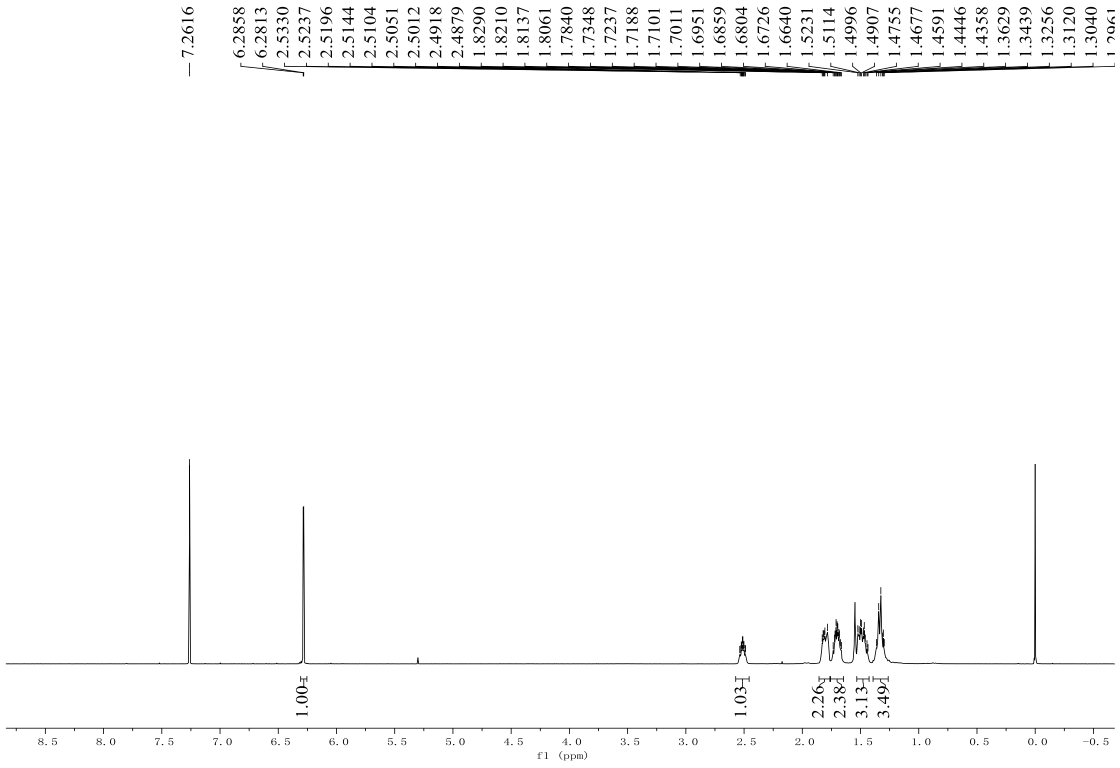
7.2616
6.2858
6.2813
2.5330
2.5237
2.5196
2.5144
2.5104
2.5051
2.5012
2.4918
2.4879
1.8290
1.8210
1.8137
1.8061
1.7840
1.7348
1.7237
1.7188
1.7101
1.7011
1.6951
1.6859
1.6804
1.6726
1.6640
1.5231
1.5114
1.4996
1.4907
1.4755
1.4677
1.4591
1.4446
1.4358
1.3629
1.3439
1.3256
1.3120
1.3040
1.00
1.03
2.26
2.38
3.13
3.49
8.5 8.0 7.5 7.0 6.5 6.0 5.5 5.0 4.5 4.0 3.5 3.0 2.5 2.0 1.5 1.0 0.5 0.0 -0.5
f1 (ppm)

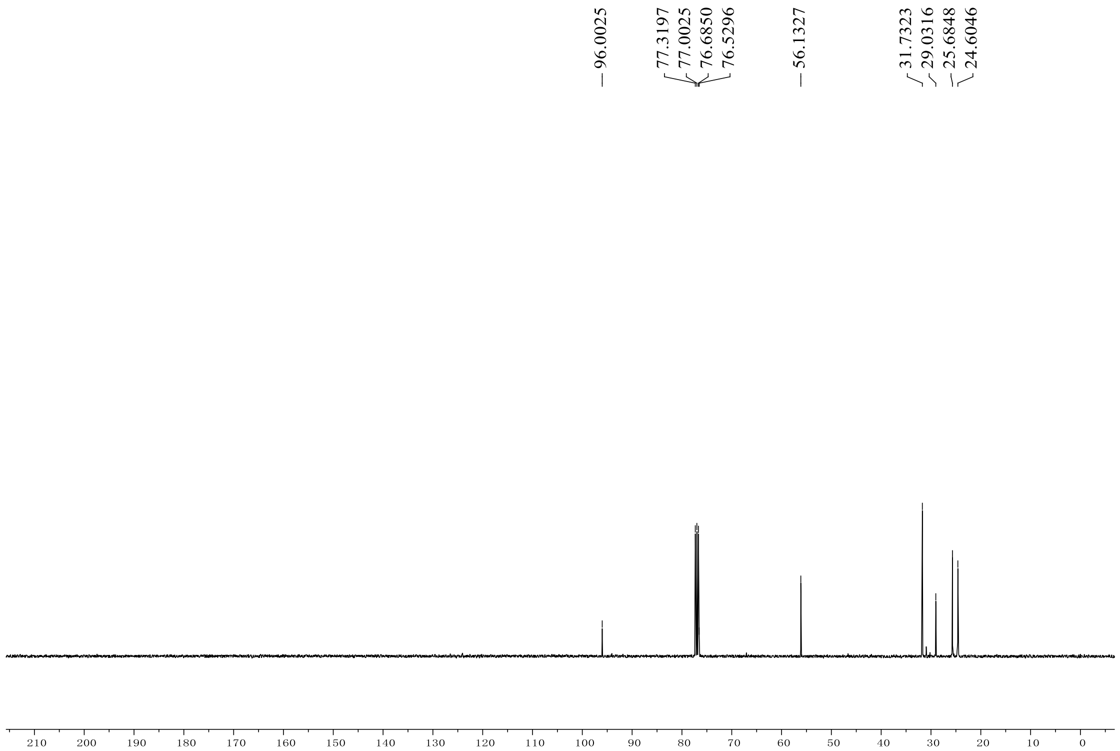
96.0025
77.3197
77.0025
76.6850
76.5296
56.1327
31.7323
29.0316
25.6848
24.6046
210 200 190 180 170 160 150 140 130 120 110 100 90 80 70 60 50 40 30 20 10 0
f1 (ppm)

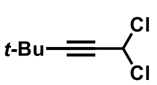
t-Bu
Cl
Cl

1q

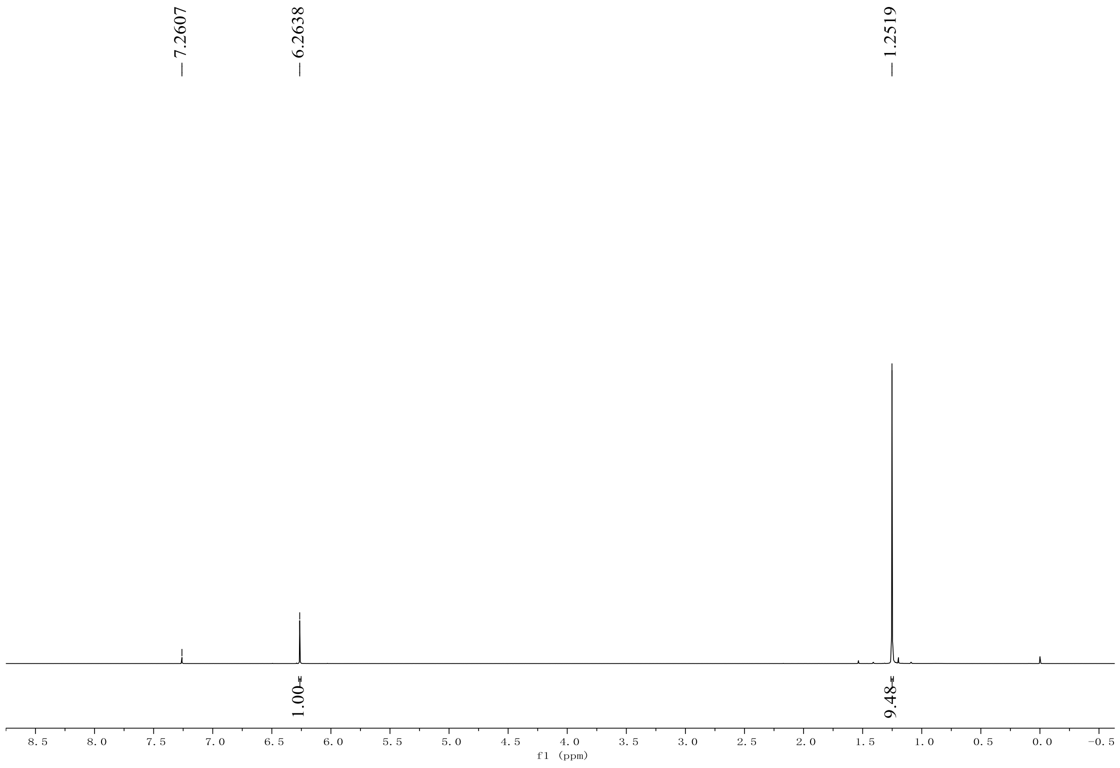
7.2607
6.2638
1.2519
1.00
9.48
8.5 8.0 7.5 7.0 6.5 6.0 5.5 5.0 4.5 4.0 3.5 3.0 2.5 2.0 1.5 1.0 0.5 0.0 -0.5
f1 (ppm)

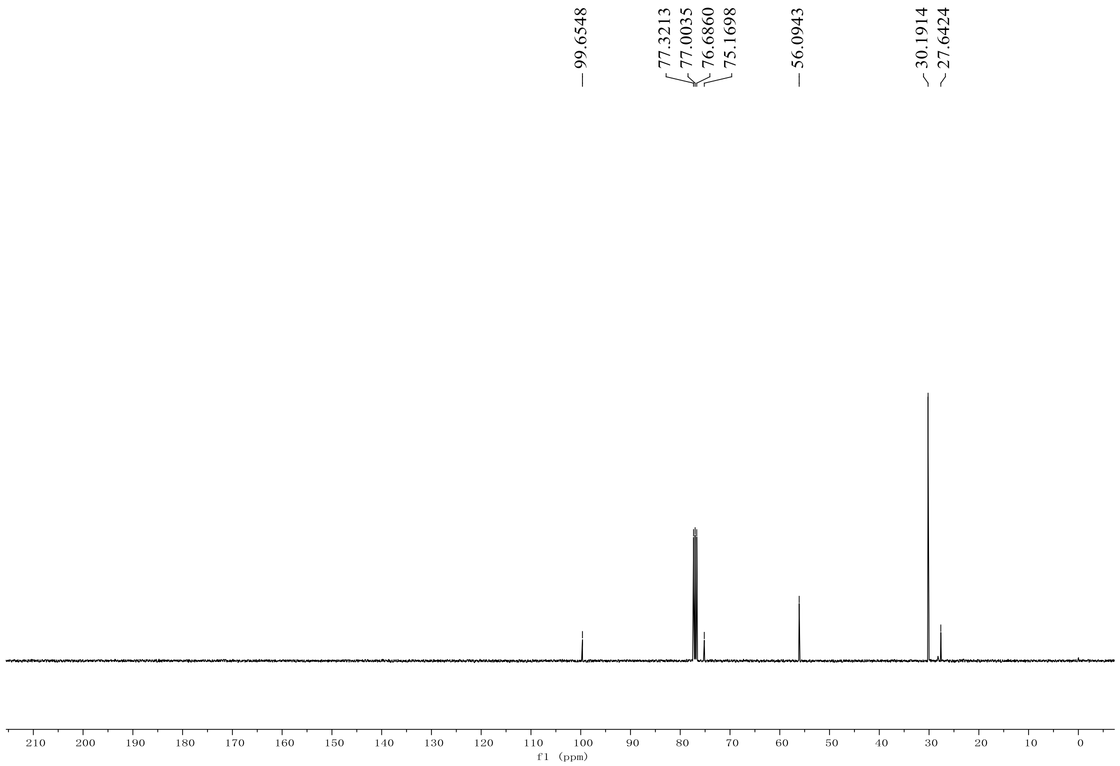
99.6548
77.3213
77.0035
76.6860
75.1698
56.0943
30.1914
27.6424
210 200 190 180 170 160 150 140 130 120 110 100 90 80 70 60 50 40 30 20 10 0
f1 (ppm)

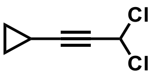
Cl
Cl

1r

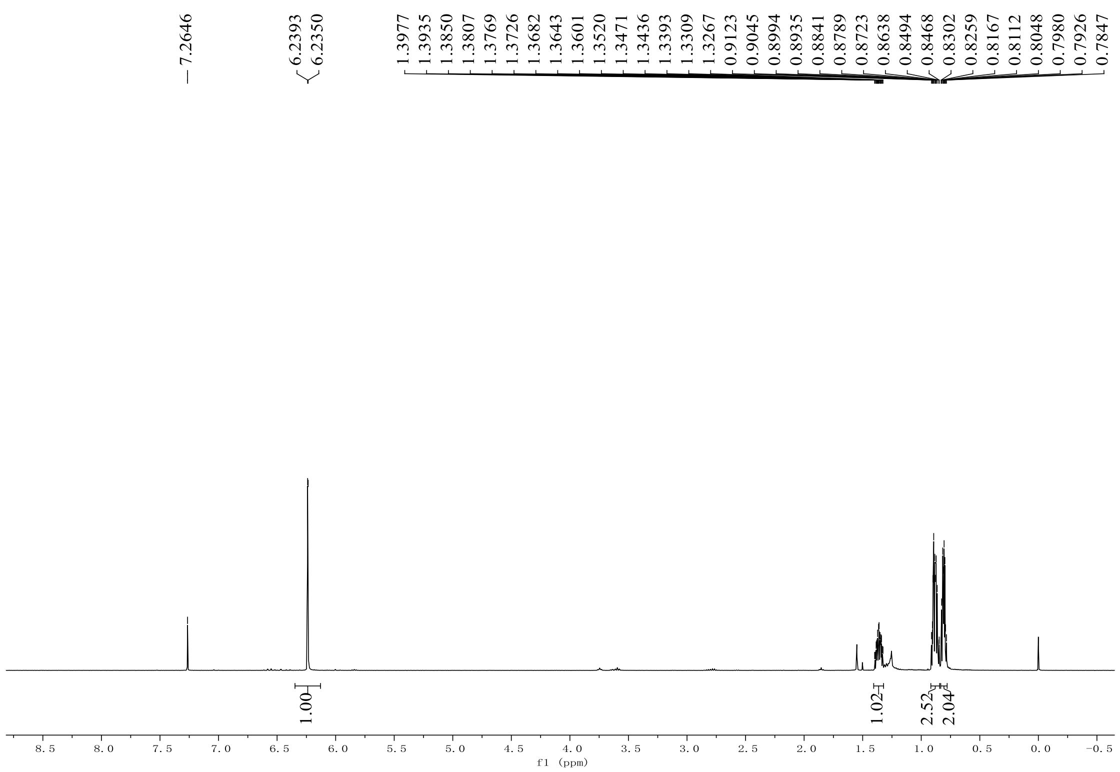

7.2646
6.2393
6.2350
1.3977
1.3935
1.3850
1.3807
1.3769
1.3726
1.3682
1.3643
1.3601
1.3520
1.3471
1.3436
1.3393
1.3309
1.3267
0.9123
0.9045
0.8994
0.8935
0.8841
0.8789
0.8723
0.8638
0.8494
0.8468
0.8302
0.8259
0.8167
0.8112
0.8048
0.7980
0.7926
0.7847
1.00
1.02
2.52
2.04
f1 (ppm)

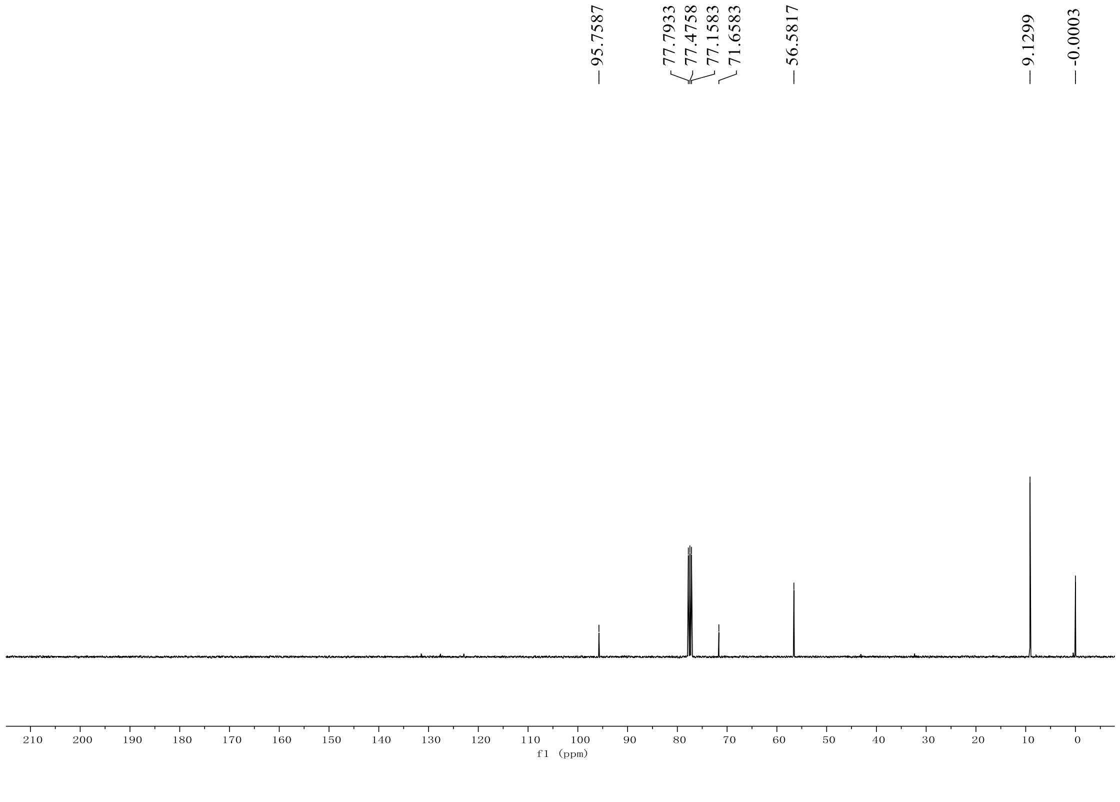

95.7587
77.7933
77.4758
77.1583
71.6583
56.5817
9.1299
-0.0003
f1 (ppm)

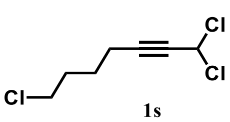

Cl
Cl
Cl

1s

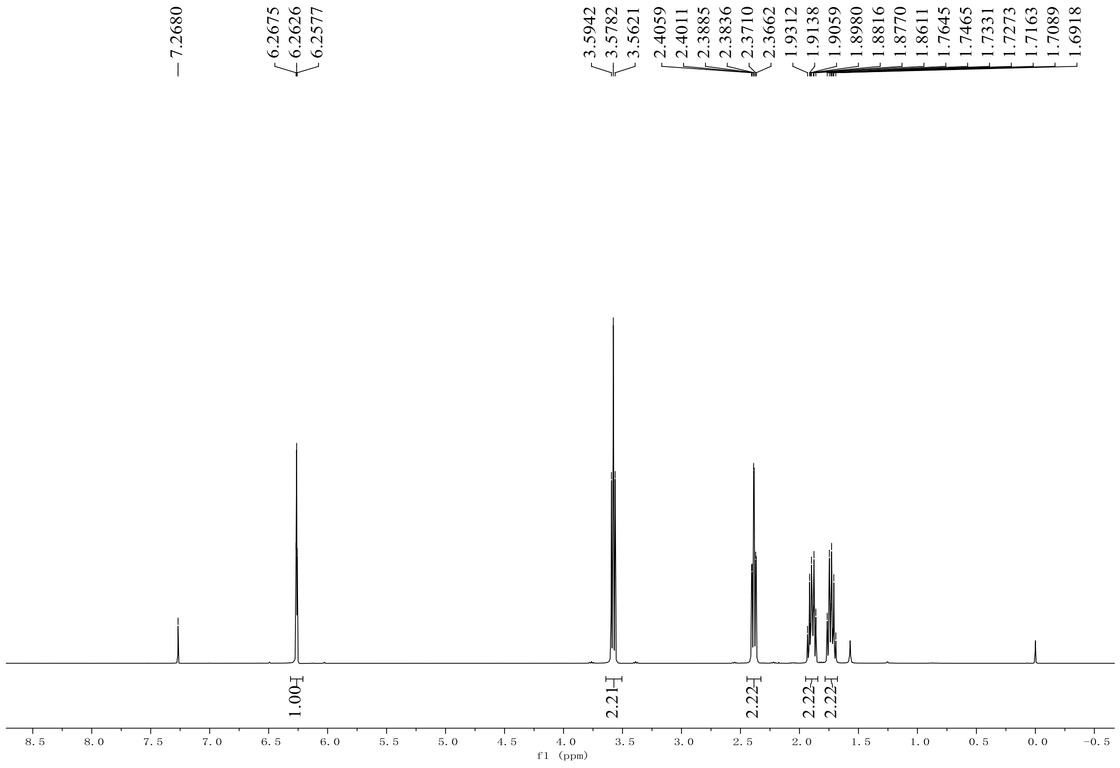
7.2680
6.2675
6.2626
6.2577
3.5942
3.5782
3.5621
2.4059
2.4011
2.3885
2.3836
2.3710
2.3662
1.9312
1.9138
1.9059
1.8980
1.8816
1.8770
1.8611
1.7645
1.7465
1.7331
1.7273
1.7163
1.7089
1.6918
1.00
2.21
2.22
2.22
2.22
f1 (ppm)

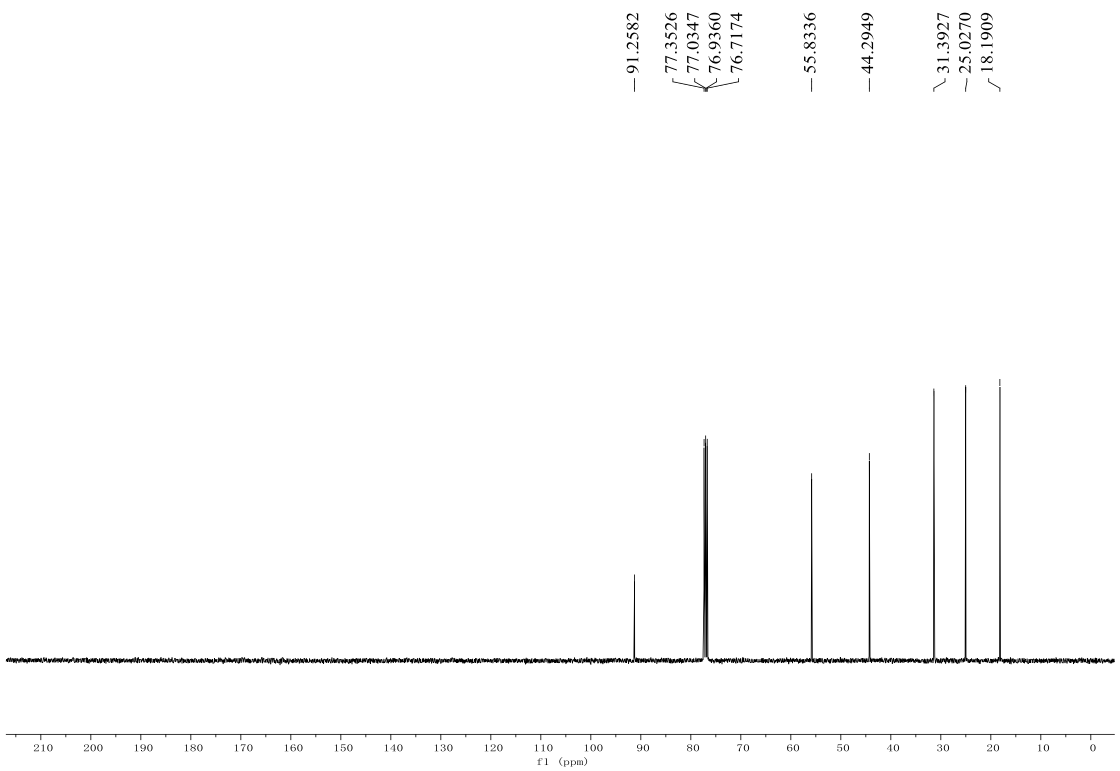
91.2582
77.3526
77.0347
76.9360
76.7174
55.8336
44.2949
31.3927
25.0270
18.1909
f1 (ppm)

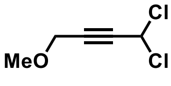
Cl
MeO
Cl

1t

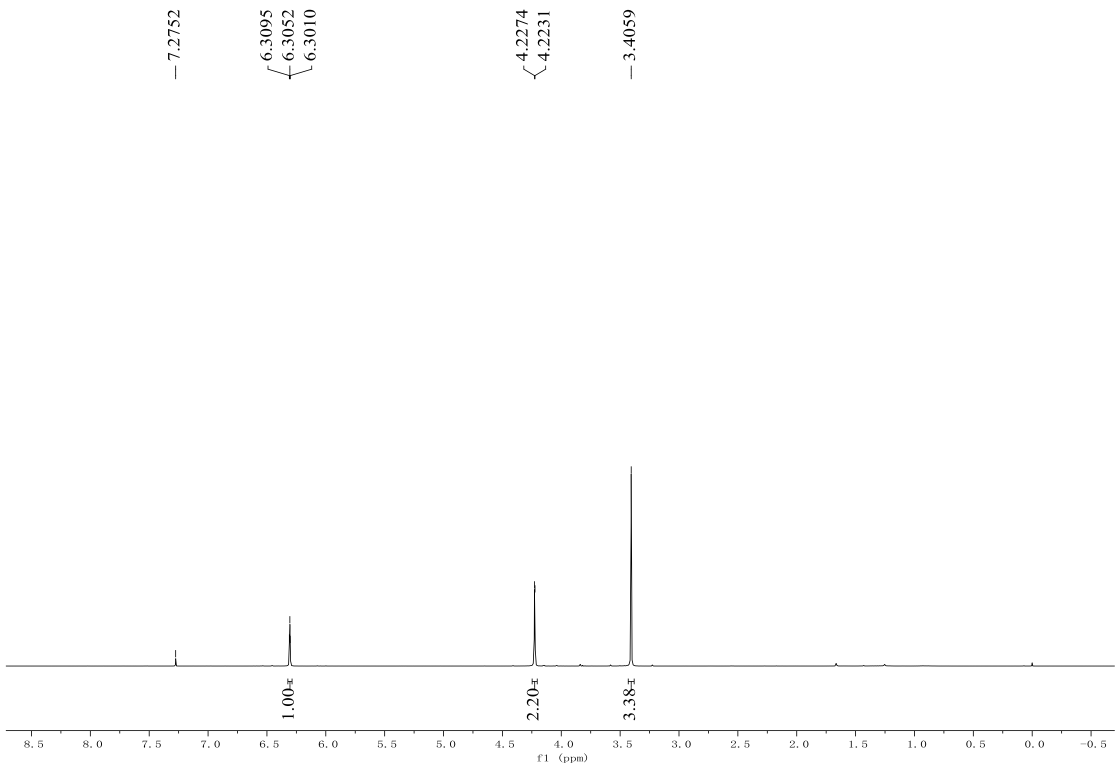
7.2752
6.3095
6.3052
6.3010
4.2274
4.2231
3.4059
1.00
2.20
3.38
8.5 8.0 7.5 7.0 6.5 6.0 5.5 5.0 4.5 4.0 3.5 3.0 2.5 2.0 1.5 1.0 0.5 0.0 -0.5
f1 (ppm)

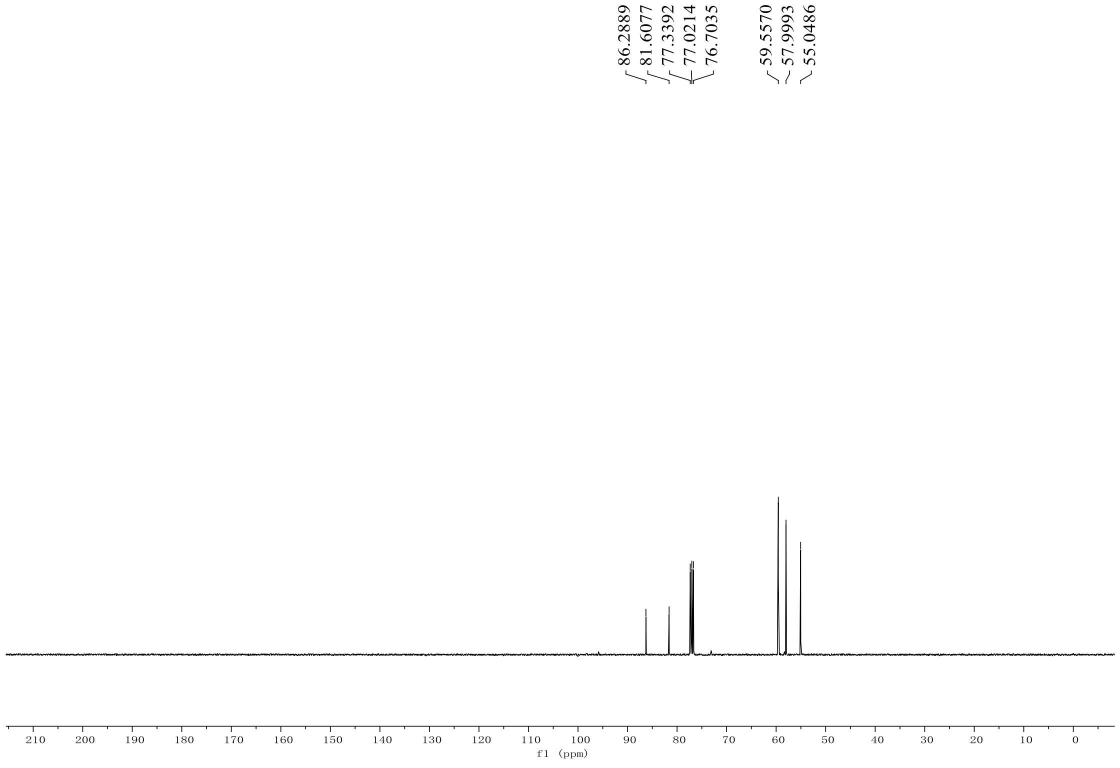
86.2889
81.6077
77.3392
77.0214
76.7035
59.5570
57.9993
55.0486
210 200 190 180 170 160 150 140 130 120 110 100 90 80 70 60 50 40 30 20 10 0
f1 (ppm)

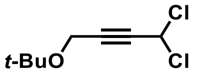
Cl
t-BuO
Cl

1u

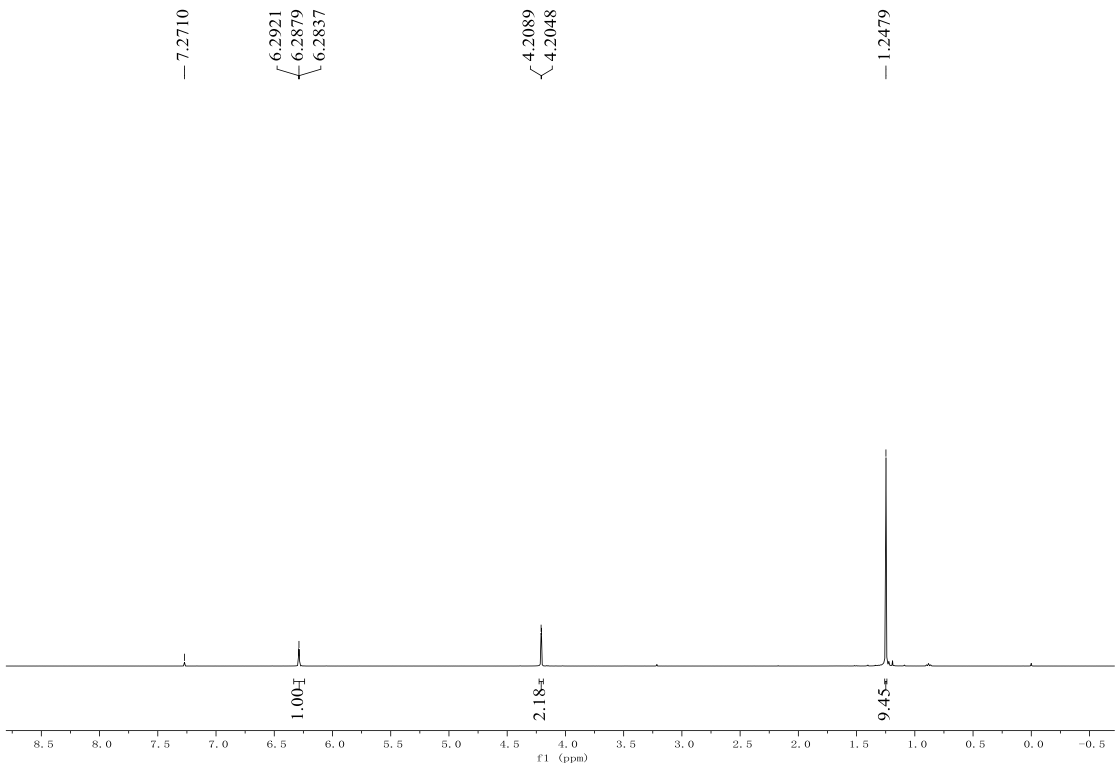
7.2710
6.2921
6.2879
6.2837
4.2089
4.2048
1.2479
1.00
2.18
9.45
8.5 8.0 7.5 7.0 6.5 6.0 5.5 5.0 4.5 4.0 3.5 3.0 2.5 2.0 1.5 1.0 0.5 0.0 -0.5
f1 (ppm)

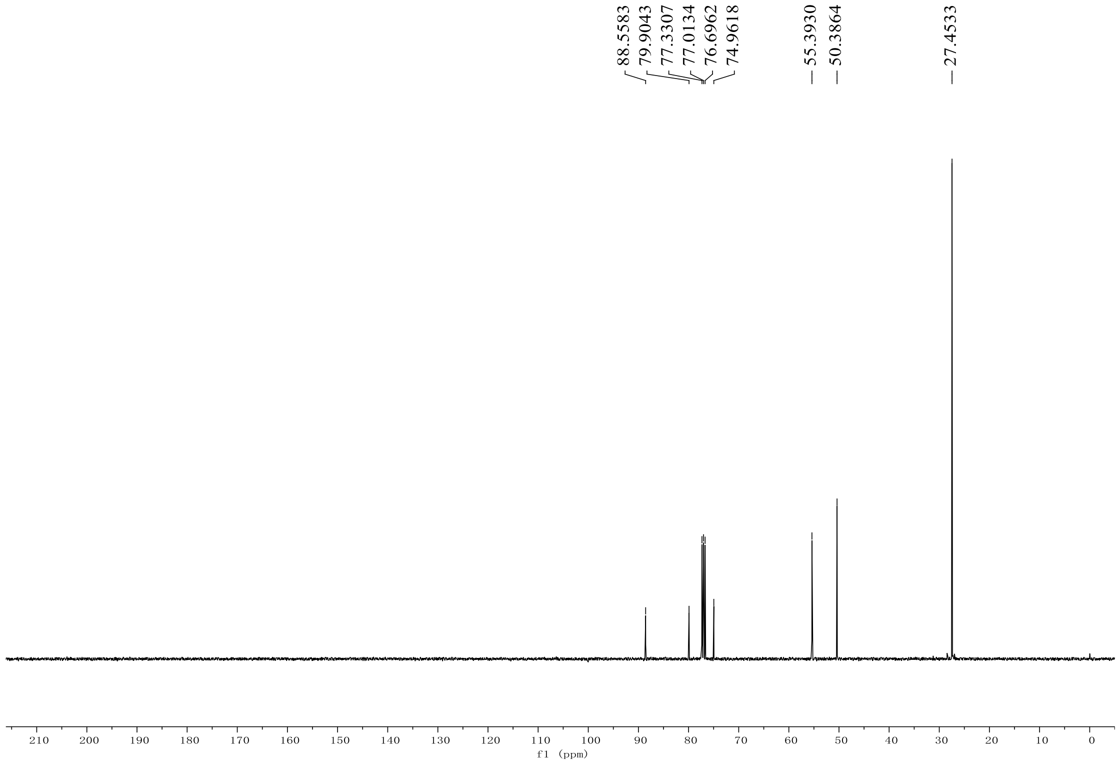
88.5583
79.9043
77.3307
77.0134
76.6962
74.9618
55.3930
50.3864
27.4533
210 200 190 180 170 160 150 140 130 120 110 100 90 80 70 60 50 40 30 20 10 0
f1 (ppm)

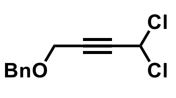
Cl
BnO
Cl

1v

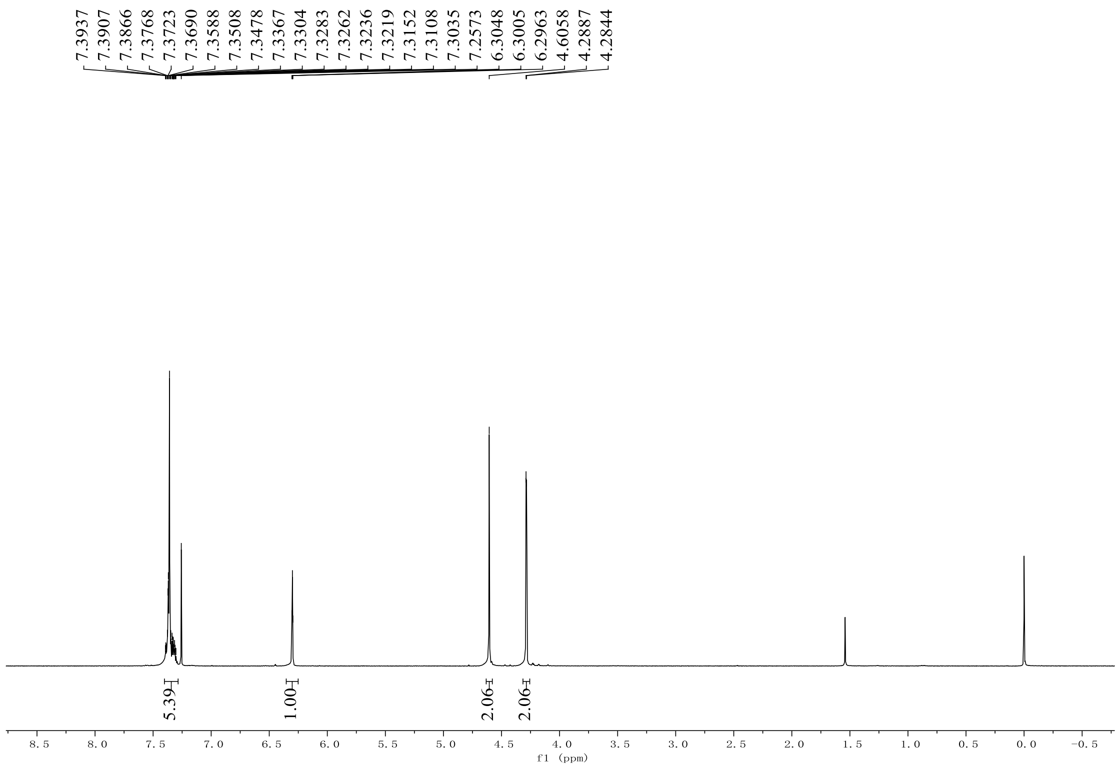
7.3937
7.3907
7.3866
7.3768
7.3723
7.3690
7.3588
7.3508
7.3478
7.3367
7.3304
7.3283
7.3262
7.3236
7.3219
7.3152
7.3108
7.3035
7.2573
6.3048
6.3005
6.2963
4.6058
4.2887
4.2844
5.39
1.00
2.06
2.06
f1 (ppm)

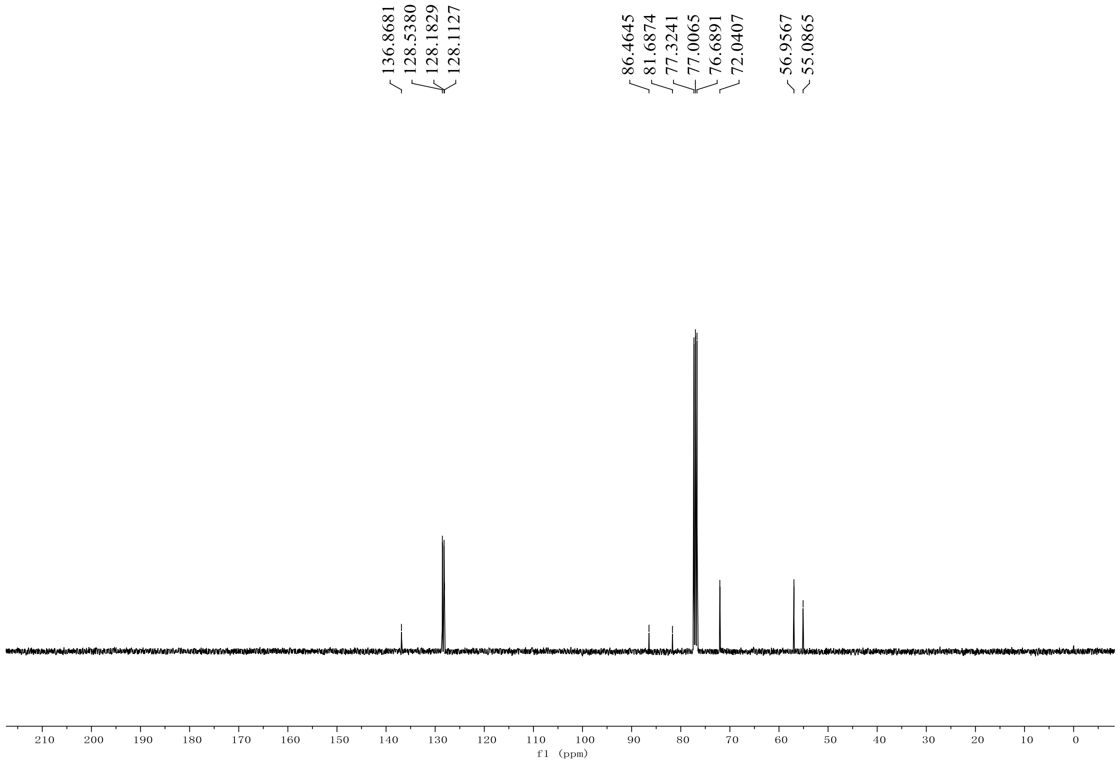
136.8681
128.5380
128.1829
128.1127
86.4645
81.6874
77.3241
77.0065
76.6891
72.0407
56.9567
55.0865
f1 (ppm)

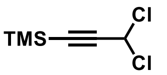
TMS
Cl
Cl

1w

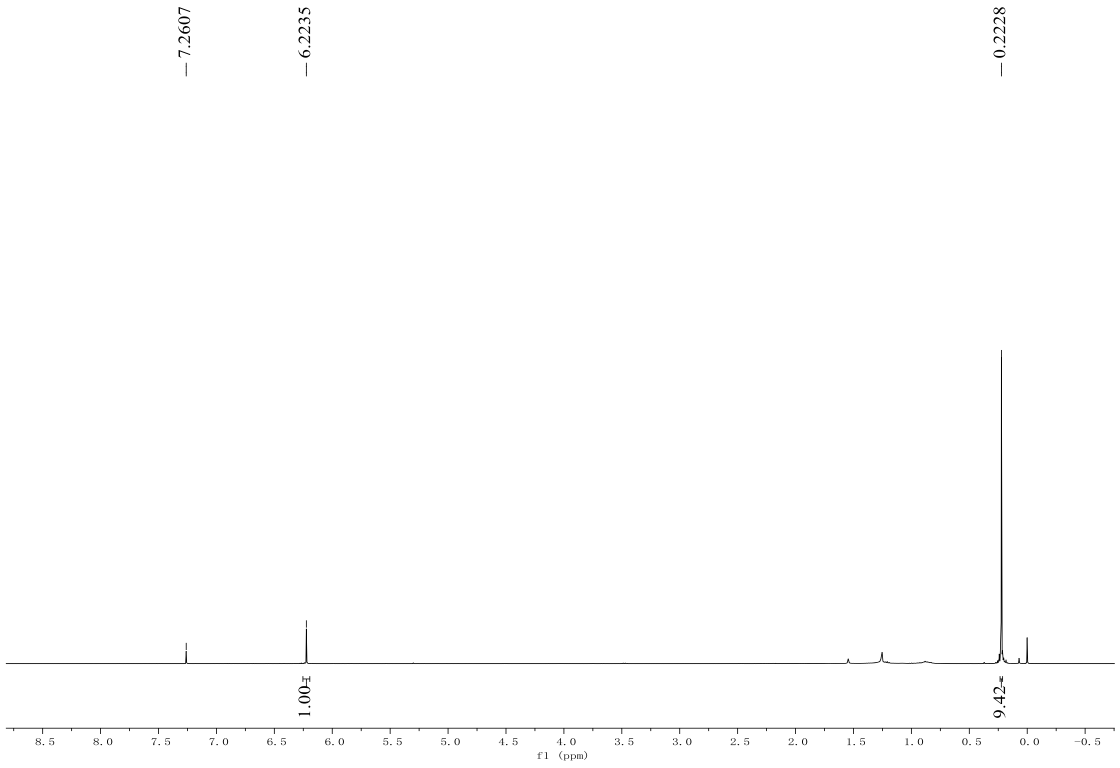
7.2607
6.2235
0.2228
1.00
9.42
8.5 8.0 7.5 7.0 6.5 6.0 5.5 5.0 4.5 4.0 3.5 3.0 2.5 2.0 1.5 1.0 0.5 0.0 -0.5
f1 (ppm)

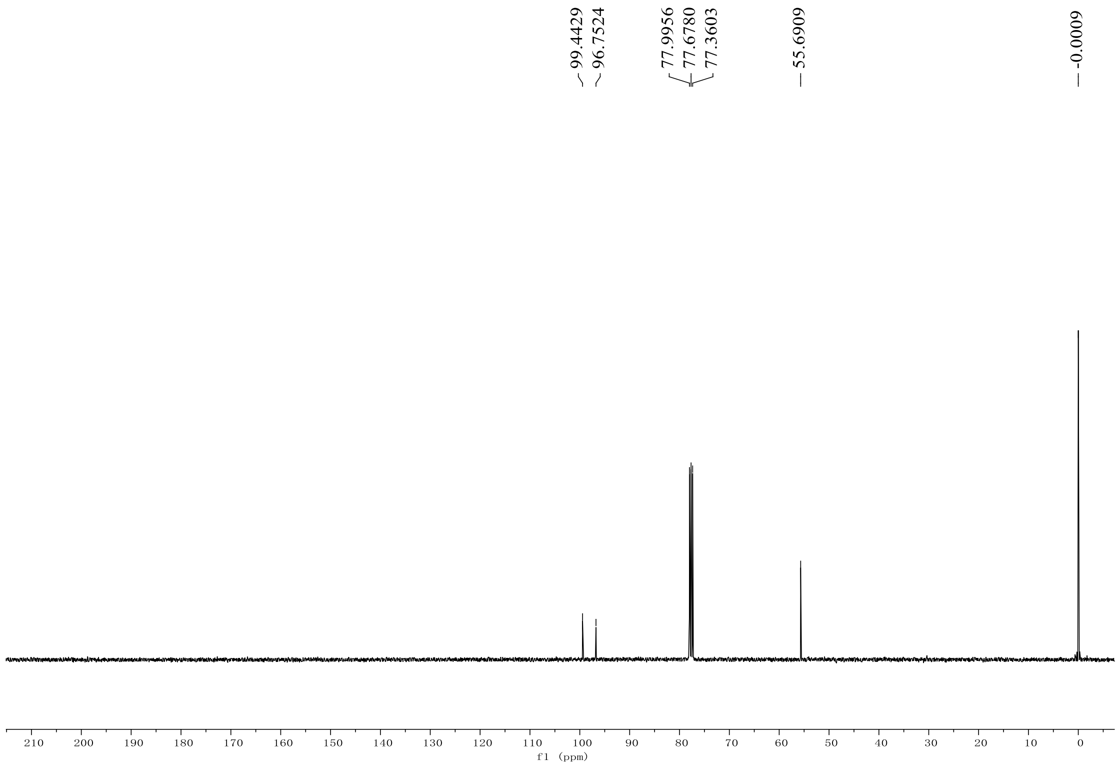
99.4429
96.7524
77.9956
77.6780
77.3603
55.6909
-0.0009
210 200 190 180 170 160 150 140 130 120 110 100 90 80 70 60 50 40 30 20 10 0
f1 (ppm)

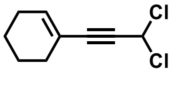
Cl
Cl

1x

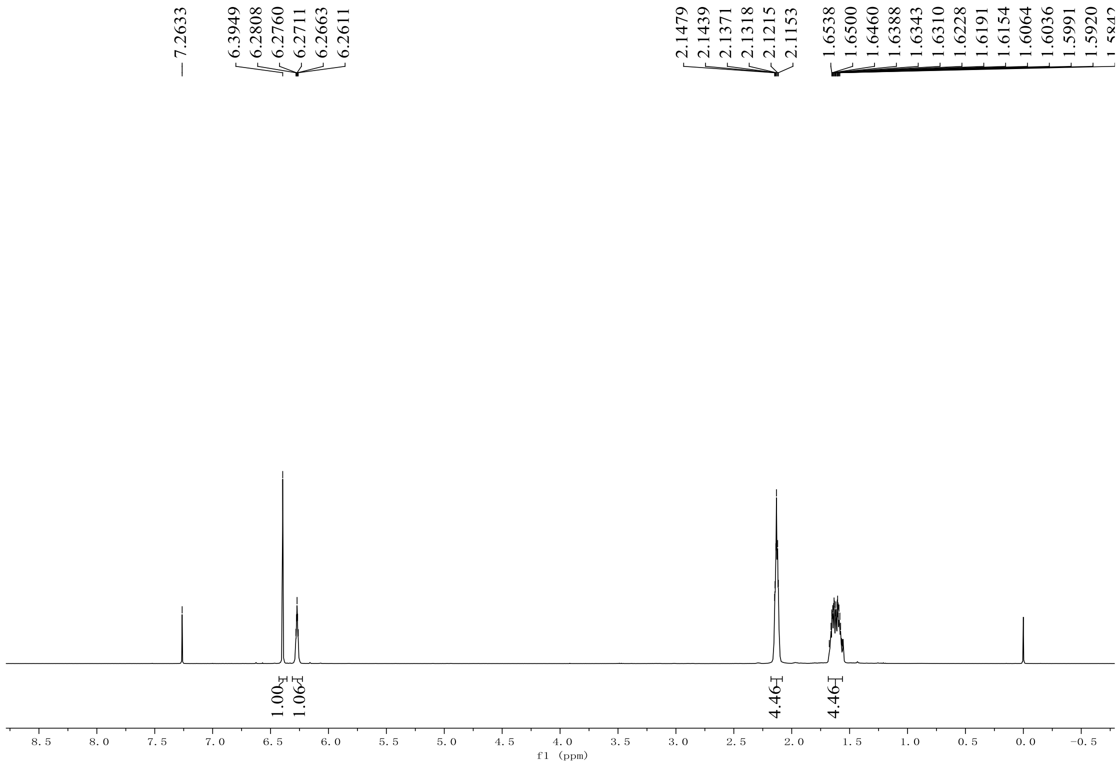
7.2633
6.3949
6.2808
6.2760
6.2711
6.2663
6.2611
2.1479
2.1439
2.1371
2.1318
2.1215
2.1153
1.6538
1.6500
1.6460
1.6388
1.6343
1.6310
1.6228
1.6191
1.6154
1.6064
1.6036
1.5991
1.5920
1.00
1.06
4.46
4.46
f1 (ppm)

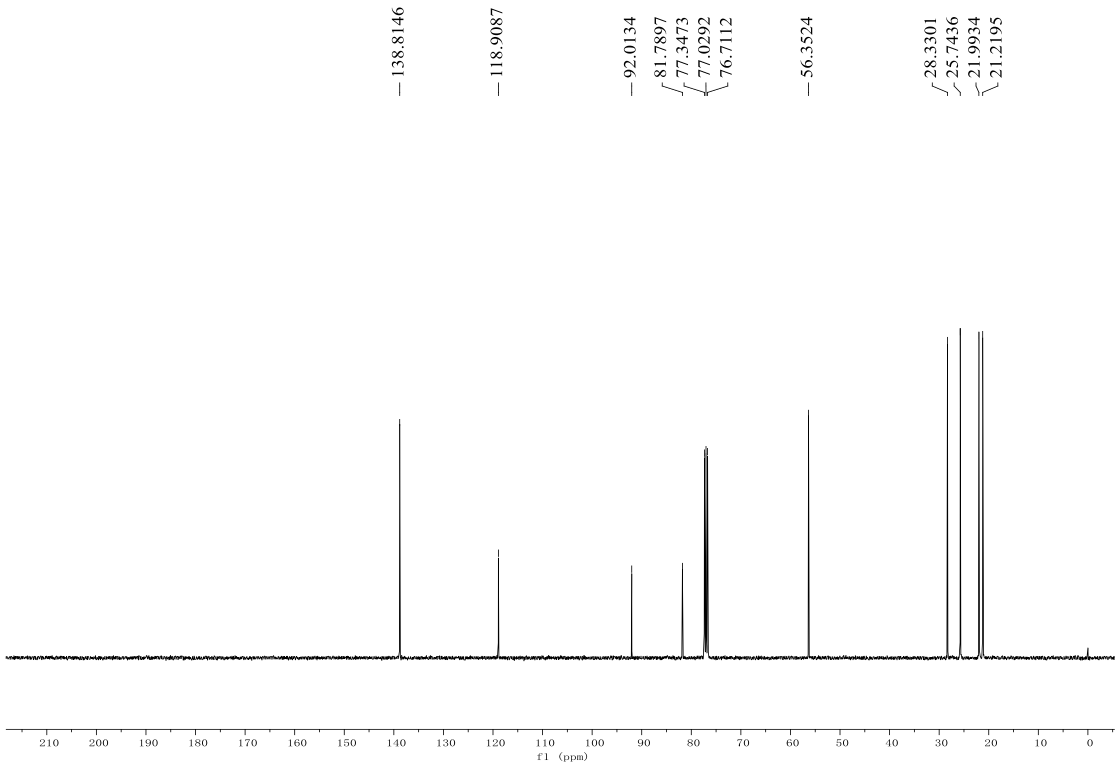
138.8146
118.9087
92.0134
81.7897
77.3473
77.0292
76.7112
56.3524
28.3301
25.7436
21.9934
21.2195
f1 (ppm)

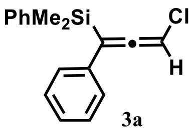

3a

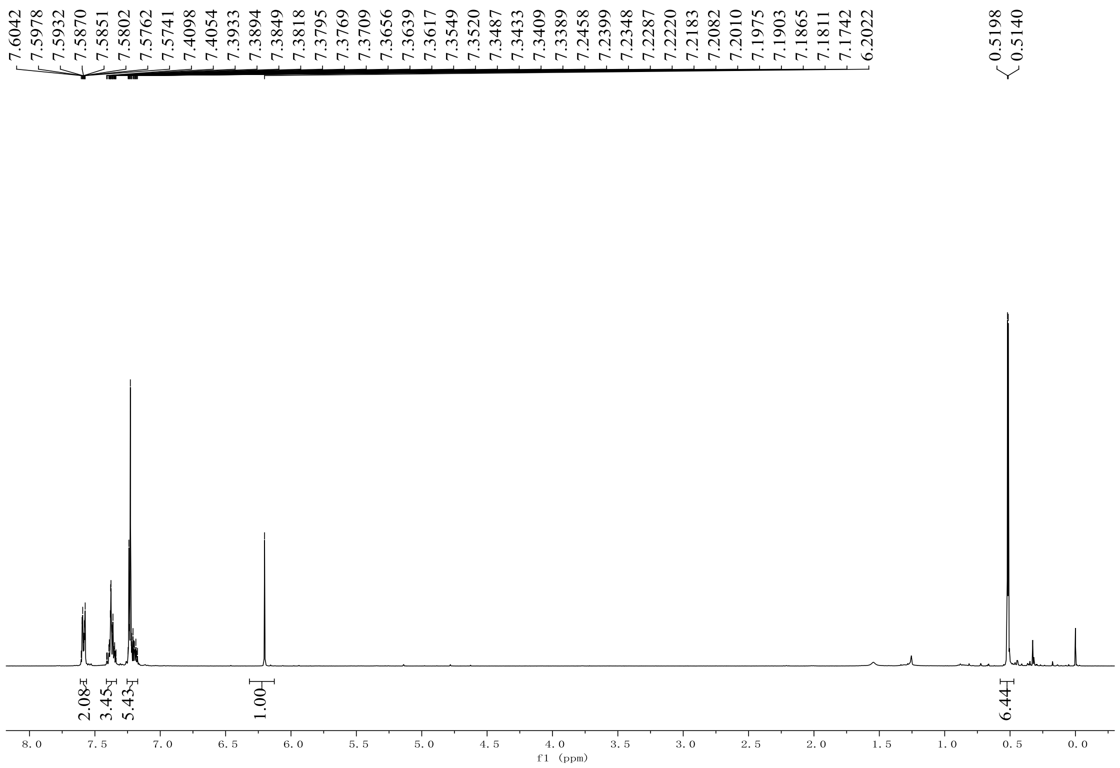
7.6042
7.5978
7.5932
7.5870
7.5851
7.5802
7.5762
7.5741
7.4098
7.4054
7.3933
7.3894
7.3849
7.3818
7.3795
7.3769
7.3709
7.3656
7.3639
7.3617
7.3549
7.3520
7.3487
7.3433
7.3409
7.3389
7.2458
7.2399
7.2348
7.2287
7.2220
7.2183
7.2082
7.2010
7.1975
7.1903
7.1865
7.1811
7.1742
6.2022
0.5198
0.5140
2.08
3.45
5.43
1.00
6.44
8.0
7.5
7.0
6.5
6.0
5.5
5.0
4.5
4.0
3.5
3.0
2.5
2.0
1.5
1.0
0.5
0.0
f1 (ppm)

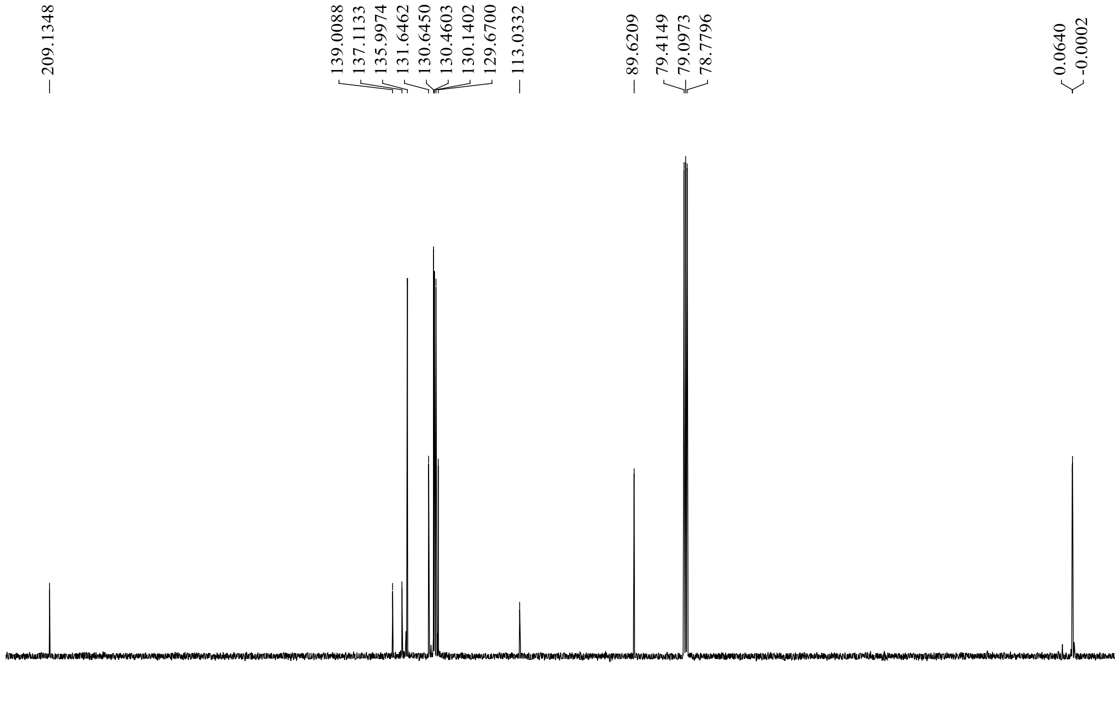
209.1348
139.0088
137.1133
135.9974
131.6462
130.6450
130.4603
130.1402
129.6700
113.0332
89.6209
79.4149
79.0973
78.7796
0.0640
-0.0002
210
200
190
180
170
160
150
140
130
120
110
100
90
80
70
60
50
40
30
20
10
0
f1 (ppm)

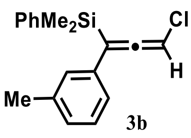
PhMe2Si
Cl
H
Me
3b

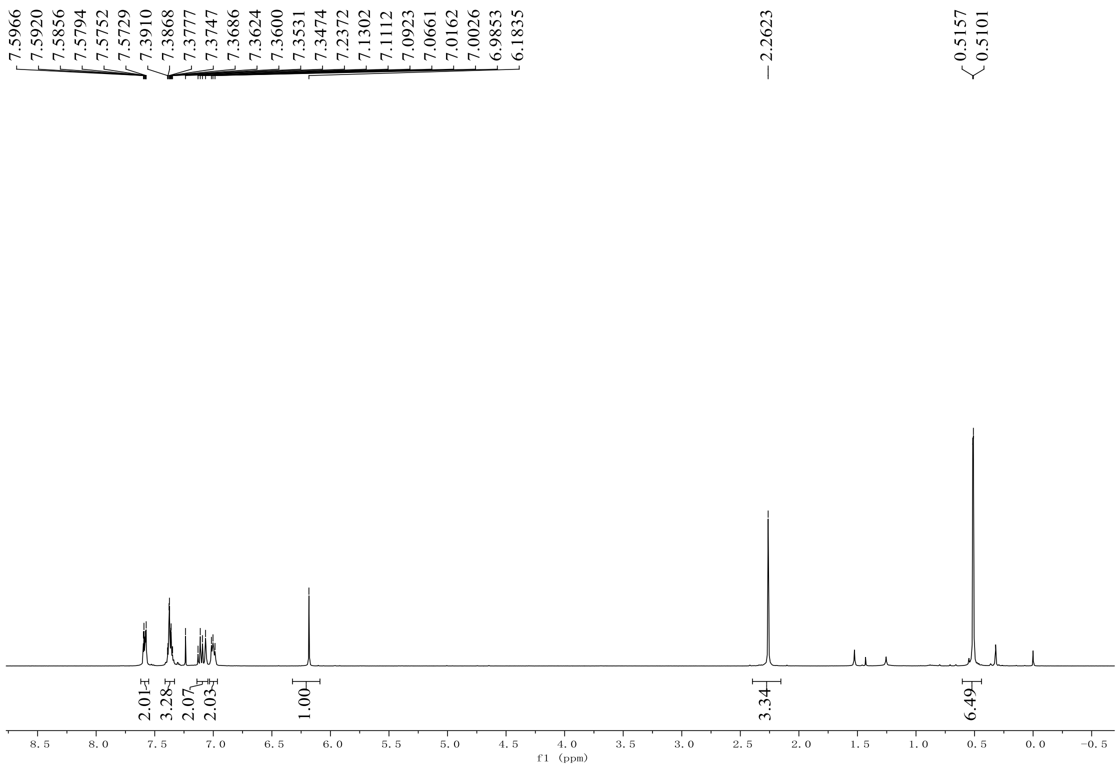
7.5966
7.5920
7.5856
7.5794
7.5752
7.5729
7.3910
7.3868
7.3777
7.3747
7.3686
7.3624
7.3600
7.3531
7.3474
7.2372
7.1302
7.1112
7.0923
7.0661
7.0162
7.0026
6.9853
6.1835
2.2623
0.5157
0.5101
2.01
3.28
2.07
2.03
1.00
3.34
6.49
f1 (ppm)

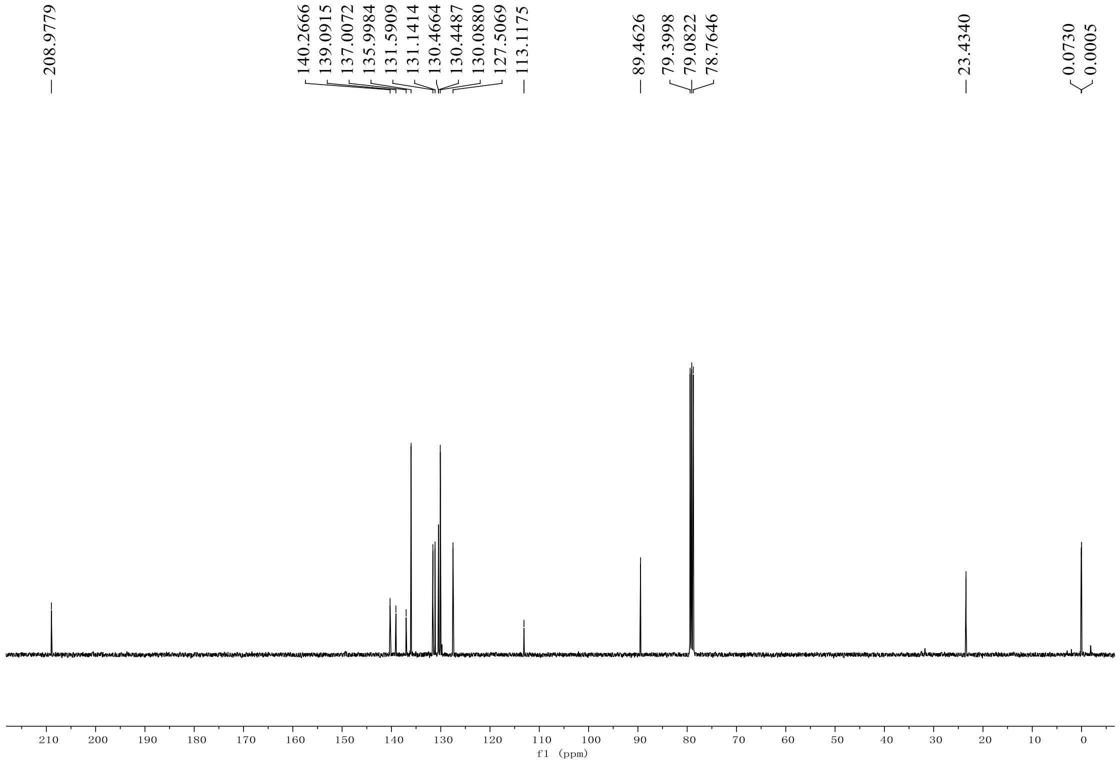
208.9779
140.2666
139.0915
137.0072
135.9984
131.5909
131.1414
130.4664
130.4487
130.0880
127.5069
113.1175
89.4626
79.3998
79.0822
78.7646
23.4340
0.0730
0.0005
f1 (ppm)

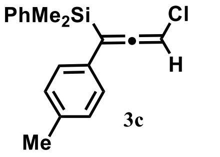
PhMe2Si
Cl
H
Me

3c

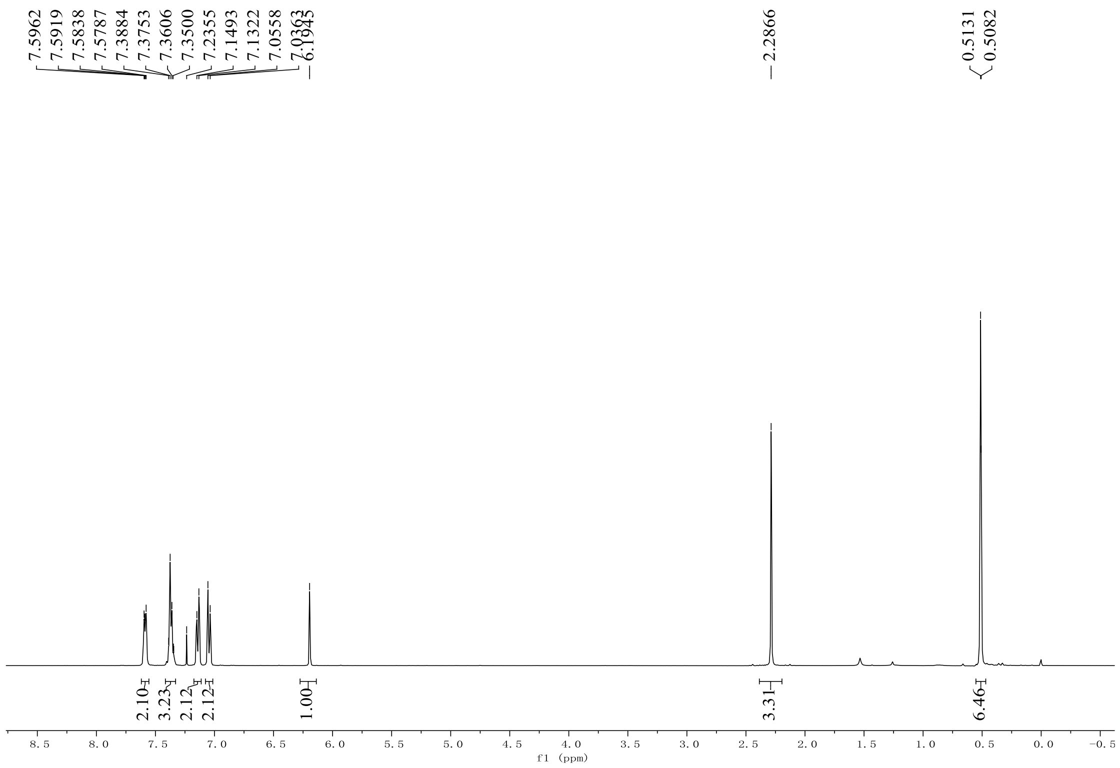
7.5962
7.5919
7.5838
7.5787
7.3884
7.3753
7.3606
7.3500
7.2355
7.1493
7.1322
7.0558
7.0363
6.1945
2.2866
0.5131
0.5082
2.10
3.23
2.12
2.12
1.00
3.31
6.46
f1 (ppm)

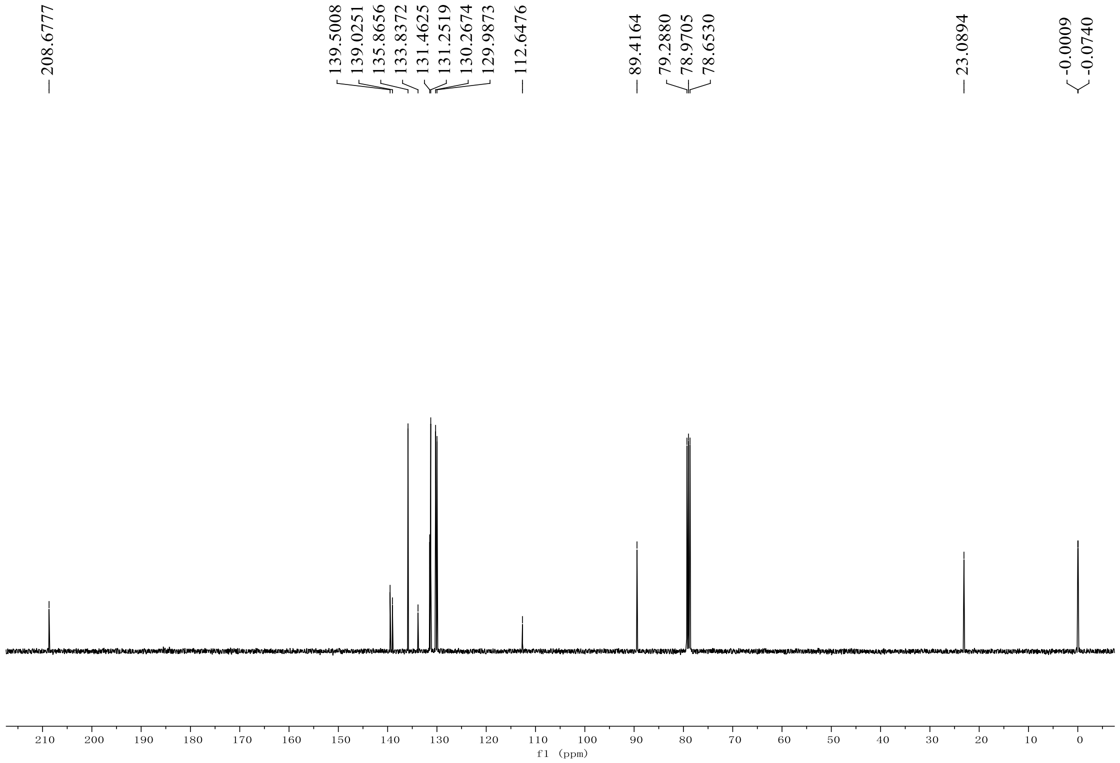
208.6777
139.5008
139.0251
135.8656
133.8372
131.4625
131.2519
130.2674
129.9873
112.6476
89.4164
79.2880
78.9705
78.6530
23.0894
-0.0009
-0.0740
f1 (ppm)

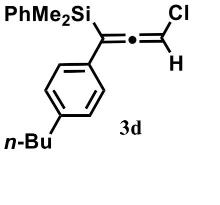
PhMe$_2$Si
Cl
H
n-Bu

3d

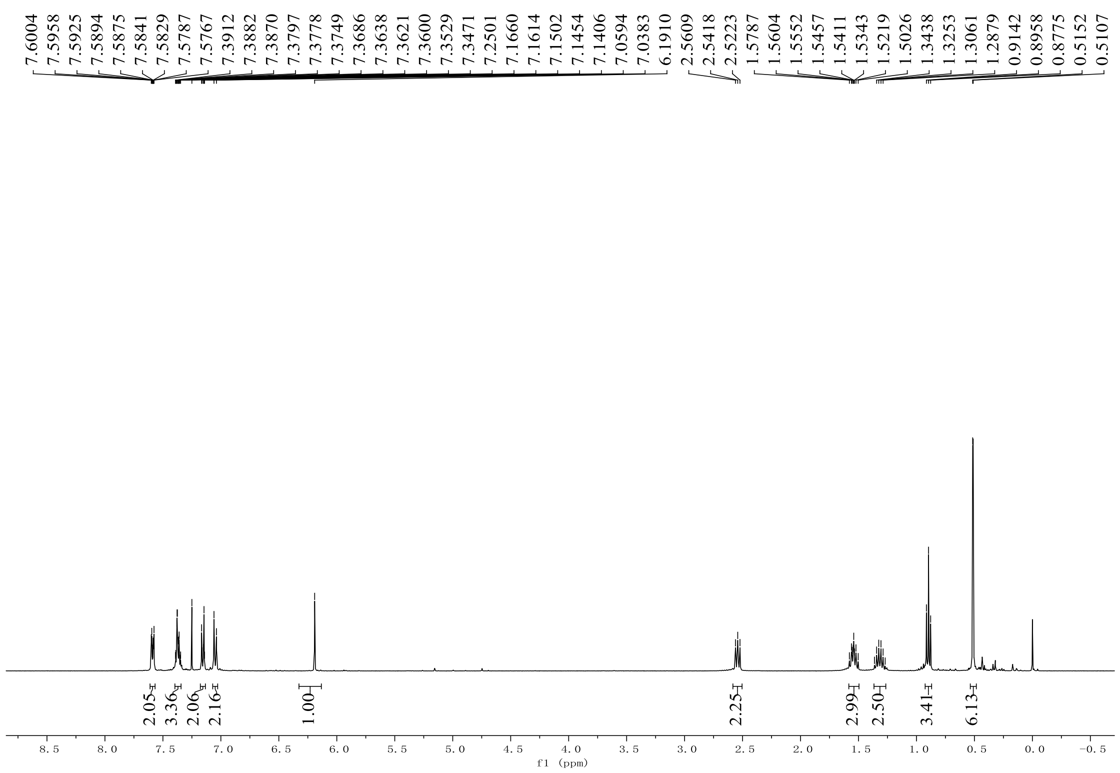
7.6004
7.5958
7.5925
7.5894
7.5875
7.5841
7.5829
7.5787
7.5767
7.3912
7.3882
7.3870
7.3797
7.3778
7.3749
7.3686
7.3638
7.3621
7.3600
7.3529
7.3471
7.2501
7.1660
7.1614
7.1502
7.1454
7.1406
7.0594
7.0383
6.1910
2.5609
2.5418
2.5223
1.5787
1.5604
1.5552
1.5457
1.5411
1.5343
1.5219
1.5026
1.3438
1.3253
1.3061
1.2879
0.9142
0.8958
0.8775
0.5152
0.5107
2.05
3.36
2.06
2.16
1.00
2.25
2.99
2.50
3.41
6.13
f1 (ppm)

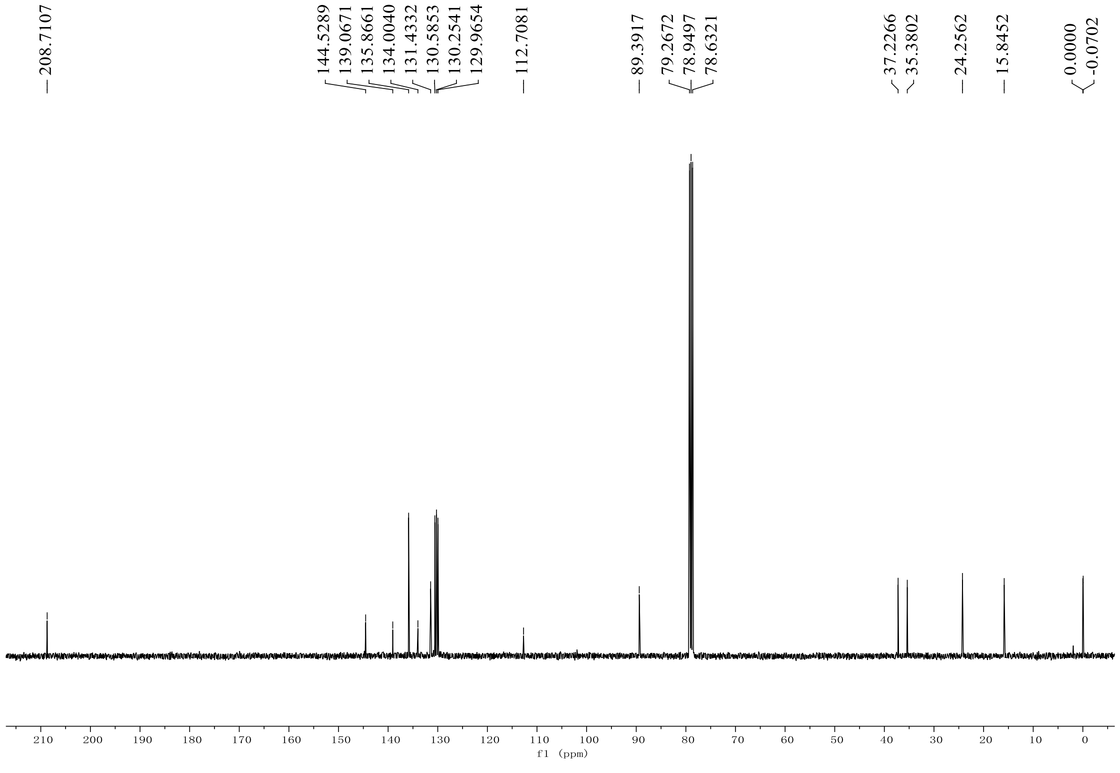
208.7107
144.5289
139.0671
135.8661
134.0040
131.4332
130.5853
130.2541
129.9654
112.7081
89.3917
79.2672
78.9497
78.6321
37.2266
35.3802
24.2562
15.8452
0.0000
-0.0702
f1 (ppm)

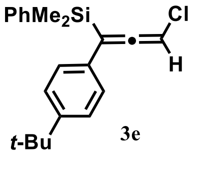
PhMe2Si
Cl
H
t-Bu

3e

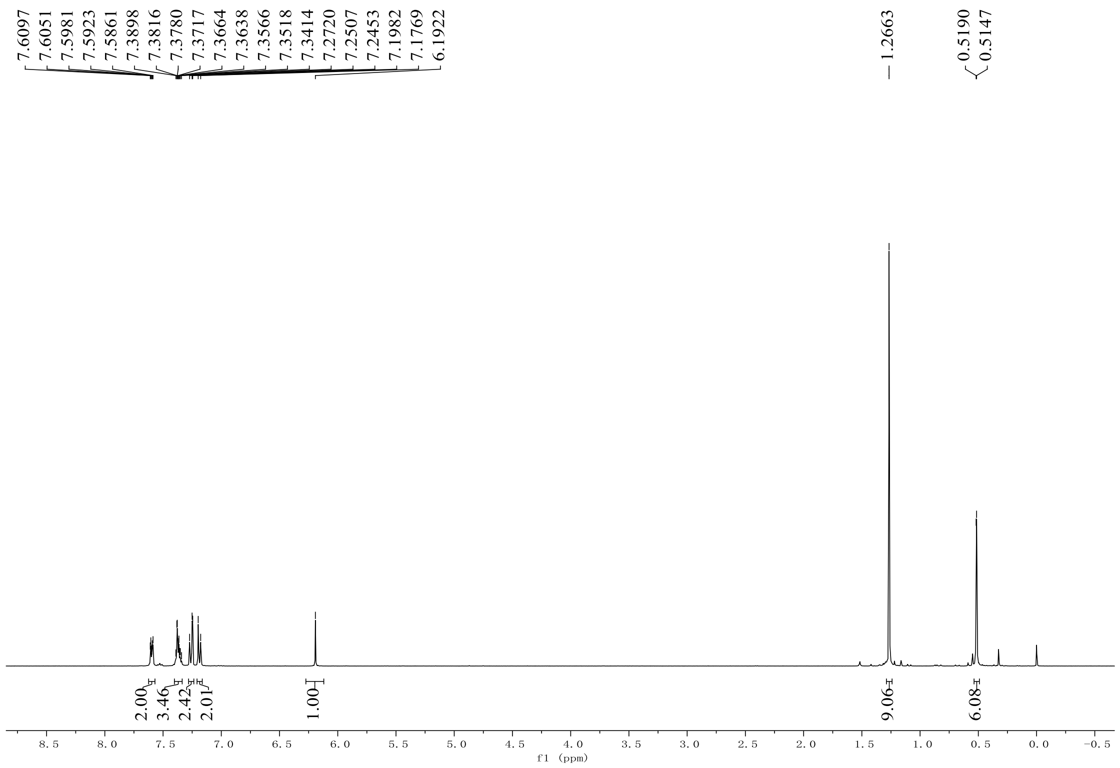
7.6097
7.6051
7.5981
7.5923
7.5861
7.3898
7.3816
7.3780
7.3717
7.3664
7.3638
7.3566
7.3518
7.3414
7.2720
7.2507
7.2453
7.1982
7.1769
6.1922
1.2663
0.5190
0.5147
2.00
3.46
2.42
2.01
1.00
9.06
6.08
f1 (ppm)

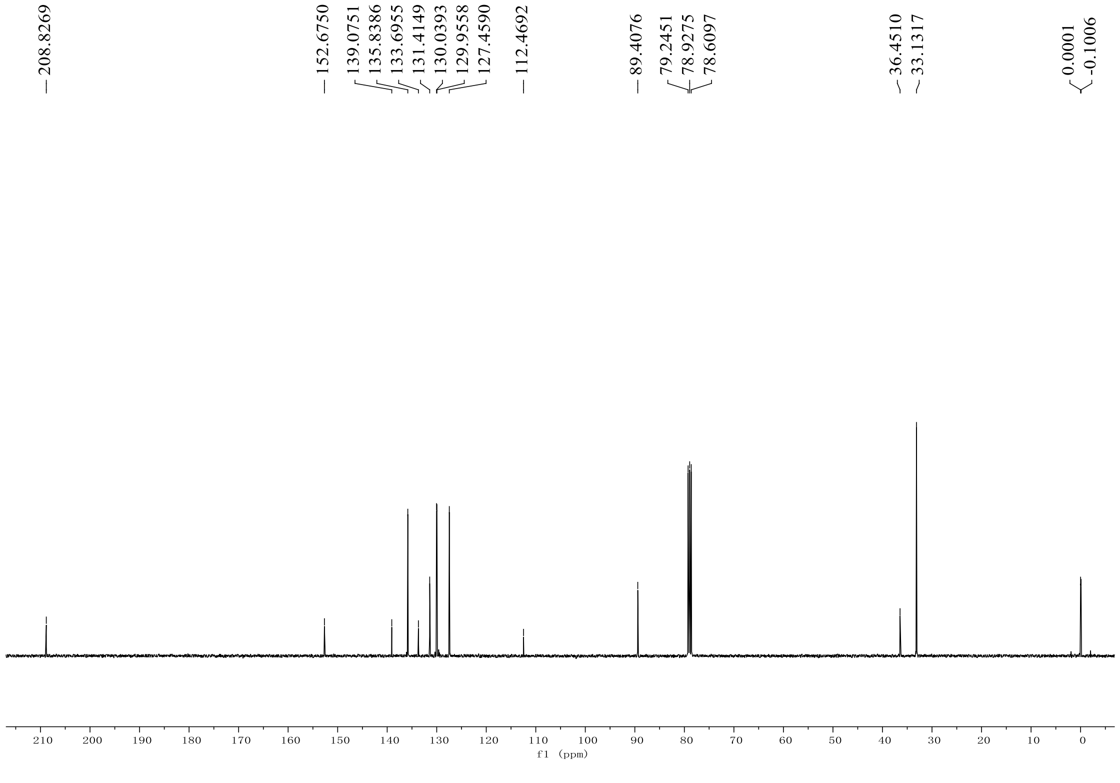
208.8269
152.6750
139.0751
135.8386
133.6955
131.4149
130.0393
129.9558
127.4590
112.4692
89.4076
79.2451
78.9275
78.6097
36.4510
33.1317
0.0001
-0.1006
f1 (ppm)

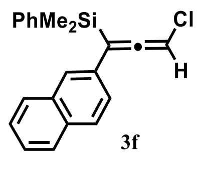
PhMe2Si
Cl
H

3f

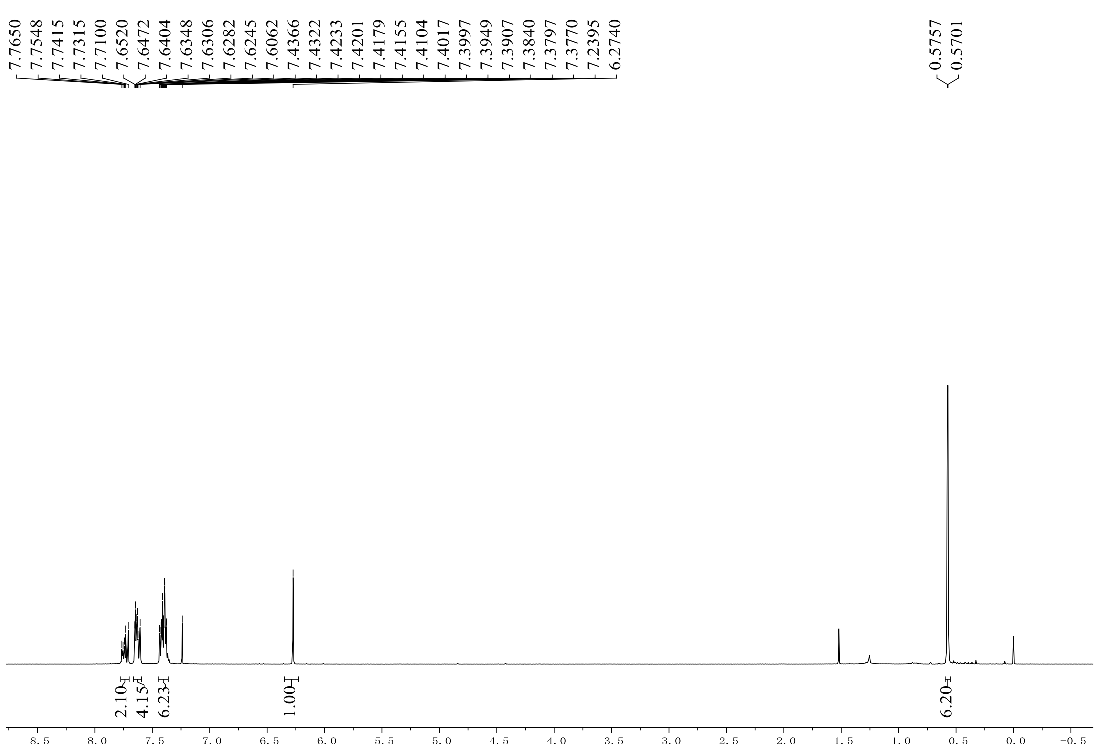
7.7650 7.7548 7.7415 7.7315 7.7100 7.6520 7.6472 7.6404 7.6348 7.6306 7.6282 7.6245 7.6062 7.4366 7.4322 7.4233 7.4201 7.4179 7.4155 7.4104 7.4017 7.3997 7.3949 7.3907 7.3840 7.3797 7.3770 7.2395 6.2740
0.5757 0.5701
2.10 4.15 6.23 1.00 6.20
f1 (ppm)

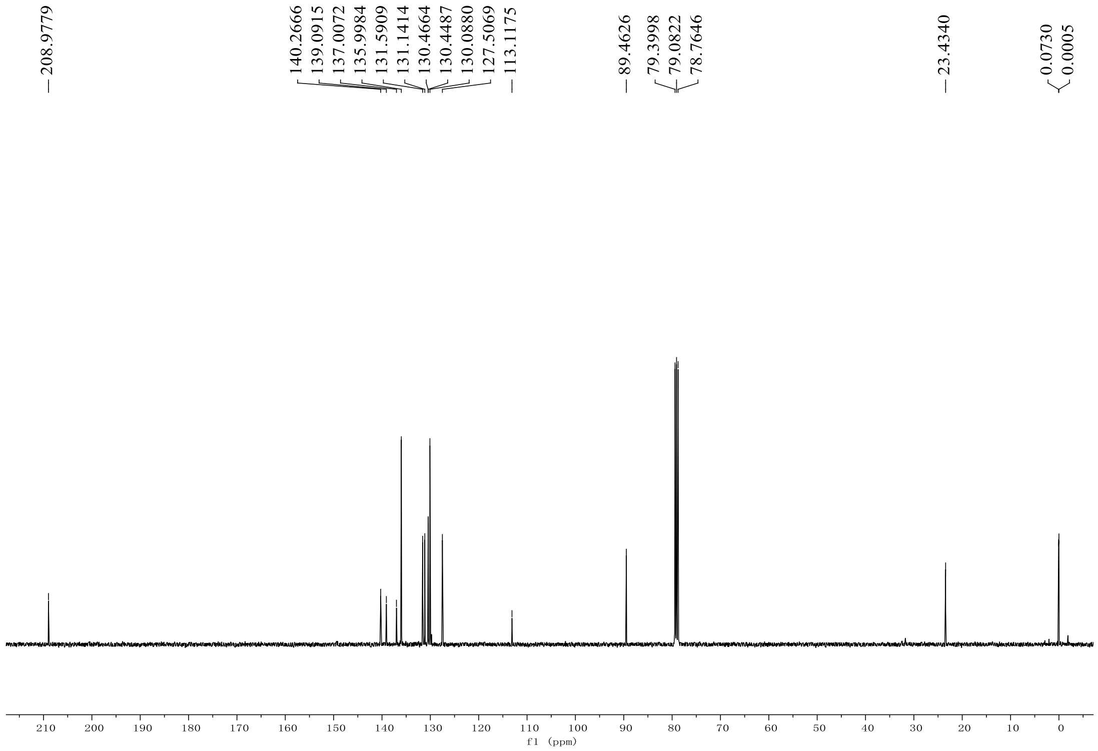
208.9779
140.2666 139.0915 137.0072 135.9984 131.5909 131.1414 130.4664 130.4487 130.0880 127.5069 113.1175
89.4626 79.3998 79.0822 78.7646
23.4340
0.0730 0.0005
f1 (ppm)

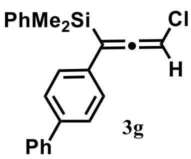
PhMe2Si
Cl
H
Ph

3g

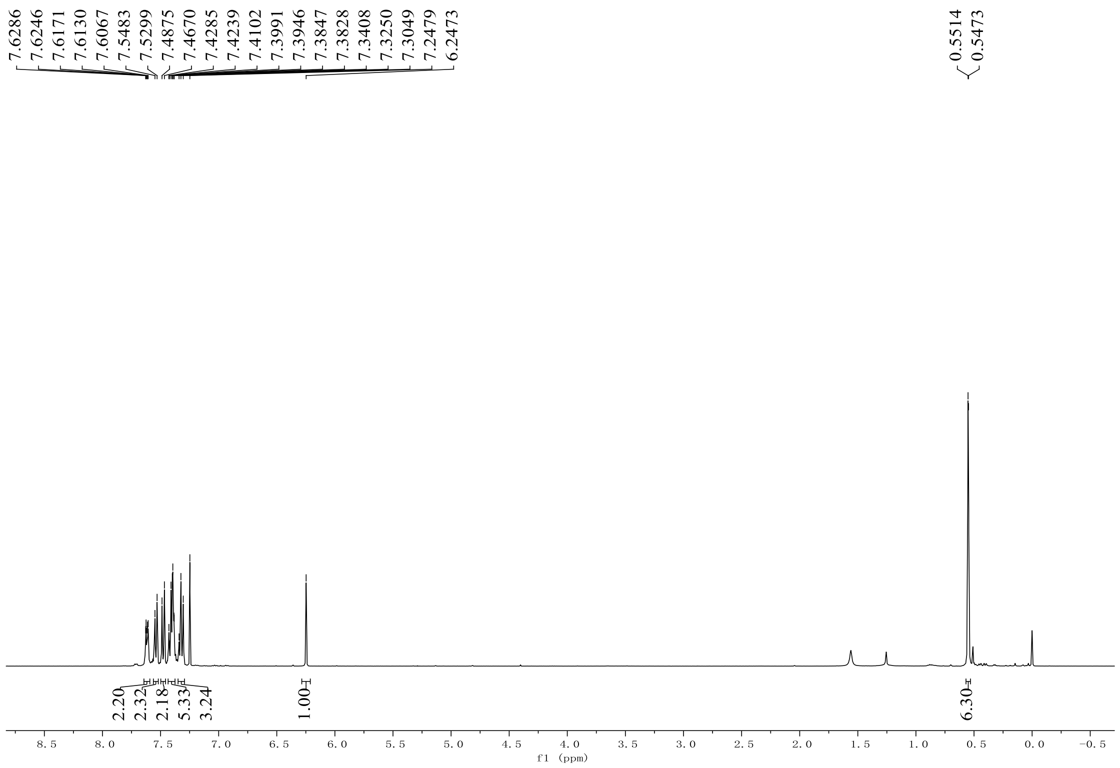
7.6286
7.6246
7.6171
7.6130
7.6067
7.5483
7.5299
7.4875
7.4670
7.4285
7.4239
7.4102
7.3991
7.3946
7.3847
7.3828
7.3408
7.3250
7.3049
7.2479
6.2473
0.5514
0.5473
2.20
2.32
2.18
5.33
3.24
1.00
6.30
f1 (ppm)

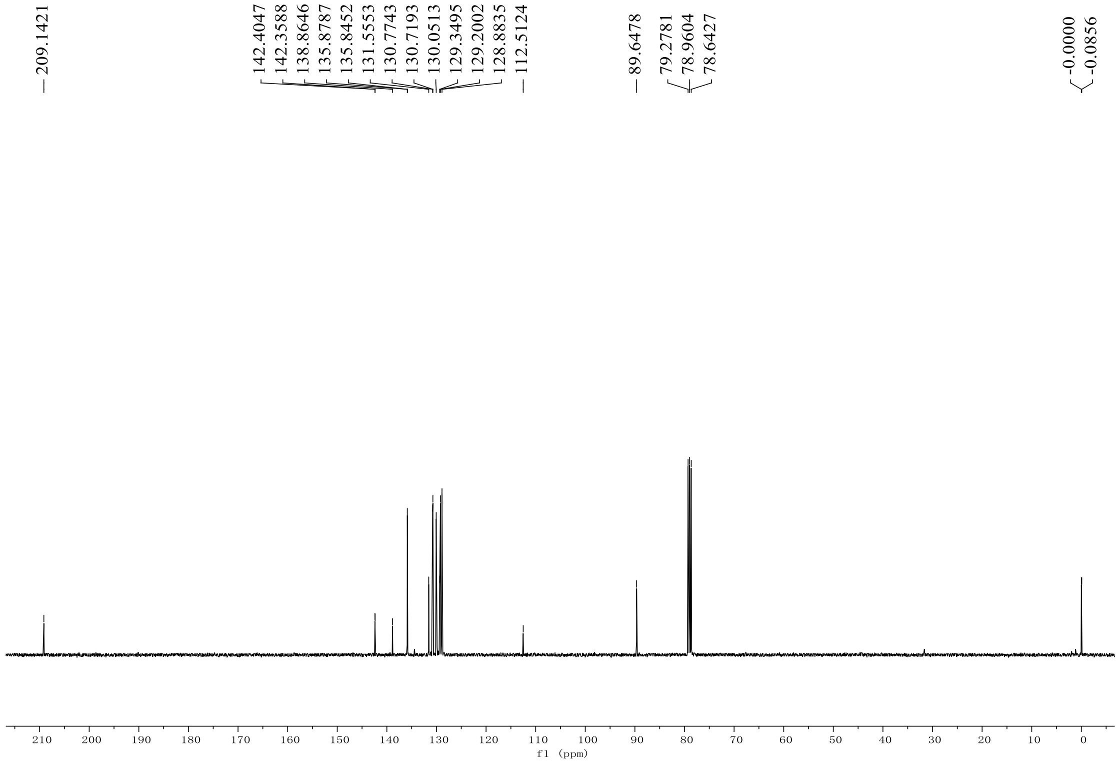
209.1421
142.4047
142.3588
138.8646
135.8787
135.8452
131.5553
130.7743
130.7193
130.0513
129.3495
129.2002
128.8835
112.5124
89.6478
79.2781
78.9604
78.6427
0.0000
-0.0856
f1 (ppm)

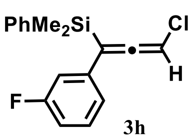
PhMe$_2$Si
Cl
H
F

3h

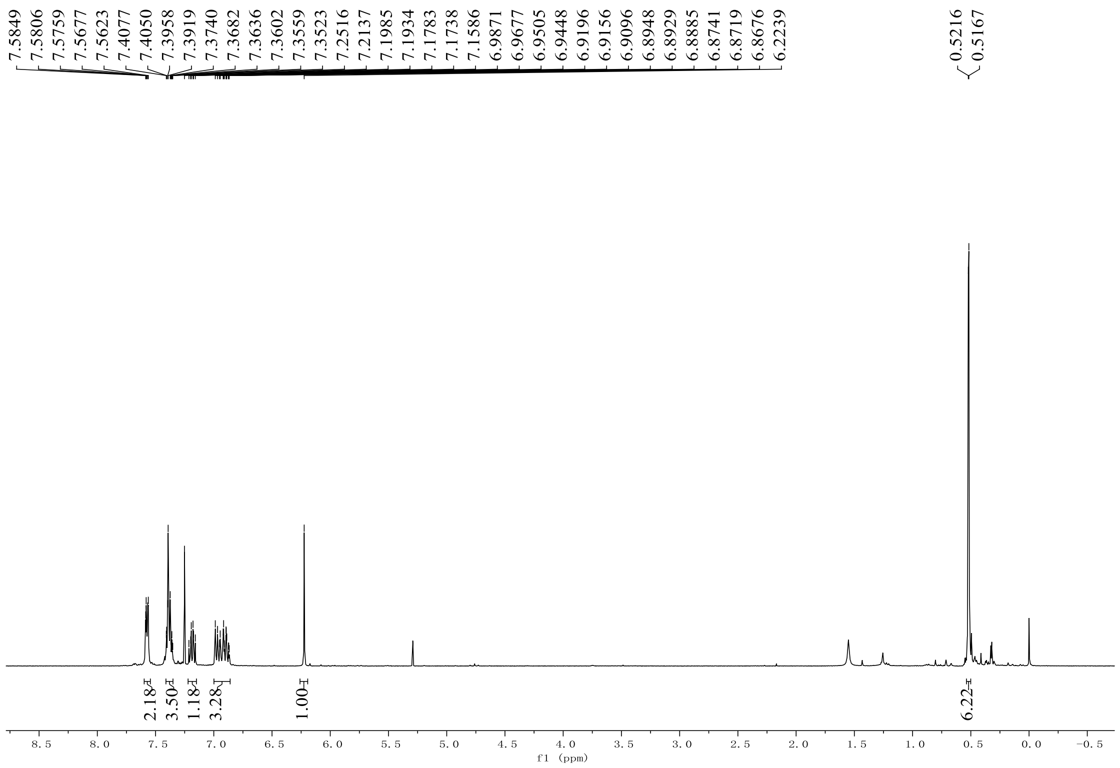
7.5849
7.5806
7.5759
7.5677
7.5623
7.4077
7.4050
7.3958
7.3919
7.3740
7.3682
7.3636
7.3602
7.3559
7.3523
7.2516
7.2137
7.1985
7.1934
7.1783
7.1738
7.1586
6.9871
6.9677
6.9505
6.9448
6.9196
6.9156
6.9096
6.8948
6.8929
6.8885
6.8741
6.8719
6.8676
6.2239
0.5216
0.5167
2.18
3.50
1.18
3.28
1.00
6.22
8.5 8.0 7.5 7.0 6.5 6.0 5.5 5.0 4.5 4.0 3.5 3.0 2.5 2.0 1.5 1.0 0.5 0.0 -0.5
f1 (ppm)

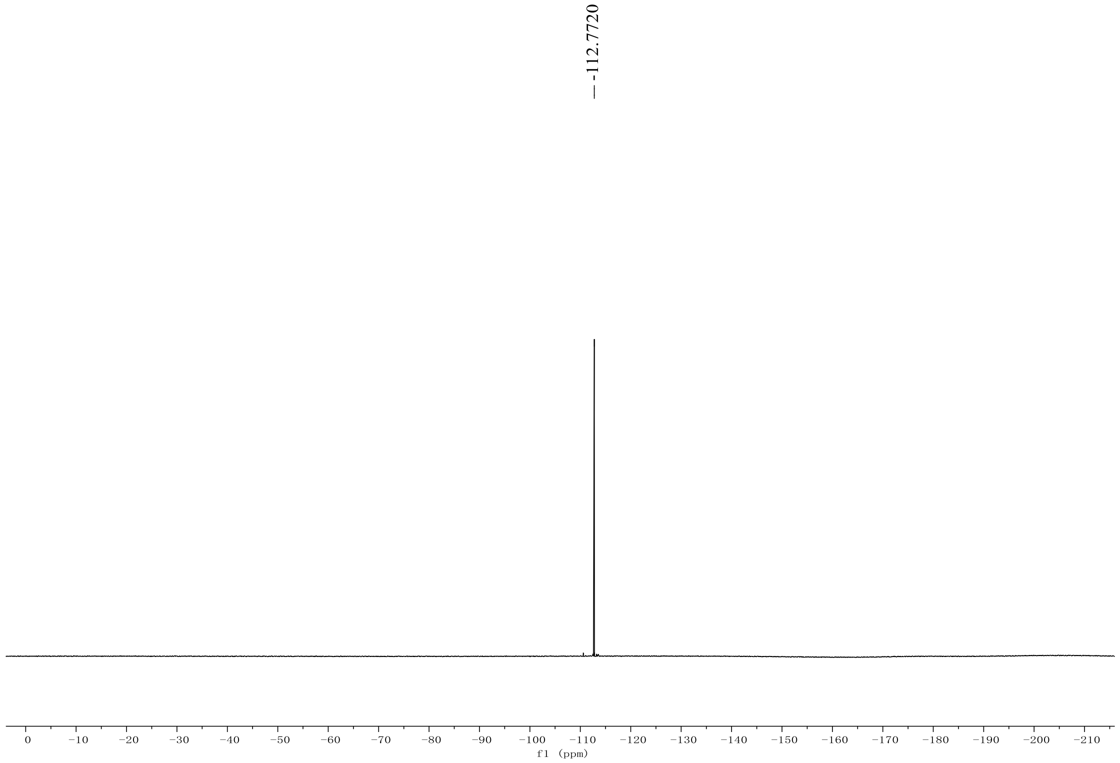
-112.7720
0 -10 -20 -30 -40 -50 -60 -70 -80 -90 -100 -110 -120 -130 -140 -150 -160 -170 -180 -190 -200 -210
f1 (ppm)

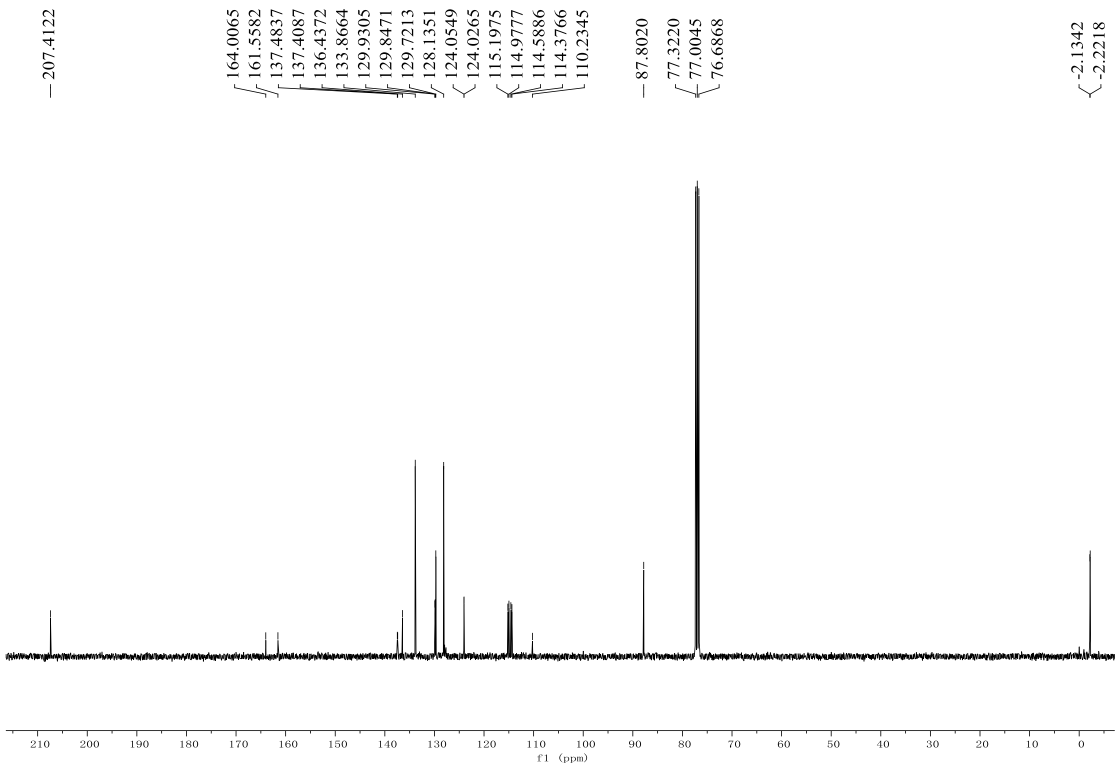
207.4122
164.0065
161.5582
137.4837
137.4087
136.4372
133.8664
129.9305
129.8471
129.7213
128.1351
124.0549
124.0265
115.1975
114.9777
114.5886
114.3766
110.2345
87.8020
77.3220
77.0045
76.6868
-2.1342
-2.2218
210 200 190 180 170 160 150 140 130 120 110 100 90 80 70 60 50 40 30 20 10 0
f1 (ppm)

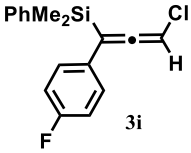
PhMe2Si
Cl
H
F

3i

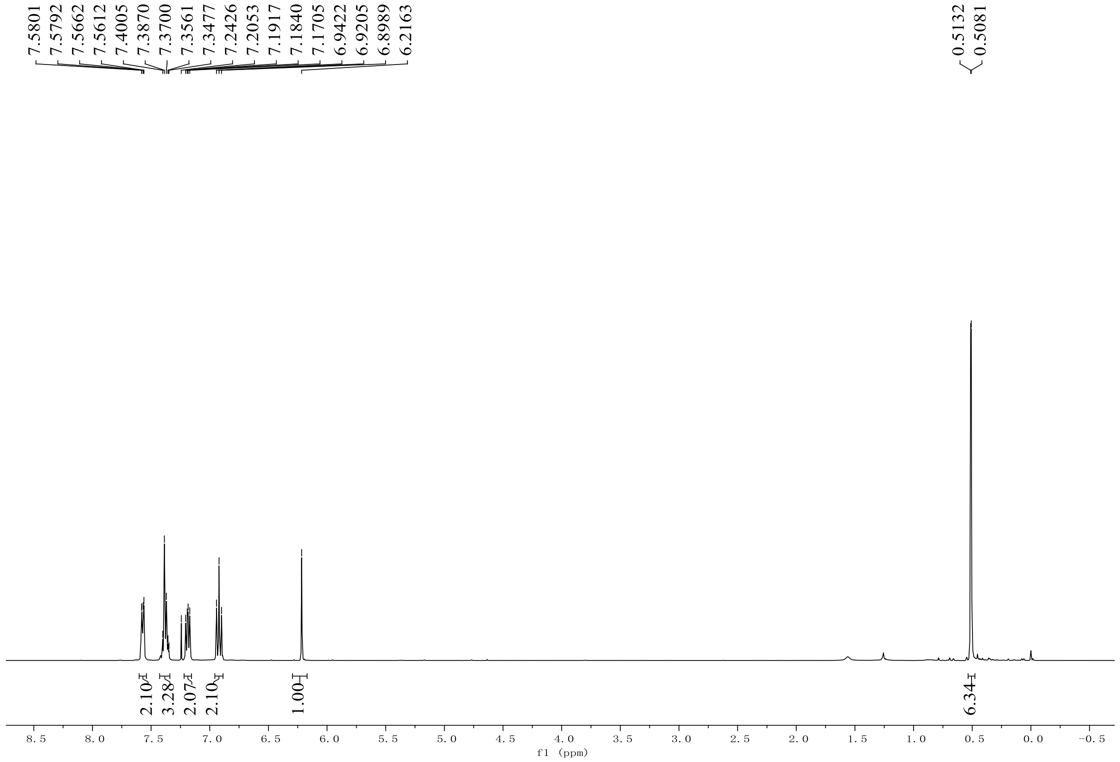
7.5801
7.5792
7.5662
7.5612
7.4005
7.3870
7.3700
7.3561
7.3477
7.2426
7.2053
7.1917
7.1840
7.1705
6.9422
6.9205
6.8989
6.2163
0.5132
0.5081
2.10
3.28
2.07
2.10
1.00
6.34
8.5 8.0 7.5 7.0 6.5 6.0 5.5 5.0 4.5 4.0 3.5 3.0 2.5 2.0 1.5 1.0 0.5 0.0 -0.5
f1 (ppm)

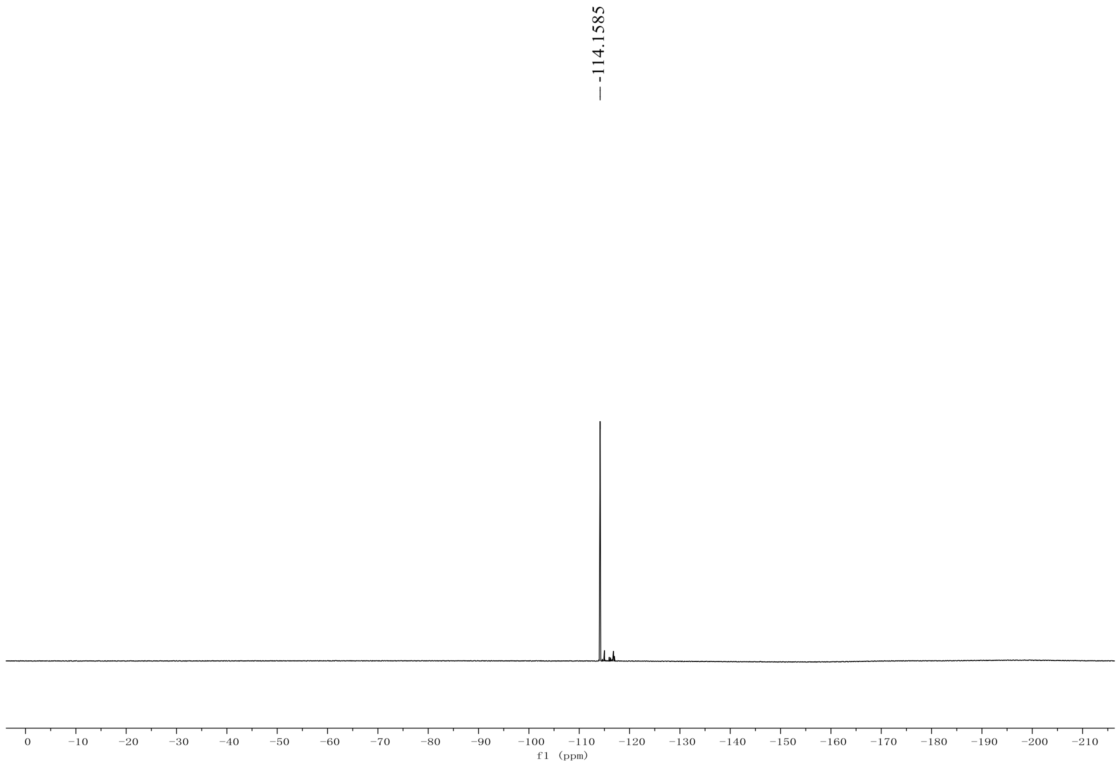
-114.1585
0
-10
-20
-30
-40
-50
-60
-70
-80
-90
-100
-110
-120
-130
-140
-150
-160
-170
-180
-190
-200
-210
f1 (ppm)

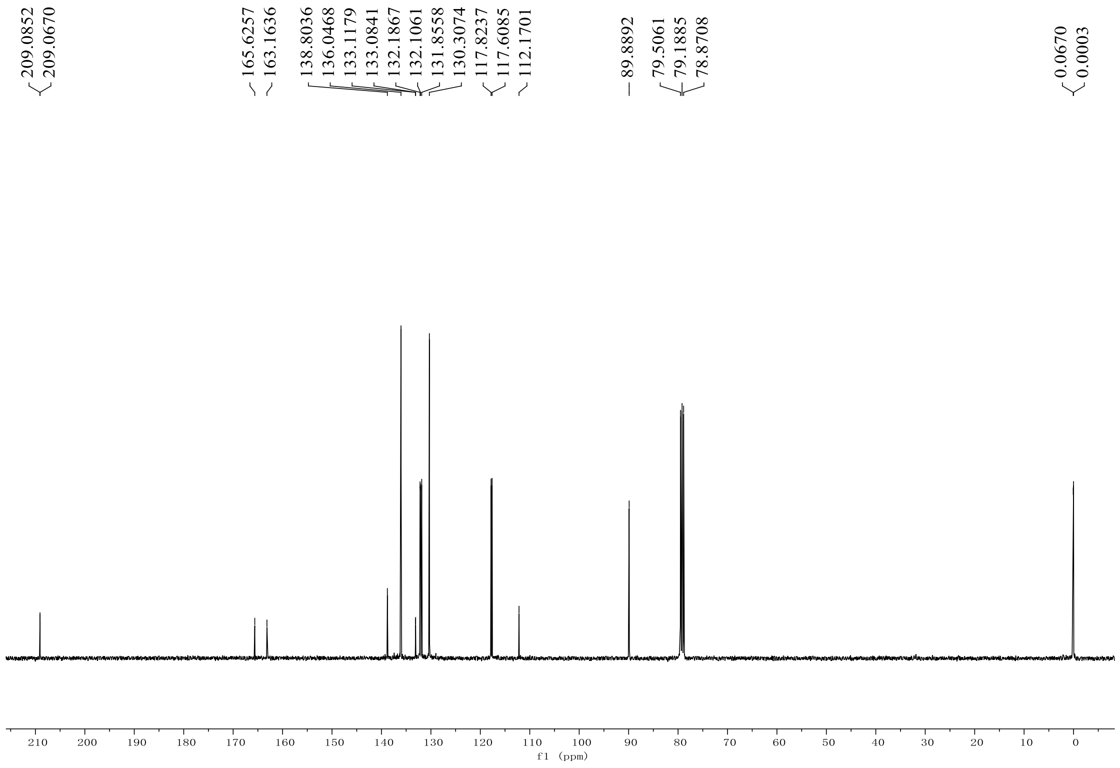
209.0852
209.0670
165.6257
163.1636
138.8036
136.0468
133.1179
133.0841
132.1867
132.1061
131.8558
130.3074
117.8237
117.6085
112.1701
89.8892
79.5061
79.1885
78.8708
0.0670
0.0003
210
200
190
180
170
160
150
140
130
120
110
100
90
80
70
60
50
40
30
20
10
0
f1 (ppm)

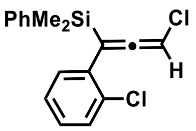
PhMe$_2$Si
Cl
H
Cl

3j

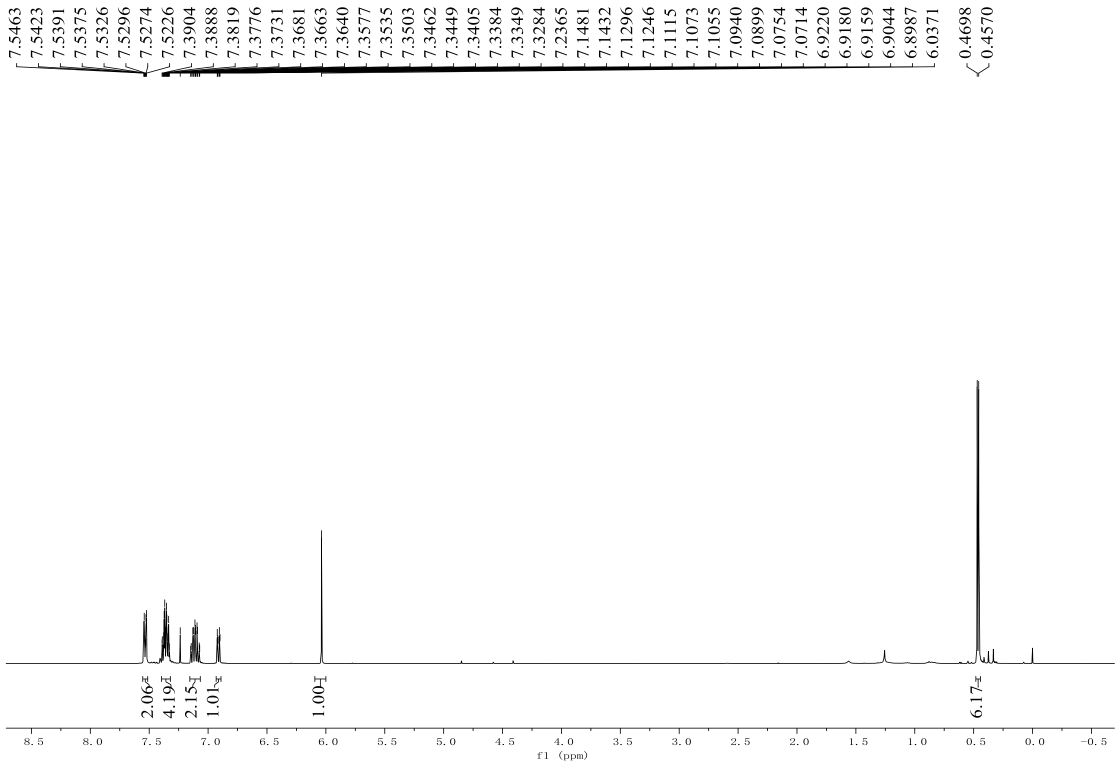
7.5463
7.5423
7.5391
7.5375
7.5326
7.5296
7.5274
7.5226
7.3904
7.3888
7.3819
7.3776
7.3731
7.3681
7.3663
7.3640
7.3577
7.3535
7.3503
7.3462
7.3449
7.3405
7.3384
7.3349
7.3284
7.2365
7.1481
7.1432
7.1296
7.1246
7.1115
7.1073
7.1055
7.0940
7.0899
7.0754
7.0714
6.9220
6.9180
6.9159
6.9044
6.8987
6.0371
0.4698
0.4570
2.06
4.19
2.15
1.01
1.00
6.17
f1 (ppm)

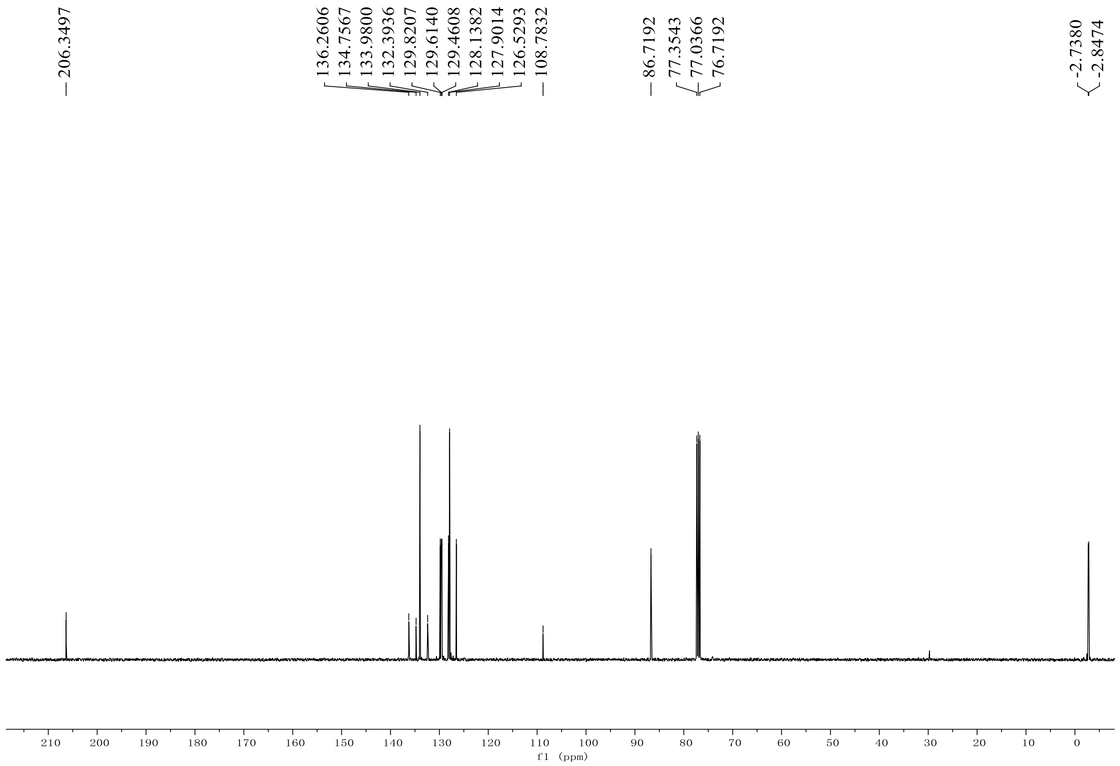
206.3497
136.2606
134.7567
133.9800
132.3936
129.8207
129.6140
129.4608
128.1382
127.9014
126.5293
108.7832
86.7192
77.3543
77.0366
76.7192
-2.7380
-2.8474
f1 (ppm)

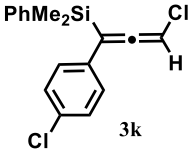
PhMe2Si
Cl
H
Cl

3k

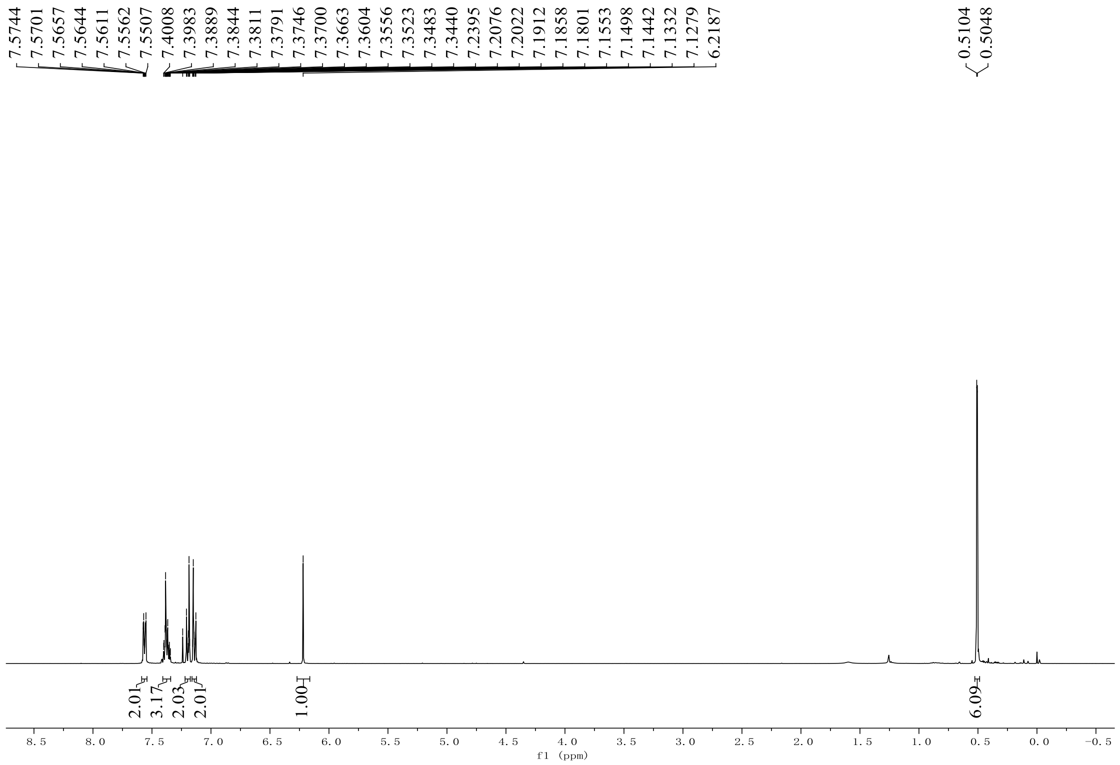
7.5744
7.5701
7.5657
7.5644
7.5611
7.5562
7.5507
7.4008
7.3983
7.3889
7.3844
7.3811
7.3791
7.3746
7.3700
7.3663
7.3604
7.3556
7.3523
7.3483
7.3440
7.2395
7.2076
7.2022
7.1912
7.1858
7.1801
7.1553
7.1498
7.1442
7.1332
7.1279
6.2187
0.5104
0.5048
2.01
3.17
2.03
2.01
1.00
6.09
f1 (ppm)

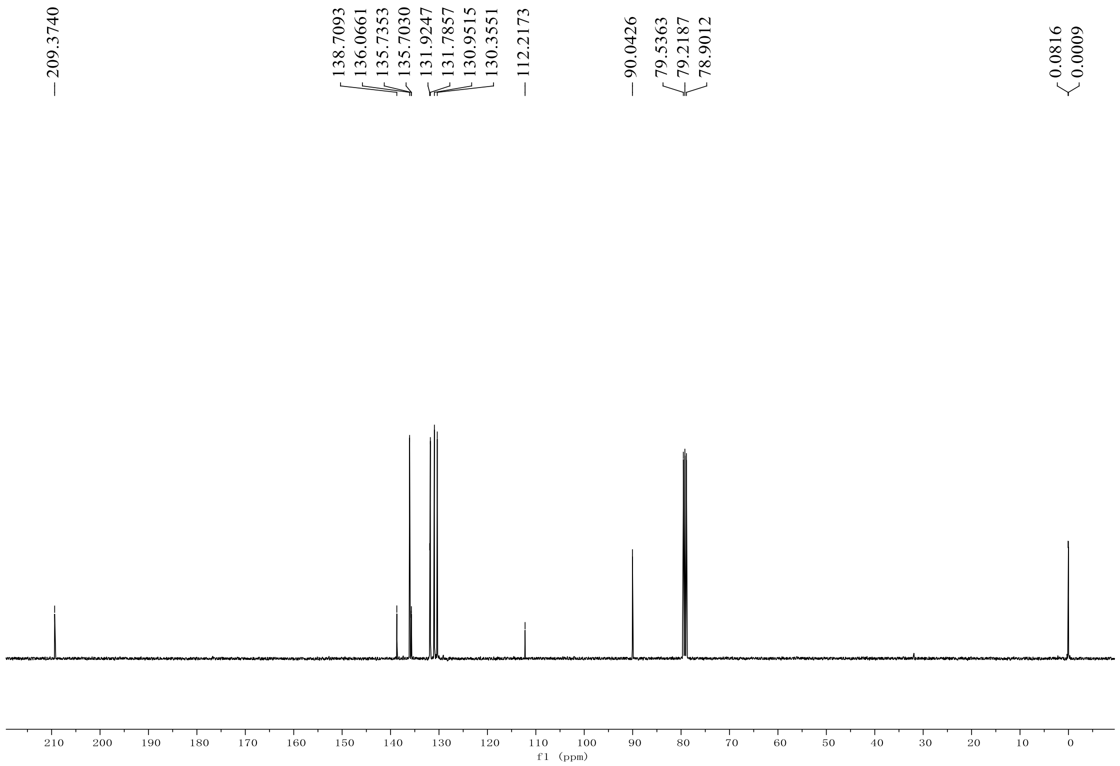
209.3740
138.7093
136.0661
135.7353
135.7030
131.9247
131.7857
130.9515
130.3551
112.2173
90.0426
79.5363
79.2187
78.9012
0.0816
0.0009
f1 (ppm)

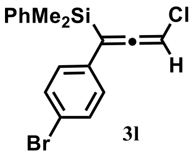
PhMe2Si
Cl
H
Br

3l

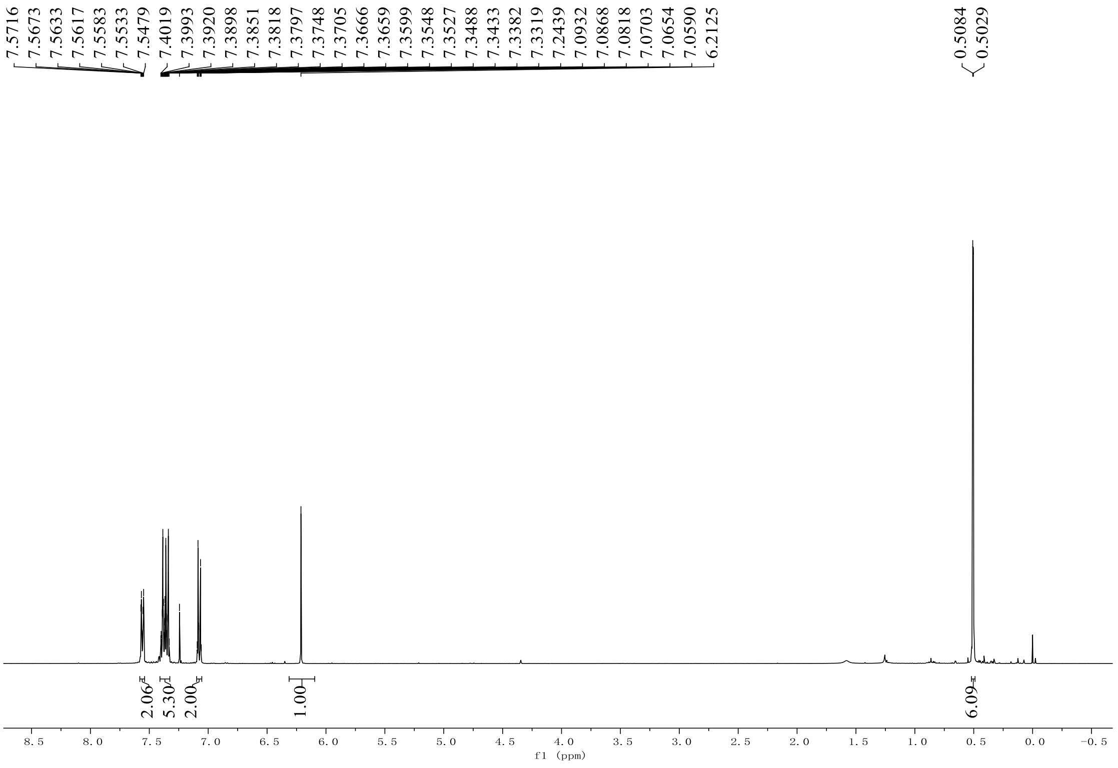
7.5716
7.5673
7.5633
7.5617
7.5583
7.5533
7.5479
7.4019
7.3993
7.3920
7.3898
7.3851
7.3818
7.3797
7.3748
7.3705
7.3666
7.3659
7.3599
7.3548
7.3527
7.3488
7.3433
7.3382
7.3319
7.2439
7.0932
7.0868
7.0818
7.0703
7.0654
7.0590
6.2125
0.5084
0.5029
2.06
5.30
2.00
1.00
6.09
8.5 8.0 7.5 7.0 6.5 6.0 5.5 5.0 4.5 4.0 3.5 3.0 2.5 2.0 1.5 1.0 0.5 0.0 -0.5
f1 (ppm)

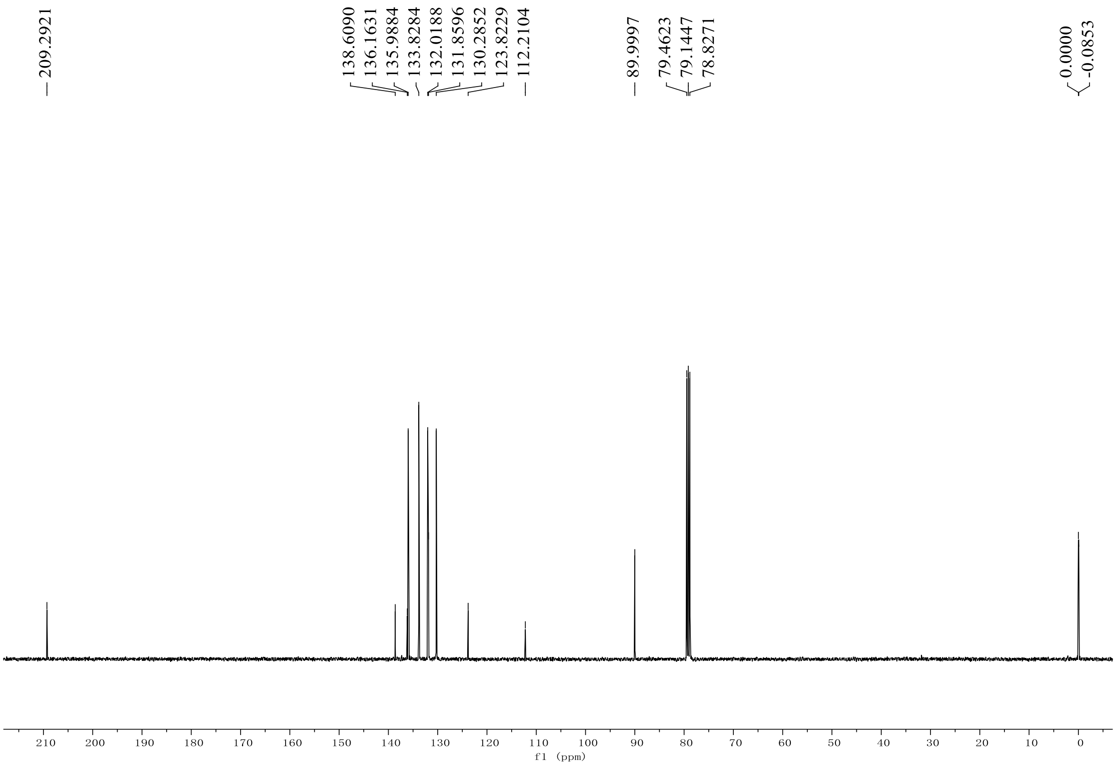
209.2921
138.6090
136.1631
135.9884
133.8284
132.0188
131.8596
130.2852
123.8229
112.2104
89.9997
79.4623
79.1447
78.8271
0.0000
-0.0853
210 200 190 180 170 160 150 140 130 120 110 100 90 80 70 60 50 40 30 20 10 0
f1 (ppm)

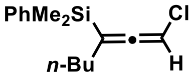
PhMe2Si
Cl
n-Bu
H

3m

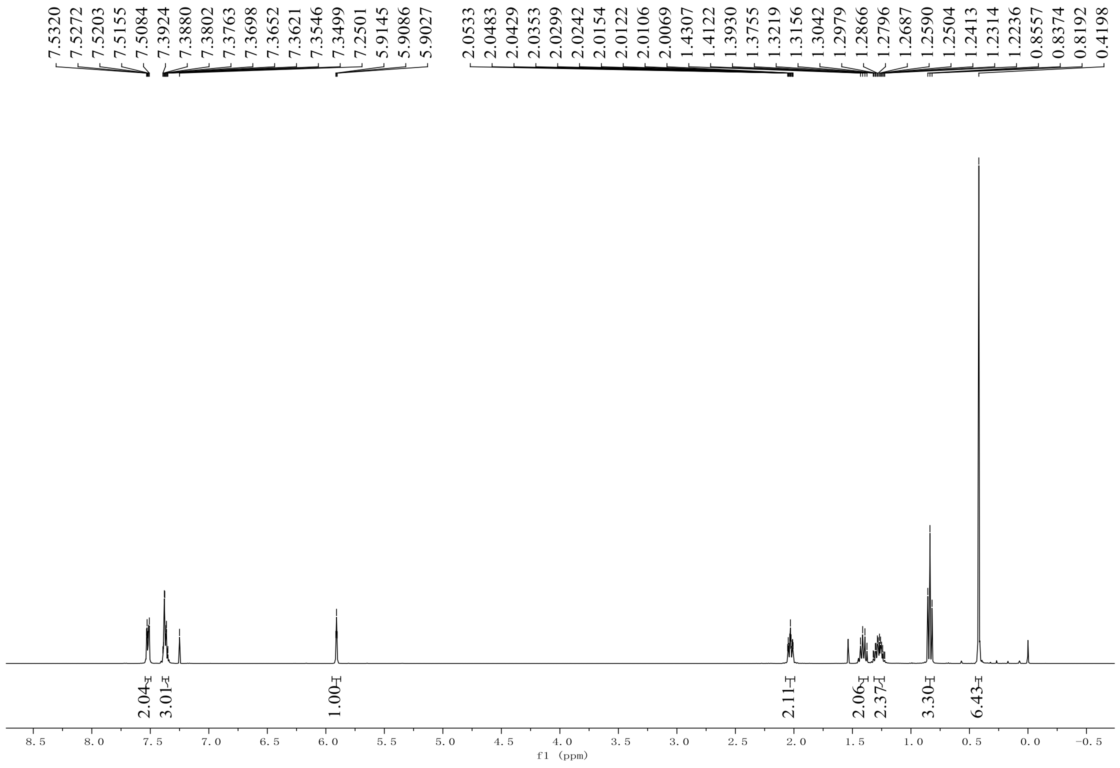
7.5320
7.5272
7.5203
7.5155
7.5084
7.3924
7.3880
7.3802
7.3763
7.3698
7.3652
7.3621
7.3546
7.3499
7.2501
5.9145
5.9086
5.9027
2.0533
2.0483
2.0429
2.0353
2.0299
2.0242
2.0154
2.0122
2.0106
2.0069
1.4307
1.4122
1.3930
1.3755
1.3219
1.3156
1.3042
1.2979
1.2866
1.2796
1.2687
1.2590
1.2504
1.2413
1.2314
1.2236
0.8557
0.8374
0.8192
0.4198
2.04
3.01
1.00
2.11
2.06
2.37
3.30
6.43
f1 (ppm)

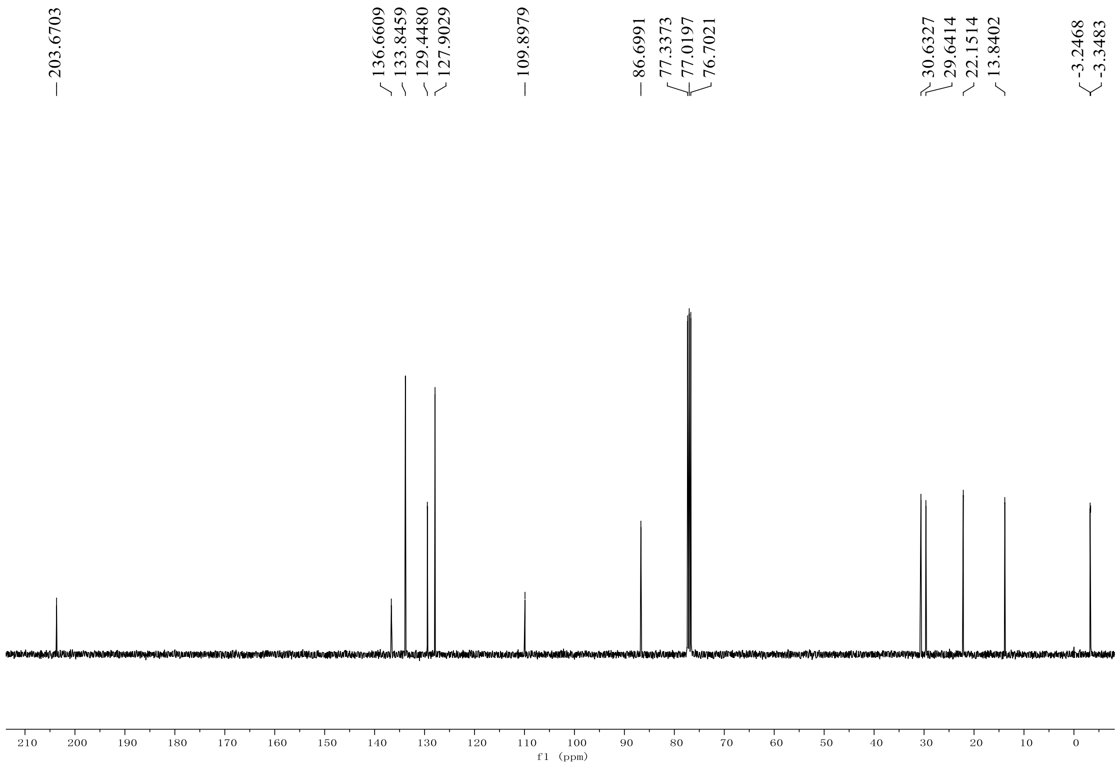
203.6703
136.6609
133.8459
129.4480
127.9029
109.8979
86.6991
77.3373
77.0197
76.7021
30.6327
29.6414
22.1514
13.8402
-3.2468
-3.3483
f1 (ppm)

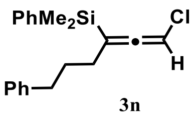

3n

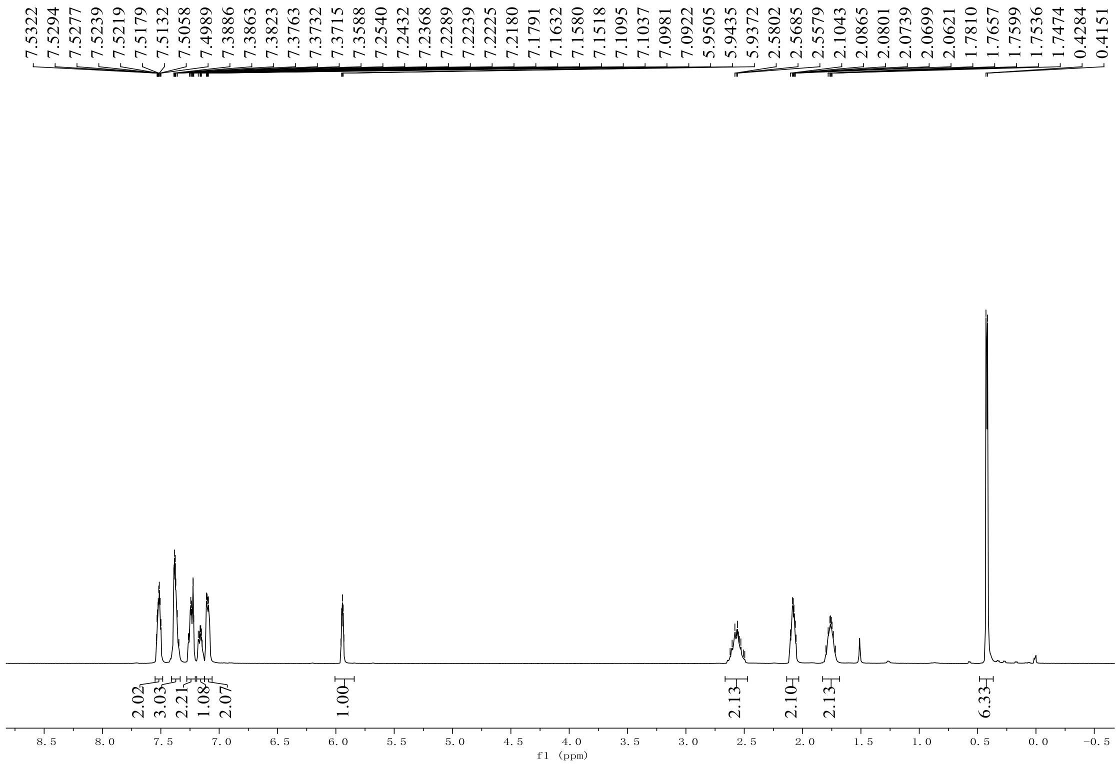

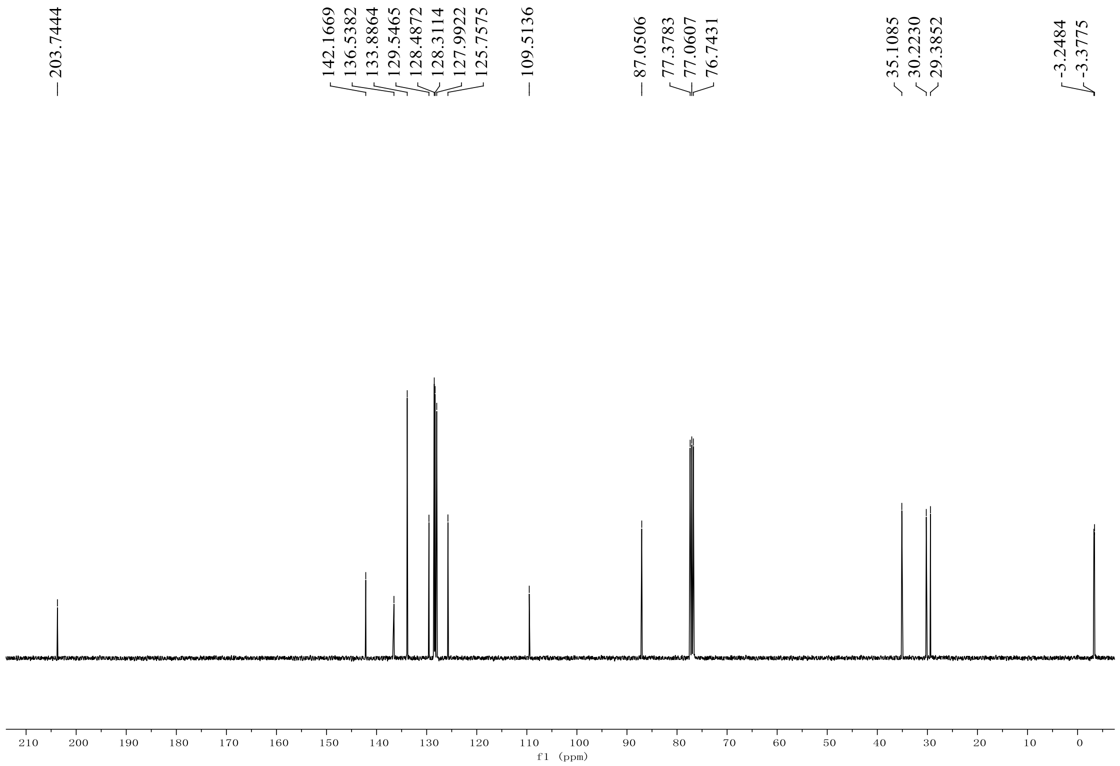

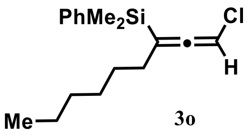
PhMe2Si
Cl
H
Me

3o

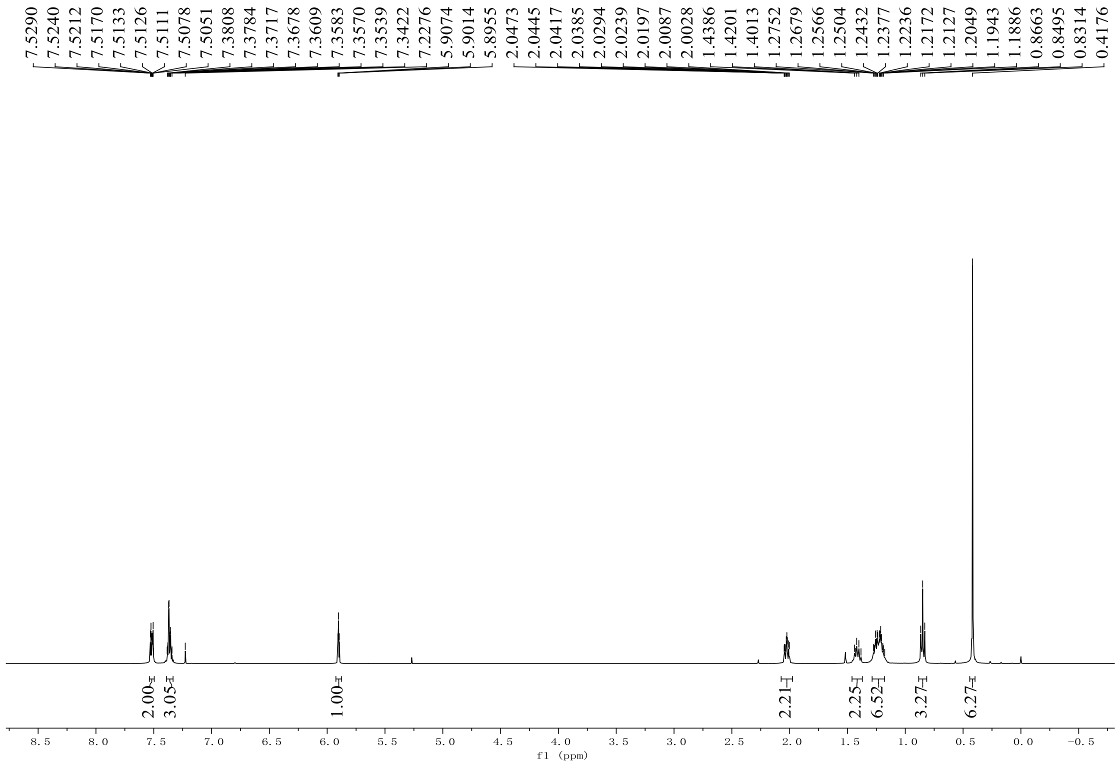
7.5290
7.5240
7.5212
7.5170
7.5133
7.5126
7.5111
7.5078
7.5051
7.3808
7.3784
7.3717
7.3678
7.3609
7.3583
7.3570
7.3539
7.3422
7.2276
5.9074
5.9014
5.8955
2.0473
2.0445
2.0417
2.0385
2.0294
2.0239
2.0197
2.0087
2.0028
1.4386
1.4201
1.4013
1.2752
1.2679
1.2566
1.2504
1.2432
1.2377
1.2236
1.2172
1.2127
1.2049
1.1943
1.1886
0.8663
0.8495
0.8314
0.4176
2.00
3.05
1.00
2.21
2.25
6.52
3.27
6.27
f1 (ppm)

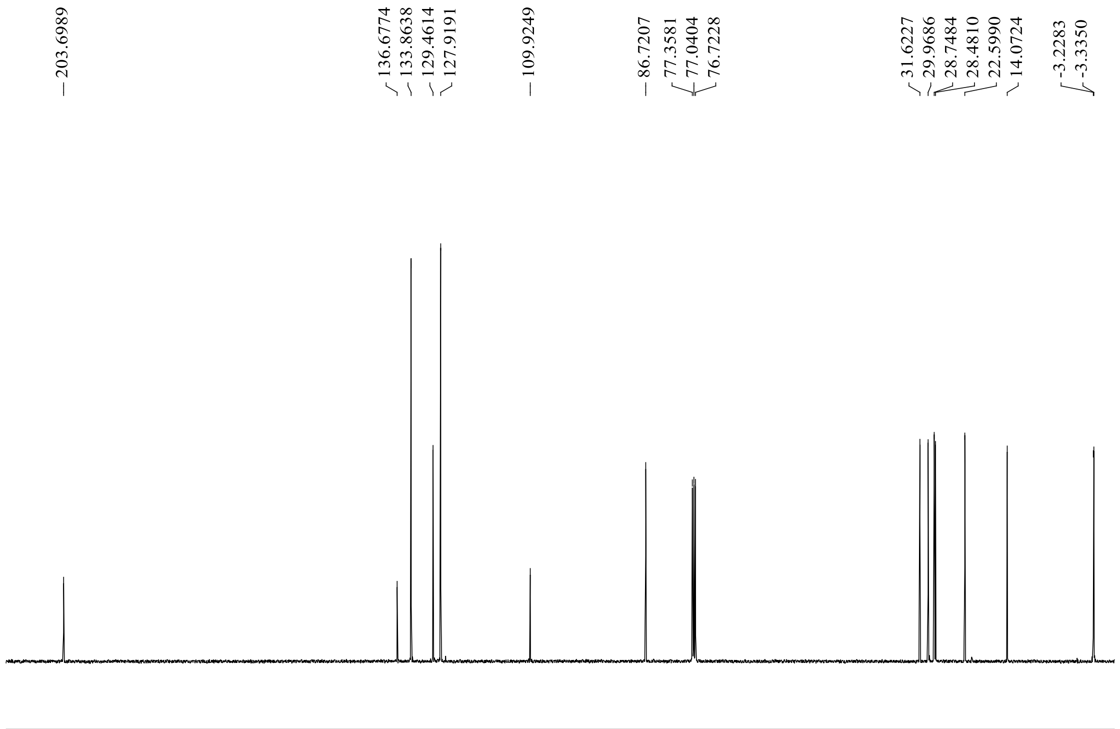
203.6989
136.6774
133.8638
129.4614
127.9191
109.9249
86.7207
77.3581
77.0404
76.7228
31.6227
29.9686
28.7484
28.4810
22.5990
14.0724
-3.2283
-3.3350
f1 (ppm)

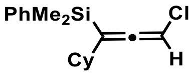
PhMe2Si
Cl
Cy
H

3p

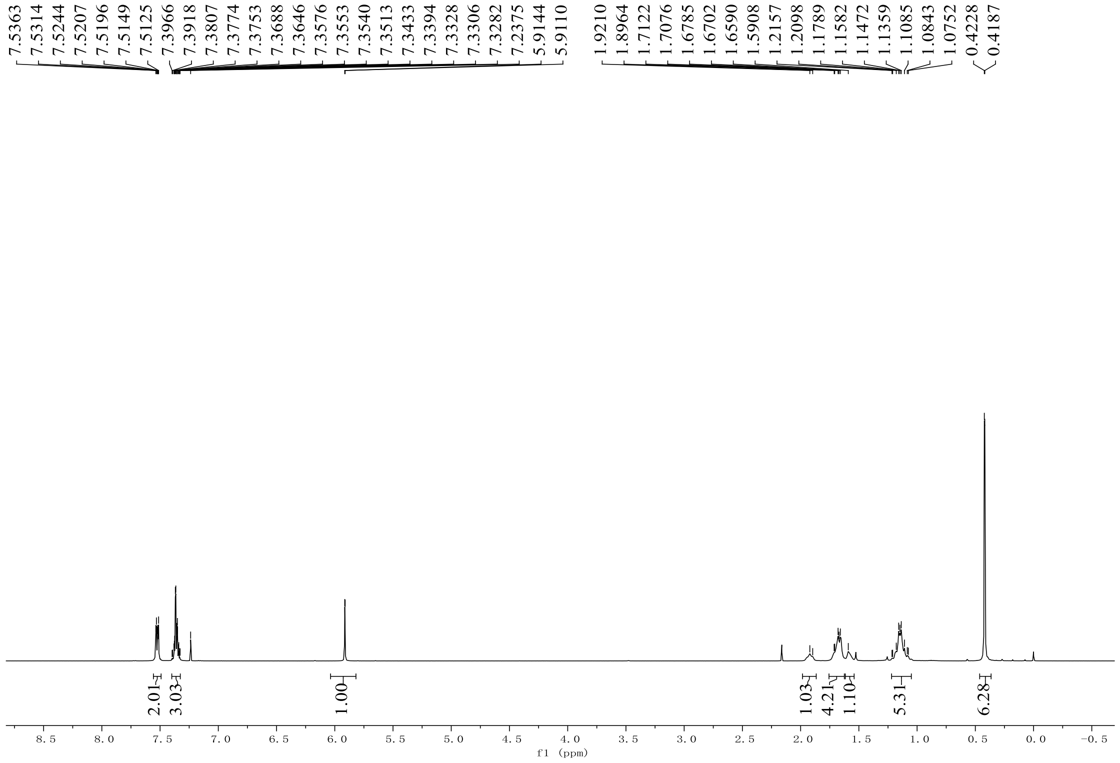
7.5363
7.5314
7.5244
7.5207
7.5196
7.5149
7.5125
7.3966
7.3918
7.3807
7.3774
7.3753
7.3688
7.3646
7.3576
7.3553
7.3540
7.3513
7.3433
7.3394
7.3328
7.3306
7.3282
7.2375
5.9144
5.9110
1.9210
1.8964
1.7122
1.7076
1.6785
1.6702
1.6590
1.5908
1.2157
1.2098
1.1789
1.1582
1.1472
1.1359
1.1085
1.0843
1.0752
0.4228
0.4187
2.01
3.03
1.00
1.03
4.21
1.10
5.31
6.28
f1 (ppm)

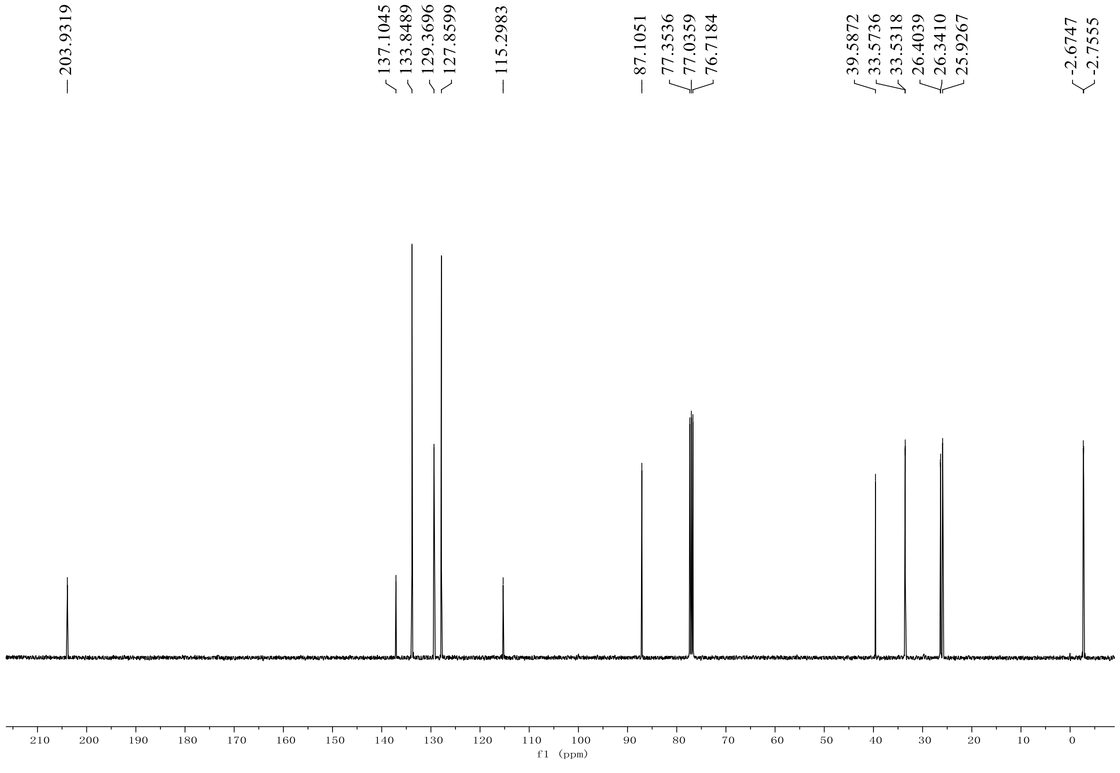
203.9319
137.1045
133.8489
129.3696
127.8599
115.2983
87.1051
77.3536
77.0359
76.7184
39.5872
33.5736
33.5318
26.4039
26.3410
25.9267
-2.6747
-2.7555
f1 (ppm)

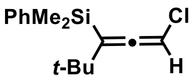
PhMe2Si
Cl
t-Bu
H

3q

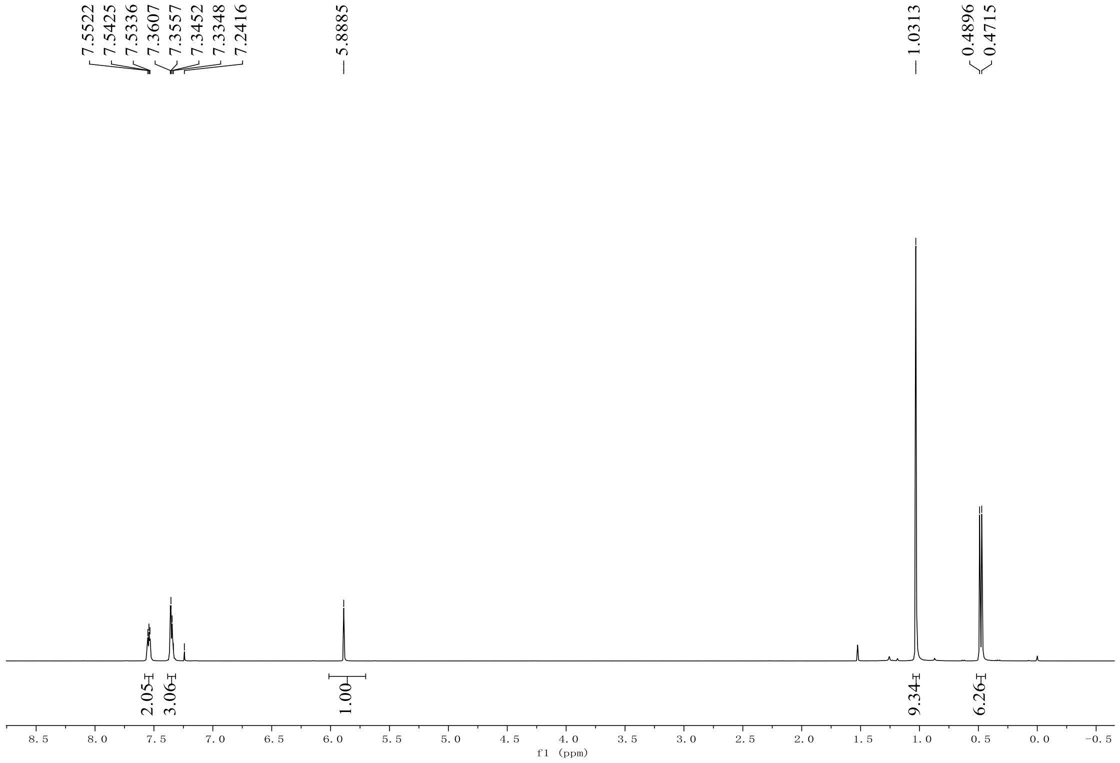
7.5522
7.5425
7.5336
7.3607
7.3557
7.3452
7.3348
7.2416
5.8885
1.0313
0.4896
0.4715
2.05
3.06
1.00
9.34
6.26
8.5 8.0 7.5 7.0 6.5 6.0 5.5 5.0 4.5 4.0 3.5 3.0 2.5 2.0 1.5 1.0 0.5 0.0 -0.5
f1 (ppm)

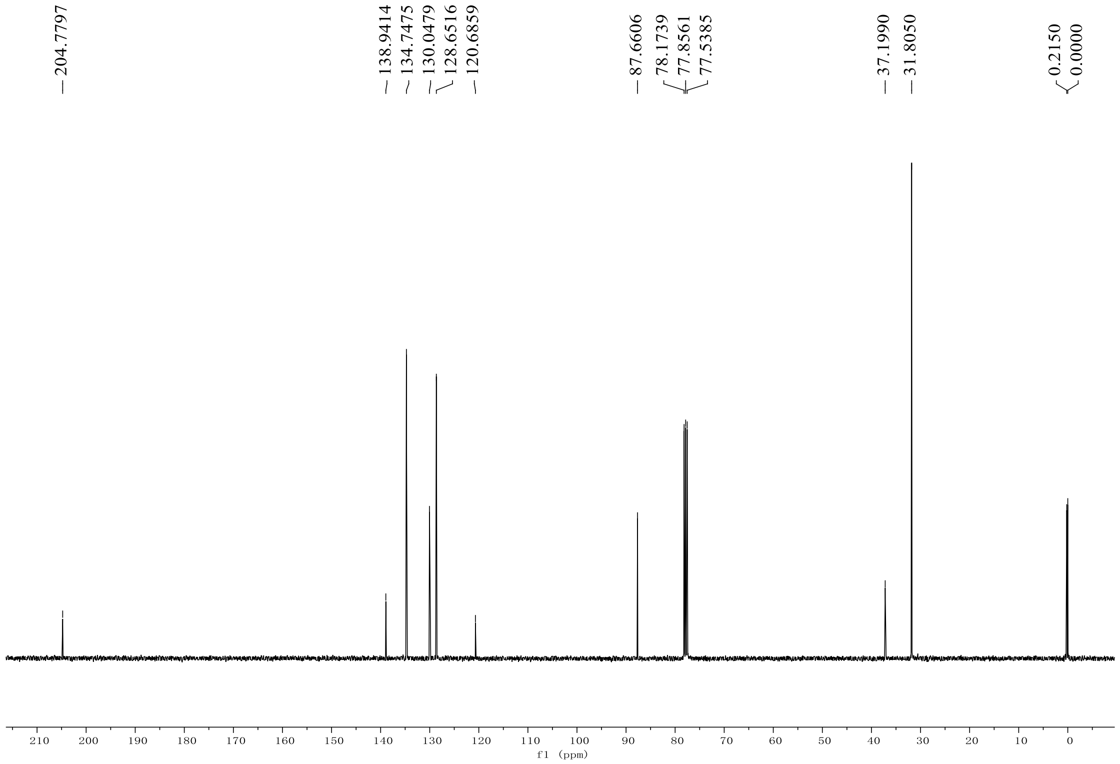
204.7797
138.9414
134.7475
130.0479
128.6516
120.6859
87.6606
78.1739
77.8561
77.5385
37.1990
31.8050
0.2150
0.0000
210 200 190 180 170 160 150 140 130 120 110 100 90 80 70 60 50 40 30 20 10 0
f1 (ppm)

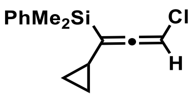
PhMe2Si
Cl
H

3r

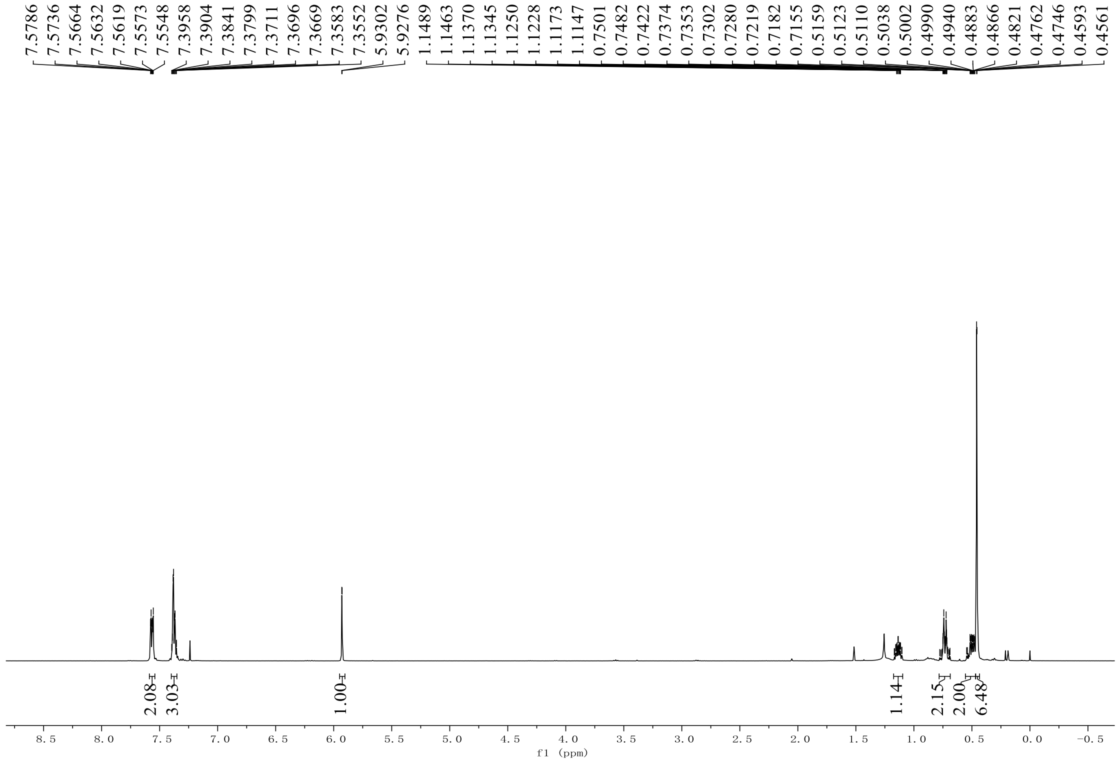
7.5786
7.5736
7.5664
7.5632
7.5619
7.5573
7.5548
7.3958
7.3904
7.3841
7.3799
7.3711
7.3696
7.3669
7.3583
7.3552
5.9302
5.9276
1.1489
1.1463
1.1370
1.1345
1.1250
1.1228
1.1173
1.1147
0.7501
0.7482
0.7422
0.7374
0.7353
0.7302
0.7280
0.7219
0.7182
0.7155
0.5159
0.5123
0.5110
0.5038
0.5002
0.4990
0.4940
0.4883
0.4866
0.4821
0.4762
0.4746
0.4593
0.4561
2.08
3.03
1.00
1.14
2.15
2.00
6.48
8.5
8.0
7.5
7.0
6.5
6.0
5.5
5.0
4.5
4.0
3.5
3.0
2.5
2.0
1.5
1.0
0.5
0.0
-0.5
f1 (ppm)

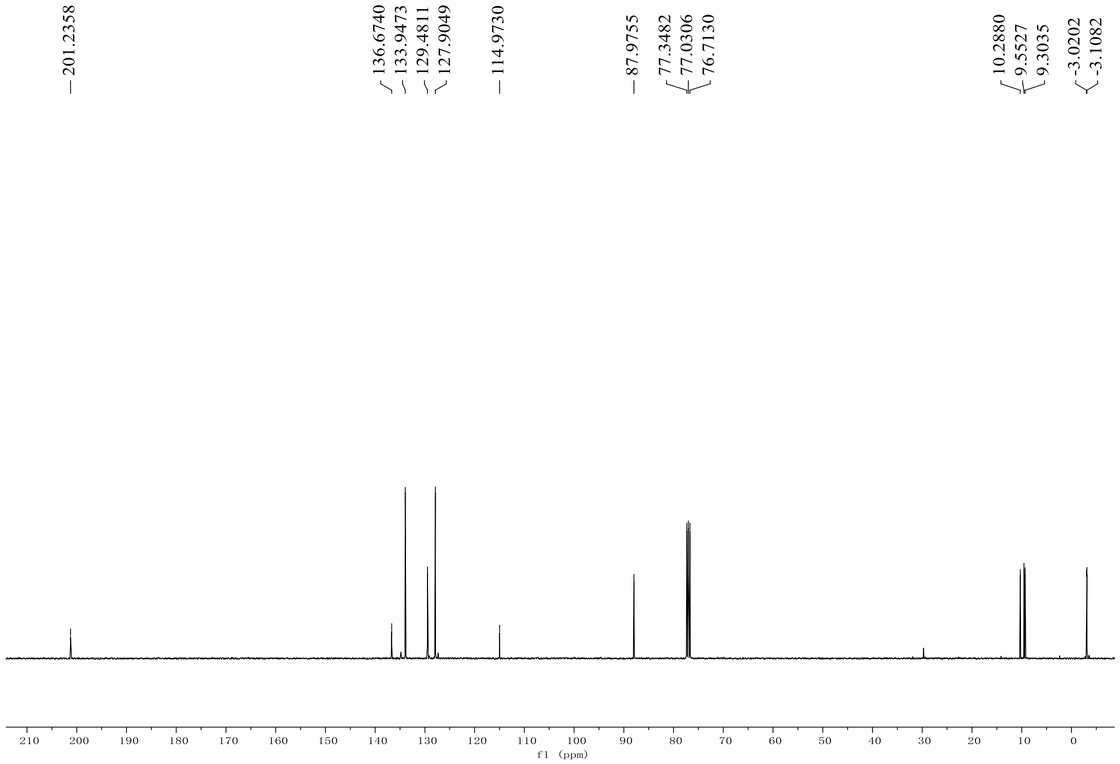
201.2358
136.6740
133.9473
129.4811
127.9049
114.9730
87.9755
77.3482
77.0306
76.7130
10.2880
9.5527
9.3035
-3.0202
-3.1082
210
200
190
180
170
160
150
140
130
120
110
100
90
80
70
60
50
40
30
20
10
0
f1 (ppm)

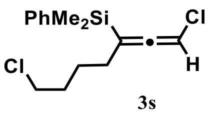
PhMe2Si
Cl
Cl
H

3s

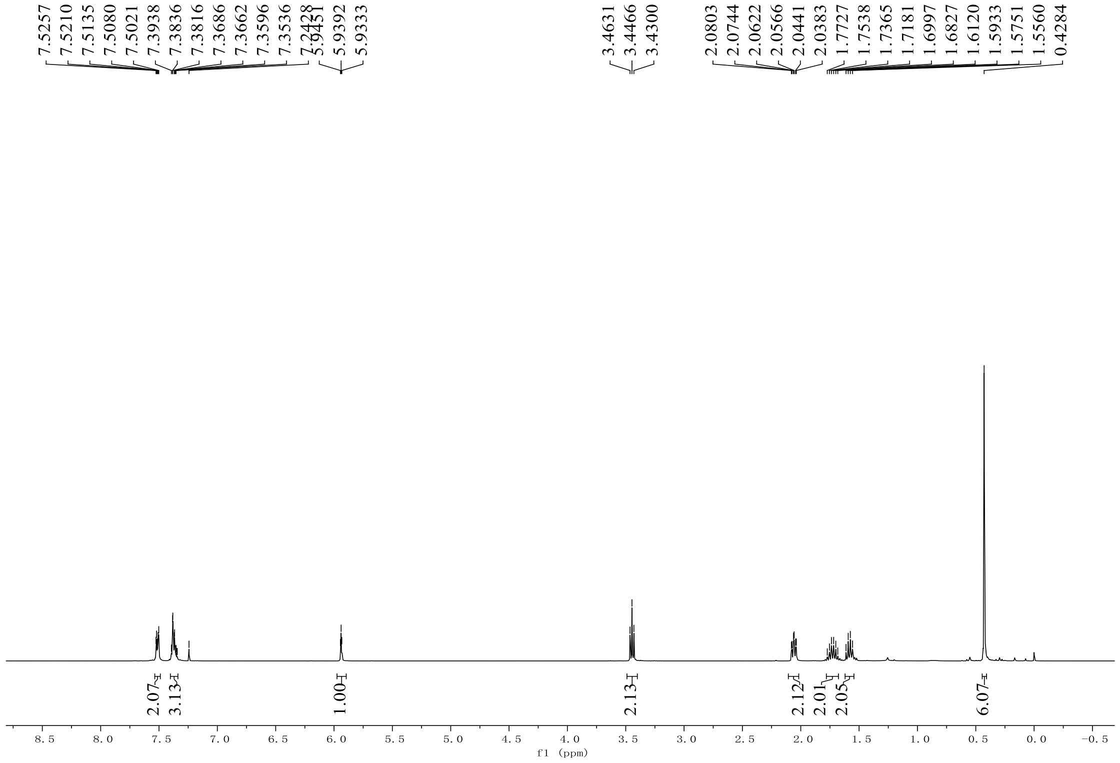
7.5257
7.5210
7.5135
7.5080
7.5021
7.3938
7.3836
7.3816
7.3686
7.3662
7.3596
7.3536
7.2428
5.9451
5.9392
5.9333
3.4631
3.4466
3.4300
2.0803
2.0744
2.0622
2.0566
2.0441
2.0383
1.7727
1.7538
1.7365
1.7181
1.6997
1.6827
1.6120
1.5933
1.5751
1.5560
0.4284
2.07
3.13
1.00
2.13
2.12
2.01
2.05
6.07
8.5
8.0
7.5
7.0
6.5
6.0
5.5
5.0
4.5
4.0
3.5
3.0
2.5
2.0
1.5
1.0
0.5
0.0
-0.5
f1 (ppm)

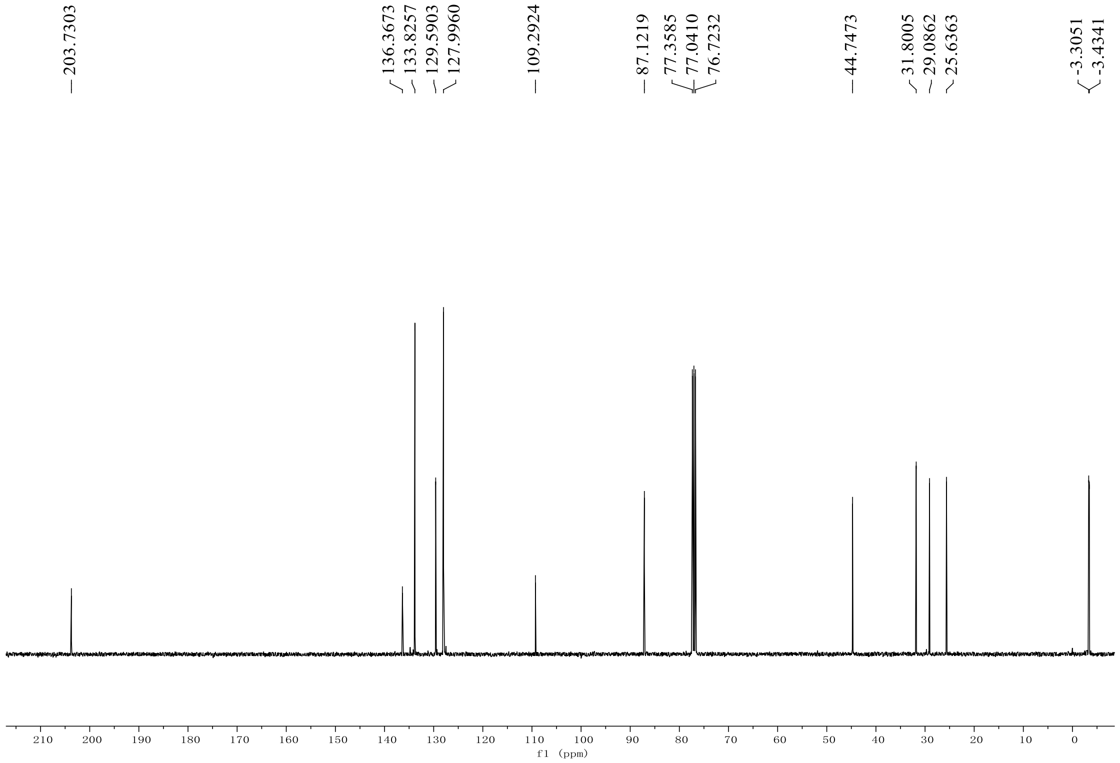
203.7303
136.3673
133.8257
129.5903
127.9960
109.2924
87.1219
77.3585
77.0410
76.7232
44.7473
31.8005
29.0862
25.6363
-3.3051
-3.4341
210
200
190
180
170
160
150
140
130
120
110
100
90
80
70
60
50
40
30
20
10
0
f1 (ppm)

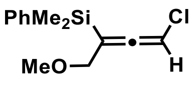
PhMe2Si
Cl
MeO
H

3t

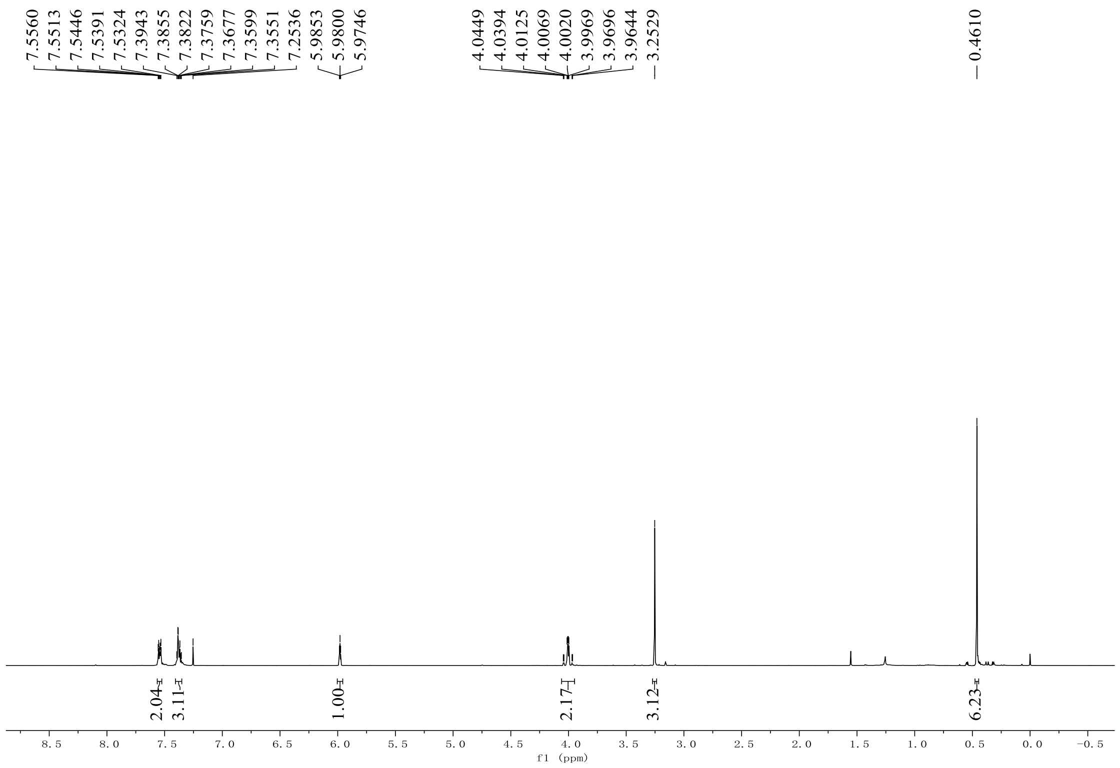
7.5560
7.5513
7.5446
7.5391
7.5324
7.3943
7.3855
7.3822
7.3759
7.3677
7.3599
7.3551
7.2536
5.9853
5.9800
5.9746
4.0449
4.0394
4.0125
4.0069
4.0020
3.9969
3.9696
3.9644
3.2529
0.4610
2.04
3.11
1.00
2.17
3.12
6.23
8.5 8.0 7.5 7.0 6.5 6.0 5.5 5.0 4.5 4.0 3.5 3.0 2.5 2.0 1.5 1.0 0.5 0.0 -0.5
f1 (ppm)

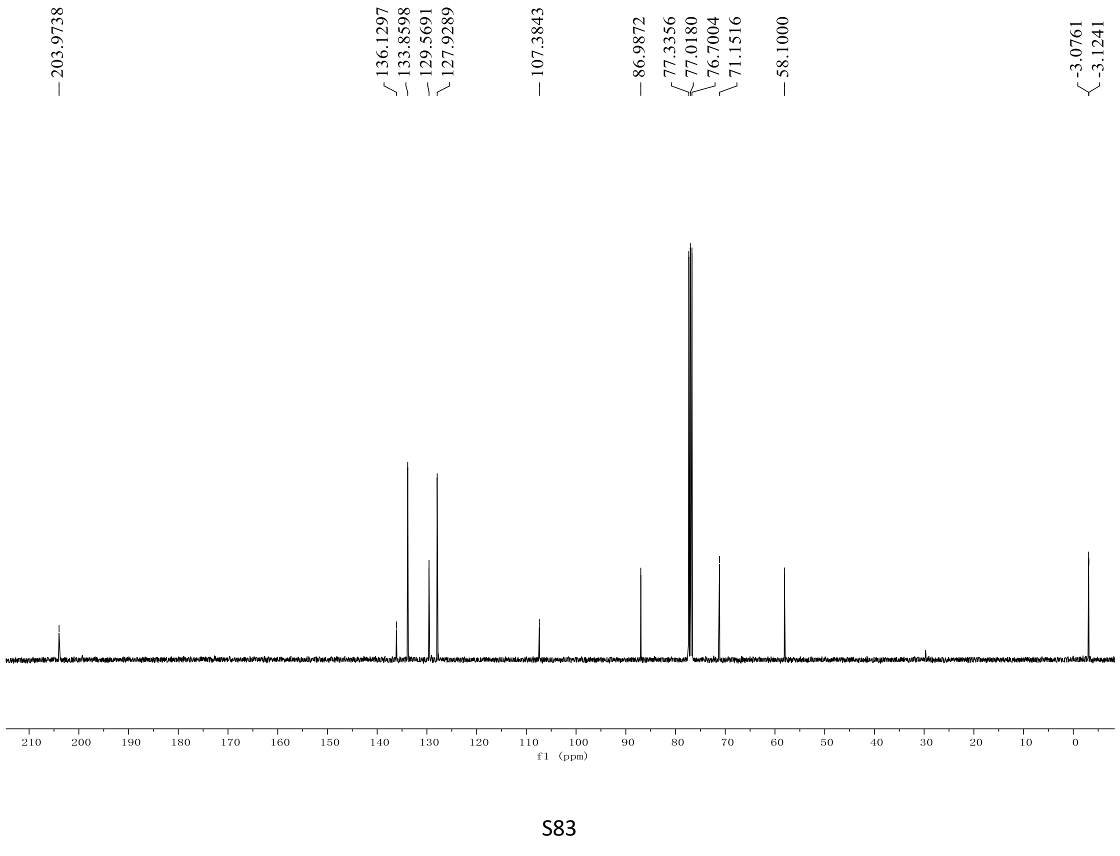
203.9738
136.1297
133.8598
129.5691
127.9289
107.3843
86.9872
77.3356
77.0180
76.7004
71.1516
58.1000
-3.0761
-3.1241
210 200 190 180 170 160 150 140 130 120 110 100 90 80 70 60 50 40 30 20 10 0
f1 (ppm)

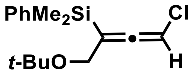
PhMe2Si
Cl
t-BuO
H

3u

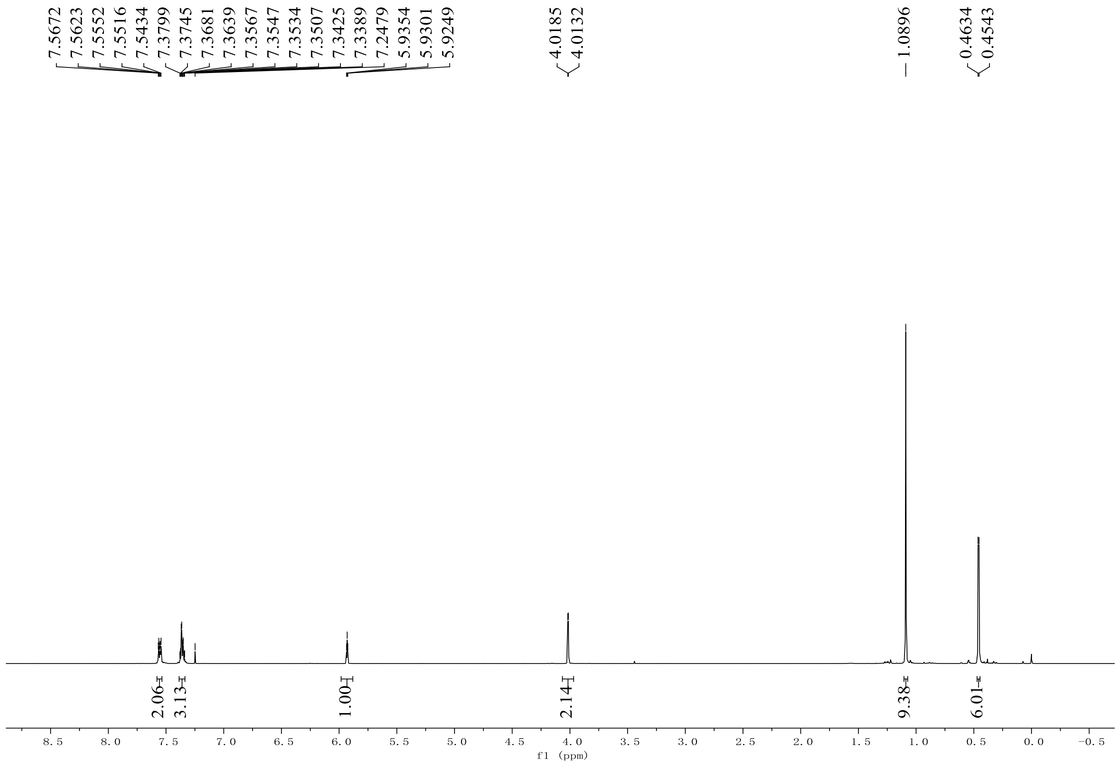
7.5672
7.5623
7.5552
7.5516
7.5434
7.3799
7.3745
7.3681
7.3639
7.3567
7.3547
7.3534
7.3507
7.3425
7.3389
7.2479
5.9354
5.9301
5.9249
4.0185
4.0132
1.0896
0.4634
0.4543
2.06
3.13
1.00
2.14
9.38
6.01
8.5
8.0
7.5
7.0
6.5
6.0
5.5
5.0
4.5
4.0
3.5
3.0
2.5
2.0
1.5
1.0
0.5
0.0
-0.5
f1 (ppm)

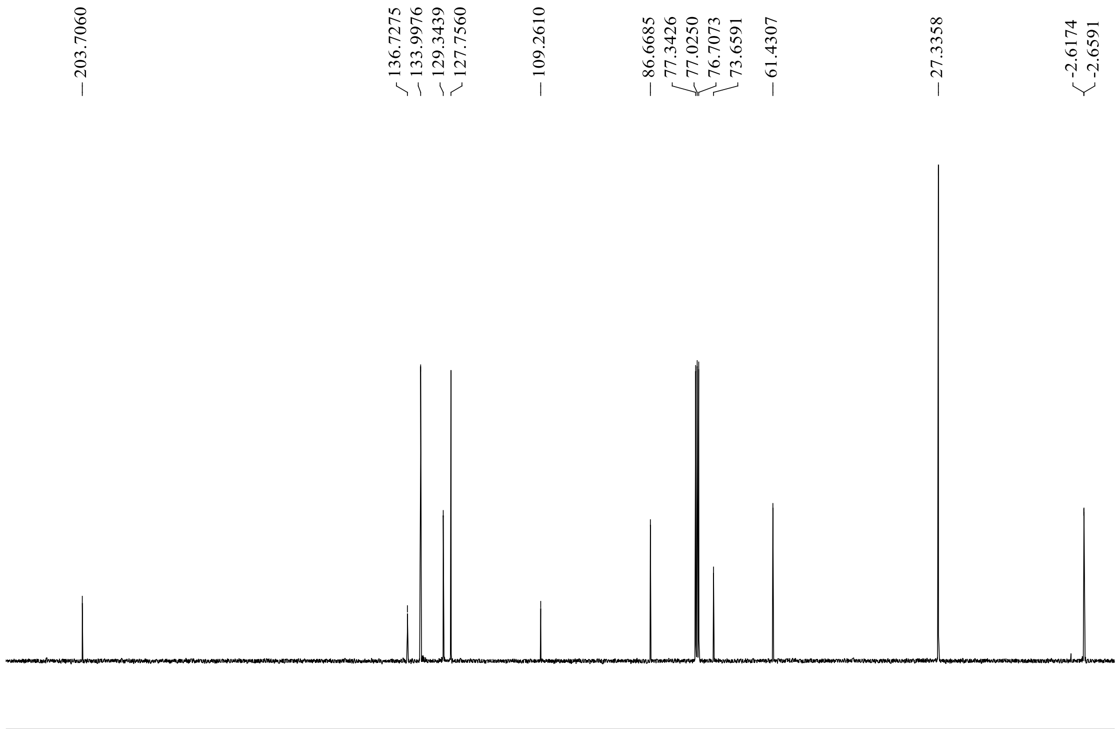
203.7060
136.7275
133.9976
129.3439
127.7560
109.2610
86.6685
77.3426
77.0250
76.7073
73.6591
61.4307
27.3358
-2.6174
-2.6591
210
200
190
180
170
160
150
140
130
120
110
100
90
80
70
60
50
40
30
20
10
0
f1 (ppm)

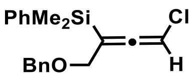
PhMe2Si
Cl
BnO
H

3v

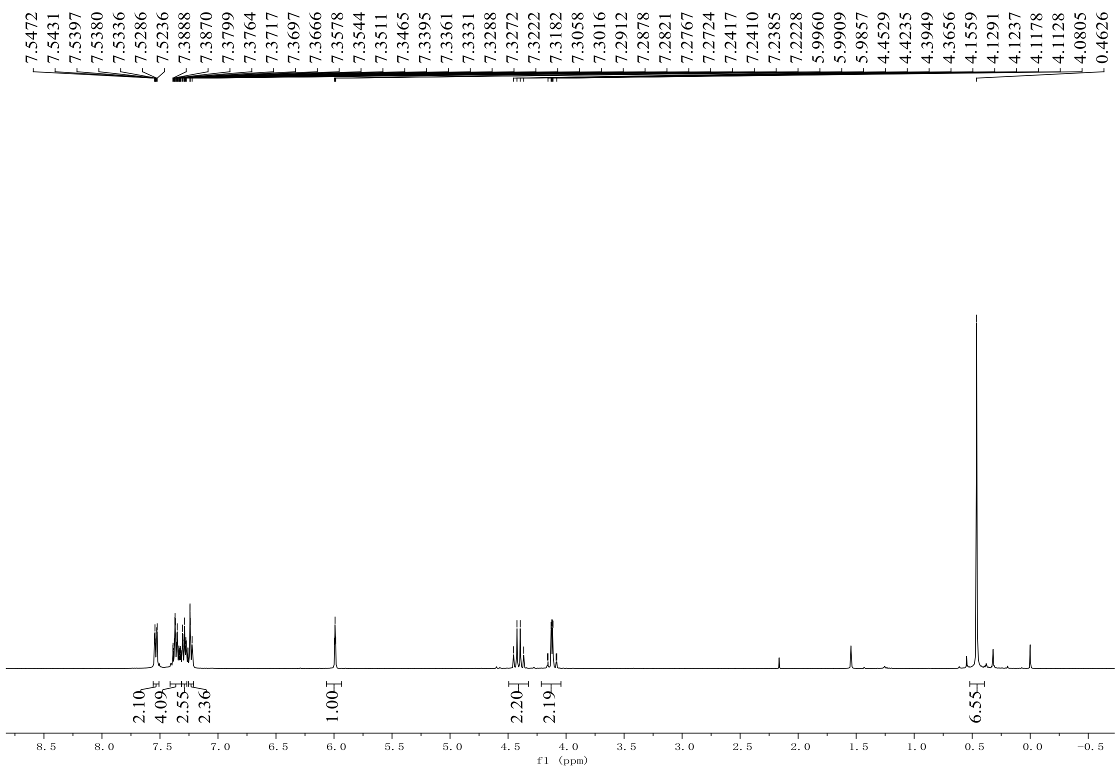
7.5472
7.5431
7.5397
7.5380
7.5336
7.5286
7.5236
7.3888
7.3870
7.3799
7.3764
7.3717
7.3697
7.3666
7.3578
7.3544
7.3511
7.3465
7.3395
7.3361
7.3331
7.3288
7.3272
7.3222
7.3182
7.3058
7.3016
7.2912
7.2878
7.2821
7.2767
7.2724
7.2417
7.2410
7.2385
7.2228
5.9960
5.9909
5.9857
4.4529
4.4235
4.3949
4.3656
4.1559
4.1291
4.1237
4.1178
4.1128
4.0805
0.4626
2.10
4.09
2.55
2.36
1.00
2.20
2.19
6.55
8.5
8.0
7.5
7.0
6.5
6.0
5.5
5.0
4.5
4.0
3.5
3.0
2.5
2.0
1.5
1.0
0.5
0.0
-0.5
f1 (ppm)

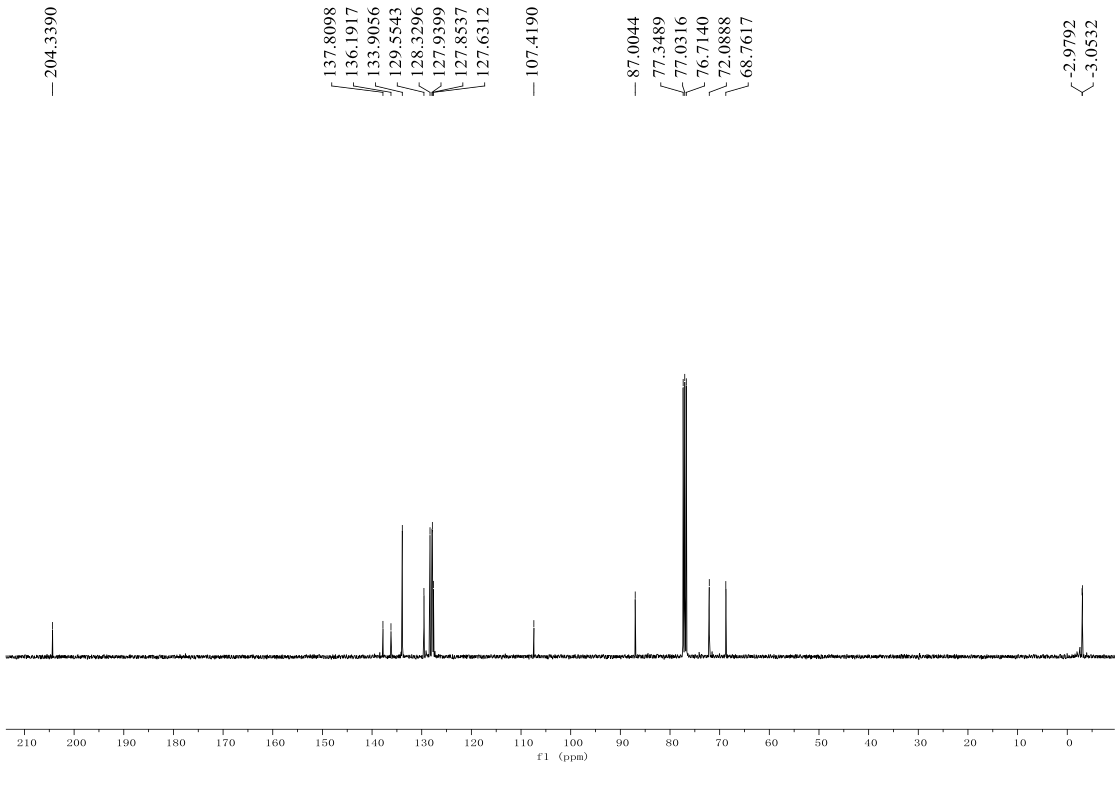
204.3390
137.8098
136.1917
133.9056
129.5543
128.3296
127.9399
127.8537
127.6312
107.4190
87.0044
77.3489
77.0316
76.7140
72.0888
68.7617
-2.9792
-3.0532
210
200
190
180
170
160
150
140
130
120
110
100
90
80
70
60
50
40
30
20
10
0
f1 (ppm)

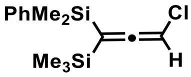

3w

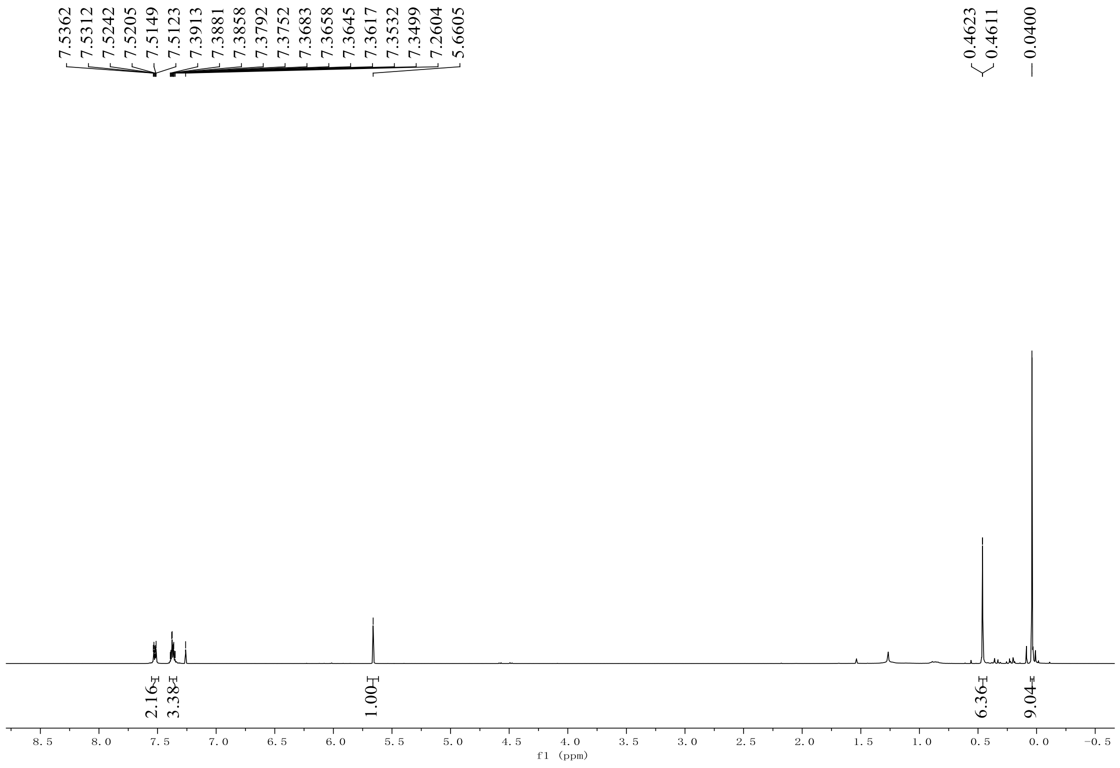

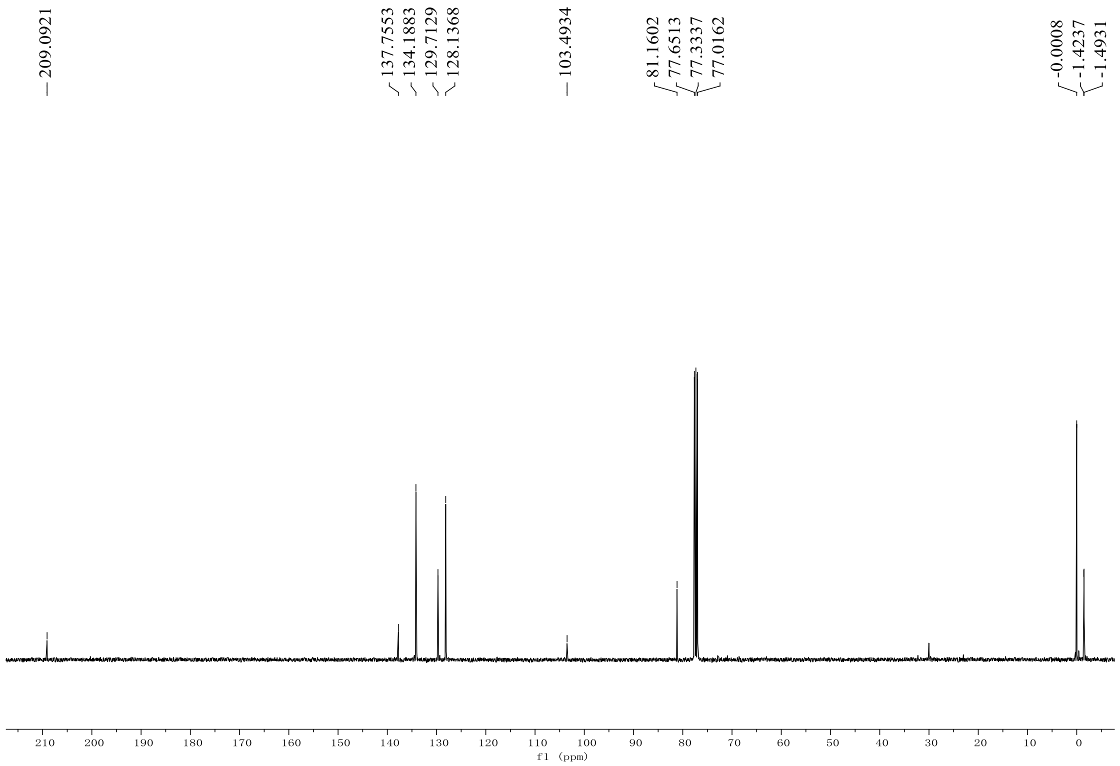

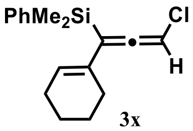
PhMe2Si
Cl
H

3x

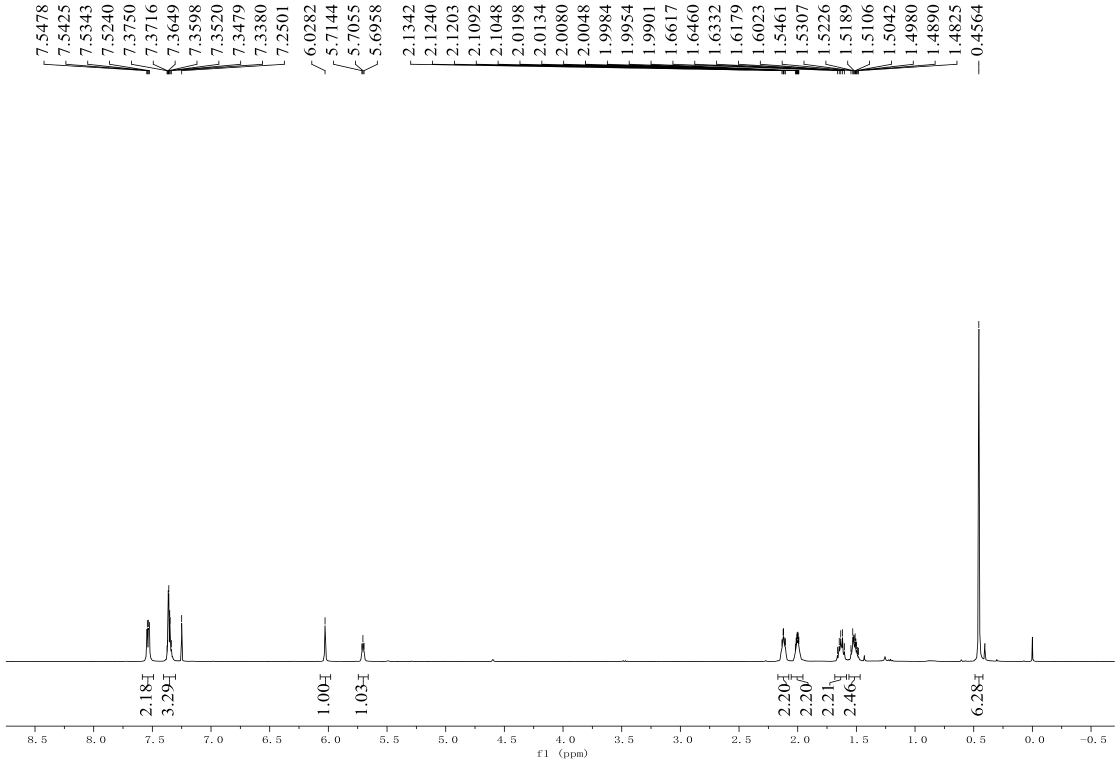
7.5478
7.5425
7.5343
7.5240
7.3750
7.3716
7.3649
7.3598
7.3520
7.3479
7.3380
7.2501
6.0282
5.7144
5.7055
5.6958
2.1342
2.1240
2.1203
2.1092
2.1048
2.0198
2.0134
2.0080
2.0048
1.9984
1.9954
1.9901
1.6617
1.6460
1.6332
1.6179
1.6023
1.5461
1.5307
1.5226
1.5189
1.5106
1.5042
1.4980
1.4890
1.4825
0.4564
2.18
3.29
1.00
1.03
2.20
2.20
2.21
2.46
6.28
f1 (ppm)

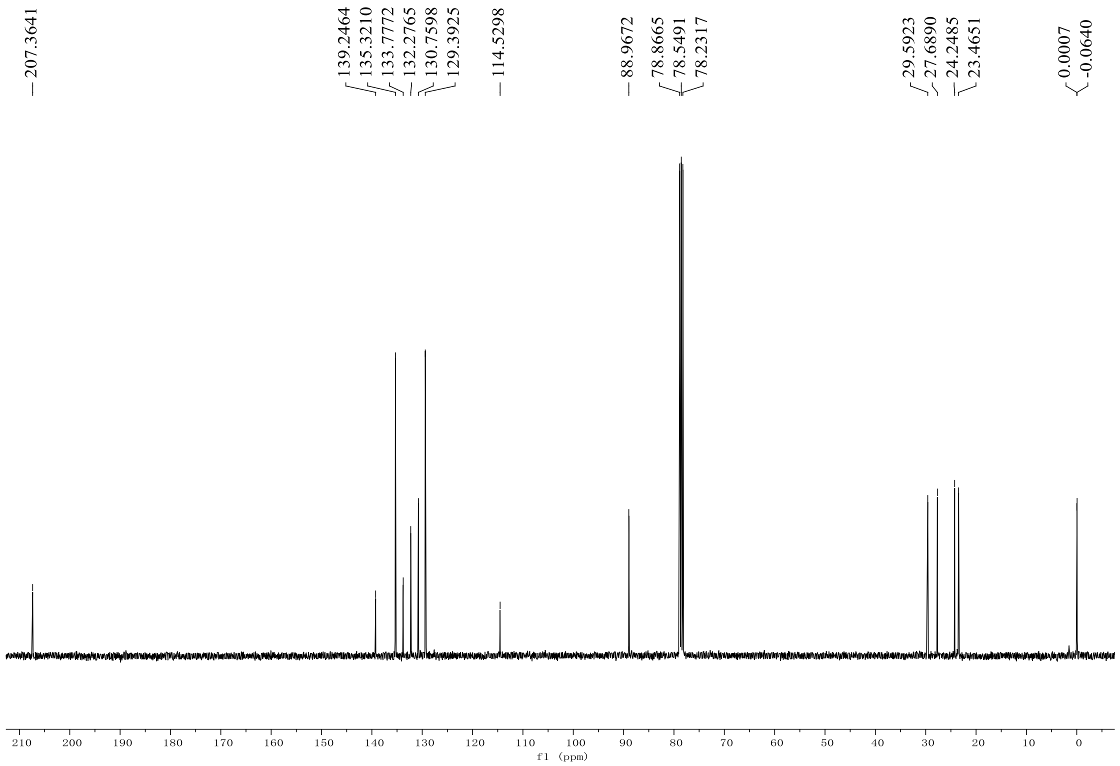
207.3641
139.2464
135.3210
133.7772
132.2765
130.7598
129.3925
114.5298
88.9672
78.8665
78.5491
78.2317
29.5923
27.6890
24.2485
23.4651
0.0007
-0.0640
f1 (ppm)

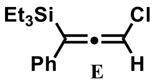
Et3Si
Cl
Ph
H

E

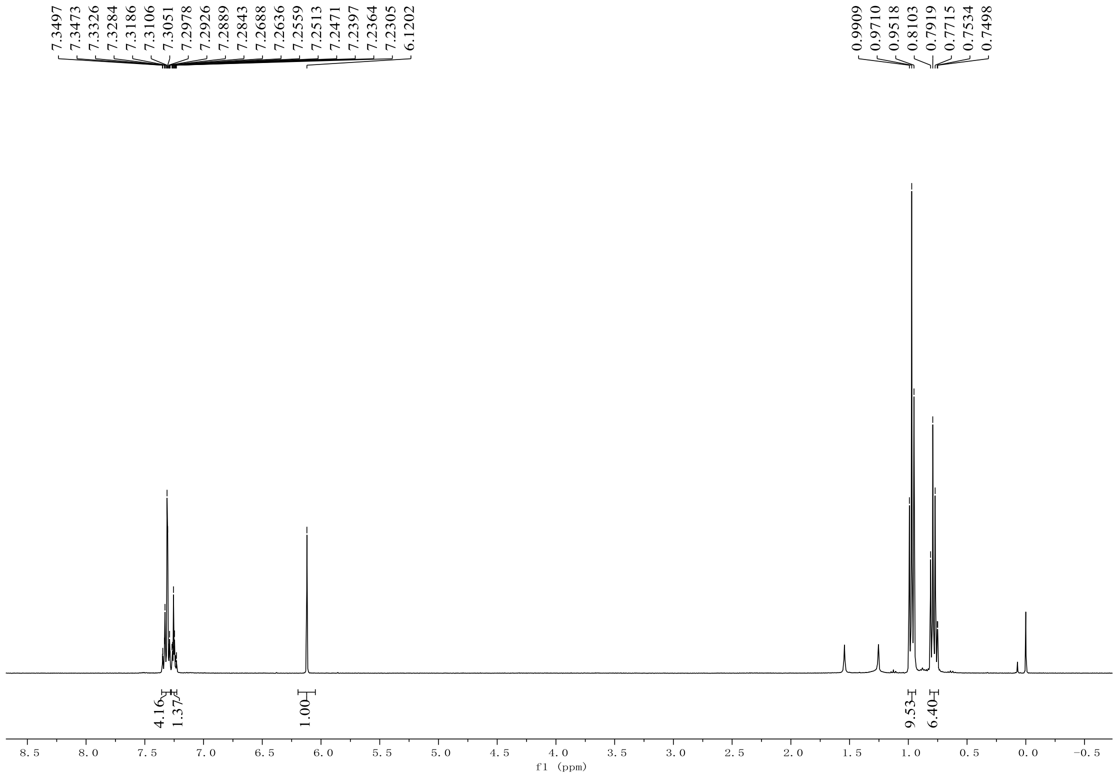
7.3497
7.3473
7.3326
7.3284
7.3186
7.3106
7.3051
7.2978
7.2926
7.2889
7.2843
7.2688
7.2636
7.2559
7.2513
7.2471
7.2397
7.2364
7.2305
6.1202
0.9909
0.9710
0.9518
0.8103
0.7919
0.7715
0.7534
0.7498
4.16
1.37
1.00
9.53
6.40
f1 (ppm)

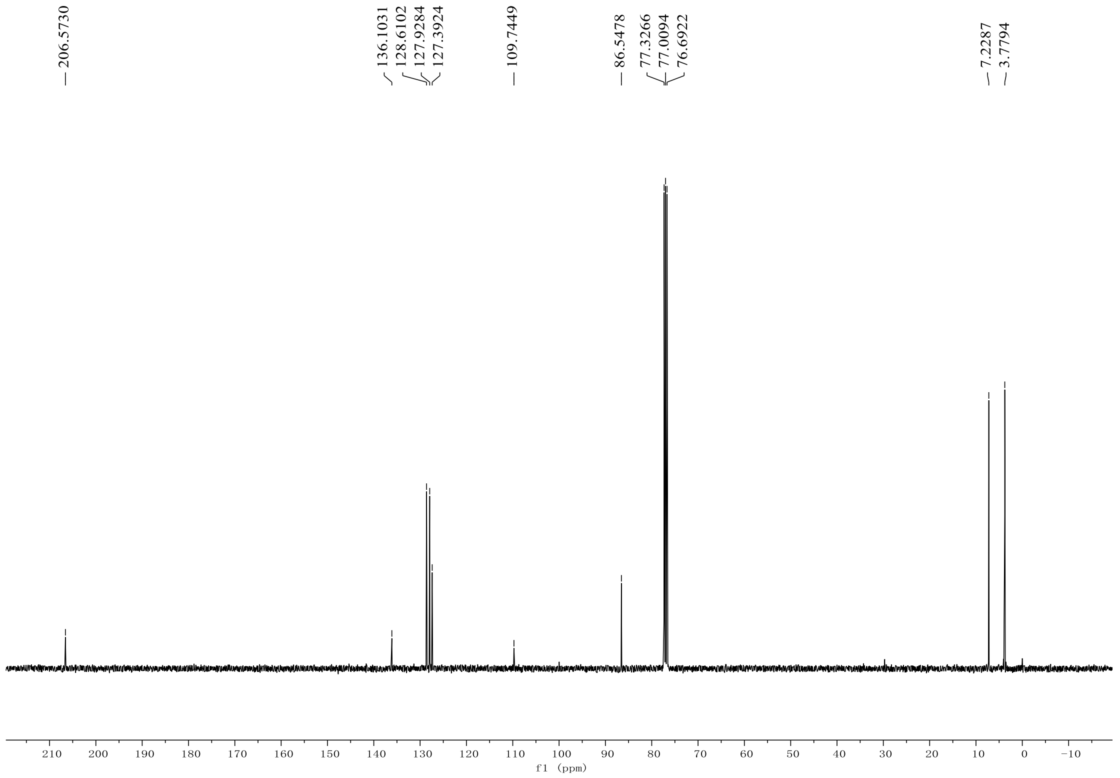
206.5730
136.1031
128.6102
127.9284
127.3924
109.7449
86.5478
77.3266
77.0094
76.6922
7.2287
3.7794
f1 (ppm)

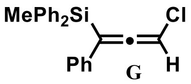
MePh2Si
Cl
Ph
G
H

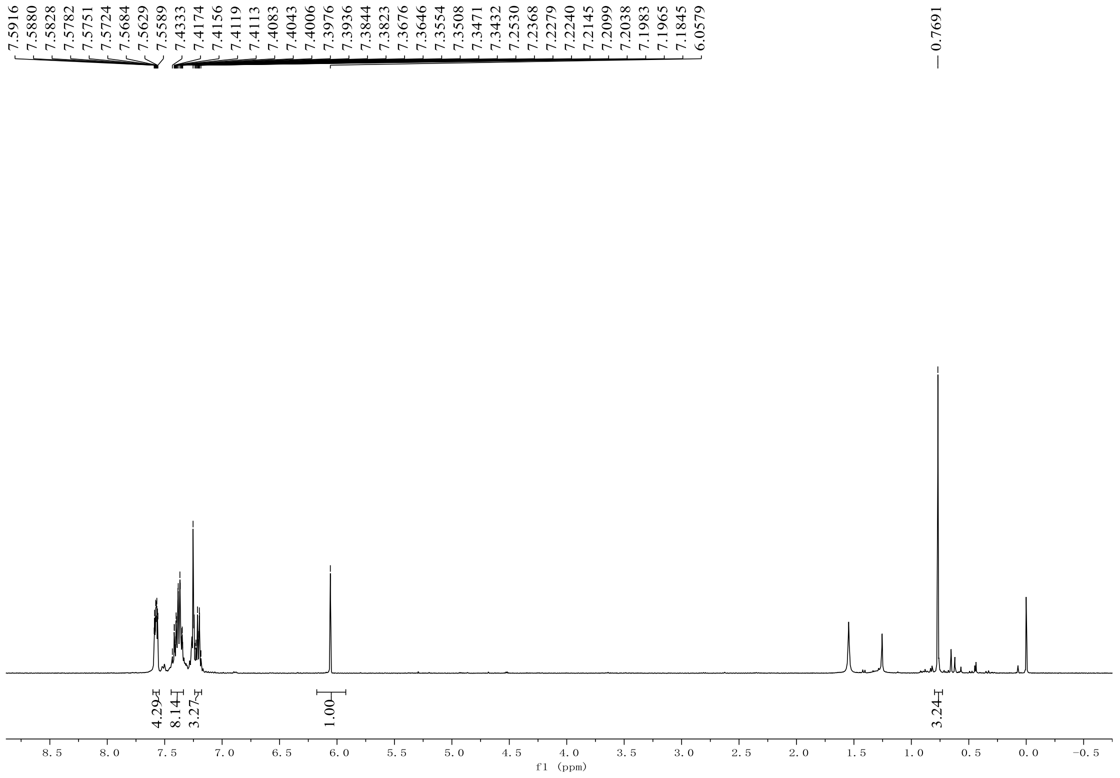
7.5916
7.5880
7.5828
7.5782
7.5751
7.5724
7.5684
7.5629
7.5589
7.4333
7.4174
7.4156
7.4119
7.4113
7.4083
7.4043
7.4006
7.3976
7.3936
7.3844
7.3823
7.3676
7.3646
7.3554
7.3508
7.3471
7.3432
7.2530
7.2368
7.2279
7.2240
7.2145
7.2099
7.2038
7.1983
7.1965
7.1845
6.0579
0.7691
4.29
8.14
3.27
1.00
3.24
f1 (ppm)

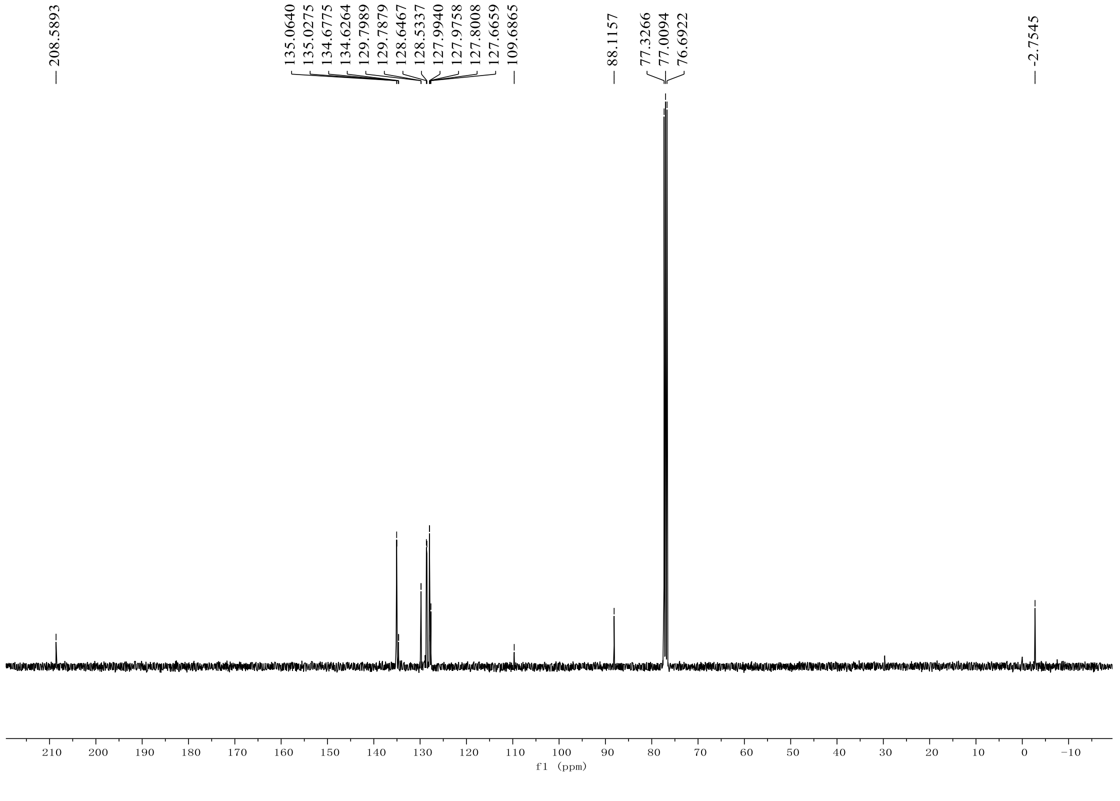
208.5893
135.0640
135.0275
134.6775
134.6264
129.7989
129.7879
128.6467
128.5337
127.9940
127.9758
127.8008
127.6659
109.6865
88.1157
77.3266
77.0094
76.6922
-2.7545
f1 (ppm)

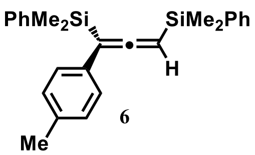
PhMe2Si
SiMe2Ph
H
Me

6

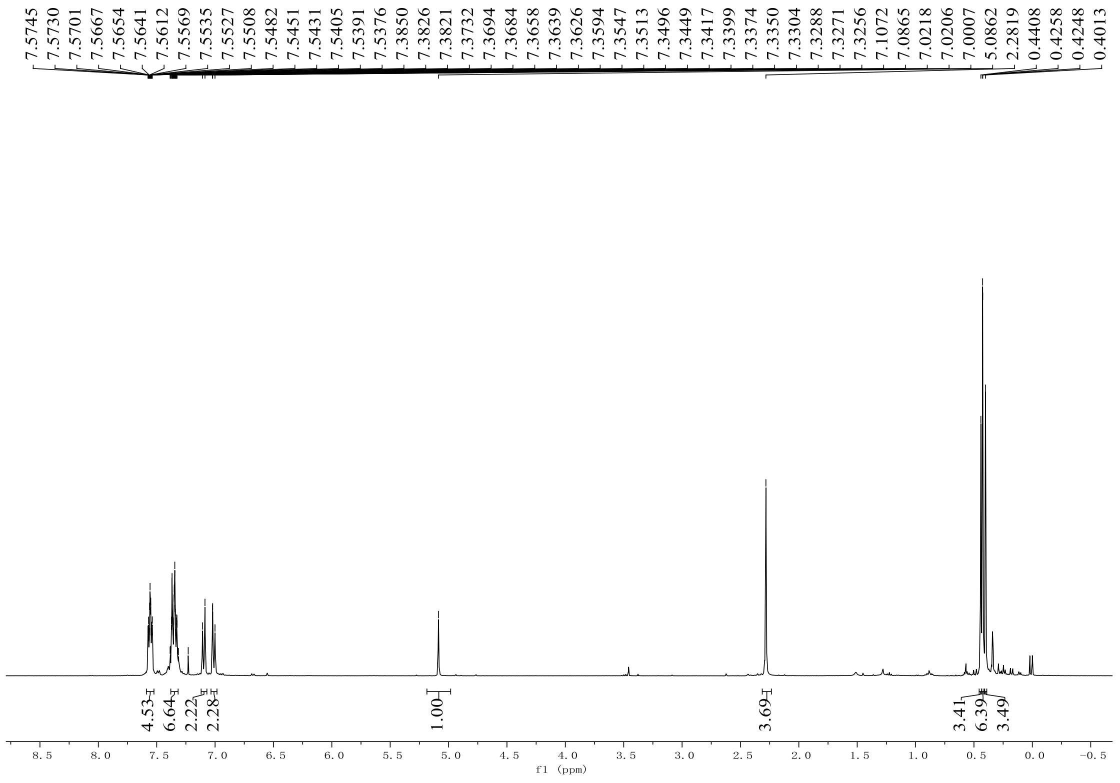
7.5745 7.5730 7.5701 7.5667 7.5654 7.5641 7.5612 7.5569 7.5535 7.5527 7.5508 7.5482 7.5451 7.5431 7.5405 7.5391 7.5376 7.3850 7.3826 7.3821 7.3732 7.3694 7.3684 7.3658 7.3639 7.3626 7.3594 7.3547 7.3513 7.3496 7.3449 7.3417 7.3399 7.3374 7.3350 7.3304 7.3288 7.3271 7.3256 7.1072 7.0865 7.0218 7.0206 7.0007 5.0862 2.2819 0.4408 0.4258 0.4248 0.4013
4.53
6.64
2.22
2.28
1.00
3.69
3.41
6.39
3.49
8.5 8.0 7.5 7.0 6.5 6.0 5.5 5.0 4.5 4.0 3.5 3.0 2.5 2.0 1.5 1.0 0.5 0.0 -0.5
f1 (ppm)

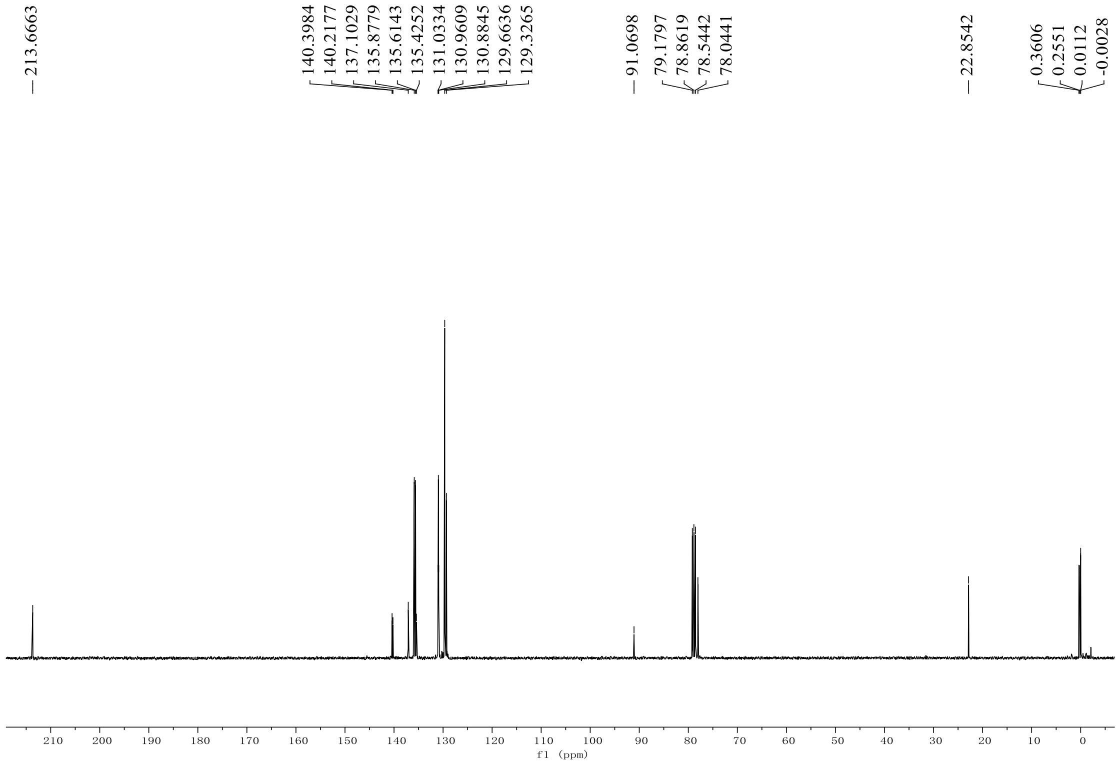
213.6663
140.3984 140.2177 137.1029 135.8779 135.6143 135.4252 131.0334 130.9609 130.8845 129.6636 129.3265
91.0698
79.1797 78.8619 78.5442 78.0441
22.8542
0.3606 0.2551 0.0112 -0.0028
210 200 190 180 170 160 150 140 130 120 110 100 90 80 70 60 50 40 30 20 10 0
f1 (ppm)

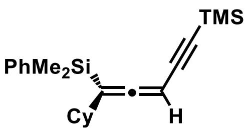
TMS
$PhMe_2Si$
Cy
H

7

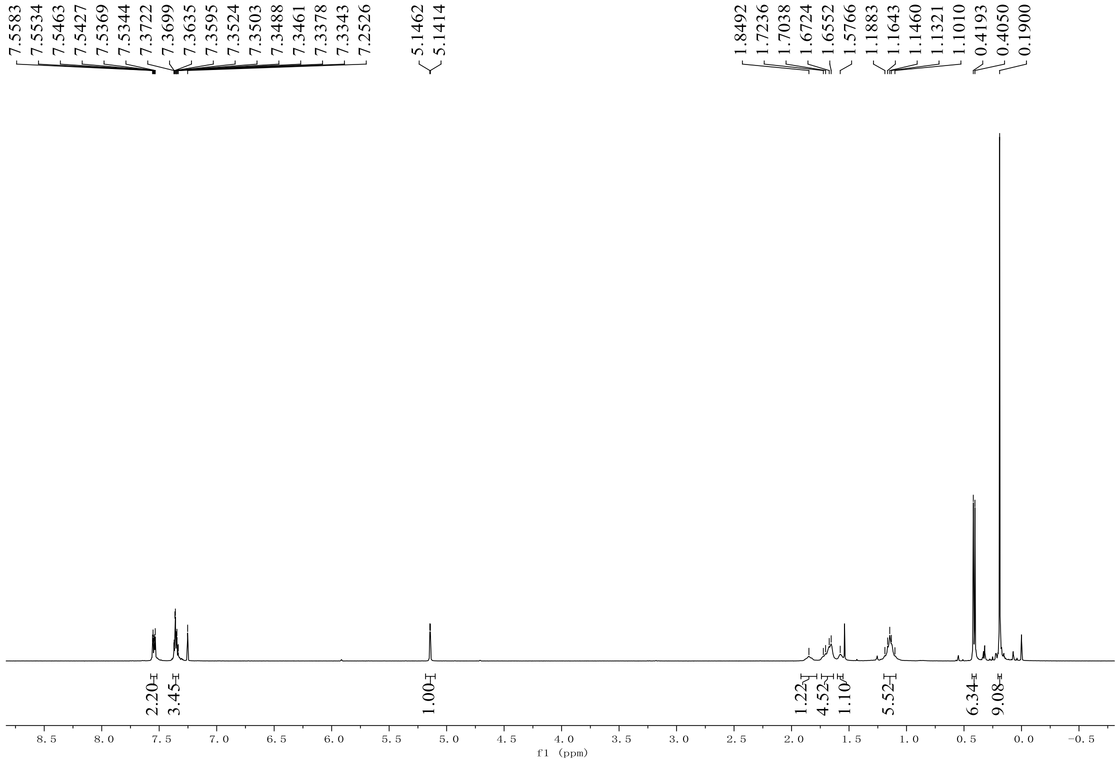
7.5583
7.5534
7.5463
7.5427
7.5369
7.5344
7.3722
7.3699
7.3635
7.3595
7.3524
7.3503
7.3488
7.3461
7.3378
7.3343
7.2526
5.1462
5.1414
1.8492
1.7236
1.7038
1.6724
1.6552
1.5766
1.1883
1.1643
1.1460
1.1321
1.1010
0.4193
0.4050
0.1900
2.20
3.45
1.00
1.22
4.52
1.10
5.52
6.34
9.08
8.5
8.0
7.5
7.0
6.5
6.0
5.5
5.0
4.5
4.0
3.5
3.0
2.5
2.0
1.5
1.0
0.5
0.0
-0.5
f1 (ppm)

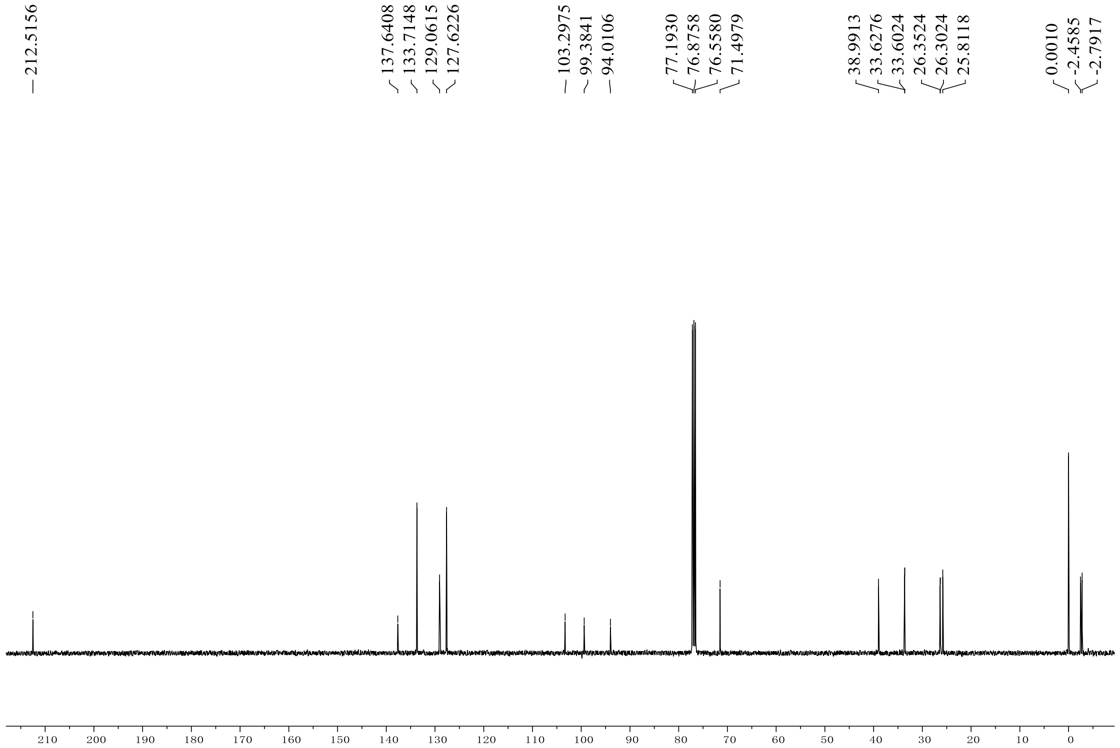
212.5156
137.6408
133.7148
129.0615
127.6226
103.2975
99.3841
94.0106
77.1930
76.8758
76.5580
71.4979
38.9913
33.6276
33.6024
26.3524
26.3024
25.8118
0.0010
-2.4585
-2.7917
210
200
190
180
170
160
150
140
130
120
110
100
90
80
70
60
50
40
30
20
10
0
f1 (ppm)

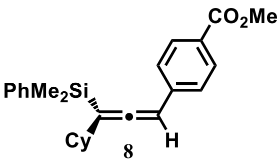
CO2Me
PhMe2Si
Cy
H

8

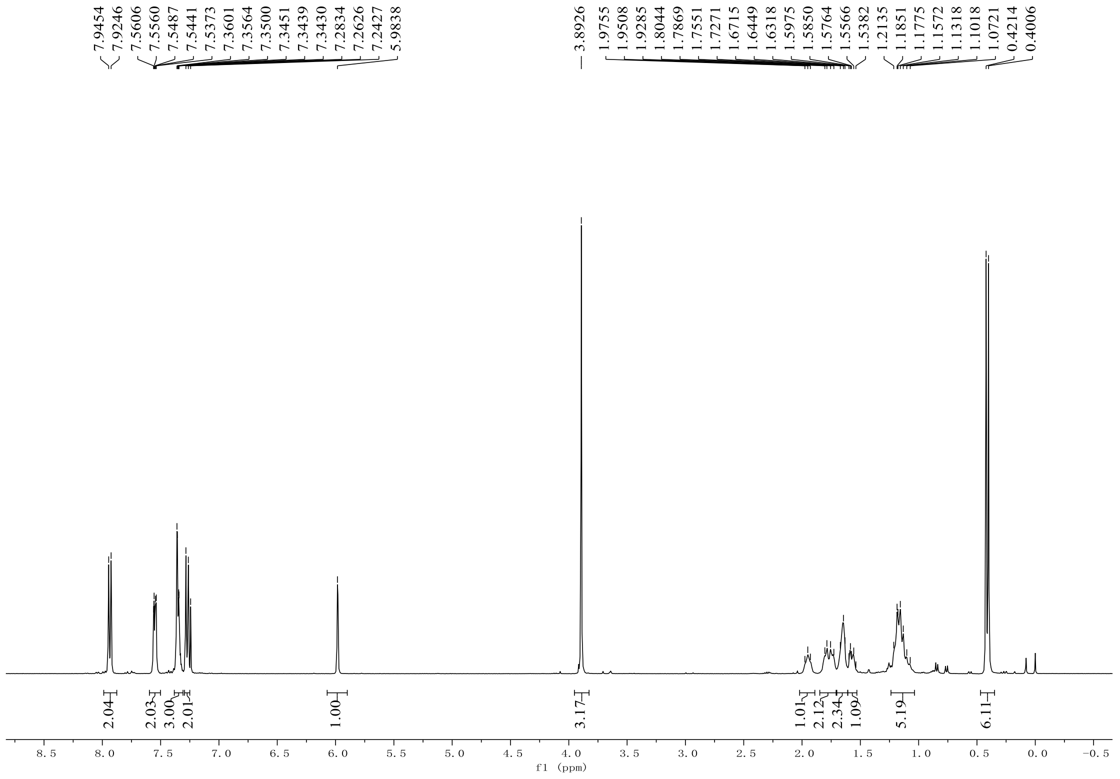
7.9454
7.9246
7.5606
7.5560
7.5487
7.5441
7.5373
7.3601
7.3564
7.3500
7.3451
7.3439
7.3430
7.2834
7.2626
7.2427
5.9838
3.8926
1.9755
1.9508
1.9285
1.8044
1.7869
1.7551
1.7271
1.6715
1.6449
1.6318
1.5975
1.5850
1.5764
1.5566
1.5382
1.2135
1.1851
1.1775
1.1572
1.1318
1.1018
1.0721
0.4214
0.4006
2.04
2.03
3.00
2.01
1.00
3.17
1.01
2.12
2.34
1.09
5.19
6.11
f1 (ppm)

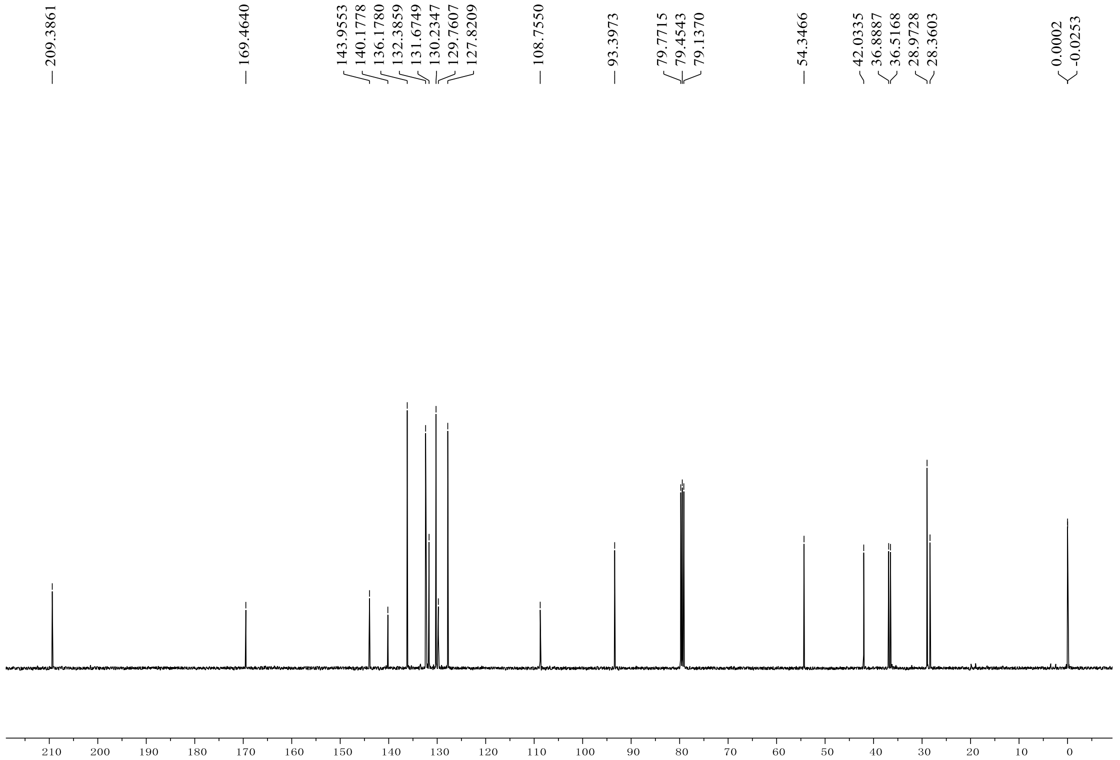
209.3861
169.4640
143.9553
140.1778
136.1780
132.3859
131.6749
130.2347
129.7607
127.8209
108.7550
93.3973
79.7715
79.4543
79.1370
54.3466
42.0335
36.8887
36.5168
28.9728
28.3603
0.0002
-0.0253
f1 (ppm)

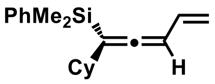
PhMe2Si
Cy
H

9

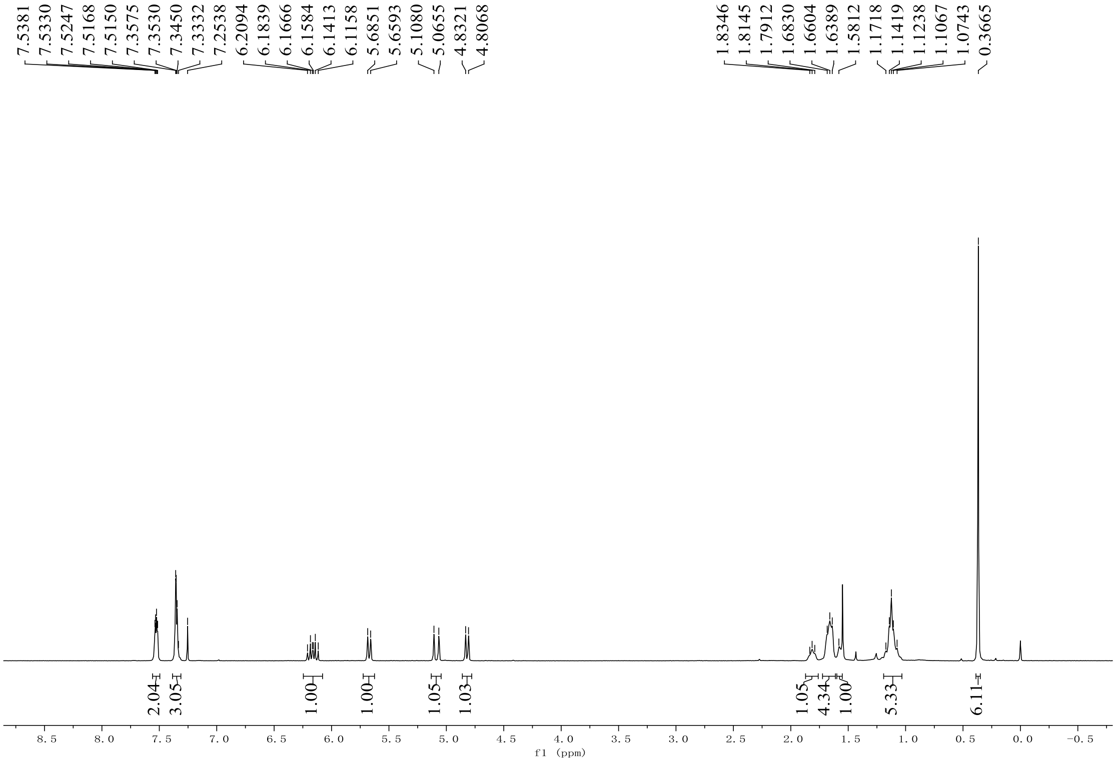
7.5381
7.5330
7.5247
7.5168
7.5150
7.3575
7.3530
7.3450
7.3332
7.2538
6.2094
6.1839
6.1666
6.1584
6.1413
6.1158
5.6851
5.6593
5.1080
5.0655
4.8321
4.8068
1.8346
1.8145
1.7912
1.6830
1.6604
1.6389
1.5812
1.1718
1.1419
1.1238
1.1067
1.0743
0.3665
2.04
3.05
1.00
1.00
1.05
1.03
1.05
4.34
1.00
5.33
6.11
8.5
8.0
7.5
7.0
6.5
6.0
5.5
5.0
4.5
4.0
3.5
3.0
2.5
2.0
1.5
1.0
0.5
0.0
-0.5
f1 (ppm)

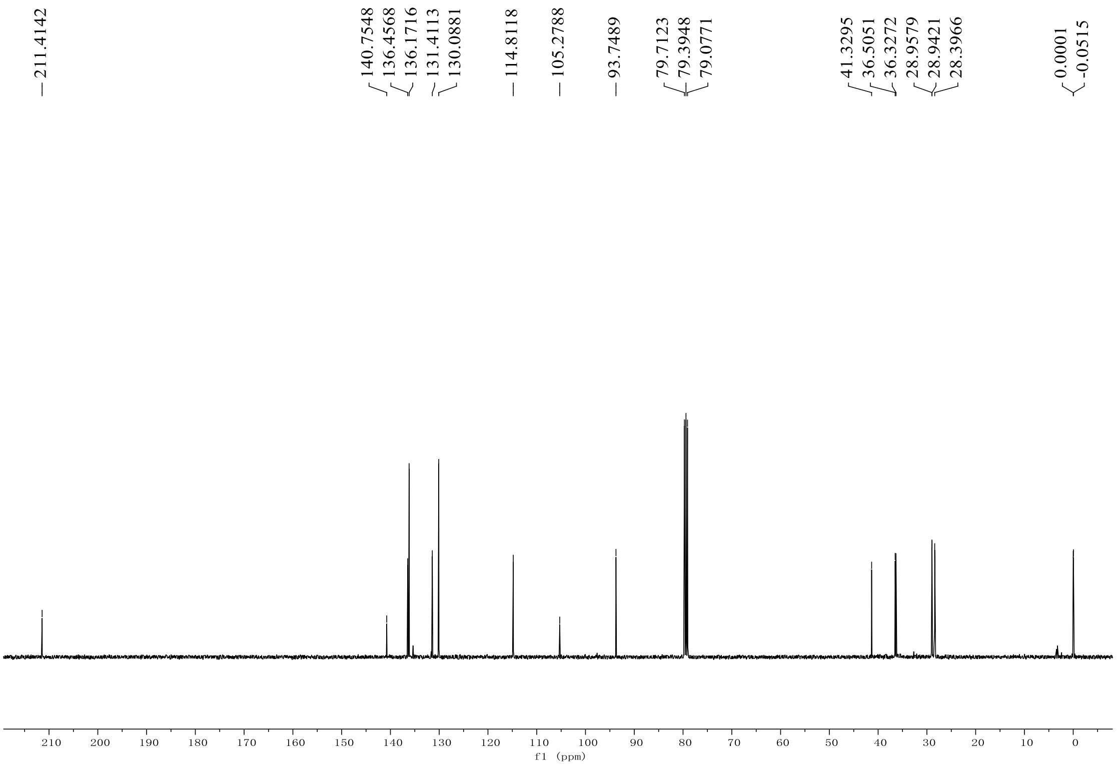
211.4142
140.7548
136.4568
136.1716
131.4113
130.0881
114.8118
105.2788
93.7489
79.7123
79.3948
79.0771
41.3295
36.5051
36.3272
28.9579
28.9421
28.3966
0.0001
-0.0515
210
200
190
180
170
160
150
140
130
120
110
100
90
80
70
60
50
40
30
20
10
0
f1 (ppm)

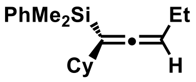

10

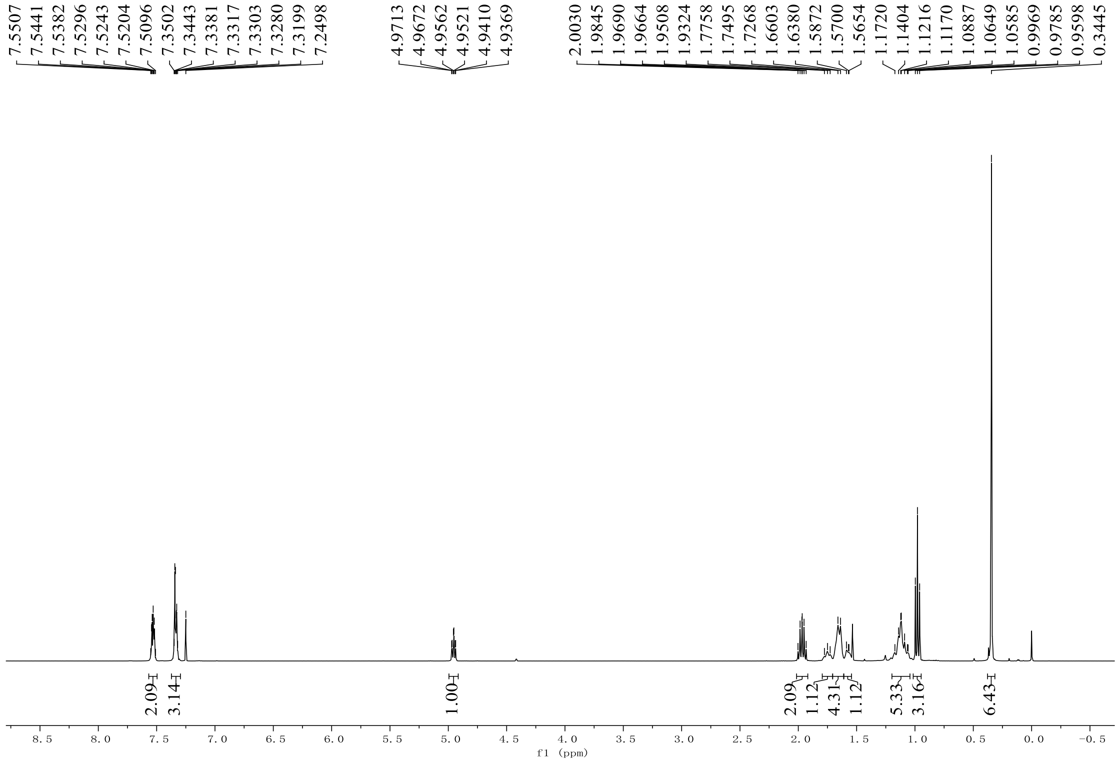

7.5507
7.5441
7.5382
7.5296
7.5243
7.5204
7.5096
7.3502
7.3443
7.3381
7.3317
7.3303
7.3280
7.3199
7.2498
4.9713
4.9672
4.9562
4.9521
4.9410
4.9369
2.0030
1.9845
1.9690
1.9664
1.9508
1.9324
1.7758
1.7495
1.7268
1.6603
1.6380
1.5872
1.5700
1.5654
1.1720
1.1404
1.1216
1.1170
1.0887
1.0649
1.0585
0.9969
0.9785
0.9598
0.3445
2.09
3.14
1.00
2.09
1.12
4.31
1.12
5.33
3.16
6.43
f1 (ppm)

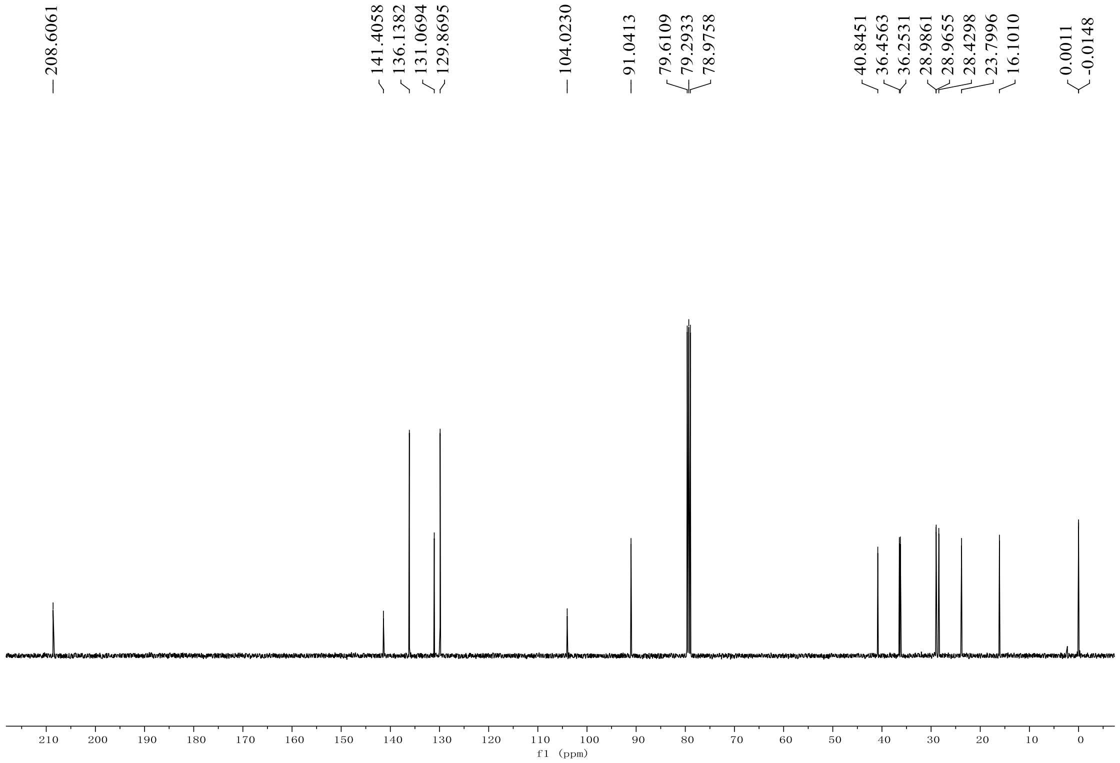

208.6061
141.4058
136.1382
131.0694
129.8695
104.0230
91.0413
79.6109
79.2933
78.9758
40.8451
36.4563
36.2531
28.9861
28.9655
28.4298
23.7996
16.1010
0.0011
-0.0148
f1 (ppm)

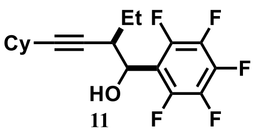
Cy
Et
F
F
F
HO
F
F

11

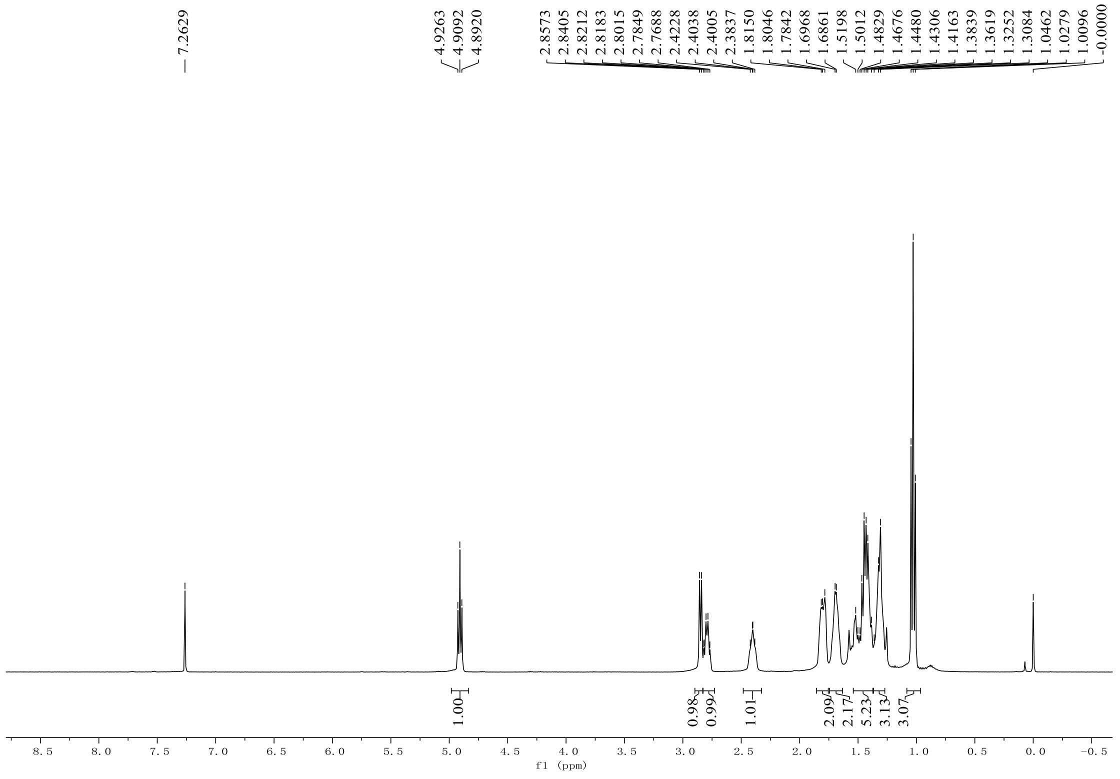
7.2629
4.9263
4.9092
4.8920
2.8573
2.8405
2.8212
2.8183
2.8015
2.7849
2.7688
2.4228
2.4038
2.4005
2.3837
1.8150
1.8046
1.7842
1.6968
1.6861
1.5198
1.5012
1.4829
1.4676
1.4480
1.4306
1.4163
1.3839
1.3619
1.3252
1.3084
1.0462
1.0279
1.0096
-0.0000
1.00
0.98
0.99
1.01
2.09
2.17
5.23
3.13
3.07
8.5 8.0 7.5 7.0 6.5 6.0 5.5 5.0 4.5 4.0 3.5 3.0 2.5 2.0 1.5 1.0 0.5 0.0 -0.5
f1 (ppm)

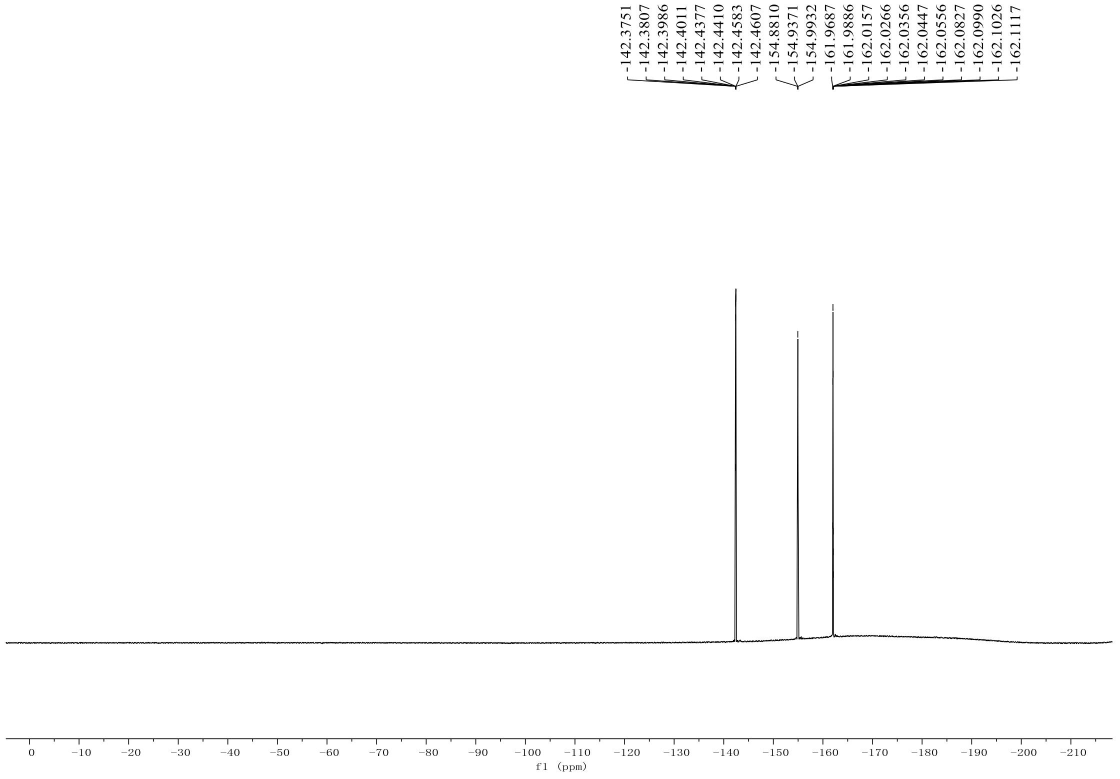
-142.3751
-142.3807
-142.3986
-142.4011
-142.4377
-142.4410
-142.4583
-142.4607
-154.8810
-154.9371
-154.9932
-161.9687
-161.9886
-162.0157
-162.0266
-162.0356
-162.0447
-162.0556
-162.0827
-162.0990
-162.1026
-162.1117
0 -10 -20 -30 -40 -50 -60 -70 -80 -90 -100 -110 -120 -130 -140 -150 -160 -170 -180 -190 -200 -210
f1 (ppm)

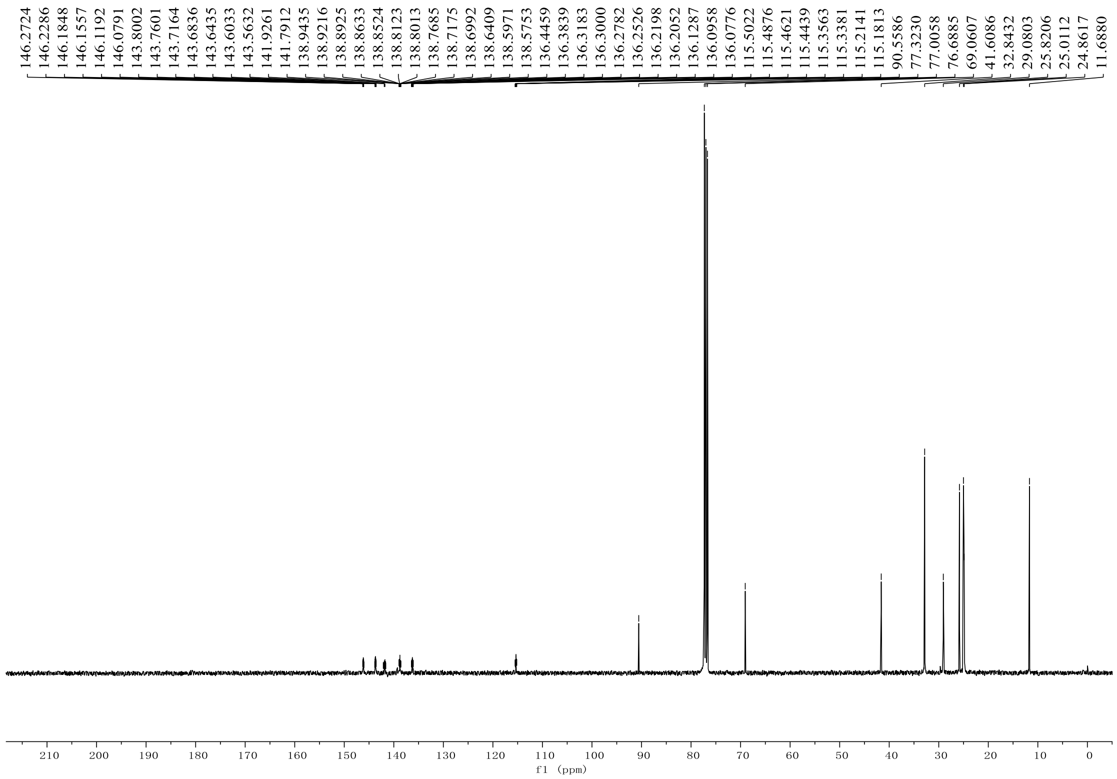
146.2724
146.2286
146.1848
146.1557
146.1192
146.0791
143.8002
143.7601
143.7164
143.6836
143.6435
143.6033
143.5632
141.9261
141.7912
138.9435
138.9216
138.8925
138.8633
138.8524
138.8123
138.8013
138.7685
138.7175
138.6992
138.6409
138.5971
138.5753
136.4459
136.3839
136.3183
136.3000
136.2782
136.2526
136.2198
136.2052
136.1287
136.0958
136.0776
115.5022
115.4876
115.4621
115.4439
115.3563
115.3381
115.2141
115.1813
90.5586
77.3230
77.0058
76.6885
69.0607
41.6086
32.8432
29.0803
25.8206
25.0112
24.8617
11.6880
210 200 190 180 170 160 150 140 130 120 110 100 90 80 70 60 50 40 30 20 10 0
f1 (ppm)

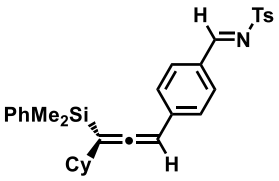
H
Ts
N
PhMe2Si
Cy
H

(*E*, *S*)-**14**

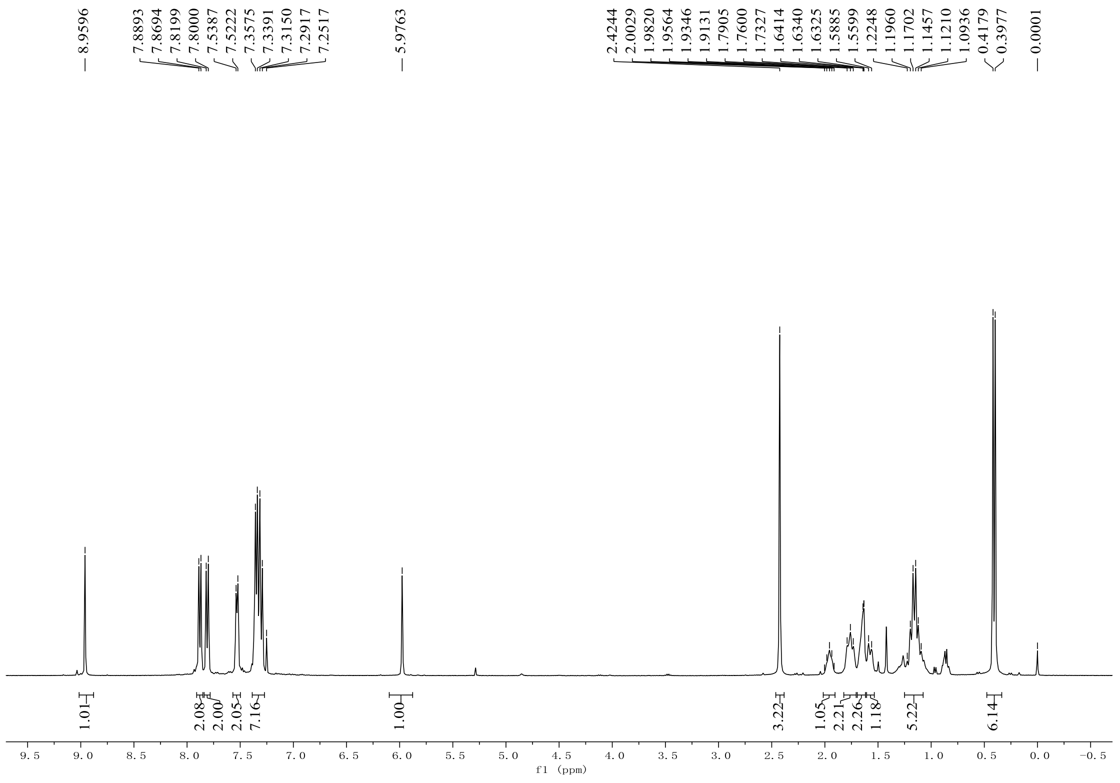
8.9596
7.8893
7.8694
7.8199
7.8000
7.5387
7.5222
7.3575
7.3391
7.3150
7.2917
7.2517
5.9763
2.4244
2.0029
1.9820
1.9564
1.9346
1.9131
1.7905
1.7600
1.7327
1.6414
1.6340
1.6325
1.5885
1.5599
1.2248
1.1960
1.1702
1.1457
1.1210
1.0936
0.4179
0.3977
0.0001
1.01
2.08
2.00
2.05
7.16
1.00
3.22
1.05
2.21
2.26
1.18
5.22
6.14
f1 (ppm)

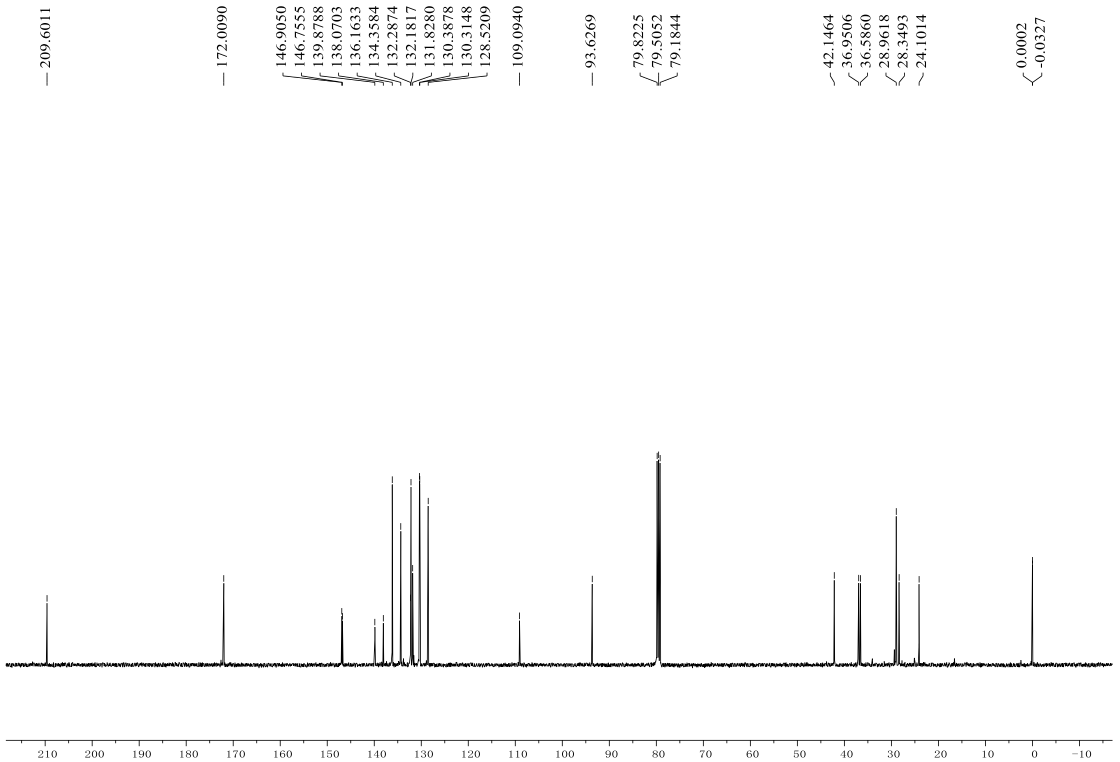
209.6011
172.0090
146.9050
146.7555
139.8788
138.0703
136.1633
134.3584
132.2874
132.1817
131.8280
130.3878
130.3148
128.5209
109.0940
93.6269
79.8225
79.5052
79.1844
42.1464
36.9506
36.5860
28.9618
28.3493
24.1014
0.0002
-0.0327
f1 (ppm)

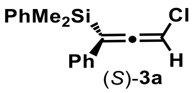

(S)-**3a**

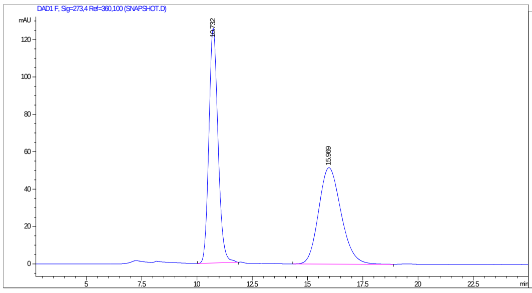

Signal 6: DAD1 F, Sig=273,4 Ref=360,100

Peak #	RetTime [min]	Type	Width [min]	Area [mAU*s]	Height [mAU]	Area %
1	10.732	BB	0.4399	3591.40381	125.50317	49.7873
2	15.969	BB	1.0798	3622.09546	51.54496	50.2127

Totals : 7213.49927 177.04812

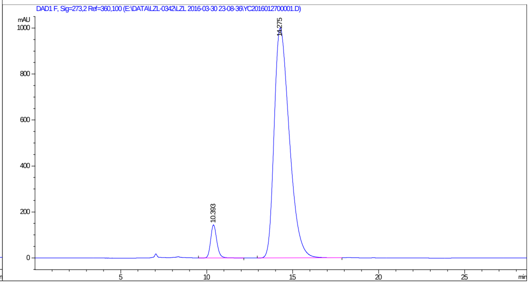

Signal 6: DAD1 F, Sig=273,2 Ref=360,100

Peak #	RetTime [min]	Type	Width [min]	Area [mAU*s]	Height [mAU]	Area %
1	10.393	BB	0.3862	3578.50073	143.85506	5.5179
2	14.275	BB	0.9323	6.12742e4	1000.72412	94.4821

Totals : 6.48527e4 1144.57918

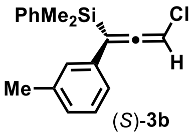

(S)-**3b**

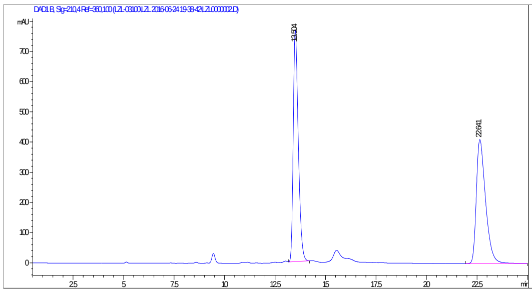

Signal 2: DAD1 B, Sig=210,4 Ref=360,100

Peak #	RetTime [min]	Type	Width [min]	Area [mAU*s]	Height [mAU]	Area %
1	13.504	VB	0.2639	1.32739e4	766.93518	50.4324
2	22.641	BBA	0.4808	1.30463e4	410.49673	49.5676

Totals : 2.63202e4 1177.43192

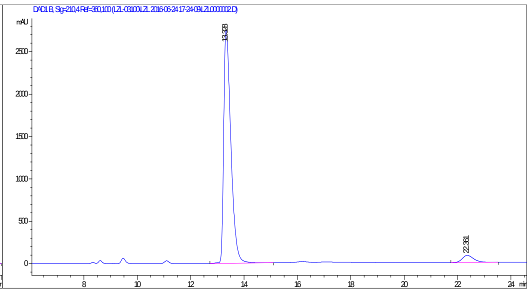

Signal 2: DAD1 B, Sig=210,4 Ref=360,100

Peak #	RetTime [min]	Type	Width [min]	Area [mAU*s]	Height [mAU]	Area %
1	13.328	MM	0.3258	5.41048e4	2767.78076	95.6240
2	22.361	MM	0.4820	2476.00220	85.62308	4.3760

Totals : 5.65808e4 2853.40384

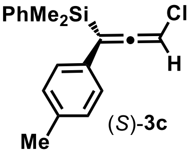

(*S*)-**3c**

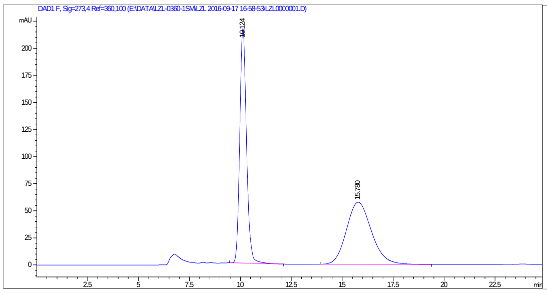

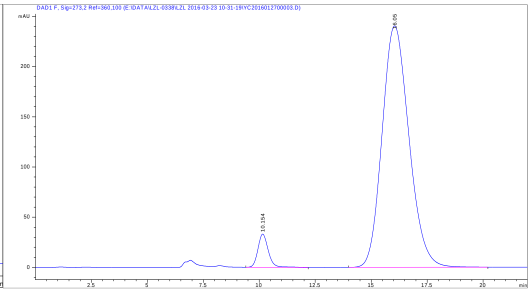

Signal 6: DAD1 F, Sig=273,4 Ref=360,100

Peak #	RetTime [min]	Type	Width [min]	Area [mAU*s]	Height [mAU]	Area %
1	10.124	BB	0.3544	4979.08789	216.41650	50.4753
2	15.780	BB	1.2984	4885.31738	57.31890	49.5247

Totals : 9864.40527 273.73540

Signal 6: DAD1 F, Sig=273,2 Ref=360,100

Peak #	RetTime [min]	Type	Width [min]	Area [mAU*s]	Height [mAU]	Area %
1	10.154	BB	0.4824	1061.86340	33.08468	5.0306
2	16.053	BB	1.0671	2.00462e4	240.33324	94.9694

Totals : 2.11081e4 273.41792

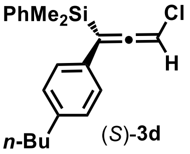

(*S*)-**3d**

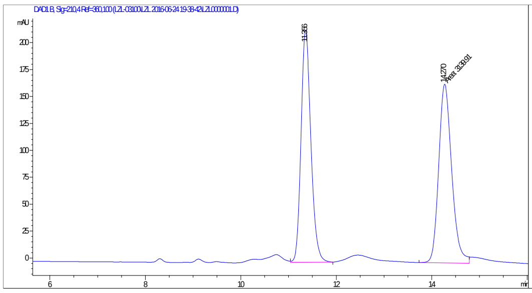

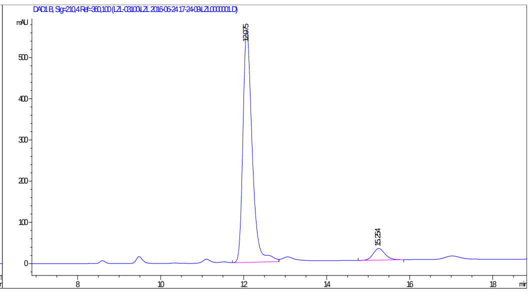

Signal 2: DAD1 B, Sig=210,4 Ref=360,100

Peak #	RetTime [min]	Type	Width [min]	Area [mAU*s]	Height [mAU]	Area %
1	11.356	VB	0.2189	3086.63818	215.75822	49.5722
2	14.270	MF	0.3151	3139.91113	166.08830	50.4278

Totals : 6226.54932 381.84653

Signal 2: DAD1 B, Sig=210,4 Ref=360,100

Peak #	RetTime [min]	Type	Width [min]	Area [mAU*s]	Height [mAU]	Area %
1	12.075	BV	0.2349	8745.37695	563.86841	94.0550
2	15.254	BB	0.3042	552.77844	28.01121	5.9450

Totals : 9298.15540 591.87962

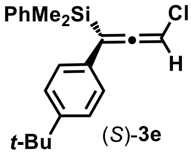

(*S*)-**3e**

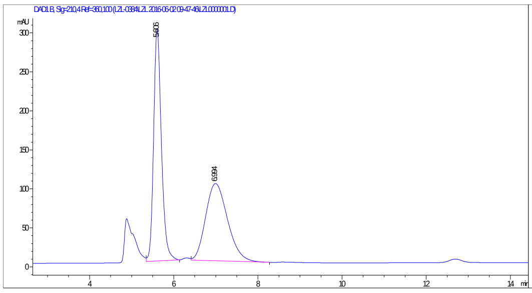

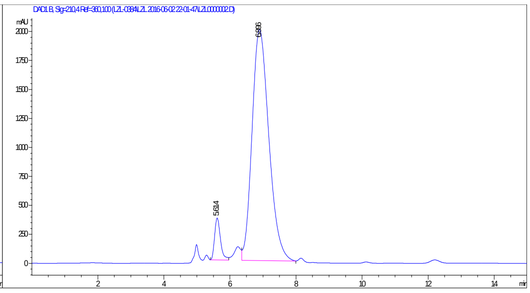

Signal 2: DAD1 B, Sig=210,4 Ref=360,100

Peak #	RetTime [min]	Type	Width [min]	Area [mAU*s]	Height [mAU]	Area %
1	5.606	VB	0.1941	3813.20703	296.42584	51.4836
2	6.994	VB	0.5650	3593.44165	99.11376	48.5164
Totals :				7406.64868	395.53960	

Signal 2: DAD1 B, Sig=210,4 Ref=360,100

Peak #	RetTime [min]	Type	Width [min]	Area [mAU*s]	Height [mAU]	Area %
1	5.614	BV	0.1903	4482.46338	362.29968	5.9778
2	6.896	VB	0.5493	7.05032e4	1990.26440	94.0222
Totals :				7.49856e4	2352.56409	

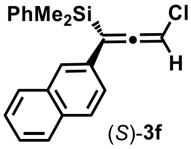

(*S*)-**3f**

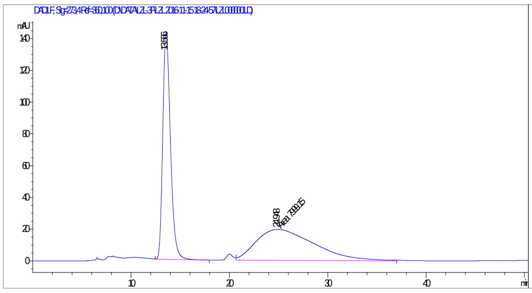

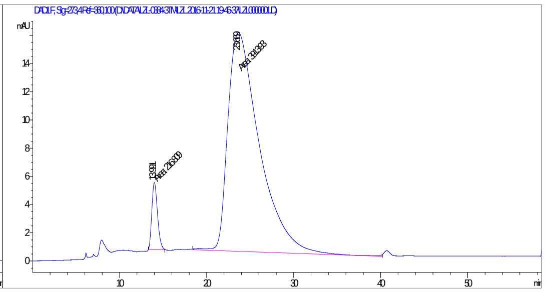

Signal 6: DAD1 F, Sig=273,4 Ref=360,100

Peak #	RetTime [min]	Type	Width [min]	Area [mAU*s]	Height [mAU]	Area %
1	13.566	BB	0.8334	7751.22900	142.77934	49.2130
2	24.948	FM	6.8567	7999.15332	19.44358	50.7870
Totals :				1.57504e4	162.22292	

Signal 6: DAD1 F, Sig=273,4 Ref=360,100

Peak #	RetTime [min]	Type	Width [min]	Area [mAU*s]	Height [mAU]	Area %
1	13.991	MM	0.7562	216.80930	4.77854	5.2487
2	23.669	MF	4.2004	3913.93481	15.53017	94.7513
Totals :				4130.74411	20.30871	

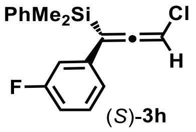

(*S*)-3h

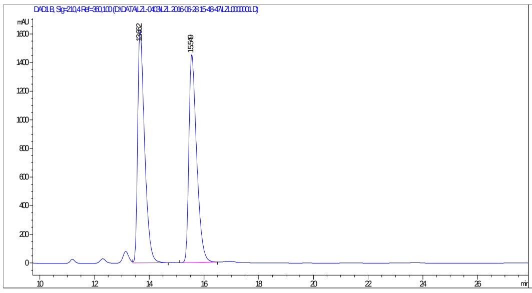

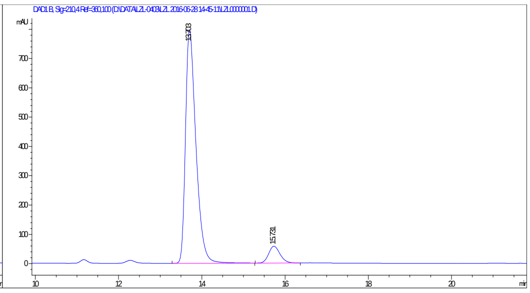

Signal 2: DAD1 B, Sig=210,4 Ref=360,100

Peak #	RetTime [min]	Type	Width [min]	Area [mAU*s]	Height [mAU]	Area %
1	13.662	VB	0.2653	2.86453e4	1628.08557	50.0092
2	15.549	BB	0.2983	2.86348e4	1450.60510	49.9908
Totals :				5.72801e4	3078.69067	

Signal 2: DAD1 B, Sig=210,4 Ref=360,100

Peak #	RetTime [min]	Type	Width [min]	Area [mAU*s]	Height [mAU]	Area %
1	13.703	BB	0.2648	1.38577e4	797.02148	92.6725
2	15.731	BB	0.2935	1095.70715	57.70712	7.3275
Totals :				1.49534e4	854.72861	

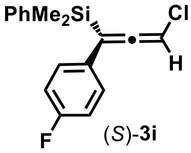

(*S*)-3i

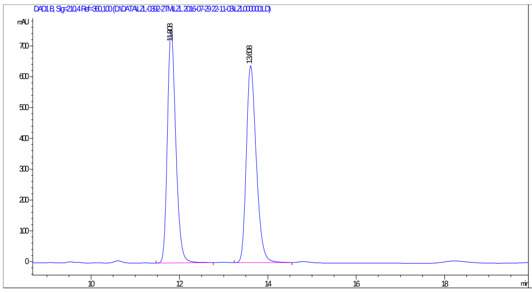

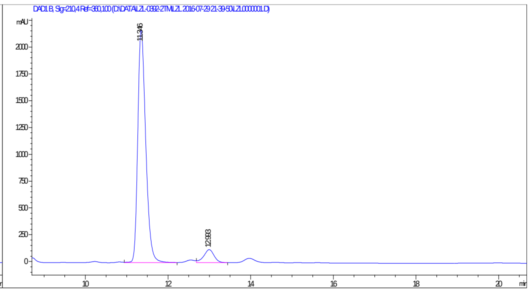

Signal 2: DAD1 B, Sig=210,4 Ref=360,100

Peak #	RetTime [min]	Type	Width [min]	Area [mAU*s]	Height [mAU]	Area %
1	11.808	BB	0.2043	1.00042e4	756.65826	50.0224
2	13.608	BB	0.2406	9995.26074	638.74432	49.9776
Totals :				1.99995e4	1395.40259	

Signal 2: DAD1 B, Sig=210,4 Ref=360,100

Peak #	RetTime [min]	Type	Width [min]	Area [mAU*s]	Height [mAU]	Area %
1	11.346	VB	0.2062	2.90800e4	2171.68506	93.3462
2	12.993	VB	0.2602	2072.83862	120.78619	6.6538
Totals :				3.11529e4	2292.47125	

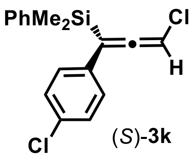

(*S*)-**3k**

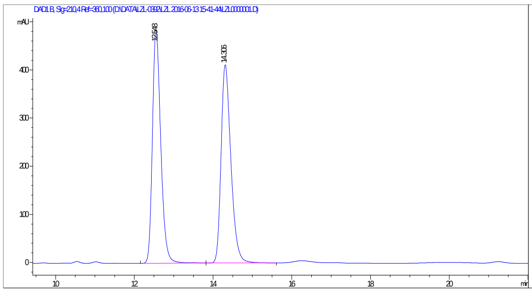

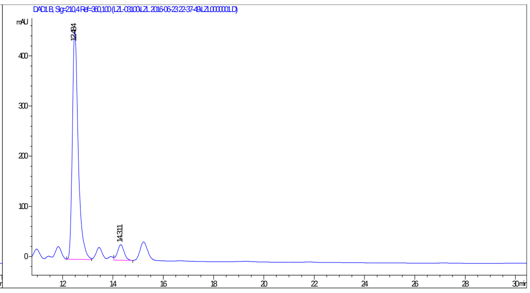

Signal 2: DAD1 B, Sig=210,4 Ref=360,100

Peak #	RetTime [min]	Type	Width [min]	Area [mAU*s]	Height [mAU]	Area %
1	12.548	BB	0.2258	7142.16895	484.89758	50.0441
2	14.306	BB	0.2641	7129.57031	411.59836	49.9559
Totals :				1.42717e4	896.49594	

Signal 2: DAD1 B, Sig=210,4 Ref=360,100

Peak #	RetTime [min]	Type	Width [min]	Area [mAU*s]	Height [mAU]	Area %
1	12.484	BV	0.2433	7428.21875	457.80365	92.9746
2	14.311	VB	0.2794	561.29901	30.97358	7.0254
Totals :				7989.51776	488.77723	

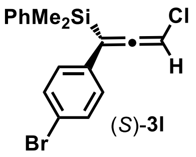

(*S*)-**3l**

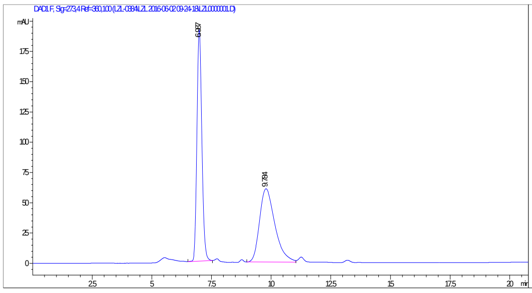

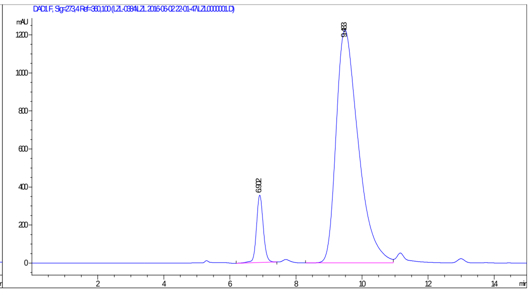

Signal 6: DAD1 F, Sig=273,4 Ref=360,100

Peak #	RetTime [min]	Type	Width [min]	Area [mAU*s]	Height [mAU]	Area %
1	6.987	BB	0.2199	2717.19019	191.09383	50.1123
2	9.784	BV	0.6854	2705.01660	60.52993	49.8877
Totals :				5422.20679	251.62375	

Signal 6: DAD1 F, Sig=273,4 Ref=360,100

Peak #	RetTime [min]	Type	Width [min]	Area [mAU*s]	Height [mAU]	Area %
1	6.902	BB	0.2209	5075.59766	354.83728	8.1639
2	9.483	BV	0.6992	5.70957e4	1226.25073	91.8361
Totals :				6.21713e4	1581.08801	

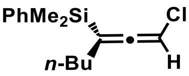

(*S*)-**3m**

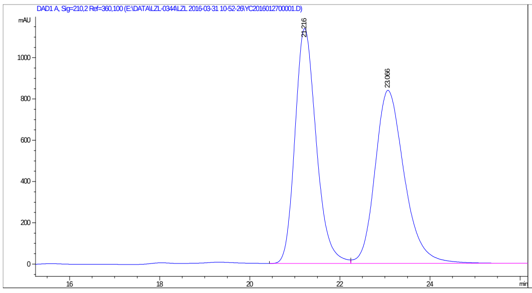

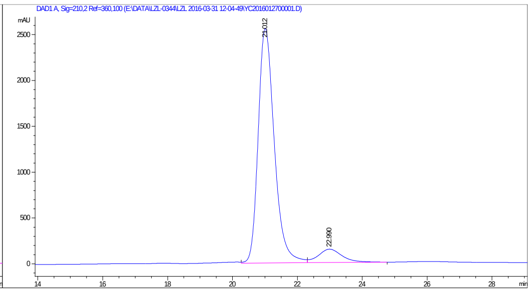

Signal 1: DAD1 A, Sig=210,2 Ref=360,100

Peak #	RetTime [min]	Type	Width [min]	Area [mAU*s]	Height [mAU]	Area %
1	21.216	BV	0.5166	3.73452e4	1138.87988	49.6501
2	23.066	VBA	0.6844	3.78716e4	839.37390	50.3499
Totals :				7.52168e4	1978.25378	

Signal 1: DAD1 A, Sig=210,2 Ref=360,100

Peak #	RetTime [min]	Type	Width [min]	Area [mAU*s]	Height [mAU]	Area %
1	21.012	VV	0.5188	8.82884e4	2557.44604	92.2333
2	22.990	VB	0.7542	7434.56201	145.96889	7.7667
Totals :				9.57230e4	2703.41493	

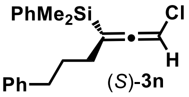

(*S*)-**3n**

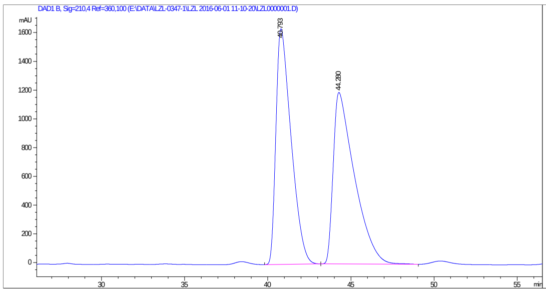

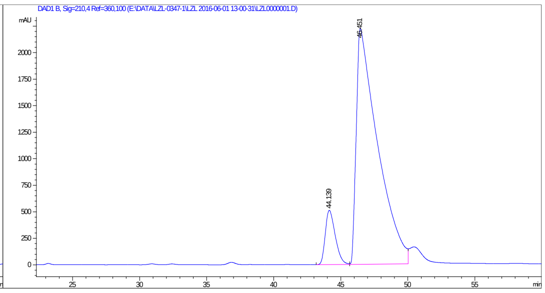

Signal 2: DAD1 B, Sig=210,4 Ref=360,100

Peak #	RetTime [min]	Type	Width [min]	Area [mAU*s]	Height [mAU]	Area %
1	40.793	BB	0.9402	1.03974e5	1638.86658	49.5261
2	44.280	BB	1.2463	1.05964e5	1192.88843	50.4739
Totals :				2.09938e5	2831.75500	

Signal 2: DAD1 B, Sig=210,4 Ref=360,100

Peak #	RetTime [min]	Type	Width [min]	Area [mAU*s]	Height [mAU]	Area %
1	44.139	BV	0.7887	2.64267e4	511.59106	9.7186
2	46.451	VV	1.4307	2.45493e5	2219.03320	90.2814
Totals :				2.71919e5	2730.62427	

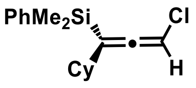

(*S*)-**3p**

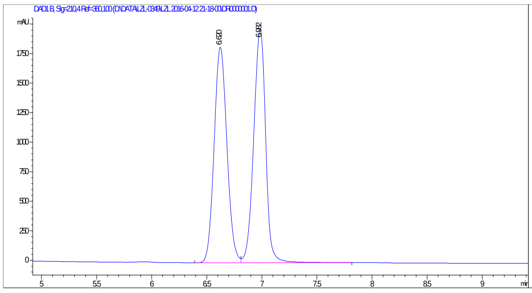

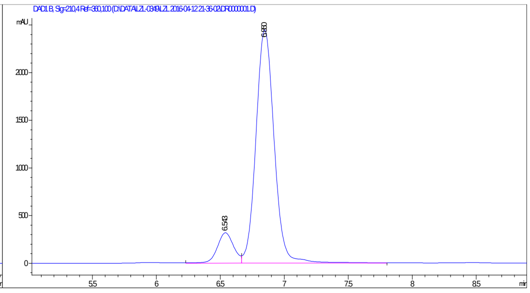

Signal 2: DAD1 B, Sig=210,4 Ref=360,100

Peak #	RetTime [min]	Type	Width [min]	Area [mAU*s]	Height [mAU]	Area %
1	6.620	BV	0.1261	1.47516e4	1828.96484	49.7436
2	6.982	VB	0.1180	1.49036e4	1974.97961	50.2564
Totals :				2.96552e4	3803.94446	

Signal 2: DAD1 B, Sig=210,4 Ref=360,100

Peak #	RetTime [min]	Type	Width [min]	Area [mAU*s]	Height [mAU]	Area %
1	6.543	VV	0.1348	2813.73413	319.26279	10.3824
2	6.850	VV	0.1537	2.42873e4	2449.73193	89.6176
Totals :				2.71010e4	2768.99472	

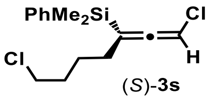

(*S*)-**3s**

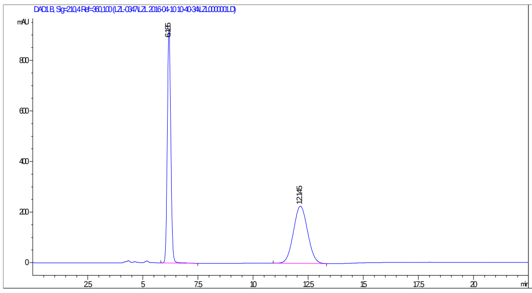

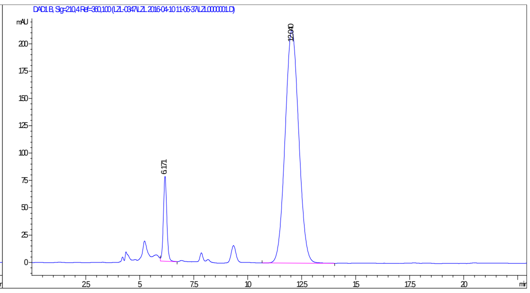

Signal 2: DAD1 B, Sig=210,4 Ref=360,100

Peak #	RetTime [min]	Type	Width [min]	Area [mAU*s]	Height [mAU]	Area %
1	6.185	BB	0.1720	1.01137e4	922.02484	50.0180
2	12.145	BB	0.6997	1.01064e4	226.02942	49.9820
Totals :				2.02201e4	1148.05426	

Signal 2: DAD1 B, Sig=210,4 Ref=360,100

Peak #	RetTime [min]	Type	Width [min]	Area [mAU*s]	Height [mAU]	Area %
1	6.171	VB	0.1710	858.67053	77.63022	8.4166
2	12.040	BB	0.6853	9343.46484	213.21170	91.5834
Totals :				1.02021e4	290.84192	

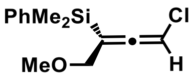

(*S*)-**3t**

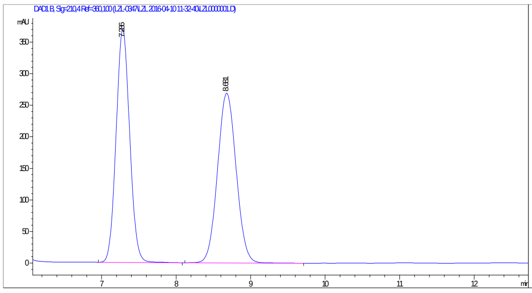

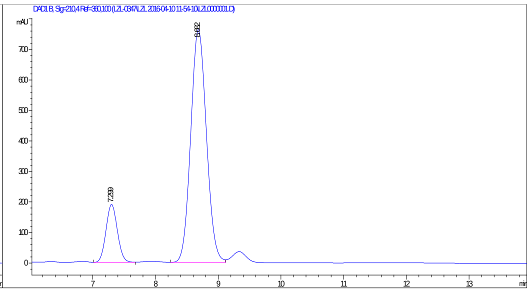

Signal 2: DAD1 B, Sig=210,4 Ref=360,100

Peak #	RetTime [min]	Type	Width [min]	Area [mAU*s]	Height [mAU]	Area %
1	7.286	BB	0.1981	4682.50098	368.83368	50.0304
2	8.681	BB	0.2708	4676.80371	268.99612	49.9696

Totals : 9359.30469 637.82980

Signal 2: DAD1 B, Sig=210,4 Ref=360,100

Peak #	RetTime [min]	Type	Width [min]	Area [mAU*s]	Height [mAU]	Area %
1	7.299	BV	0.1972	2395.32202	189.80386	15.2877
2	8.682	BV	0.2709	1.32730e4	763.28540	84.7123

Totals : 1.56683e4 953.08926

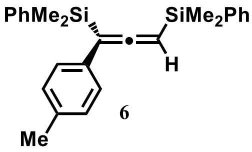

6

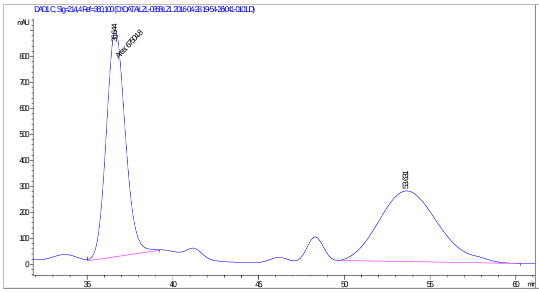

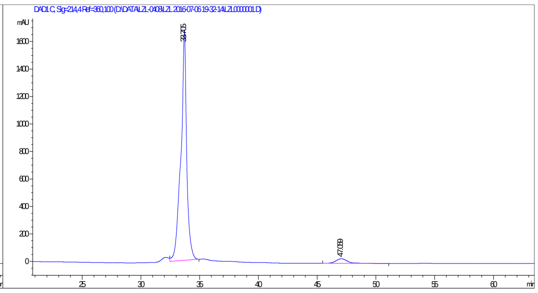

Signal 3: DAD1 C, Sig=214,4 Ref=360,100

Peak #	RetTime [min]	Type	Width [min]	Area [mAU*s]	Height [mAU]	Area %
1	36.644	MF	1.3214	7.04392e4	888.44092	51.9406
2	53.631	BB	3.4392	6.51756e4	271.83408	48.0594

Totals : 1.35615e5 1160.27499

Signal 3: DAD1 C, Sig=214,4 Ref=360,100

Peak #	RetTime [min]	Type	Width [min]	Area [mAU*s]	Height [mAU]	Area %
1	33.705	VB	0.4654	5.76402e4	1675.46606	96.2515
2	47.059	BB	1.0501	2244.77319	33.23087	3.7485

Totals : 5.98850e4 1708.69693

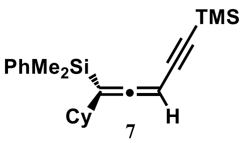

7

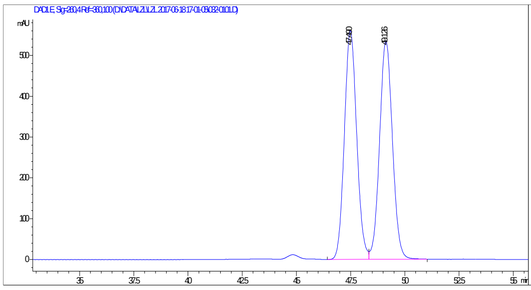

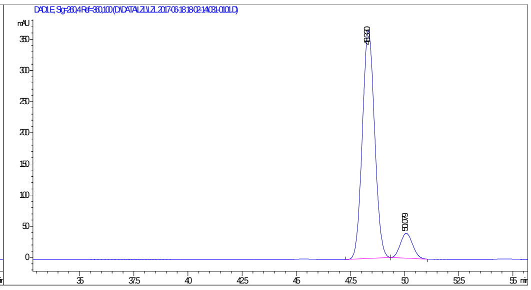

Signal 5: DAD1 E, Sig=260,4 Ref=360,100

Peak #	RetTime [min]	Type	Width [min]	Area [mAU*s]	Height [mAU]	Area %
1	47.490	BV	0.6046	2.21081e4	559.93231	50.0481
2	49.126	VB	0.5930	2.20656e4	535.21979	49.9519

Totals : 4.41737e4 1095.15210

Signal 5: DAD1 E, Sig=260,4 Ref=360,100

Peak #	RetTime [min]	Type	Width [min]	Area [mAU*s]	Height [mAU]	Area %
1	48.330	BB	0.6058	1.43922e4	365.14581	90.1630
2	50.079	BB	0.6060	1570.22803	39.82764	9.8370

Totals : 1.59625e4 404.97345

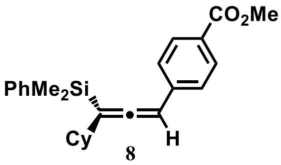

8

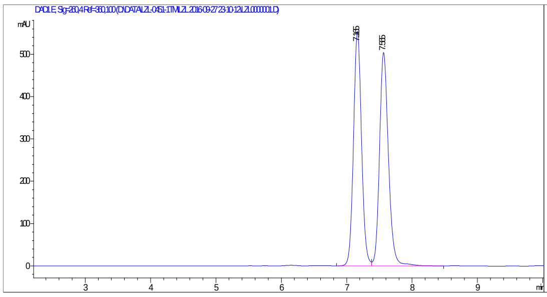

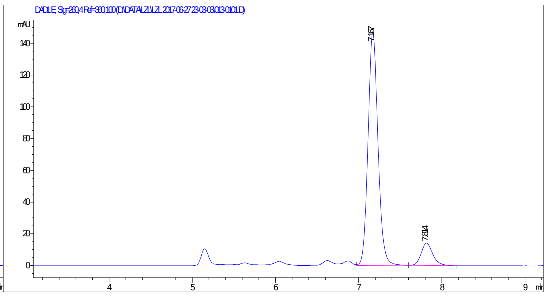

Signal 5: DAD1 E, Sig=260,4 Ref=360,100

Peak #	RetTime [min]	Type	Width [min]	Area [mAU*s]	Height [mAU]	Area %
1	7.165	BV	0.1317	4733.23047	553.94714	49.6984
2	7.565	VB	0.1448	4790.68701	504.41898	50.3016

Totals : 9523.91748 1058.36612

Signal 4: DAD1 E, Sig=260,4 Ref=360,100

Peak #	RetTime [min]	Type	Width [min]	Area [mAU*s]	Height [mAU]	Area %
1	7.167	VB	0.1285	1216.48901	147.12424	89.9513
2	7.814	BB	0.1489	135.89771	14.04717	10.0487

Totals : 1352.38672 161.17141

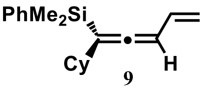

9

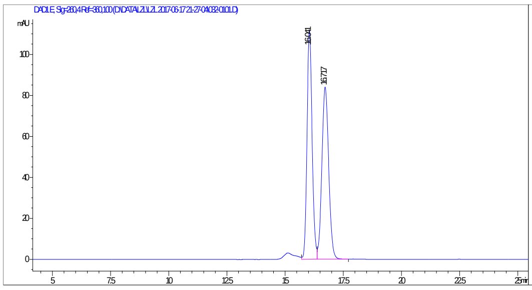

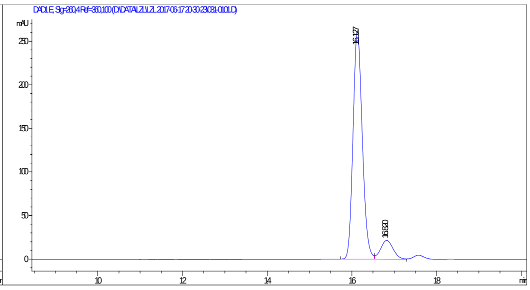

Signal 5: DAD1 E, Sig=260,4 Ref=360,100

Peak #	RetTime [min]	Type	Width [min]	Area [mAU*s]	Height [mAU]	Area %
1	16.041	VV	0.2359	1701.91345	111.58607	49.8030
2	16.717	VB	0.3161	1715.37744	84.05329	50.1970
Totals :				3417.29089	195.63937	

Signal 5: DAD1 E, Sig=260,4 Ref=360,100

Peak #	RetTime [min]	Type	Width [min]	Area [mAU*s]	Height [mAU]	Area %
1	16.127	BV	0.2371	3977.01440	261.97821	90.0135
2	16.820	VV	0.3168	441.22870	21.55248	9.9865
Totals :				4418.24310	283.53069	

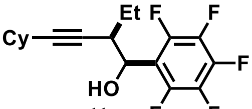

11

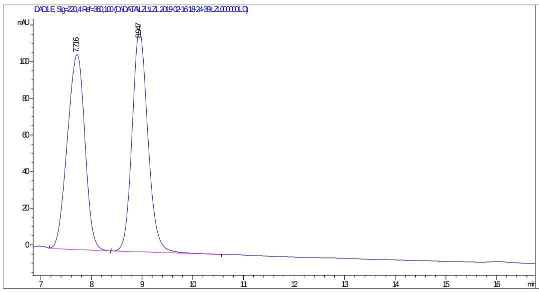

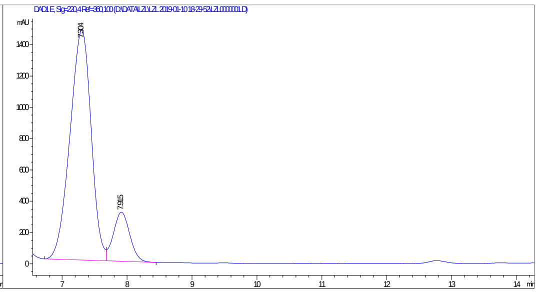

Signal 5: DAD1 E, Sig=220,4 Ref=360,100

Peak #	RetTime [min]	Type	Width [min]	Area [mAU*s]	Height [mAU]	Area %
1	7.716	VB	0.3851	2564.62891	106.43555	49.4731
2	8.947	BB	0.3327	2619.25781	120.93251	50.5269
Totals :				5183.88672	227.36806	

Signal 5: DAD1 E, Sig=220,4 Ref=360,100

Peak #	RetTime [min]	Type	Width [min]	Area [mAU*s]	Height [mAU]	Area %
1	7.304	BV	0.3547	3.33212e4	1468.88794	85.7893
2	7.915	VB	0.2730	5519.54688	314.13483	14.2107
Totals :				3.88407e4	1783.02277	

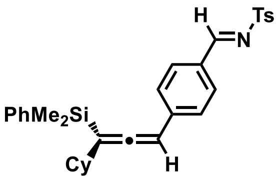

(*E*, *S*)-**14**

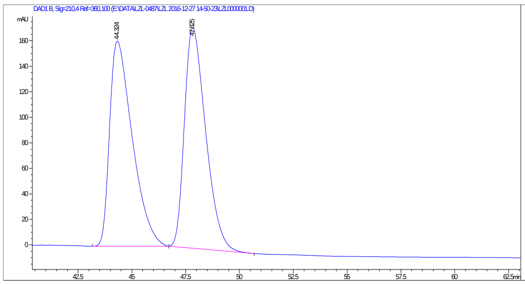

Signal 2: DAD1 B, Sig=210,4 Ref=360,100

Peak #	RetTime [min]	Type	Width [min]	Area [mAU*s]	Height [mAU]	Area %
1	44.324	BB	1.1011	1.17833e4	160.76486	49.7724
2	47.825	BB	1.0527	1.18910e4	172.81844	50.2276
Totals :				2.36743e4	333.58330	

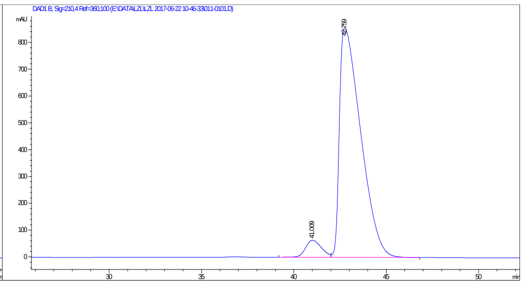

Signal 2: DAD1 B, Sig=210,4 Ref=360,100

Peak #	RetTime [min]	Type	Width [min]	Area [mAU*s]	Height [mAU]	Area %
1	41.009	BV	0.9078	3784.01025	63.44725	5.2536
2	42.759	VB	1.1437	6.82433e4	856.37207	94.7464
Totals :				7.20273e4	919.81932	

12 Crystal Data for (*E*, *S*)-14.

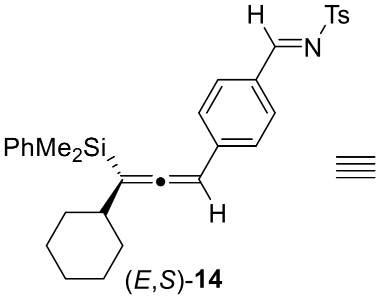

(*E*,*S*)-**14** ≡

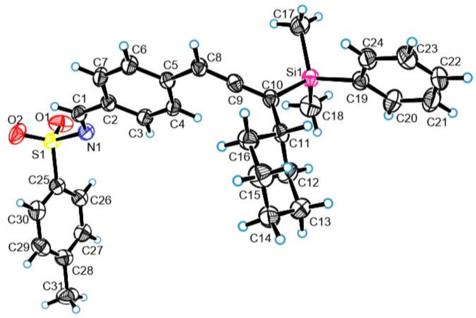

**Table Crystal data and structure refinement for lzl-86.**	
Identification code	lzl-86
Empirical formula	$C_{31}H_{35}NO_2SSi$
Formula weight	513.75
Temperature/K	290(2)
Crystal system	monoclinic
Space group	C2
a/Å	12.12950(10)
b/Å	13.86570(10)
c/Å	17.2627(2)
α / °	90
β / °	104.7730(10)
γ / °	90
Volume/Å^3	2807.34(5)
Z	4
ρ_{calc}g/cm^3	1.216
μ /mm^{-1}	1.643
F(000)	1096.0
Crystal size/mm^3	0.230 × 0.220 × 0.150
Radiation	CuKα (λ = 1.54184)
2Θ range for data collection/°	9.878 to 142.544
Index ranges	-14 ≤ h ≤ 14, -16 ≤ k ≤ 16, -18 ≤ l ≤ 20
Reflections collected	10139
Independent reflections	5264 [R_{int} = 0.0265, R_{sigma} = 0.0328]
Data/restraints/parameters	5264/1/328
Goodness-of-fit on F^2	1.029
Final R indexes [I>=2σ (I)]	R_1 = 0.0350, wR_2 = 0.0938
Final R indexes [all data]	R_1 = 0.0362, wR_2 = 0.0955
Largest diff. peak/hole / e Å^{-3}	0.28/-0.15
Flack parameter	-0.011(11)
